# Collective Total Synthesis of Casbane Diterpenes: One Strategy, Multiple Targets

**DOI:** 10.1002/anie.202015243

**Published:** 2021-01-28

**Authors:** Lorenz E. Löffler, Conny Wirtz, Alois Fürstner

**Affiliations:** ^1^ Max-Planck-Institut für Kohlenforschung 45470 Mülheim/Ruhr Germany

**Keywords:** alkyne metathesis, cyclopropanes, hydrostannation, macrocycles, terpenes

## Abstract

Of the more than 100 casbane diterpenes known to date, only the eponymous parent hydrocarbon casbene itself has ever been targeted by chemical synthesis. Outlined herein is a conceptually new approach that brings not a single but a variety of casbane derivatives into reach, especially the more highly oxygenated and arguably more relevant members of this family. The key design elements are a catalyst‐controlled intramolecular cyclopropanation with or without subsequent equilibration, chain extension of the resulting stereoisomeric cyclopropane building blocks by chemoselective hydroboration/cross‐coupling, and the efficient closure of the strained macrobicyclic framework by ring‐closing alkyne metathesis. A hydroxy‐directed catalytic *trans*‐hydrostannation allows for late‐stage diversity. These virtues are manifested in the concise total syntheses of depressin, yuexiandajisu A, and *ent*‐pekinenin C. The last compound turned out to be identical to euphorhylonal A, the structure of which had clearly been misassigned.

## Graphical abstract

A cornucopia: Sessile soft corals as well as certain higher plants produce a multitude of casbane diterpenes with different oxygenation patterns. A new synthetic approach to this family of natural products (see structural framework) was designed that allows a large subset to be encompassed, yet is short, efficient, and selective. Key to success was a combination of catalytic cyclopropanation, alkyne metathesis, and *trans*‐hydrometalation chemistry.

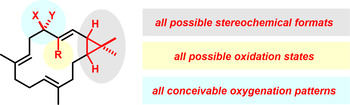

## Introduction

The bicyclo[12.1.0]pentadecane framework of the casbane diterpenes represents the formal progenitor of the more complex polycyclic skeletons of the lathyrane, jatrophane, tigliane, daphnane and ingenane families.[^1^, ^2^] Despite their role as a central node within this large biosynthetic network, casbane derivatives themselves are rare: the flowering plants of the genus *Euphorbia* and the biologically totally unrelated soft corals of the genus *Sinularia* are by far the major sources. From this small number of producing organisms, however, more than 100 different structural variants have been isolated to date, which feature a bewildering diversity of oxygenation patterns and stereochemical permutations along the hydrocarbon rim.

**Figure 1:**
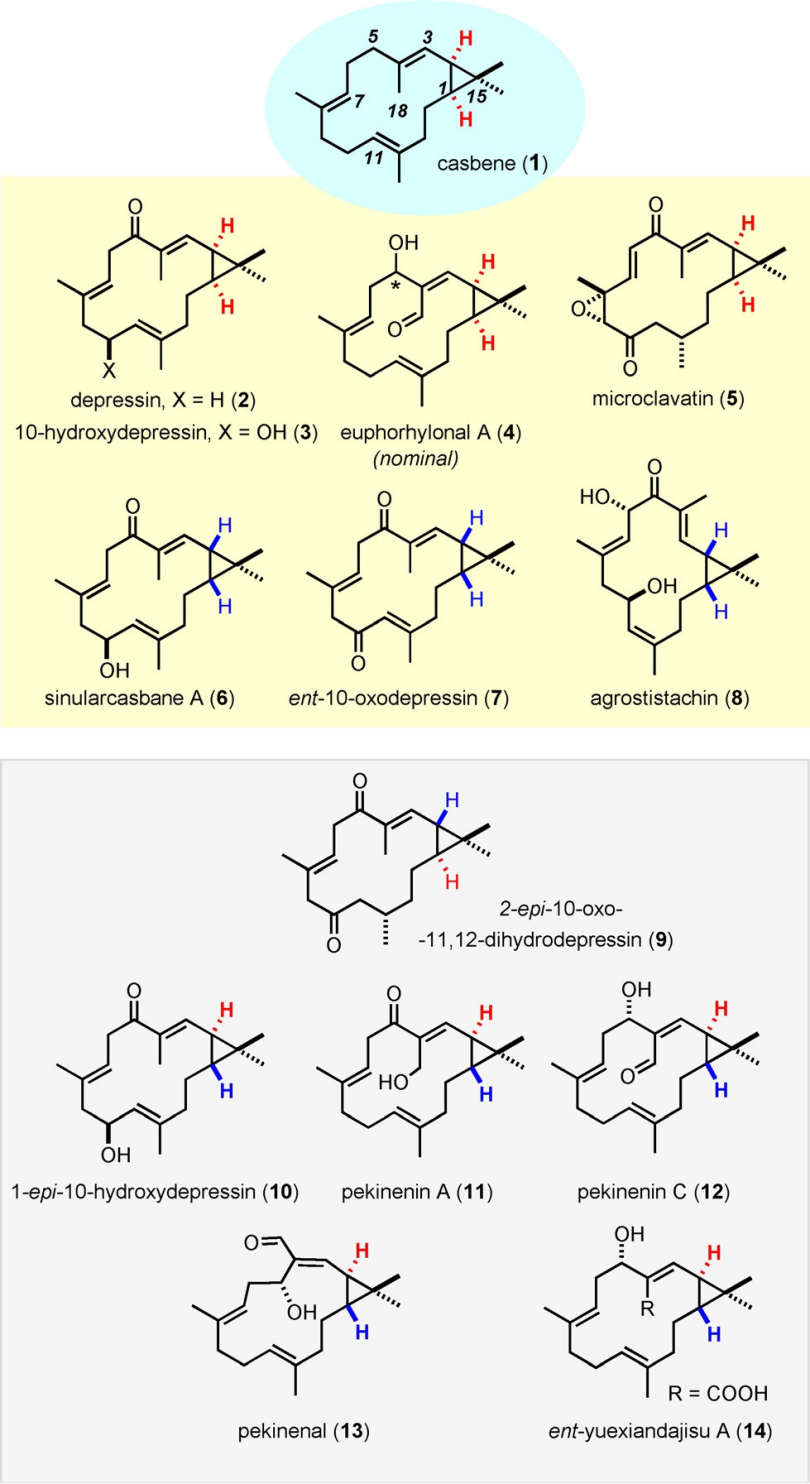
Selection of naturally occurring casbane diterpenes.

The few examples shown in Figure 1 are representative. Most naturally occurring casbane diterpenes comprise a *cis*‐disubstituted cyclopropane unit, which appears in both antipodal formats (compare the depressin derivatives **2**, **3** ^[3]^ with the “pseudoenantiomeric” compounds **6**,^[4]^ **7** ^[5]^). The same is true for the *trans*‐cyclopropane series, as illustrated by **9** and **10**, which are isomeric even though they derive from the same soft coral.^[3]^ It is of note, however, that the absolute configuration of several casbanes was either not addressed by the isolation teams or has only been inferred by plausibility arguments, especially in the older literature. Another common structural variation concerns the oxygenation pattern of the “northern” sector, in that the C5 methylene group is frequently oxidized to the alcohol or ketone level, with or without concomitant oxygenation of the surrounding positions. Once again, the stereochemistry is evidently not uniform as the comparison of **12** ^[6]^ and **13** ^[7]^ suggests, which supposedly have opposite configurations at C5 (in other cases such as **4**,^[8]^ the configuration of this center is simply unknown). Moreover, the parent C18 methyl substituent can be transformed to the alcohol (**11**),^[6]^ aldehyde (**4**,^[8]^ **12**,^[6]^ **13** ^[7]^) or carboxylic acid level (e.g. yuexiandajisu A, which is either **14** or *ent*‐**14**).^[9]^ Additional diversity is gained by isomerization (**8**,^[10]^ **13** ^[7]^), (partial) saturation (**9**)^[3]^ and/or epoxidation (**5**)^[11]^ of the trisubstituted alkenes and/or by additional oxygenation of the “lower” sector of the constituent framework.^[12]^

The fact that casbane diterpenes were evolved by sessile marine sponges and, in parallel, by higher plants is fascinating and suggestive at the same time. In this context, it is worth remembering that *Euphorbia* plants (the “spurge” family) are commonly considered toxic; some of them play important roles in traditional Chinese medicine (“*Lang Du*”) for the treatment of various ailments.^[13]^ In line with this notion, pekinenin C (**12**) was shown to cause cell cycle arrest in the G0/G1 phase and apoptotic cell death via the mitochondrial as well as the death‐receptor pathways.^[14]^ Equally noteworthy is the role of certain casbanes as phytoalexins: while the amount of **1** in seedlings of the castor bean (*Rhicinus communis*) is minute,^[15]^ exposure to certain fungi massively upregulates its biosynthesis as part of the plant's defense mechanism.[^16^, ^17^] Along the same lines, *ent*‐10‐oxodepressin (**7**) as a prototype member of the “enantiomeric” series was recognized as phytoalexin of rice.^[5]^ This report also marks a rare case of a casbane derivative isolated from a higher plant not belonging to the *Euphorbia* genus; in view of the importance of rice for the sustention of humankind, the discovery of the potential fungicidal properties of **7** is arguably relevant and warrants further scrutiny.

The increasing awareness of the possible biological roles of the casbane diterpenes is not yet reflected in advanced synthetic studies. So far, the eponymous hydrocarbon **1** is the only member that has been targeted in the past: five independent chemical syntheses and an enzymatic approach were published prior to the turn of the millennium;[^18^, ^19^, ^20^, ^21^, ^22^, ^23^] all activity seems to have ceased since then. Although a detailed analysis of this prior art is beyond the scope of this Research Article, it is noteworthy that all approaches—except for the enzymatic route^[23]^—struggled with the macrocyclization step, which was low yielding in most cases. What is more, either the ring closure itself or the necessary functional group manipulations in its aftermath invariably led to isomer mixtures that required tedious separation. Any new foray must address these issues; most importantly, positional and/or configurational double bond isomerism should be strictly avoided. Since the known chemical syntheses gave racemic **1** unless the “chiral pool” was tapped (*cis*‐chrysanthemic acid, (+)‐2‐carene),^[24]^ it is also appropriate and timely to envisage a catalytic enantioselective route. In recognition of the fact that some of the oxygenated casbane derivatives are of larger biological significance than the parent compound itself, a high level of synthetic flexibility is also deemed necessary; from the conceptual viewpoint, any (late‐stage) diversity as integral part of the synthesis blueprint would mark a notable strategic advance.[^25^, ^26^, ^27^]

## Results and Discussion

**Scheme 1:**
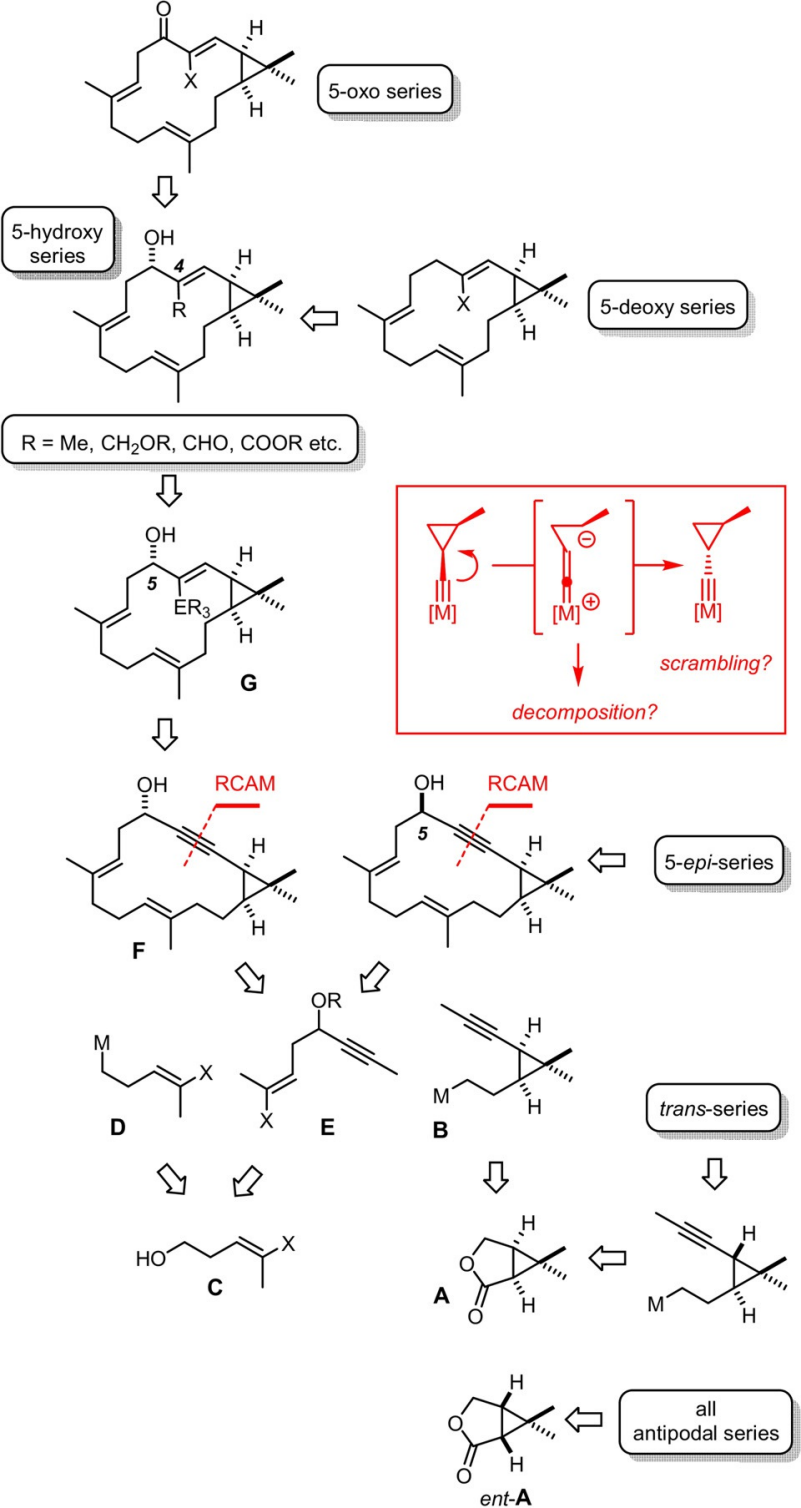
Diversity‐oriented retrosynthetic analysis.

We conjectured that the *trans*‐hydrometalation of internal alkynes, which has recently become a focal point of research in our laboratory,^[28]^ provides valuable opportunities in this context (Scheme 1). Specifically, treatment of propargyl alcohols with R_3_E−H (E=Si, Ge, Sn) in the presence of [Cp*RuCl]_4_ as catalyst follows a stereochemically unorthodox *trans*‐addition mode and delivers the R_3_E− group with high fidelity to the proximal C atom of the former triple bond. This outcome is mechanistically well understood; in essence, it originates from the cooperativity between the unprotected −OH group of the substrate and the polarized [Ru−Cl] unit of the catalyst.^[29]^ When applied to a cycloalkyne of type **F**, the resulting alkenylstannane (germane, silane) **G** should allow either a methyl, a hydroxymethyl, a formyl, or a carboxyl group to be stitched to the C4 position of the casbane framework where such variation is common (see above). Since the directing C5−OH group can either be kept as such or be oxidized to the corresponding ketone (or even be removed), all basic oxygenation patterns in the “northern” sector should be accessible from a single platform. Cycloalkyne **F**, in turn, might be forged by ring closing alkyne metathesis (RCAM):[^30^, ^31^, ^32^] since the best available catalysts activate triple bonds exclusively while leaving all sorts of double bonds untouched, the integrity of the trisubstituted alkenes punctuating the carbon skeleton of **F** seems guaranteed. The same, however, cannot be taken for granted for the cyclopropyl group: any alkyne metathesis catalyst is a Schrock alkylidyne, which is nucleophilic at carbon.^[33]^ This inherent polarization might entail (reversible) ring opening and/or decomposition, especially if forcing conditions are needed (see the Insert in Scheme 1): this is likely to be the case in the projected application given the strain of the resulting 14‐membered ring **F** that incorporates two *E*‐configured alkenes as well as the linear alkyne; the annelated cyclopropyl ring rigidifies the skeleton even further. Therefore, an application to the casbane series will also clarify an as yet unknown aspect of alkyne metathesis chemistry in general. Further analysis reveals that the building block **C** can be used twice en route to **F**, which reduces the synthetic exertion and helps render the route practical. The cyclopropyl fragment **B** can be traced back to lactone **A**, which is available by rhodium catalyzed intramolecular cyclopropanation:[^34^, ^35^] since this step proceeds under catalyst control and the elaboration of **A** into **B** can be performed with or without epimerization of a derived aldehyde intermediate, all possible isomers are accessible. The blueprint shown in Scheme 1 hence combines flexibility at the outset with late‐stage diversity and should therefore bring a considerable number of casbane derivatives into reach.

**Scheme 2:**
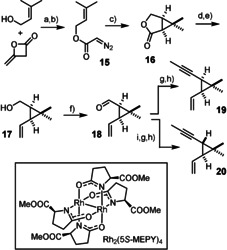
a) NaOAc, THF, reflux, 70 %; b) (i) *p*‐acetamidobenzenesulfonyl azide, Et_3_N, MeCN; (ii) LiOH, H_2_O, 75 %; c) [Rh_2_(5*S*‐MEPY)_4_]⋅(MeCN)_2_ (0.6 mol %), CH_2_Cl_2_, reflux, 87 %, 93 % *ee*; d) Dibal‐H, CH_2_Cl_2_, −78 °C; e) Ph_3_P=CH_2_, THF, 0 °C→RT, 55 % (over both steps); f) DMP, CH_2_Cl_2_, 0 °C→RT; g) CBr_4_, PPh_3_, CH_2_Cl_2_, 0 °C; h) *n*BuLi, Et_2_O/DMPU, then MeI, −78 °C→RT, 51 % (**19**, over three steps), 63 % (**20**, over four steps, *cis*/*trans*=1:9); i) K_2_CO_3_, MeOH, 50 °C; Dibal‐H=diisobutylaluminum hydride, DMP=Dess–Martin periodinane, DMPU=*N*,*N*′‐dimethylpropyleneurea.

In the forward sense, diazoester **15** was prepared by a literature route from prenyl alcohol and diketene followed by diazo transfer (Scheme 2); its treatment with catalytic Rh_2_(*S*‐MEPY)_4_ furnished multigram amounts of **16** with high optical purity (93 % *ee*).[^34^, ^35^] After reduction of the lactone with Dibal‐H in CH_2_Cl_2_ at low temperature, the resulting crude lactol was subjected to Wittig olefination to give alcohol **17**,^[36]^ which was then oxidized to the corresponding aldehyde **18**. It is at this point that the synthesis route bifurcates for the first time: when **18** was subjected to regular Corey/Fuchs alkynylation^[37]^ and C‐methylation under optimized conditions,^[38]^ the *cis*‐configured enyne **19** was obtained. Stirring of a solution of **18** in MeOH with K_2_CO_3_, however, causes equilibration with formation of the thermodynamically more stable *trans* isomer and hence opens access to the epimeric *trans*‐configured building block **20** (dr ≥9:1).^[39]^ The somewhat modest yields of **19** and **20** solely reflect the high volatility of these hydrocarbons. The enantiomeric compounds are obviously equally well accessible.^[40]^

**Scheme 3:**
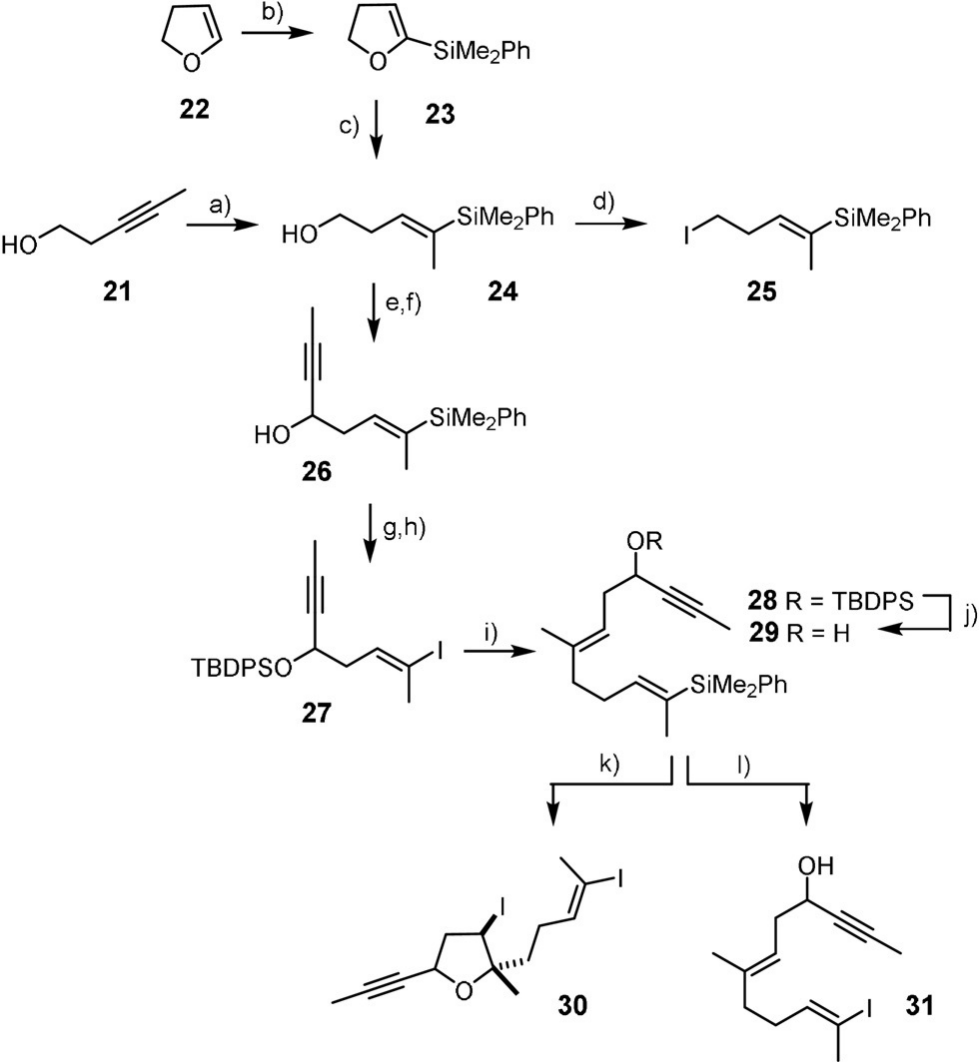
a) (i) *n*BuLi, THF, −78 °C→−30 °C; (ii) (PhMe_2_Si)_2_Cu(CN)Li_2_, −78 °C, 90 %; b) *n*BuLi, PhMe_2_SiCl, THF, −30 °C→RT, quant.; c) [(PPh_3_)_2_NiCl_2_] (8 mol %), MeMgBr, toluene, reflux, 91 %; d) I_2_, PPh_3_, imidazole, CH_2_Cl_2_, 0 °C→RT, 93 %; e) DMP, CH_2_Cl_2_, 0 °C→RT; f) MeC≡CMgBr, THF, 0 °C, 78 % (over both steps); g) TBDPSCl, imidazole, CH_2_Cl_2_/DMF, 84 %; h) NIS, 2,6‐lutidine, HFIP, CH_2_Cl_2_, −20 °C, 89 %; i) (i) **25**, Zn, LiCl, TMSCl, 1,2‐diiodoethane, THF; ii) Pd(PPh_3_)_4_ (6 mol %), 82 %; j) TBAF, THF, 0 °C→RT, 84 %; k) NIS, HFIP, 0 °C, 73 % (dr=42:58); l) NIS, HFIP, HOAc, 0 °C, 70 %; DMF=*N*,*N*‐dimethylformamide, HFIP=hexafluoroisopropanol, NIS=*N*‐iodosuccinimide, TBAF=tetra‐*n*‐butylammonium fluoride, TBDPS=*tert*‐butyldiphenylsilyl.

Fragment **24** to be used twice during the synthesis was readily obtained on treatment of the lithium salt of 3‐pentyn‐1‐ol (**21**) with the cyanocuprate derived from PhMe_2_SiLi and CuCN (Scheme 3).^[41]^ Alternatively, this very same product can be made from 2,3‐dihydrofuran **22** by metalation/silylation followed by nickel catalyzed opening of the resulting compound **23** with MeMgBr.^[42]^ Whereas the first method works well on a ≤3 g scale and takes a single step, the two‐step procedure is more practical when it comes to making much larger quantities. Oxidation of **24** to the corresponding aldehyde followed by addition of propynylmagnesium bromide gave **26**. Although it would be fairly straightforward to set this propargyl alcohol center in isomerically pure form,^[43]^ no effort in this direction was undertaken at this stage: in view of the uncertainty concerning the configurational assignment of the C5−OH group in various casbane derivatives, access to both isomers was probably necessary anyway. Compound **26** was then O‐silylated prior to iododesilylation on reaction with NIS in CH_2_Cl_2_ at −20 °C in the presence of hexafluoroisopropanol (HFIP) and 2,6‐lutidine following a procedure developed by Zakarian and co‐workers.^[44]^ Under these conditions, the yield of alkenyl iodide **27** was high and no sign of double bond isomerization was observed within the limits of detection (*E*:*Z* ≥95:5, ^1^H NMR). This product was then subjected to a palladium catalyzed Negishi coupling^[45]^ with the organozinc reagent derived from iodide **25**,^[46]^ which also derives from **24**. Somewhat unexpectedly, however, the iododesilylation of the resulting product **28** did not proceed well under the conditions that had worked so smoothly for the shorter building block **26**;^[44]^ several alternative methods were also tried but equally found low‐yielding, erratic, or unselective.^[47]^ Since (inseparable) isomer mixtures need to be strictly avoided for the sake of practicality, considerable optimization was necessary at this stage. Part of the problem is the poor solubility of **28** in the polar medium comprised of HFIP as (co)solvent. Therefore we considered the cleavage of the TBDPS ether *prior* to iododesilylation, even though this order of the events bears risk and has hardly any precedent:^[48]^ upon addition of an [I^+^] source to the resulting polyunsaturated substrate **29**, the unprotected −OH group might interfere by attacking any transient iodonium ion and thus entail iodoetherification. This was indeed observed when the reaction was carried out with NIS in HFIP: the cyclic ether **30** was formed as a mixture of two diastereomers. Addition of HOAc,^[49]^ however, allowed the problem to be solved and the desired product **31** to be obtained in well reproducible 70 % yield with impeccable integrity of the double‐bond geometry (*E*:*Z* ≥95:5, ^1^H NMR). Although the generality of this finding needs yet to be explored in detail, it suggests that these modified Zakarian conditions might further increase the scope of iododesilylation and render the reaction applicable to otherwise “protecting group free” synthetic ventures.

**Figure 2:**
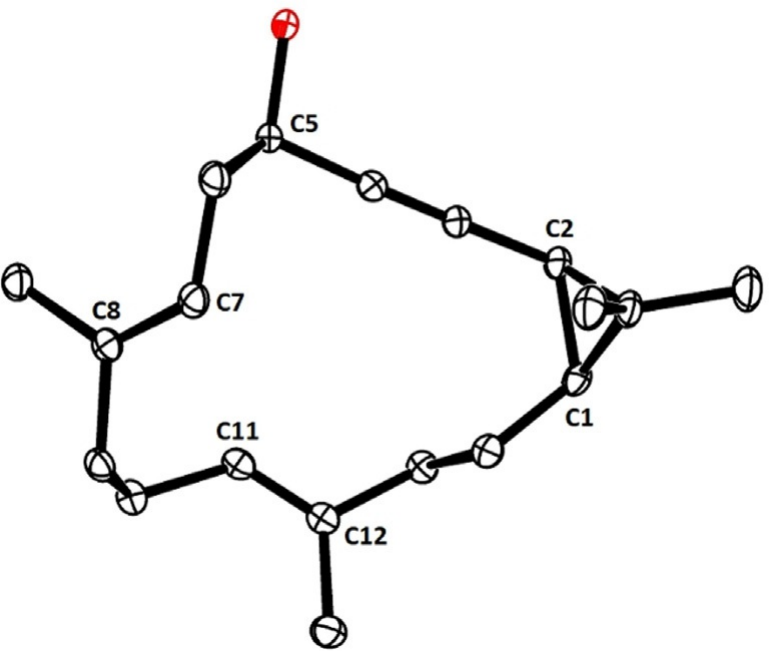
Structure of **34** in the solid state (casbane numbering scheme).

**Scheme 4:**
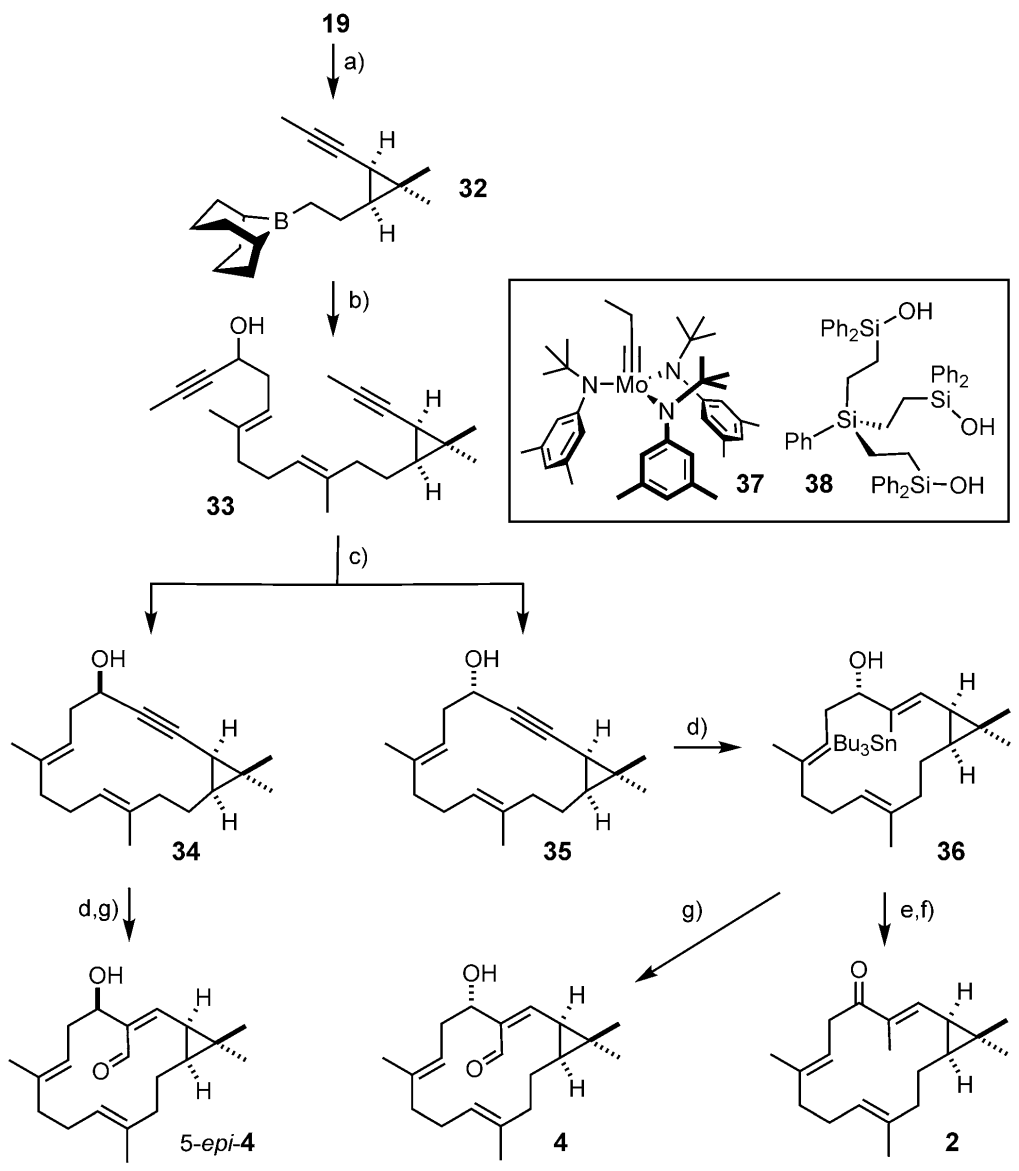
a) 9‐H‐9‐BBN, THF, 0 °C→RT; b) **31**, [(dppf)PdCl_2_] (10 mol %), Ba(OH)_2_⋅8 H_2_O, 69 %; c) **37** (20 mol %), **38** (22 mol %), MS 5 Å, toluene, reflux, 60 % (**34**+**35**); d) [Cp*RuCl]_4_ (2.5 mol %), Bu_3_SnH, CH_2_Cl_2_, 88 % (**36**); e) MeI, Pd(PPh_3_)_4_ (5 mol %), [Bu_4_N][Ph_2_PO_2_], CuTC, DMF, 67 %; f) MnO_2_, CH_2_Cl_2_, 73 %; g) MeLi, THF, then DMF, −78 °C→RT, 68 % (**4**), 53 % (5‐*epi*‐**4**); 9‐BBN=9‐borobicyclo[3.3.1]nonane, Cp*=pentamethylcyclopentadienyl, CuTC=copper thiophene‐2‐carboxylate.

The coupling of the building blocks commenced with an exquisitely chemoselective hydroboration of the terminal alkene in **19** in the presence of the internal alkyne, which was achieved on treatment with 9‐H‐9‐BBN dimer in THF; the choice of solvent is critical (Scheme 4).[^50^, ^51^] The resulting alkylborane **32** was then subjected to a Suzuki coupling^[52]^ with alkenyl iodide **31** using Ba(OH)_2_⋅8 H_2_O as promotor;[^53^, ^54^] other bases gave inferior results. Since the latest generations of alkyne metathesis catalysts endowed with (chelating) silanolate ligands work even in the presence of unprotected alcohols despite the inherent nucleophilicity/basicity of the operative Schrock‐type alkylidyne unit,[^55^, ^56^] diyne **33** thus formed could be directly subjected to RCAM to give the cycloalkynes **34** and **35**. The fact that the reaction had to be carried out in refluxing toluene is tentatively ascribed to the high ring strain of these compounds; this aspect and the rather forcing conditions notwithstanding, the cyclopropyl unit survived. At this stage, the two diastereomeric alcohols were easily separated by flash chromatography and the structure of **34** in the solid state was confirmed (Figure 2). It shows that the *cis* configuration of the three‐membered ring is intact: neither cleavage nor scrambling had interfered during the metathesis of the adjacent triple bond.

With **34** and **35** in hand and the configuration of the C5−OH group rigorously established, the project reached the phase of late‐stage diversification. To this end, **35** was subjected to ruthenium catalyzed *trans*‐hydrostannation to give product **36** as the only detectable isomer in good yield.[^29^, ^57^] A formal Stille cross‐coupling with MeI under conditions previously developed in our laboratory allowed the yet missing methyl substituent to be introduced,[^58^, ^59^] before the resulting product was oxidized to the corresponding ketone **2**. The NMR spectra as well as the specific rotation of synthetic **2** are in excellent agreement with those of depressin reported in the literature.^[3]^ We hence confirm the constitution and absolute configuration assigned to this natural product derived from the soft coral *Sinularia depressa* collected off the shore of Hainan Province, China.^[60]^

Next, stannane **36** was transformed into the corresponding aldehyde **4** upon treatment with MeLi (2 equiv) followed by quenching of the resulting organolithium species with DMF.^[61]^ Compound **4** could represent euphorhylonal A, a natural product extracted from *Euphorbia hylonoma*; the spectra of **4**, however, were not matching. Since the configuration of euphorhylonal A at C5 had not been established by the isolation team and is therefore an obvious point of concern,^[8]^ the epimeric cycloalkyne **34** was subjected to an analogous sequence of *trans*‐hydrostannation/formylation. Yet, the recorded spectra of the resulting aldehyde 5‐*epi*‐**4** were not in accord with the literature either. It was hence clear that euphorhylonal A does not contain a *cis*‐configured cyclopropane as proposed in the literature;^[8]^ unfortunately, the published data do not provide any hint as to which of the two positions on the three‐membered ring might have been misassigned and needs to be inverted.^[8]^

**Scheme 5:**
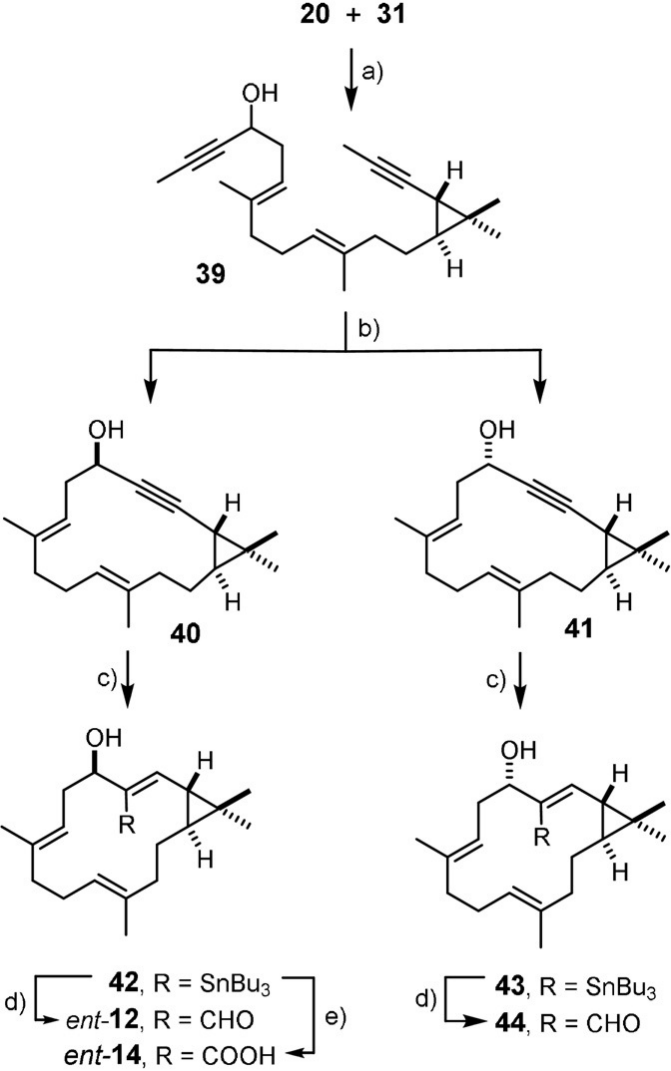
a) **20**, 9‐H‐9‐BBN, THF, 0 °C→RT, then **31**, [(dppf)PdCl_2_] (5 mol %), Ba(OH)_2_⋅8 H_2_O, 82 %; b) **37** (20 mol %), **38** (22 mol %), MS 5 Å, toluene, reflux, 76 % (**40**+**41**); c) [Cp*RuCl]_4_ (2.5 mol %), Bu_3_SnH, CH_2_Cl_2_, 65 % (**42**) + 12 % (isomer, see the Supporting Information),^[62]^ 74 % (**43**); d) MeLi, THF, then DMF, −78 °C→RT, 61 % (*ent*‐**12**), 69 % (**44**); e) MeLi, THF, then CO_2_, −78 °C→RT, 51 %.

Since we had planned a foray into the *trans* series anyway in order to showcase the flexibility of the synthesis blueprint, pekinenin C (**12**)^[6]^ isolated from the roots of *Euphorbia pekinensis* was the obvious target because it differs from nominal eyphorhylonal A (**4**) solely in the configuration of one center on the cyclopropane ring. This compound exhibits appreciable cytotoxicity rooted in its ability to induce apoptotic cell death.^[14]^ The synthesis follows the established route in that the appropriate enyne **20** (see Scheme 2) was subjected to chemoselective hydroboration followed by cross‐coupling of the resulting alkylborane with **31** to give diyne **39** in readiness for RCAM (Scheme 5). Using the same catalyst generated in situ from complex **37** and ligand **38**,^[55]^ the macrocyclization proceeded within minutes at 70 °C; the comparison with the cyclization of diyne **33**, which had required reflux temperature, suggests that the *trans*‐configured cyclopropane imposes less strain on the transition state and the resulting bicyclic scaffold of **40** and **41**. That the three‐membered ring remained intact during the alkyne metathesis reaction echoes the results discussed above. Once again, the diastereomeric alcohols could be readily separated at this stage; the configuration of the C5−OH was determined by Mosher ester analysis (see the SI). Each isomer was then subjected to *trans*‐hydrostannation and subsequent formylation to give isomers *ent*‐**12** and **44**, respectively.^[62]^ Gratifyingly, the spectral data as well as the specific rotation of synthetic *ent*‐**12** correspond well to those of euphorhylonal A;^[8]^ the NMR spectrum is also identical with that of pekinenin C, whereas the sign of the [α]_D_ is opposite.^[6]^ We hence conclude that euphorhylonal A and pekinenin C are not diastereomeric but almost certainly enantiomeric to each other.^[63]^ This finding shows that phylogenetically closely related *Euphorbia* plants can produce *antipodal* secondary metabolites.

Finally, **42** was carboxylated by tin/lithium exchange followed by a CO_2_ quench; the resulting acid *ent*‐**14** represents naturally occurring (+)‐yuexiandajisu A and clarifies the previously unknown absolute configuration of this secondary metabolite with antibacterial properties derived from the medical plant *Euphorbia ebracteolata*; the roots of this plant are one of the ingredients of the traditional Chinese medicine recipe “*Lang du*”.^[9]^

## Conclusion

In summary, we have developed a short, efficient, selective, modular, and inherently flexible approach to the casbane family of diterpenes, as illustrated by the total synthesis of depressin, yuexiandajisu A, and *ent*‐pekinenin C; the latter proved identical with natural euphorhylonal A which had obviously been misassigned by the isolation team. The chosen route also clarifies a previously unknown aspect of alkyne metathesis in that it proves that a cyclopropane ring conjugated to the triple bond to be activated and catalytically transformed remains intact even under forcing conditions. Finally, the present study further illustrates the virtues of directed *trans*‐hydrometalation, a reaction manifold that had been largely unknown until recently but proves versatile and enabling, not least as an entry point into a variety of stereodefined trisubstituted alkenes.[^28^, ^59^, ^60a^, ^64^] Further studies along these lines, including approaches to even more complex target compounds, are underway and will be reported in due course.

## Conflict of interest

The authors declare no conflict of interest.

## Supporting information

As a service to our authors and readers, this journal provides supporting information supplied by the authors. Such materials are peer reviewed and may be re‐organized for online delivery, but are not copy‐edited or typeset. Technical support issues arising from supporting information (other than missing files) should be addressed to the authors.

Supplementary
