# Supplementary material for: Collective Total Synthesis of Casbane Diterpenes: One Strategy, Multiple Targets
Source: Angew Chem Int Ed Engl. 2021 Jan 28;60(10):5316–22. doi: 10.1002/anie.202015243 (PMC7986786; doi:10.1002/anie.202015243)
Supplement: Supplementary file 1 — Supplementary [file ANIE-60-5316-s001.pdf]

Angewandte Chemie
Eine Zeitschrift der Gesellschaft Deutscher Chemiker

Supporting Information

Collective Total Synthesis of Casbane Diterpenes: One Strategy, Multiple Targets

*Lorenz E. Löffler, Conny Wirtz, and Alois Fürstner**

anie_202015243_sm_miscellaneous_information.pdf

SUPPORTING CRYSTALLOGRAPHIC INFORMATION

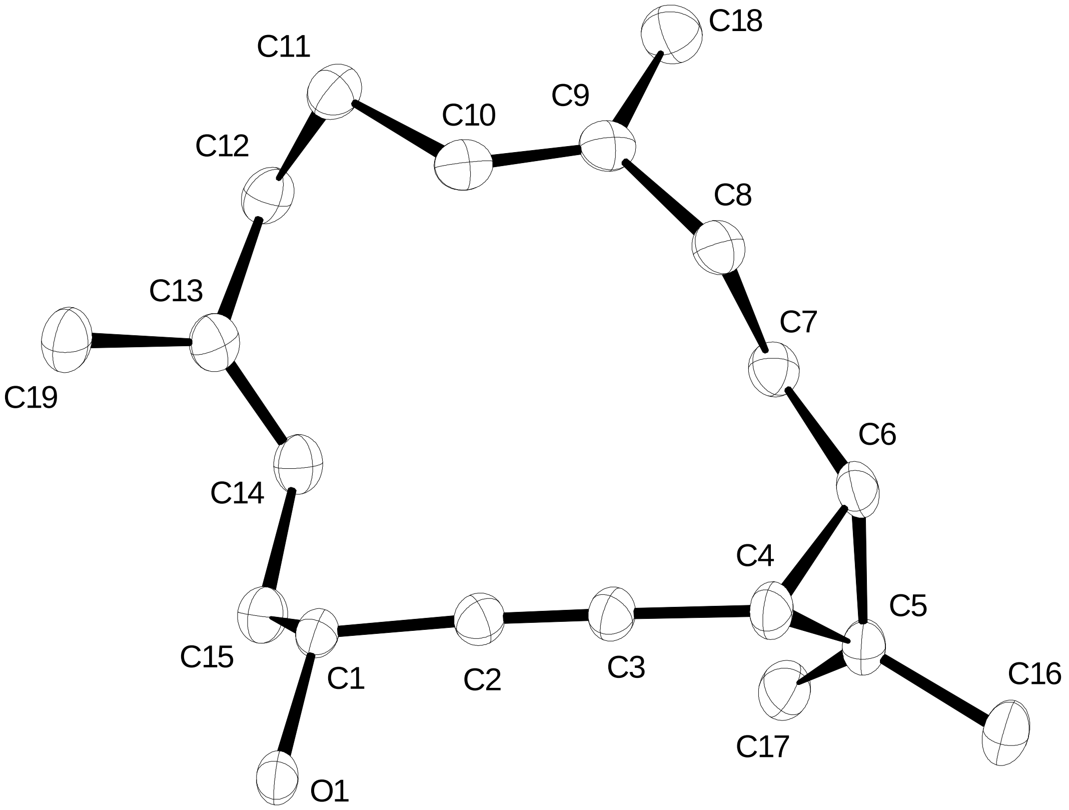

Figure S1. Structure of cycloalkyne **34** in the solid state; arbitrary numbering system

X-ray Crystal Structure Analysis of Compound 34: $C_{19}H_{28}O$, M_r = 272.41 g · mol^{-1}, colourless plate, crystal size 0.141 x 0.062 x 0.043 mm^3, tetragonal, space group $P4_3$[78], a = 13.9323(3) Å, b = 13.9323(3) Å, c = 8.8998(3) Å, V = 1727.53(9) Å^3, T = 100(2) K, Z = 4, D_{calc} = 1.047 g · cm^3, λ = 1.54178 Å, $\mu(Cu\text{-}K_\alpha)$ = 0.470 mm^{-1}, analytical absorption correction (T_{min} = 0.95, T_{max} = 0.98), Bruker AXS Enraf-Nonius KappaCCD diffractometer with a FR591 rotating Mo-anode X-ray source, 3.172 < θ < 71.125°, 46603 measured reflections, 3122 independent reflections, 2837 reflections with $I > 2\sigma(I)$, R_{int} = 0.0626. S = 1.051, 190 parameters, absolute structure parameter = 0.0(4), residual electron density +0.2 (1.77 Å from H1) / -0.2 (1.02 Å from C6) e · Å^{-3}. The hydrogen at O1 was found and refined, all other hydrogens were placed in calculated positions.

The structure was solved by *SHELXT* and refined by full-matrix least-squares (*SHELXL*) against F^2 to R_1 = 0.033 [$I > 2\sigma(I)$], wR_2 = 0.084. **CCDC-2041047**.

General. Unless stated otherwise, all reactions were carried out in flame-dried glassware using anhydrous solvents under argon. The following solvents and reagents were purified by distillation over the drying agents as indicated and were transferred under argon: THF, Et_2O (Mg/anthracene), toluene (Na/K alloy), MeOH (Mg, stored over MS 3 Å); 2,6-lutidine, MeCN, DMF, Et_3N, CH_2Cl_2, DMPU (CaH_2).

All commercially available compounds (Alfa Aesar, Aldrich, TCI Chemicals, Strem Chemicals, ChemPUR, Fluorochem) were used as received, unless stated otherwise. The following compounds were prepared according to the cited literature: Active MnO_2 was synthesized by a literature procedure,[1] $[Cp^*RuCl]_4$,[2] Mo complex **37**,[3] and ligand **38**.[4]

Hexafluoroisopropanol (HFIP) was stored over molecular sieves at RT for 2 d prior to use. CuCN was dried for 14 h at 120°C (oil bath) under vacuum prior to use, and stored and transferred under argon atmosphere. *N*-Iodosuccinimde was recrystallized form pentane and stored under Argon in the dark. Diiodoethane was purified by washing the dissolved reagent in Et_2O with saturated aqueous $Na_2S_2O_3$ solution; the ether phase was dried over $MgSO_4$ and concentrated, and the resulting product stored under argon atmosphere. The molecular sieves were dried at 140°C (oil bath) under vacuum overnight prior to use; they were stored and transferred under argon atmosphere.

Thin layer chromatography (TLC): Macherey-Nagel precoated plates (POLYGRAM®SIL/UV254). Detection was achieved under UV light (254 nm) and by staining with either acidic *p*-anisaldehyde, cerium ammonium molybdenate, or basic KMnO4 solution. Flash chromatography: Merck silica gel 40-63 µm with predistilled or HPLC grade solvents. Preparqative HPLC separations were carried out on an Agilent 1260 Infinity II Preparative LC System.

IR: Spectra were recorded on an Alpha Platinum ATR instrument (Bruker) at ambient temperature, wavenumbers ($\tilde{\nu}$) in cm^{-1}. MS: ESI-MS: ESQ3000 (Bruker), accurate mass determinations: Bruker APEX III FT-MS (7 T magnet) or Mat 95 (Finnigan). Optical rotations ($[\alpha]_D$) were measured with an A-Krüss Otronic Model P8000-t polarimeter at a wavelength of 589 nm. NMR: Spectra were recorded on a Bruker AVIII 400 or AVIII 600 or AV600neo (the latter two both equipped with cryoprobes) spectrometer in the solvents indicated; chemical shifts (δ) are given in ppm relative to TMS, coupling constants (*J*) in Hz. The solvent signals were used as references and the chemical shifts converted to the TMS scale ($CDCl_3$: $\delta_C \equiv 77.0$ ppm;

---

1 J. Attenburrow, A. F. B. Cameron, J. H. Chapman, R. M. Evans, B. A. Hems, A. B. A. Jansen, T. Walker, *J. Chem. Soc.* **1952**, 1094.

2 a) P. J. Fagan, W. S. Mahoney, J. C. Calabrese, I. D. Williams, *Organometallics* **1990**, *9*, 1843-1852; b) P. J. Fagan, M. D. Ward, J. C. Calabrese, *J. Am. Chem. Soc*. **1989**, *111*, 1698-1719.

3 W. Zhang, Y. Lu, J. S. Moore, *Org. Synth*. **2007**, *84*, 163-176.

4 S. Schaubach, K. Gebauer, F. Ungeheuer, L. Hoffmeister, M. K. Ilg, C. Wirtz, A. Fürstner, *Chem. Eur. J*. **2016**, *22*, 8494-9507.

residual $CHCl_3$ in $CDCl_3$: $\delta_H \equiv 7.26$ ppm; CD_2Cl_2: $\delta_C \equiv 53.8$ ppm; residual $CDHCl_2$: $\delta_H \equiv 5.32$ ppm; all spectra were recorded at 25 °C. Multiplets are indicated by the following abbreviations: s: singlet, d: doublet, t: triplet, q: quartet, p: pentet, h: hextet, hept: heptet, m: multiplet, br: broad. ^{13}C spectra were recorded in {^1H}-decoupled manner and the values of the chemical shifts are rounded to one decimal point. Signal assignments were established using HSQC, HMBC, COSY, NOESY and other 2D experiments; numbering schemes as shown in the inserts. GC analyses were conducted on an Agilent technologies 7890B instrument with a FID detector.

Preparation of the Cyclopropyl Building Blocks

3-Methylbut-2-en-1-yl 3-oxobutanoate (S1). A solution of freshly distilled diketene (15.70 g, 84.07 mmol) in THF (19 mL) was added to 3-methyl-2-buten-1-ol (16.94 mL, 14.36 g, 166.73 mmol) and sodium acetate (766 mg, 9.34 mmol) in refluxing THF (47 mL) over the course of 1 h. Stirring was continued for 30 min at reflux temperature before the mixture was cooled to RT and concentrated. The residue was purified by distillation to yield the title compound as a colorless liquid (19.76 g, 70%). B.p. 85-88 °C (10 mbar); ^1H NMR (400 MHz, $CDCl_3$): δ = 12.08 (s, enol form), 5.34 (ddt, J = 7.3, 4.2, 1.4 Hz, 1H), 4.98 (m, enol form), 4.64 (d, J = 7.3 Hz, 2H), 3.44 (s, 2H), 2.26 (s, 3H), 1.94 (s, enol form), 1.76 (s, 3H), 1.71 ppm (s, 3H); ^{13}C NMR (101 MHz, $CDCl_3$): δ = 200.6, 167.1, 139.9, 117.9, 62.2, 50.1, 30.1, 25.7, 18.0 ppm (minor signals of the enol tautomer are visible); IR (film) $\tilde{\nu}$ = 2973, 2935, 1736, 1714, 1646, 1411, 1360, 1311, 1232, 1147, 953, 542 cm^{-1}; HRMS (ESI): m/z calcd. for $C_9H_{14}O_3$ [M^++Na]: 193.08351; found: 193.08372.

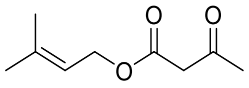

3-Methylbut-2-en-1-yl 2-diazoacetate (15). A solution of p-acetamidobenzenesulfonyl azide (18.44 g, 76.76 mmol) in MeCN (50 mL) was added to a solution of 3-methyl-2-buten-1-yl acetoacetate (10.05 g, 59.05 mmol) and Et_3N (10.70 mL, 7.77 g, 76.76 mmol) in MeCN (50 mL) over 30 min. A white precipitate of p-acetamidobenzenesulfonamide was observed after ≈30 min; at this point, additional MeCN (30 mL) was added and stirring continued for additional 4 h. A solution of LiOH (4.67 g, 194.85 mmol) in water (20 mL) was added and the mixture was stirred at RT for 12 h. The aqueous layer was separated and extracted with Et_2O/EtOAc (2:1, 3 × 70 mL). The combined organic phases were dried over $MgSO_4$ and concentrated. The residue was purified by flash chromatography (hexane/EtOAc, 15:1) to yield the title compound as a yellow oil (9.10 g, 75%). ^1H NMR (400 MHz, $CDCl_3$): δ = 5.34 (ddq, J = 8.6, 5.7, 1.4 Hz, 1H), 4.74 (s, 1H), 4.66 (d, J = 7.2 Hz, 2H), 1.76 (s, 3H), 1.72 ppm (s, 3H); ^{13}C NMR (101 MHz, $CDCl_3$): δ = 166.9, 139.3, 118.5, 61.7, 46.2, 25.8, 18.0 ppm; IR (film) $\tilde{\nu}$ = 3113, 2973, 2935, 2103, 1684, 1444, 1386, 1356, 1342, 1234, 1172, 995, 462, 433 cm^{-1}; HRMS (ESI):

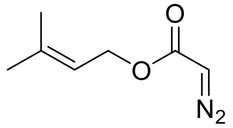

m/z calcd. for $C_7H_{10}N_2O_2$ [M^++Na]: 177.06345; found: 177.06346.

(1*S*,5*R*)-6,6-Dimethyl-3-oxabicyclo[3.1.0]hexan-2-one (16). A solution of diazo ester **15** (5.15 g, 33.41 mmol) in CH_2Cl_2 (17 mL) was added to a clear violet solution of [Rh_2(5*S*-MEPY)$_4$]·(MeCN)$_2$ (168.3 mg, 196.5 µmol, 0.6 mol%) in CH_2Cl_2 (110 mL) at reflux temperature via syringe pump over the course of 18 h. Once the addition was complete, stirring was continued for an additional 30 min before the mixture was cooled to RT and concentrated. The residue was purified by flash chromatography (hexane/EtOAc, 10:1 → 3:1) to give the title compound as a colourless oil (3.68 g, 87%, 93% *ee*). $[\alpha]_D^{20}$ = +86.9 (1.09 g/100 mL, $CHCl_3$); ^1H NMR (400 MHz, $CDCl_3$): δ = 4.36 (dd, *J* = 9.9, 5.5 Hz, 1H), 4.15 (dt, *J* = 9.9, 1.1 Hz, 1H), 2.04 (ddd, *J* = 6.5, 5.5, 1.1 Hz, 1H), 1.95 (dd, *J* = 6.3, 1.0 Hz, 1H), 1.18 (s, 3H), 1.17 ppm (s, 3H); ^{13}C NMR (101 MHz, $CDCl_3$) δ = 175.0, 66.5, 30.5, 30.0, 25.2, 23.0, 14.4 ppm; IR (film) $\tilde{\nu}$ = 2961, 2909, 2878, 1766, 1458, 1382, 1361, 1283, 1217, 1178, 1118, 1092, 1049, 1023, 974, 958, 892, 857 cm^{-1}; HRMS (ESI): *m/z* calcd. for $C_7H_{10}O_2$ [M^++Na]: 149.05730; found: 149.05725.

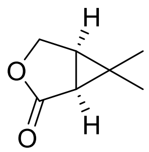

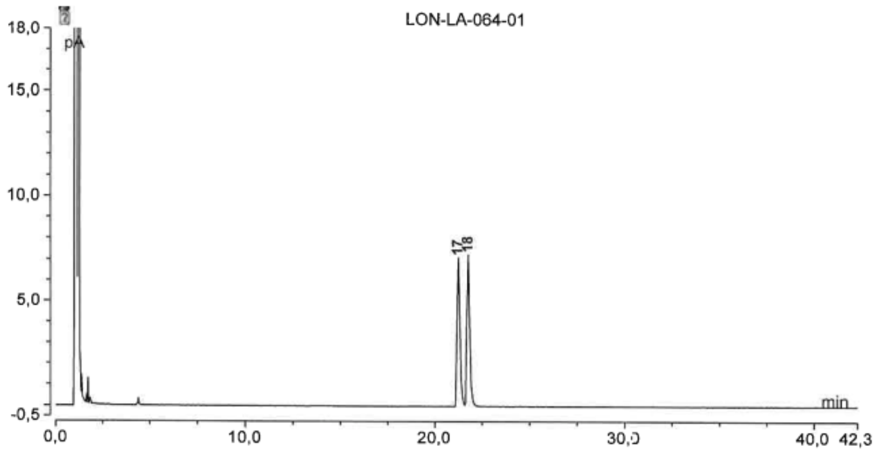

Sample:	**LON-LA-064-01**	Instrument:	**GC_122**
Sequenz:	**3766 LON-LA FK**	Measured:	**15.11.17 12:41**
Sequenz date:	**15.11.17**	Processing M.:	MPI
		Report-File:	Verhältnis

chirale Messung, ee-Verhältnis, Racemat-Probe
Zuordnung laut Herrn Löffler, siehe achirale Messung

No.	Ret.Time min	Rel.Area %	Peak Name
17	21,25	49,84	
18	21,77	50,16	

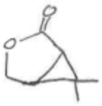

α = 1,025

Instrument parameters:

Column:	25,0 m	Lipodex-A ; G/732
Temperature:	220/65iso/350	
Gas:	0,60 bar	H2
Sample size:	1,0 µL	

F. Kohler

Page 1-1

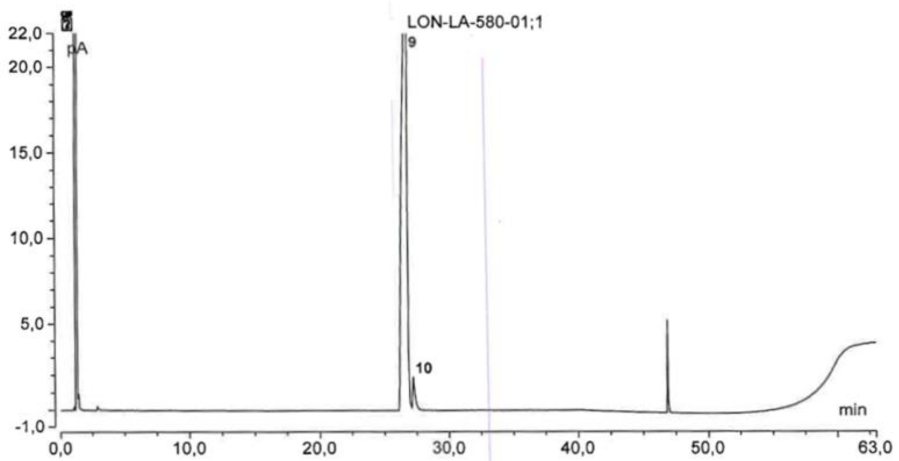

Sample:	LON-LA-580-01;1	Instrument:	GC_212
Sequenz:	6908 LON-LA PH	Measured:	22.11.19 07:44
Sequenz date:	21.11.19	Processing M.:	ee 580
		Report-File:	Verhältnis

Verhältnis Enantiomere
Zuordnung nach Vergleichsmessung LON-LA-064-01 17/3766

No.	Ret.Time min	Rel.Area %	Peak Name
9	26,78	96,68	.
10	27,22	3,32	.

Instrument parameters:

Column:	25,0 m	Lipodex-A 0,25/?df G/732
Temperature:	220 / 60, 40 min iso 8/min 220, 3 min iso / 350	
Gas:	0,60 bar	H2
Sample size:	0,2 µL	

((1*R*,3*S*)-2,2-Dimethyl-3-vinylcyclopropyl)methanol (17). DIBAL-H (1.0 M in CH_2Cl_2, 9.06 mL, 9.06 mmol) was added dropwise to a solution of lactone **16** (1.12 g, 8.89 mmol) in CH_2Cl_2 at −78 °C and the resulting mixture was stirred for 30 min at this temperature. The reaction was quenched at −78 °C with MeOH, followed by addition of saturated aqueous Rochelle Salt solution. The resulting mixture was rapidly stirred at RT for 1 h before the aqueous layer was separated and extracted with CH_2Cl_2 (3 × 30 mL). The combined organic phases were dried over $MgSO_4$ and concentrated, and the crude lactol was used without further purification.

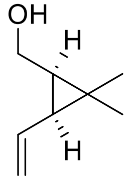

n-BuLi (1.6 M in hexane, 16.66 mL, 26.66 mmol) was added to a suspension of methyltriphenylphosphonium bromide (9.52 g, 26.66 mmol) in THF (84 mL) at 0 °C and the resulting suspension was stirred at RT for 1 h. A solution of the lactol in THF (2 mL) was added to the ylide suspension at 0 °C and the resulting mixture was stirred at RT for 3 h. The reaction was quenched with saturated aqueous NH_4Cl solution, and the aqueous layer was separated and was extracted with Et_2O (3 × 20 mL). The combined organic phases were dried over $MgSO_4$ and concentrated. The residue was purified by flash chromatography (pentane/Et_2O, 10:1) to yield the title compound as a colourless oil (614 mg, 55%). $[\alpha]_D^{20}$ = +44.2 (1.29 g/100 mL, $CHCl_3$); ^1H NMR (400 MHz, $CDCl_3$): δ = 5.62 (dt, J = 16.9, 10.1 Hz, 1H), 5.21

(ddd, J = 17.0, 2.1, 0.7 Hz, 1H), 5.06 (ddd, J = 10.3, 2.1, 0.6 Hz, 1H), 3.72 (m, 2H), 1.44 (t, J = 9.3 Hz, 1H), 1.17 (m, 1H), 1.13 (s, 3H), 1.11 ppm (s, 3H); ^{13}C NMR (101 MHz, $CDCl_3$): δ = 134.3, 116.1, 60.3, 32.3, 31.3, 28.7, 22.0, 15.4 ppm; IR (film) $\tilde{\nu}$ = 3330, 3081, 2986, 2946, 2925, 2866, 1632, 1454, 1377, 1259, 1165, 1017, 988, 896, 801, 725, 661 cm^{-1}; HRMS (ESI): m/z calcd. for $C_8H_{14}O$ [M^++H]: 127.11174; found: 127.11160.

(2*R*,3*S*)-1,1-Dimethyl-2-(prop-1-yn-1-yl)-3-vinylcyclopropane (19). Dess-Martin-periodinane (2.6 g, 6.2 mmol) was added to a solution of alcohol **17** (523.0 mg, 4.1 mmol) in CH_2Cl_2 (40 mL) at 0 °C. The resulting mixture was stirred at 0 °C for 10 min, followed by stirring at RT for 4 h. The reaction was quenched with saturated aqueous $NaHCO_3/Na_2S_2O_3$ solution (1:1 *v/v*, 50 mL). The mixture was rapidly stirred for 30 min, the aqueous layer was separated and extracted with CH_2Cl_2 (3 × 30 mL), the organic phases were dried over $MgSO_4$, filtered and concentrated, and the resulting aldehyde **18** was used without further purification.

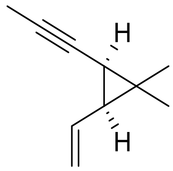

The crude aldehyde **18** was added at 0 °C to a mixture of PPh_3 (8.70 g, 33.15 mmol) and CBr_4 (5.50 g, 16.58 mmol) in CH_2Cl_2 (40 mL), which had previously been stirred at RT for 10 min. The resulting mixture was vigorously stirred for 10 min before it was diluted with pentane (10 mL). The suspension was filtered through a plug of Celite, which was carefully rinsed with pentane (20 mL). The combined filtrates were washed with water and brine, dried over $MgSO_4$ and concentrated. The resulting dibromide was used without further purification.

n-BuLi (1.6 M in hexane, 12.95 mL, 20.72 mmol) was added to a solution of the dibromide in Et_2O (65 mL) at −78 °C and the mixture was stirred for 1 h at this temperature. DMPU (3.01 mL, 3.19 g, 24.87 mmol) was added at −78 °C, followed, after 10 min, by MeI (3.87 mL, 8.82 g, 62.17 mmol) . The resulting mixture was warmed to RT overnight. The reaction was quenched with saturated aqueous NH_4Cl solution, and the aqueous layer was separated and extracted with pentane (2 × 10 mL) and Et_2O (1 × 10 mL). The combined organic phases were washed with saturated aqueous NaCl solution, dried over $MgSO_4$ and concentrated. The residue was purified by flash chromatography (pentane) to yield the title compound as a colourless oil (262.0 mg, 51%). $[\alpha]_D^{20}$ = +82.6 (0.99 g/100 mL, $CHCl_3$); ^{1}H NMR (600 MHz, CD_2Cl_2): δ = 5.65 (ddd, J = 17.2, 10.4, 9.4 Hz, 1H), 5.18 (ddd, J = 17.1, 2.2, 0.6 Hz, 1H), 5.08 (ddd, J = 10.4, 2.1, 0.6 Hz, 1H), 1.81 (d, J = 2.2 Hz, 3H), 1.44 (dd, J = 9.3, 8.4 Hz, 1H), 1.40 (dq, J = 8.3, 2.2 Hz, 1H), 1.11 (s, 3H), 1.08 ppm (s, 3H); ^{13}C NMR (151 MHz, CD_2Cl_2): δ = 136.0, 115.8, 76.9, 76.5, 33.6, 27.3, 24.1, 21.2, 16.9, 3.7 ppm; HRMS (ESI): m/z calcd. for $C_{10}H_{14}$ [M^+]: 134.10900; found: 134.10911.

(2*S*,3*S*)-1,1-Dimethyl-2-(prop-1-yn-1-yl)-3-vinylcyclopropane (20). Dess-Martin-periodinane (10.15 g, 23.93 mmol) was added to a solution of alcohol **17** (1.51 g, 12.96 mmol) in CH_2Cl_2 (115 mL) at 0 °C. The resulting mixture was stirred at 0 °C for 10 min and for antoher 4 h at RT. The reaction was quenched with saturated aqueous $NaHCO_3/Na_2S_2O_3$ solution (200 mL, vol 1:1). The mixture was rapidly stirred for 30 min before the aqueous layer was separated and extracted with CH_2Cl_2 (3 × 40 mL). The combined organic phases were dried over $MgSO_4$, filtered and concentrated, and the resulting crude aldehyde **18** was used without further purification.

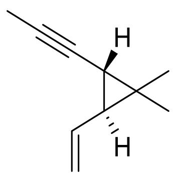

K_2CO_3 (8.3 g, 59.83 mmol) was added to a solution of the crude aldehyde in MeOH (50 mL). The resulting suspension was stirred at 50 °C for 3 h. The reaction was quenched at RT with saturated aqueous NH_4Cl solution. The aqueous layer was separated and extracted with CH_2Cl_2 (3 × 40 mL), and the combined organic phases were dried over $MgSO_4$ and concentrated. The resulting aldehyde 2-*epi*-**18** was used without further purification.

This crude aldehyde was added to a mixture of PPh_3 (25.11 g, 95.72 mmol) and CBr_4 (15.87 g, 47.86 mmol) in CH_2Cl_2 (115 mL) at 0 °C, which had previously been stirred at RT for 10 min. After 10 min, the mixture was diluted with pentane and the suspension filtered through a plug of Celite, which was carefully rinsed with pentane. The combined filtrates were washed with water and brine, dried over $MgSO_4$ and concentrated. The resulting dibromide was used without further purification.

n-BuLi (1.6 M in hexane, 37.4 mL, 59.83 mmol) was added to a solution of the crude dibromide in Et_2O (100 mL) at −78 °C and the mixture was stirred for 1 h. DMPU (8.7 mL, 71.79 mmol) was added at −78°C, followed, after 10 min, by MeI (11.17 mL, 179.48 mmol). The resulting mixture was warmed to RT overnight before the reaction was quenched with saturated aqueous NH_4Cl solution. The aqueous layer was separated and extracted with pentane (3 × 10 mL), and the combined organic phases were washed with saturated aqueous NaCl solution, dried over $MgSO_4$ and concentrated. The residue was purified by flash chromatography (pentane) to yield the title compound as a colourless oil (1.02 g, 63%, *cis*:*trans* = 1:9). $[\alpha]_D^{20}$ = −66.9 (2.08 g/100 mL, $CHCl_3$); ^1H NMR (600 MHz, CD_2Cl_2): δ = 5.50 (dddd, J = 17.0, 10.3, 8.9, 0.4 Hz, 1H), 5.12 (ddd, J = 17.0, 1.9, 0.8 Hz, 1H), 5.01 (ddd, J = 10.3, 1.9, 0.6 Hz, 1H), 1.78 (d, J = 2.1 Hz, 3H), 1.37 (dd, J = 8.8, 5.1 Hz, 1H), 1.19 (s, 3H), 1.14 (dd, J = 5.0, 2.3 Hz, 1H), 1.05 ppm (s, 3H); ^{13}C NMR (151 MHz, CD_2Cl_2): δ = 137.0, 115.3, 78.7, 74.8, 38.2, 25.2, 23.0, 22.2, 20.8, 3.6 ppm; HRMS (ESI): m/z calcd. for $C_{10}H_{14}$ [M^++H]: 135.11683; found: 135.11686.

Preparation of the Alkenyl Iodide Building Block

(*E*)-4-(Dimethyl(phenyl)silyl)pent-3-en-1-ol (24). $PhMe_2SiCl$ (7.39 mL, 7.51 g, 44.00 mmol) was added to a suspension of lithium sand (916 mg, 132.0 mmol) in THF (120 mL) at −10 °C. The resulting mixture was stirred at −10 °C for 36 h. [The titer of the $PhMe_2SiLi$ solution was determined by addition of an aliquot of the resulting mixture (2 mL) to water (5 mL) followed by titration with HCl (1 M in water)].

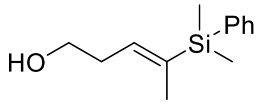

The resulting $PhMe_2SiLi$ solution (102.00 mL, 37.74 mmol, 0.37 M in THF) was added dropwise to a suspension of CuCN (1.69 g, 18.87 mmol, dried at 120 °C for 14 h under high vacuum prior to use) in THF (5 mL) at −78 °C. The resulting mixture was stirred at −30 °C for 30 min before it was cooled to −78 °C. *n*BuLi (1.59 M in hexane, 98.7 mL, 16.98 mmol) was added dropwise to a solution of 3-pentyn-1-ol (1.43 g, 16.98 mmol) in Et_2O at −78 °C. The mixture was stirred at −30 °C for 20 min before it was cooled to −78 °C. The resulting mixture was added dropwise to the solution of the higher order silyl cuprate at −78 °C. The mixture was stirred at −78 °C for 1 h before the reaction was quenched with saturated aqueous NH_4Cl/NH_3 solution. The aqueous layer was separated and extracted with ethyl acetate (3 × 200 mL). The combined organic phases were washed with saturated aqueous NaCl solution, dried over $MgSO_4$ and concentrated. The residue was purified by flash chromatography (hexane/EtOAc, 10:1) to yield the title compound as a colourless oil (3.38 g, 90%). ^1H NMR (400 MHz, $CDCl_3$): δ = 7.49 (m, 2H), 7.35 (dd, *J* = 5.0, 1.8 Hz, 3H), 5.82 (ddt, *J* = 6.9, 5.2, 1.8 Hz, 1H), 3.69 (t, *J* = 6.6 Hz, 2H), 2.43 (dddd, *J* = 7.6, 6.7, 5.8, 0.9 Hz, 2H), 1.71 (dd, *J* = 1.7, 0.9 Hz, 3H), 1.46 (s, 1H), 0.34 ppm (s, 6H); ^{13}C NMR (101 MHz, $CDCl_3$): δ = 138.3, 138.1, 136.5, 133.9, 128.9, 127.7, 62.1, 32.1, 15.0, −3.5 ppm; IR (film) $\tilde{\nu}$ = 3337, 3068, 2956, 1618, 1427, 1248, 1110, 1045, 831, 814, 773, 731, 700, 638, 473 cm^{-1}; HRMS (ESI): *m/z* calcd. for $C_{13}H_{21}OSi$ [M^+]: 221.13562; found: 221.13540.

(4,5-Dihydrofuran-2-yl)dimethyl(phenyl)silane (23). *n*BuLi (1.6 M in hexane, 73.0 mL, 116.8 mmol) was added to a solution of 2,3-dihydrofuran (9.5 mL, 8.8 g, 125.6 mmol) in THF (45 mL) at −30 °C. The resulting mixture was stirred for 30 min at this temperature and for another 30 min at RT. The solution was cooled to –30°C before $PhMe_2SiCl$ (15.0 mL, 15.3 g, 89.4 mmol) was introduced and stirring was continued for 30 min. The mixture was slowly warmed over 1 h and stirred at RT for 12 h. The reaction was quenched with aqueous saturated NH_4Cl solution. The aqueous layer was separated and extracted with pentane (3 × 200 mL). The combined organic phases were washed with aqueous saturated NaCl solution, dried over $MgSO_4$ and concentrated. The residue was filtered through a plug of basic alumina, rinsing with pentane, and the combined filtrates were concentrated. The residue was purified by flash chromatography (hexane/EtOAc, 50:1) to yield the title compound as a colourless oil (18.7 g, quant.). ^1H NMR (400 MHz, $CDCl_3$): δ = 7.58 (m, 2H), 7.36 (m, 3H),

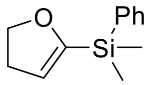

5.25 (t, J = 2.6 Hz, 1H), 4.31 (t, J = 9.6 Hz, 2H), 2.60 (td, J = 9.6, 2.6 Hz, 2H), 0.42 ppm (s, 6H); ^{13}C NMR (101 MHz, $CDCl_3$): δ = 160.7, 136.8, 133.9, 129.3, 127.8, 113.1, 70.6, 30.7, −3.5 ppm; IR (film) $\tilde{\nu}$ = 3393, 3070, 2958, 1768, 1733, 1428, 1406, 1252, 1190, 1152, 1118, 1041, 998, 868, 830, 782, 736, 700, 645, 471, 447 cm^{-1}. HRMS (ESI): *m/z* calcd. for $C_{12}H_{16}OSi$ [M^++H]: 205.10432; found: 205.10418.

(*E*)-4-(Dimethyl(phenyl)silyl)pent-3-en-1-ol (24). MeMgBr (3.0 M in Et_2O, 77.0 mL, 231.0 mmol) was added to a suspension of [$(PPh_3)_2NiCl_2$] (3.8 g, 5.8 mmol, 8 mol%) in toluene (50 mL). The resulting mixture

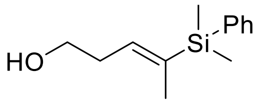

was stirred at RT for 20 min before the bulk of the solvent was removed under reduced pressure and the dark residue was suspended in toluene (461 mL). A solution of compound **23** (14.8 g, 72.1 mmol) in toluene (50 mL) was added and the resulting mixture stirred at 105 °C (bath temperature) for 30 h. After cooling to RT, the reaction was quenched with aqueous saturated NH_4Cl solution. The aqueous layer was separated and extracted with *tert*-butyl methyl ether (3 × 200 mL). The combined organic phases were washed with aqueous saturated NaCl solution, dried over $MgSO_4$ and concentrated. The residue was purified by flash chromatography (hexane/EtOAc, 10:1) to yield the title compound as a colourless oil (14.5 g, 91%). Spectral data as described above.

(*E*)-7-(Dimethyl(phenyl)silyl)oct-6-en-2-yn-4-ol (26). Dess-Martin-periodinane (9.53 g, 22.46 mmol) was

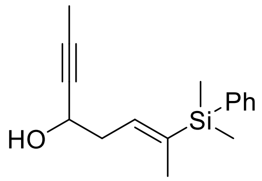

added to a solution of alcohol **24** (3.30 g, 14.97 mmol) in CH_2Cl_2 (144 mL) at 0 °C. The resulting mixture was stirred at 0 °C for 15 min, followed by stirring at RT for 4 h. The mixture was diluted with CH_2Cl_2 (50 mL) and stirred rapidly with saturated aqueous $NaHCO_3/Na_2S_2O_3$ solution (1:1 *v/v*, 50 mL) for 30 min. The aqueous layer was separated and extracted with CH_2Cl_2 (3 × 100 mL). The organic phases were dried over $MgSO_4$, filtered and concentrated. The resulting aldehyde was used without further purification.

Propynylmagnesium bromide (0.5 M in THF, 100.0 mL, 50.0 mmol) was rapidly added to a solution of the crude aldehyde in THF (390 mL) at 0 °C and the resulting mixture was stirred at 0 °C for 5 h. The reaction was quenched with saturated aqueous NH_4Cl (30 mL). The aqueous layer was separated and extracted with EtOAc (3 × 100 mL). The combined organic phases were washed with saturated aqueous NaCl solution, dried over $MgSO_4$ and concentrated. The residue was purified by flash chromatography (hexane/EtOAc, 10:1) to yield the title compound as a yellow oil (3.01 g, 78%). ^{1}H NMR (400 MHz, $CDCl_3$): δ = 7.50 (m, 2H), 7.34 (m, 3H), 5.91 (ddt, J = 6.8, 5.1, 1.8 Hz, 1H), 4.40 (tt, J = 6.4, 2.1 Hz, 1H), 2.54 (m, 2H), 1.83 (d, J = 2.1 Hz, 3H), 1.70 (m, 3H), 1.65 (s, 1H), 0.35 ppm (d, J = 0.7 Hz, 6H); ^{13}C NMR (101 MHz, $CDCl_3$): δ = 138.5, 138.4, 135.2, 133.9, 128.9, 127.7, 81.2, 80.0, 62.1, 37.2, 15.2, 3.5, −3.5 ppm; IR (film) $\tilde{\nu}$ = 3341, 3068, 2956, 2918, 2856, 1619, 1427, 1247, 1147, 1110, 1028, 830, 810, 772, 729, 699, 638, 471 cm^{-1}; HRMS (ESI): *m/z* calcd. for $C_{16}H_{22}OSi$ [M^++Na]: 281.13321; found: 281.13354.

(*E*)-*tert*-Butyl-((7-(dimethyl(phenyl)silyl)oct-6-en-2-yn-4-yl)oxy)diphenylsilane (S2). Imidazole (0.48 g, 7.04 mmol) and TBDPSCl (1.37 mL, 1.45 g, 5.28 mmol) were added to a solution of propargylic alcohol **26** (0.91 g, 3.52 mmol) in CH_2Cl_2 (45 mL) and DMF (3 mL) and the resulting mixture was stirred at RT for 1 h. The reaction was quenched with saturated aqueous NH_4Cl solution and the aqueous layer was extracted with CH_2Cl_2 (3 × 15 mL). The combined organic phases were dried over $MgSO_4$ and concentrated. The residue was purified by flash chromatography (hexane/EtOAc, 10:1) to yield the title compound as a colourless oil (1.75 g, 84%). ^{1}H NMR (400 MHz, $CDCl_3$): δ = 7.73 (ddd, *J* = 25.2, 8.0, 1.5 Hz, 4H), 7.49 (m, 2H), 7.36 (m, 9H), 5.95 (tt, *J* = 5.0, 1.8 Hz, 1H), 4.39 (ddt, *J* = 6.4, 4.3, 2.1 Hz, 1H), 2.50 (m, 2H), 1.63 (d, *J* = 2.1 Hz, 3H), 1.60 (m, 3H), 1.07 (s, 9H), 0.31 ppm (d, *J* = 2.4 Hz, 6H); ^{13}C NMR (101 MHz, $CDCl_3$): δ = 138.6, 136.6, 136.5, 136.1, 135.9, 134.0, 133.9, 129.6, 129.4, 128.7, 127.6, 127.5, 127.2, 81.1, 80.5, 63.6, 37.8, 26.9, 19.3, 15.0, 3.4, −3.4 ppm; IR (film) $\tilde{\nu}$ = 3069, 2957, 2931, 2857, 1472, 1427, 1110, 1079, 819, 773, 736, 700, 612, 505, 486 cm^{-1}; HRMS (ESI): *m*/*z* calcd. for $C_{32}H_{40}OSi_2$ [M^++Na]: 519.25099; found: 519.25144.

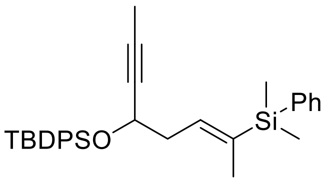

(*E*)-*tert*-Butyl-((7-iodooct-6-en-2-yn-4-yl)oxy)diphenylsilane (27). *N*-Iodosuccinimde (2.35 g, 10.45 mmol) was added to a solution of 2,6-lutidine (3.20 mL, 2.95 g, 27.50 mmol), hexafluoroisopropanol (HFIP) (20.85 mL, 33.28 g, 198.0 mmol) and compound **S2** (2.73 g, 5.50 mmol) in CH_2Cl_2 (236 mL) at −20 °C. The mixture was stirred at −20 °C for 4 h before the reaction was quenched with saturated aqueous $Na_2S_2O_3$ solution and MeOH at this temperature. The aqueous layer was separated and extracted with *tert*-butyl methyl ether (3 × 100 mL). The combined organic phases were dried over $MgSO_4$ and concentrated, and the residue was purified by flash chromatography (hexane/toluene, 10:1) to yield the title compound as a colourless oil (2.40 g, 89%). ^{1}H NMR (400 MHz, $CDCl_3$): δ = 7.72 (m, 4H), 7.39 (m, 6H), 6.19 (ddt, *J* = 9.2, 7.7, 1.5 Hz, 1H), 4.29 (ddt, *J* = 6.1, 4.1, 2.1 Hz, 1H), 2.33 (m, 2H), 2.27 (m, 3H), 1.68 (d, *J* = 2.1 Hz, 3H), 1.07 ppm (s, 9H); ^{13}C NMR (101 MHz, $CDCl_3$): δ = 136.6, 136.1, 135.9, 133.7, 133.6, 129.7, 129.5, 127.6, 127.3, 96.2, 81.6, 79.8, 62.8, 39.6, 27.8, 26.9, 19.2, 3.5 ppm; IR (film) $\tilde{\nu}$ = 2930, 2856, 1427, 1105, 1071, 1052, 945, 821, 737, 699, 610, 501, 485 cm^{-1}; HRMS (ESI): *m*/*z* calcd. for $C_{24}H_{29}OSi$ [*M*+Na^+]: 511.09246; found: 511.09265.

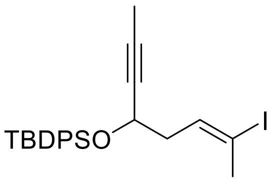

(*E*)-(5-Iodopent-2-en-2-yl)dimethyl(phenyl)silane (25). PPh_3 (384 mg, 1.46 mmol), imidazole (99.6 mg, 1.46 mmol) and iodine (371 mg, 1.46 mmol) were added to a solution of alcohol **24** (215 mg, 0.976 mmol) in CH_2Cl_2 (4 mL) at 0 °C. The mixture was warmed to RT over 30 min and the reaction was quenched with water (2 mL). The aqueous layer was separated and extracted with pentane (3 × 10 mL). The combined organic phases were dried

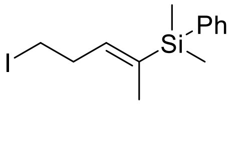

over $MgSO_4$ and concentrated, and the residue was purified by flash chromatography (pentane) to yield the title compound as a colourless oil (300 mg, 93%). ^{1}H NMR (400 MHz, $CDCl_3$): δ = 7.51 (m, 2H), 7.35 (dd, *J* = 4.9, 1.9 Hz, 3H), 5.72 (m, 1H), 3.17 (t, *J* = 7.3 Hz, 2H), 2.73 (tdd, *J* = 7.5, 6.6, 0.9 Hz, 2H), 1.67 (m, 3H), 0.35 ppm (s, 6H); ^{13}C NMR (101 MHz, $CDCl_3$): δ = 139.0, 138.1, 137.5, 134.0, 128.9, 127.7, 32.6, 15.0, 4.9, −3.5 ppm; IR (film) $\tilde{\nu}$ = 3067, 3007, 2956, 1615, 1427, 1245, 1171, 1110, 950, 831, 813, 773, 731, 700, 638, 473 cm^{-1}; HRMS (ESI): *m/z* calcd. for $C_{13}H_{19}ISi$ [M^+]: 330.02953; found: 330.02986.

***tert*-Butyl-(((6*E*,10*E*)-11-(dimethyl(phenyl)silyl)-7-methyldodeca-6,10-dien-2-yn-4-yl) oxy)diphenyl-silane (28).** A thoroughly dried Schlenk flask was charged with LiCl (95.3 mg, 2.3 mmol) and Zn dust (267.5 mg, 4.1 mmol) and was then heated under vacuum. After reaching ambient temperature, THF (24 mL) was added, followed by diiodoethane (26.0 mg, 92.1 µmol) and TMSCl (23.4 µL, 20.0 mg, 184.2 µmol). The resulting suspension was stirred for 2 min at reflux temperature to ensure activation of the zinc dust.

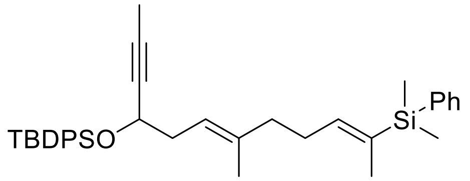

Alkyl iodide **25** (699.8 mg, 2.1 mmol) was added and the mixture was stirred at RT for 3 h before it was filtered through a glasswool filter that was rinsed with THF (2 mL). Alkenyl iodide **27** (793.1 mg, 1.6 mmol) was added to the solution of the organozinc derivative, followed by $Pd(PPh_3)_4$ (106.5 mg, 92.1 µmol, 6 mol%). The resulting mixture was stirred at RT for 3 h before it was diluted with toluene (10 mL) and filtered through a plug of Celite, which was carefully rinsed with toluene (20 mL). The combined filtrates were concentrated and the residue purified by flash chromatography (hexane/toluene, 10:1) to give the title compounds as a colourless oil (753.2 mg, 82%). ^{1}H NMR (400 MHz, $CDCl_3$): δ = 7.72 (ddd, *J* = 25.1, 7.9, 1.6 Hz, 4H), 7.48 (m, 2H), 7.36 (m, 9H), 5.78 (m, 1H), 5.18 (m, 1H), 4.28 (ddt, *J* = 6.5, 4.3, 2.1 Hz, 1H), 2.35 (td, *J* = 7.0, 3.5 Hz, 2H), 2.18 (m, 2H), 2.02 (t, *J* = 7.8 Hz, 2H), 1.66 (d, *J* = 2.1 Hz, 3H), 1.63 (s, 3H), 1.51 (s, 3H), 1.07 (s, 9H), 0.30 ppm (s, 6H); ^{13}C NMR (101 MHz, $CDCl_3$): δ = 141.3, 138.8, 137.4, 136.1, 135.9, 134.02, 133.95, 133.93, 133.90, 129.5, 129.4, 128.7, 127.6, 127.4, 127.2, 119.7, 80.78, 80.75, 64.1, 39.2, 37.5, 27.1, 26.9, 19.3, 16.2, 14.7, 3.5, −3.4 ppm; IR (film) $\tilde{\nu}$ = 3069, 2957, 2931, 2857, 1617, 1472, 1428, 1247, 1111, 1074, 940, 815, 773, 737, 701, 613, 505 cm^{-1}; HRMS (ESI): *m/z* calcd. for $C_{37}H_{48}OSi_2$ [M+Na^+]: 587.31359; found: 587.31403.

(6*E*,10*E*)-11-(Dimethyl(phenyl)silyl)-7-methyldodeca-6,10-dien-2-yn-4-ol (29). TBAF (1 M in THF, 4.67 mL, 4.67 mmol) was added to a solution of compound **28** (1.32 g, 2.34 mmol) in THF (57 mL) at 0 °C. The mixture was stirred at 0 °C for 10 min and for another 5 h at RT. The reaction was quenched with saturated aqueous NH_4Cl solution and the aqueous layer was separated and extracted with EtOAc (3 × 100 mL). The combined

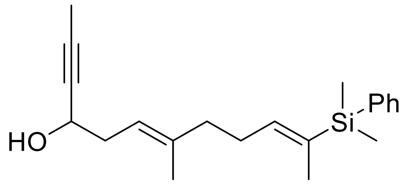

organic phases were dried over $MgSO_4$ and concentrated, and the residue was purified by flash chromatography (hexane/EtOAc, 10:1) to yield the title compound as a colourless oil (637.0 mg, 84%). ^{1}H NMR (400 MHz, $CDCl_3$): *δ* = 7.49 (m, 2H), 7.34 (m, 3H), 5.78 (tq, *J* = 6.7, 1.8 Hz, 1H), 5.24 (q, *J* = 1.3 Hz, 1H), 4.31 (m, 1H), 2.41 (m, 2H), 2.25 (m, 2H), 2.12 (dd, *J* = 8.6, 6.5 Hz, 2H), 1.84 (d, *J* = 2.1 Hz, 3H), 1.76 (d, *J* = 5.7 Hz, 1H), 1.66 (dd, *J* = 1.7, 0.9 Hz, 6H), 0.32 ppm (s, 6H); ^{13}C NMR (101 MHz, $CDCl_3$): *δ* = 141.0, 139.4, 138.8, 134.3, 133.9, 128.8, 127.6, 118.8, 80.9, 80.2, 62.3, 39.2, 36.8, 27.0, 16.4, 14.8, 3.6, −3.5 ppm; IR (film) $\tilde{\nu}$ = 3365, 2955, 2919, 2855, 1617, 1428, 1247, 1110, 1039, 999, 831, 814, 773, 731, 701 cm^{-1}; HRMS (ESI): *m*/*z* calcd. for $C_{21}H_{30}OSi$ [*M*+Na$^+$]: 349.19581; found: 349.19563.

3-Iodo-2-((*E*)-4-iodopent-3-en-1-yl)-2-methyl-5-(prop-1-yn-1-yl)tetrahydrofuran (30). *N*-Iodosuccinimde (20.1 mg, 89.4 µmol) was added to a solution of compound **29** (14.6 mg, 44.7 µmol) in hexafluoroisopropanol (HFIP) (1.2 mL). The mixture was stirred at 0 °C for 2 min before the reaction was quenched with saturated aqueous $Na_2S_2O_3$ solution. The aqueous layer was separated and extracted with *tert*-butyl methyl ether (3 × 5 mL). The combined organic phases were dried over $MgSO_4$ and concentrated, and the residue was purified by flash chromatography (hexane/EtOAc, 20:1) to yield the title compounds as a colourless oil (14.4 mg, *cis*/*trans* = 42:58, 73%). *Spectral data of the* ***cis-isomer***: ^{1}H NMR (600 MHz, $CDCl_3$): *δ* = 6.13 (m, 1H), 4.48 (ddq, *J* = 8.8, 6.5, 2.1 Hz, 1H), 3.90 (dd, *J* = 11.8, 7.1 Hz, 1H), 2.75 (dt, *J* = 12.7, 6.9 Hz, 1H), 2.51 (m, 1H), 2.37 (d, *J* = 1.3 Hz, 3H), 2.14 (dd, *J* = 7.1, 4.0 Hz, 2H), 1.84 (d, *J* = 2.2 Hz, 3H), 1.74 (m, 1H), 1.59 (m, 1H), 1.46 ppm (s, 3H); ^{13}C NMR (151 MHz, $CDCl_3$): *δ* = 140.4, 94.0, 84.3, 81.6, 78.8, 66.9, 44.5, 37.3, 27.5, 26.0, 25.7, 25.2, 3.7 ppm.

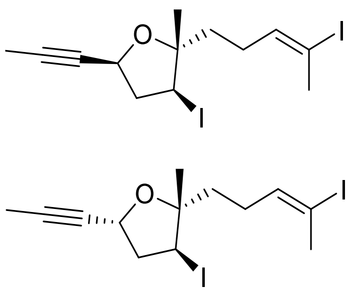

Spectral data of the ***trans-isomer***: ^{1}H NMR (600 MHz, $CDCl_3$): *δ* = 6.17 (tt, *J* = 7.7, 1.6 Hz, 1H), 4.66 (ddq, *J* = 8.5, 4.3, 2.1 Hz, 1H), 4.16 (dd, *J* = 9.4, 7.4 Hz, 1H), 2.60 (m, 2H), 2.39 (d, *J* = 1.3 Hz, 3H), 2.29 (m, 2H), 1.83 (d, *J* = 2.1 Hz, 3H), 1.74 (m, 2H), 1.34 ppm (s, 3H); ^{13}C NMR (151 MHz, $CDCl_3$): *δ* = 140.6, 93.9, 84.7 81.7, 78.8, 66.7, 44.7, 37.4, 27.9, 27.5, 25.7, 25.5, 3.7 ppm; IR (film) $\tilde{\nu}$ = 2971, 2918, 2851, 2243, 1784, 1716, 1677, 1635, 1592, 156, 1448, 1428, 1356, 1260, 1172, 1155, 1108, 10590, 1015, 952, 917, 804, 737, 701, 664, 618 cm^{-1}; HRMS (ESI): *m*/*z* calcd. for $C_{13}H_{18}OI_2$ [*M*$^+$+Na]: 466.93393; found: 466.93403.

(6*E*,10*E*)-11-Iodo-7-methyldodeca-6,10-dien-2-yn-4-ol (31). *N*-Iodosuccinimde (454.9 mg, 2.0 mmol) was added to a solution of compound **29** (617.0 mg, 1.9 mmol) in hexafluoroisopropanol (HFIP) (50 mL) and HOAc (1.1 mL, 18.9 mmol). The mixture was stirred at 0 °C for 5 min before the reaction was quenched with saturated aqueous $Na_2S_2O_3$ solution. The aqueous layer was separated and extracted with *tert*-butyl methyl ether (3 × 50 mL).

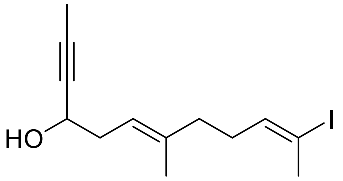

The combined organic phases were dried over $MgSO_4$ and concentrated, and the residue was purified by flash chromatography (hexane/EtOAc, 10:1) to yield the title compound as a colourless oil (423.2 mg, 70%). ^{1}H NMR (400 MHz, $CDCl_3$): δ = 6.13 (tt, J = 5.7, 1.6 Hz, 1H), 5.24 (ddt, J = 8.6, 7.3, 1.3 Hz, 1H), 4.34 (ddt, J = 6.2, 4.2, 2.1 Hz, 1H), 2.41 (ddt, J = 7.2, 6.3, 0.8 Hz, 2H), 2.36 (dt, J = 1.7, 0.9 Hz, 3H), 2.13 (m, 4H), 1.86 (d, J = 2.1 Hz, 3H), 1.64 ppm (m, 3H); ^{13}C NMR (101 MHz, $CDCl_3$): δ = 140.7, 138.1, 119.6, 93.6, 81.0, 80.1, 62.3, 38.7, 36.8, 29.0, 27.5, 16.3, 3.6 ppm; IR (film) $\tilde{\nu}$ = 3391, 2917, 2854, 1765, 1714, 1634, 1430, 1377, 1256, 1175, 1135, 1104, 1053, 880, 839, 621 cm^{-1}; HRMS (ESI): *m/z* calcd. for $C_{13}H_{19}OI$ [M^++Na]: 341.03728; found: 341.03766.

Completion of the Total Syntheses

Compound 33. A solution of 9-H-9-BBN (173.5 mg, 1.4 mol) in THF (4 mL) was added to a solution of compound **19** (530.0 mg, 947.7 µmol, 24 % *w/w* in pentane) in THF (15 mL) at 0 °C. The ice bath was removed and the mixture was stirred at RT for 3 h. Water (1.5 mL, 1.5 g, 83.3 mmol) and $Ba(OH)_2{\cdot}(H_2O)_8$ (448.5 mg, 1.4 mmol) were sequentially added and the mixture was stirred for 15 min. Alkenyl iodide **37** (232.1 mg, 729.6 µmol) and Pd(dppf) (69.3 mg, 94.8 µmol, 10 mol%) were introduced and the resulting mixture was stirred at RT for 2 h. The reaction was quenched with saturated aqueous NH_4Cl solution. The aqueous layer was separated and extracted with *tert*-butyl methyl ether (3 × 50 mL), the combined organic phases were dried over $MgSO_4$ and concentrated, and the residue was purified by flash chromatography (hexane/EtOAc, 20:1) to yield the title compound as a colourless oil (165.3 mg, 69%). ^{1}H NMR (400 MHz, $CDCl_3$) δ = 5.23 (ddt, J = 7.4, 6.1, 1.3 Hz, 1H), 5.13 (ddd, J = 6.9, 5.5, 2.7 Hz, 1H), 4.32 (ddt, J = 6.1, 4.1, 2.1 Hz, 1H), 2.41 (t, J = 6.8 Hz, 2H), 2.07 (m, 6H), 1.85 (d, J = 2.1 Hz, 3H), 1.81 (d, J = 2.2 Hz, 3H), 1.65 (s, 3H), 1.62 (s, 3H), 1.45 (m, 2H), 1.09 (dq, J = 8.5, 2.2 Hz, 1H), 1.05 (s, 3H), 1.03 (s, 3H), 0.63 ppm (dt, J = 8.6, 7.1 Hz, 1H); ^{13}C NMR (101 MHz, $CDCl_3$) δ = 139.8, 135.6, 124.1, 118.5, 80.8, 80.2, 77.6, 74.9, 62.3, 39.9, 39.3, 36.8, 29.0, 27.7, 26.5, 24.3, 21.0, 17.8, 16.4, 16.1, 16.0, 3.7, 3.6 ppm; IR (film) $\tilde{\nu}$ = 3394, 2981, 2918, 2858, 1450, 1378, 1134, 1038, 881, 831 cm^{-1}; HRMS (ESI): *m/z* calcd. for $C_{23}H_{34}O$ [M^++H]: 327.26824; found: 327.26802.

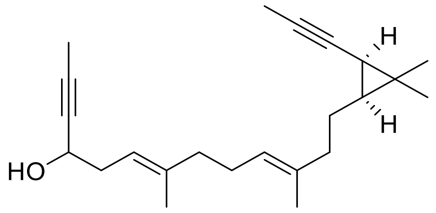

Compound 39. Prepared analogously as a colourless oil (222.3 mg, 82%). ^{1}H NMR (600 MHz, $CDCl_3$) δ = 5.23 (m, 1H), 5.11 (tq, J = 7.0, 1.5 Hz, 1H), 4.32 (tdq, J = 6.0, 4.1, 1.8 Hz, 1H), 2.41 (m, 2H), 2.07 (m, 6H), 1.85 (d, J = 2.1 Hz, 3H), 1.80 (d, J = 2.1 Hz, 3H), 1.65 (s, 3H), 1.59 (s, 3H), 1.45 (m, 1H), 1.37 (m, 1H), 1.15 (s, 3H), 1.04 (s, 3H), 0.69 (dt, J = 4.4, 2.2 Hz, 1H), 0.62 ppm (td, J = 7.2, 5.2 Hz, 1H); ^{13}C NMR

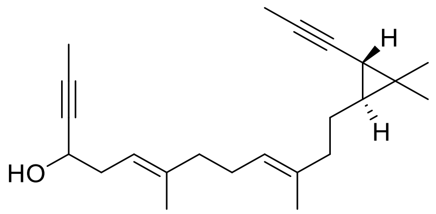

(151 MHz, $CDCl_3$) δ = 139.7, 135.0, 124.3, 118.6, 80.8, 80.2, 80.1, 73.3, 62.3, 39.8, 39.6, 36.8, 33.7, 27.7, 26.5, 23.5, 22.4, 20.1, 20.0, 16.4, 16.0, 3.7, 3.6 ppm; IR (film) $\tilde{\nu}$ = 3411, 2970, 2918, 2857, 1667, 1450, 1379, 1333, 1124, 1036, 881, 839 cm^{-1}; HRMS (ESI): *m/z* calcd. for $C_{23}H_{34}O$ [M^++Na]: 349.25018; found: 349.24996.

Cycloalkynes 34 and 35. Powdered MS 5 (100 mg) and MS 4 (100 mg) [pre-activated at 140 °C under vacuum overnight] were added to a solution of diyne **33** (45.7 mg, 140.0 µmol) in toluene (150 mL) and the mixture was stirred at RT for 1 h. In a second Schlenk flask, a solution of trisilanol **38** (24.2 mg, 30.8 µmol) in toluene (1 mL) was added to the molybdenum complex **37** (18.6 mg, 28.0 µmol) and the mixture was stirred at RT for 5 min. The resulting catalyst solution was added to the preheated solution of the diyne at reflux temperature. After stirring for 25 min, the mixture was cooled to RT before it was filtered through a pad of Celite, which was rinsed with toluene. The combined filtrates were concentrated and the residue was purified by flash chromatography (hexane/EtOAc, 20:1) to yield the title compounds as a colourless oil (22.6 mg, 83.0 µmol, 60%).

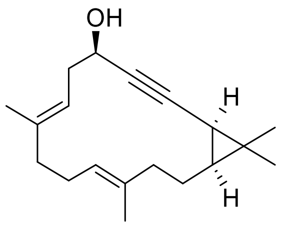

34 (1*R*,4*R*,14*S*)

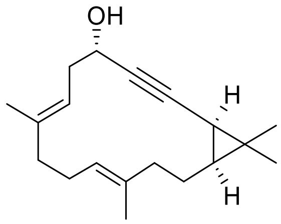

35 (1*R*,4*S*,14*S*)

Analytical and spectral data of macrocycle **34:** $[\alpha]_D^{20}$ = −4.2 (0.13 g/100 mL, $CHCl_3$); ^1H NMR (600 MHz, $CDCl_3$): δ = 5.06 (m, 2H), 4.40 (ddd, *J* = 9.7, 4.1, 1.7 Hz, 1H), 2.47 (ddd, *J* = 13.9, 6.7, 4.1 Hz, 1H), 2.27 (ddd, *J* = 14.1, 9.9, 7.1 Hz, 1H), 2.17 (m, 1H), 2.11 (m, 1H), 2.01 (m, 4H), 1.84 (dddd, *J* = 13.6, 11.2, 6.8, 2.6 Hz, 1H), 1.61 (d, *J* = 1.3 Hz, 3H), 1.57 (m, 3H), 1.18 (dd, *J* = 8.3, 1.7 Hz, 1H), 1.07 (s, 3H), 1.06 (m, 1H), 1.05 (s, 3H), 0.75 ppm (ddd, *J* = 11.1, 8.4, 2.6 Hz, 1H); ^{13}C NMR (151 MHz, $CDCl_3$): δ = 136.7, 135.9, 124.1, 120.0, 84.5, 81.2, 63.0, 39.7, 39.6, 36.8, 30.6, 27.4, 26.4, 23.9, 22.1, 17.7, 16.1, 15.8, 15.6 ppm. IR (film) $\tilde{\nu}$ = 3278, 2948, 2929, 2852, 1667, 1452, 1378, 1325, 1294, 1261, 1092, 1016, 853, 832, 537, 525 cm^{-1}. HRMS (ESI): *m/z* calcd. for $C_{19}H_{28}O$ [M^++Na]: 295.20323; found: 295.20323.

Analytical and spectral data of macrocycle **35:** $[\alpha]_D^{20}$ = −80.9 (0.11 g/100 mL, $CHCl_3$); ^1H NMR (600 MHz, $CDCl_3$): δ = 5.11 (m, 2H), 4.52 (ddd, *J* = 7.6, 3.4, 1.8 Hz, 1H), 2.49 (dt, *J* = 14.1, 8.1 Hz, 1H), 2.31 (m, 1H), 2.16 (m, 2H), 2.04 (m, 4H), 1.81 (m, 1H), 1.63 (s, 6H), 1.20 (dd, *J* = 8.3, 1.8 Hz, 1H), 1.17 (m, 1H), 1.05 (s, 3H), 1.04 (s, 3H), 0.74 ppm (ddd, *J* = 10.8, 8.3, 2.5 Hz, 1H); ^{13}C NMR (151 MHz, $CDCl_3$): δ = 137.7, 135.9, 123.8, 119.8, 84.4, 80.9, 62.6, 39.8, 39.3, 36.1, 30.5, 27.4, 25.7, 24.1, 22.2, 18.0, 16.3, 16.2, 16.1 ppm; IR (film) $\tilde{\nu}$ = 3354, 2980, 2918, 2857, 2224, 1667, 1450, 1377, 1261, 1095, 1035, 992, 883, 801, 525 cm^{-1}. HRMS (ESI): *m/z* calcd. for $C_{19}H_{28}O$ [M^++Na]: 295.20323; found: 295.20317.

Cycloalkynes 40 and 41. Prepared analogously as a colourless oil (42.5 mg, 76%). Analytical and spectral data of compound **40**: $[\alpha]_D^{20}$ = +47.6 (0.45 g/100 mL); ^{1}H NMR (600 MHz, $CDCl_3$): δ = 5.28 (tq, J = 7.3, 1.4 Hz, 1H), 5.22 (m, 1H), 4.49 (t, J = 5.2 Hz, 1H), 2.38 (m, 2H), 2.19 (m, 6H), 1.87 (m, 1H), 1.79 (s, 1H), 1.59 (s, 6H), 1.13 (s, 3H), 1.04 (s, 3H), 0.92 (m, 1H), 0.65 (s, 1H), 0.64 ppm (m, 1H) ; ^{13}C NMR (151 MHz, $CDCl_3$): δ = 137.1, 133.1, 126.2, 119.0, 87.8, 78.0, 62.8, 39.0, 38.5, 36.2, 34.0, 24.8, 24.4, 23.5, 23.4, 20.4, 19.2, 15.8, 15.0 ppm. IR (film) $\tilde{\nu}$ = 3358, 2969, 2923, 2857, 2232, 1437, 1378, 1308, 1256, 1098, 1037, 864, 896, 823 cm^{-1}. HRMS (ESI): m/z calcd. for $C_{19}H_{28}O$ [M^+]: 272.21347; found: 272.21351.

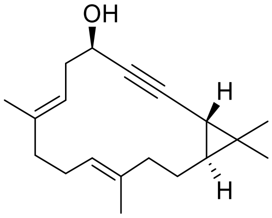

(1*S*,4*R*,14*S*)

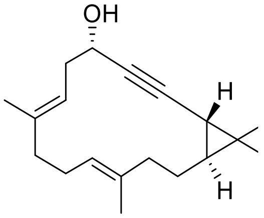

(1*S*,4*S*,14*S*)

Analytical and spectral data of compound **41**: $[\alpha]_D^{20}$ = −5.5 (1.50 g/100 mL); ^{1}H NMR (600 MHz, $CDCl_3$): δ = 5.13 (m, 2H), 4.44 (m, 1H), 2.41 (m, 1H), 2.34 (m, 2H), 2.16 (m, 4H), 2.08 (td, J = 12.5, 3.3 Hz, 1H), 1.86 (ddt, J = 14.0, 12.3, 3.1 Hz, 1H), 1.70 (s, 1H), 1.59 (s, 3H), 1.56 (s, 3H), 1.16 (s, 3H), 1.05 (s, 3H), 0.89 (m, 1H), 0.67 (ddd, J = 11.6, 5.2, 3.0 Hz, 1H), 0.63 ppm (dd, J = 5.3, 2.5 Hz, 1H); ^{13}C NMR (151 MHz, $CDCl_3$): δ = 136.9, 133.3, 126.3, 120.0, 87.8, 79.1, 63.1, 39.0, 38.6, 37.4, 34.3, 25.0, 24.5, 24.0, 23.6, 20.5, 19.2, 15.4, 15.2 ppm. IR (film) $\tilde{\nu}$ = 3328, 2969, 2923, 2856, 2226, 1440, 1378, 1306, 1256, 1113, 1029, 965, 867, 825 cm^{-1}. HRMS (ESI): m/z calcd. for $C_{19}H_{28}O$ [M^+]: 272.21347; found: 272.21331.

Compound 36. A solution of Bu_3SnH (0.2 M in CH_2Cl_2, 0.8 mL, 157.5 µmol) was added dropwise to a solution of $[Cp^*RuCl]_4$ (1.4 mg, 1.1 µmol, 2.5 mol%) and alkyne **35** (14.3 mg, 52.5 µmol) in CH_2Cl_2 (2 mL) at RT. The mixture was stirred for 2 h before it was concentrated. The residue was purified by flash chromatography (hexane/EtOAc, 20:1) to yield the title compound as a colourless oil (26.1 mg, 46.3 µmol, 88%). $[\alpha]_D^{20}$ = −30.0 (0.02 g/100 mL, $CHCl_3$); ^{1}H NMR (600 MHz, $CDCl_3$): δ = 5.81 (dd, J = 125.9, 120.5 Hz, 1H), 4.99 (m, 1H), 4.86 (td, J = 6.8, 3.6 Hz, 1H), 4.11 (dt, J = 11.0, 3.0 Hz, 1H), 2.39 (ddd, J = 13.7, 5.7, 3.8 Hz, 1H), 2.26 (dt, J = 13.4, 6.1 Hz, 1H), 2.12 (m, 4H), 1.92 (m, 2H), 1.81 (dddd, J = 14.1, 7.1, 5.7, 1.5 Hz, 1H), 1.62 (d, J = 1.3 Hz, 3H), 1.59 (d, J = 1.3 Hz, 3H), 1.51 (m, 6H), 1.43 (d, J = 2.6 Hz, 1H), 1.34 (h, J = 7.3 Hz, 6H), 1.17 (m, 1H), 1.12 (dd, J = 10.4, 8.5 Hz, 1H), 1.06 (s, 3H), 0.99 (m, 6H), 0.95 (s, 3H), 0.90 (t, J = 7.3 Hz, 9H), 0.67 ppm (ddd, J = 10.3, 8.5, 1.4 Hz, 1H); ^{13}C NMR (151 MHz, $CDCl_3$): δ = 147.1, 139.4, 134.89, 134.86, 124.2, 120.4, 82.5, 39.8, 38.6, 36.4, 32.4, 30.6, 29.3, 28.9, 27.5, 24.1, 23.1, 22.1, 17.3, 16.9, 15.7, 13.7, 11.3 ppm; ^{119}Sn NMR (224 MHz, $CDCl_3$) δ = −57.2 ppm; IR (film) $\tilde{\nu}$ = 3424, 2954, 2923, 2870, 2854, 1674, 1606, 1456, 1376, 1260, 1081, 1019, 866, 799, 665, 597,504 cm^{-1}; HRMS (ESI): m/z calcd. for $C_{31}H_{56}OSn$ [M^++Na]: 587.3245; found: 587.32415.

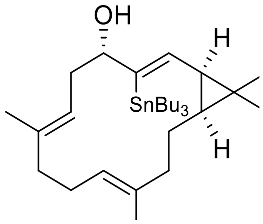

Compound S2. Prepared analogously as a colourless oil (12.8 mg, 79%). $[\alpha]_D^{20}$ = −16.8 (0.74 g/100 mL, $CHCl_3$); ^{1}H NMR (400 MHz, $CDCl_3$): δ = 5.96 (dddd, *J* = 129.9, 10.4, 3.0, 1.7 Hz, 1H), 4.98 (m, 2H), 4.43 (m, 1H), 2.58 (dt, *J* = 14.9, 7.5 Hz, 1H), 2.26 (m, 2H), 2.13 (m, 3H), 2.00 (m, *J* = 17.0, 10.2, 5.2 Hz, 1H), 1.88 (m, 2H), 1.65 (d, *J* = 1.3 Hz, 3H), 1.60 (s, 3H), 1.50 (m, *J* = 16.5, 9.8, 4.7, 2.6 Hz, 6H), 1.33 (m, 6H), 1.20 (m, 1H), 1.11 (dd, *J* = 10.3, 8.4 Hz, 1H), 1.06 (s, 3H), 0.95 (m, 9H), 0.90 (t, *J* = 7.3 Hz, 9H), 0.72 ppm (ddd, *J* = 10.9, 8.4, 1.4 Hz, 1H) ppm; ^{13}C NMR (101 MHz, $CDCl_3$): δ = 144.2, 136.9, 135.4, 134.3, 125.0, 117.4, 74.5, 40.0, 38.4, 34.2, 32.0, 31.6, 29.3, 28.9, 27.5, 24.3, 24.1, 21.9, 17.3, 17.5, 15.6, 13.7, 10.4 ppm; ^{119}Sn NMR (149 MHz, $CDCl_3$) δ = −54.6 ppm; IR (film) $\tilde{\nu}$ = 3453, 2953, 2921, 2871, 2853, 1608, 1455, 1376, 1289, 1261, 1058, 1020, 874, 802, 688, 666, 593 cm^{-1}; HRMS (ESI): *m/z* calcd. for $C_{31}H_{56}OSn$ [M^++Na]: 587.3245; found: 587.32483.

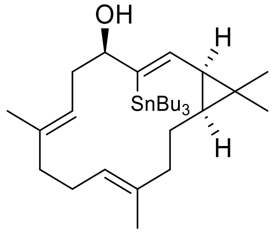

Compound 42 and Isomer. Prepared analogously as a colourless oil (6.9 mg, 65%). $[\alpha]_D^{20}$ = +7.0 (0.57 g/100 mL, $CHCl_3$); ^{1}H NMR (400 MHz, $CDCl_3$): δ = 5.54 (dd, *J* = 126.4, 10.0 Hz, 1H), 5.08 (d, *J* = 5.9 Hz, 1H), 4.89 (m, 1H), 4.20 (m, 1H), 2.44 (m, 1H), 2.05 (m, 7H), 1.58 (m, 3H), 1.56 (s, 3H), 1.50 (m, 7H), 1.34 (dq, *J* = 14.2, 7.3 Hz, 6H), 1.06 (s, 3H), 1.03 (s, 3H), 0.99 (m, 7H), 0.90 (t, *J* = 7.3 Hz, 9H), 0.73 (m, 1H), 0.47 (dt, *J* = 11.0, 4.5 Hz, 1H); ^{13}C NMR (101 MHz, $CDCl_3$): δ = 145.1, 143.7, 134.8, 132.7, 126.3, 120.7, 81.8, 38.9, 38.0, 35.6, 33.8, 33.5, 29.3, 27.6, 24.4, 24.2, 23.9, 22.9, 22.0, 17.1, 14.9, 13.7, 11.1 ppm; ^{119}Sn NMR (149 MHz, $CDCl_3$) : δ = −56.35 ppm; IR (film) $\tilde{\nu}$ = 3423, 1954, 2923, 2870, 2853, 1608, 1455, 1376, 1259, 1182, 1119, 1020, 964, 897, 877, 691, 669, 593, 541 cm^{-1}. HRMS (ESI): *m/z* calcd. for $C_{31}H_{56}OSn$ [M^++Na]: 587.3245; found: 587.32466.

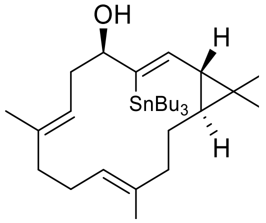

In this case, a second isomer was obtained, which was identified as the corresponding "*alpha,cis*"-adduct (1.3 mg, 12%): ^{1}H NMR (600 MHz, $CDCl_3$) δ = 5.22 (dd, *J* = 75.0, 7.0, 1.5 Hz, 1H), 5.21 (m, 1H), 5.10 (t, *J* = 6.7 Hz, 1H), 4.70 (s, 1H), 2.55 (ddd, *J* = 14.0, 8.4, 5.1 Hz, 1H), 2.20 (m, 4H), 2.16 (d, *J* = 5.9 Hz, 1H), 2.12 (m, 2H), 1.92 (m, 1H), 1.77 (m, 1H), 1.62 (s, 3H), 1.64 (s, 3H), 1.47 (m, 7H), 1.30 (m, 7H), 1.05 (s, 3H), 1.02 (s, 3H), 0.87 (m, 15H), 0.55 ppm (dt, *J* = 8.2, 5.1 Hz, 1H). ^{13}C NMR (151 MHz, $CDCl_3$) δ = 146.0, 140.1, 137.3, 134.6, 124.4, 118.6, 39.2, 38.3, 35.0, 34.8, 32.7, 29.1, 27.4, 25.8, 24.7, 23.0, 21.8, 21.4, 17.8, 16.7, 13.7, 9.9, 1.0 ppm.

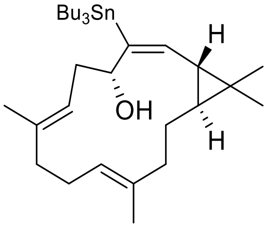

Compound 43. Prepared analogously as a colourless oil (18.7 mg, 74%). $[\alpha]_D^{20}$ = −22.6 (1.73 g/100 mL); ^{1}H NMR (400 MHz, $CDCl_3$): δ = 5.75 (ddd, J = 132.2, 9.8, 1.2 Hz, 1H), 5.00 (dt, J = 11.4, 6.1 Hz, 2H), 4.38 (m, 1H), 2.55 (dt, J = 15.9, 8.2 Hz, 1H), 2.26 (m, 2H), 2.04 (m, 6H), 1.57 (s, 3H), 1.53 (s, 3H), 1.50 (m, 6H), 1.34 (m, 6H), 1.21 (d, J = 6.2 Hz, 1H), 1.05 (s, 3H), 1.04 (s, 3H), 0.96 (m, 7H), 0.90 (t, J = 7.3 Hz, 9H), 0.68 (m, 1H), 0.52 ppm (ddd, J = 10.9, 4.9, 3.2 Hz, 1H); ^{13}C NMR (101 MHz, $CDCl_3$): δ = 142.8, 140.1, 133.7, 133.0, 126.0, 121.1, 74.4, 38.8, 38.4, 34.8, 34.1, 33.8, 29.3, 27.6, 24.4, 24.3, 23.8, 23.0, 21.9, 15.9, 14.8, 13.7, 10.2 ppm; ^{119}Sn NMR (149 MHz, $CDCl_3$): δ = −49.71 ppm; IR (film) $\tilde{\nu}$ = 3457, 2955, 2923, 2871, 2853, 1612, 1455, 1376, 1118, 1070, 1018, 962, 897, 872, 687, 665, 596 cm^{-1}. HRMS (ESI): m/z calcd. for $C_{31}H_{56}OSn$ [M^++Na]: 587.3245; found: 587.32455.

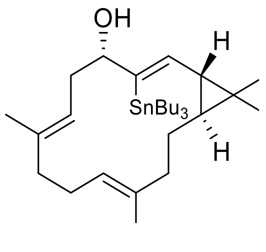

Compound S4. $Pd(PPh_3)_4$ (2.7 mg, 2.3 µmol, 5 mol%) was added to a solution of alkenyl stannane **36** (26.1 mg, 46.3 mmol) and $[Ph_2PO_2]^-[Bu_4N]^+$ (23.4 mg, 51.0 µmol) in DMF (0.2 mL) and the mixture was stirred for 10 min. Methyl iodide (4.3 µL, 9.9 mg, 69.5 µmol) was added, immediately followed (after 10 sec !) by CuTC (9.3 mg, 48.6 µmol). The resulting mixture was stirred at RT for 4 h. At this point, additional $Pd(PPh_3)_4$ (1.4 mg, 1.2 mmol, 2.5 mol%), methyl iodide (2.2 µL, 5.0 mg, 34.8 µmol), and CuTC (4.7 mg, 24.3 µmol) were added sequentially (10 sec time difference between MeI and CuTC) and stirring was continued for another 2 h. The reaction was quenched with aqueous Et_3N (0.1 ml), the mixture diluted with *tert*-butyl methyl ether and washed with aqueous NH_3 (25%)/NH_4Cl solution (1:9). The aqueous layer was separated and extracted with *tert*-butyl methyl ether (3 × 10 mL). The combined organic phases were dried over $MgSO_4$ and concentrated, and the residue was purified by flash chromatography (hexane/EtOAc, 10:1) to yield the title compound as a colourless oil (2.4 mg, 67%). $[\alpha]_D^{20}$ = −77.4 (0.28 g/100 mL, $CHCl_3$); ^{1}H NMR (600 MHz, $CDCl_3$): δ = 5.10 (m, 1H), 4.94 (ddt, J = 6.5, 5.2, 1.3 Hz, 1H), 4.78 (ddq, J = 7.7, 5.2, 1.3 Hz, 1H), 4.08 (m, 1H), 2.41 (ddd, J = 14.4, 11.2, 8.6 Hz, 1H), 2.31 (m, 1H), 2.20 (dt, J = 14.1, 6.9 Hz, 1H), 2.09 (m, 3H), 1.87 (m, 2H), 1.73 (m, 1H), 1.69 (d, J = 1.3 Hz, 3H), 1.60 (s, 3H), 1.58 (s, 3H), 1.42 (d, J = 2.8 Hz, 1H), 1.26 (m, 1H), 1.07 (s, 3H), 0.96 (s, 3H), 0.63 ppm (ddd, J = 10.2, 8.8, 1.4 Hz, 1H); ^{13}C NMR (151 MHz, $CDCl_3$): δ = 137.2, 135.2, 135.0, 125.9, 123.4, 120.5, 79.4, 40.4, 39.3, 33.1, 31.5, 28.8, 25.5, 24.0, 23.6, 20.5, 16.7, 16.1, 15.7, 10.4 ppm; IR (film) $\tilde{\nu}$ = 3342, 2918, 2858, 1448, 1376, 1013, 871 cm^{-1}; HRMS (ESI): m/z calcd. for $C_{20}H_{32}O$ [M^++Na]: 311.23453; found: 311.23490.

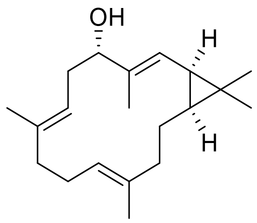

Depressin (2). MnO_2 (43.4 mg, 0.5 mmol) was added to a solution of alcohol **S4** (4.8 mg, 16.6 µmol) in

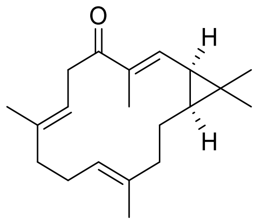

CH_2Cl_2 (2 mL). The suspension was stirred at RT for 4 h before it was filtered through a plug of silica, which was carefully rinsed with *tert*-butyl methyl ether. The combined filtrates were concentrated and the residue was purified by flash chromatography (hexane/EtOAc, 20:1) to yield the title compound as a colourless oil (3.5 mg, 73%). $[\alpha]_D^{20}$ = −85.0 (0.02 g/100 mL, $CHCl_3$); ^1H NMR (600 MHz, $CDCl_3$): δ = 6.38 (dq, J = 10.2, 1.3 Hz, 1H), 5.07 (ddt, J = 8.7, 5.9, 1.4 Hz, 1H), 4.84 (dd, J = 9.0, 5.1 Hz, 1H), 3.55 (dd, J = 13.9, 8.6 Hz, 1H), 2.98 (dd, J = 13.9, 5.7 Hz, 1H), 2.08 (m, 6H), 1.87 (d, J = 1.3 Hz, 3H), 1.75 (ddd, J = 12.8, 9.9, 2.9 Hz, 1H), 1.57 (t, J = 1.2 Hz, 3H), 1.56 (s, 3H), 1.49 (dd, J = 10.2, 8.6 Hz, 1H), 1.16 (m, 3H), 1.14 (m, 1H), 1.09 (s, 3H), 0.86 ppm (dddd, J = 13.8, 12.6, 9.6, 2.9 Hz, 1H); ^{13}C NMR (151 MHz, $CDCl_3$): δ = 199.9, 143.2, 137.1, 136.6, 135.9, 124.4, 119.4, 39.9, 39.4, 39.0, 35.2, 29.0, 27.7, 26.3, 25.4, 23.9, 15.9, 15.6, 15.3, 11.6 ppm; IR (film) $\tilde{\nu}$ = 2923, 2853 1654, 1626, 1454, 1379, 1318, 1270, 1189, 1152, 1110, 1064, 1041, 1018, 870, 827, 801, 762, 748, 595, 523 cm^{-1}; HRMS (ESI): m/z calcd. for $C_{20}H_{30}O$ [M^+]: 287.23694; found: 287.23682.

	DEPRESSIN	SYNTHETIC 2		DEPRESSIN	SYNTHETIC 2
$[\alpha]_D$	**−80.0°, c = 0.26**	**−85.0°, c = 0.02**			
	**^{1}H NMR** δ (ppm, *J* [Hz])			**^{13}C NMR** δ [ppm]	
**1**	1.15	1.14 (m)	**1**	35.2	35.2
**2**	1.50 (dd, 10.2, 8.7)	1.49 (dd, 10.2, 8.6)	**2**	27.6	27.7
**3**	6.37 (d, 10.2)	6.38 (dq, 1.3, 10.2)	**3**	143.1	143.2
**4**	-	-	**4**	136.6	136.6
**5**	-	-	**5**	199.9	199.9
**6a**	3.55 (dd, 13.8, 5.7)	3.55 (dd, 13.9, 8.6)	**6**	39.4	39.4
**6b**	2.97 (dd, 13.8, 5.7)	2.98 (dd, 13.9, 5.7)	**7**	119.4	119.4
**7**	5.08 (t, 6.6)	5.07 (ddt, 8.7, 5.9, 1.4, 1.4)	**8**	137.1	137.1
**8**	-	-	**9**	39.0	39.0
**9a**	2.15	2.09 (m)	**10**	23.9	23.9
**9b**	2.00	2.00 (m)	**11**	124.4	124.4
**10a**	2.17	2.16 (m)	**12**	135.9	135.9
**10b**	1.96	1.98 (m)	**13**	39.9	39.9
**11**	4.84 (t, 5.4)	4.84 (dd, 9.0, 5.1)	**14**	26.3	26.3
**12**	-	-	**15**	25.4	25.4
**13a**	2.20	2.20 (d, 12.8)	**16**	29.0	29.0
**13b**	1.75	1.75 (ddd, 12.8, 9.9, 2.9)	**17**	15.8	15.9
**14a**	2.05	2.06 (m)	**18**	11.6	11.6
**14b**	0.80	0.86 (dddd, 13.8, 12.6, 9.6, 2.9)	**19**	15.6	15.6
**15**	-	-	**20**	15.3	15.3
**16**	1.16	1.16 (s)			
**17**	1.09	1.09 (s)			
**18**	1.87	1.87 (d, 1.3)			
**19**	1.56	1.57 (t, 1.2)			
**20**	1.56	1.56 (s)			

Compound 4 ("Nominal Euphorhylonal A"). MeLi (1.6 M in Et_2O, 16.1 µL, 25.8 µmol) was added dropwise to a solution of alkenyl stannane **36** (6.6 mg, 13.1 mmol) in THF (1.5 mL) at −78 °C. The mixture was stirred at −78 °C for 5 min and for additional 30 min at RT before it was cooled again to –78°C. DMF (9.1 µl, 117.1 mmol) was added dropwise at this temperature and stirring was continued for 20 min at −78 °C and for 1 h at RT. The reaction was quenched with a saturated aqueous NH_4Cl solution, and the aqueous layer was separated and extracted with *tert*-butyl methyl ether (3 × 5 mL). The combined organic phases were dried over $MgSO_4$ and concentrated, and the residue was purified by flash chromatography (hexane/EtOAc, 20:1) to yield the title compound as a colourless oil (2.4 mg, 68%).

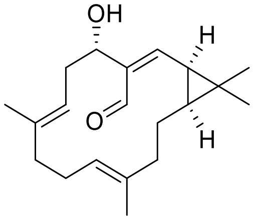

$[\alpha]_D^{20}$ = −37.7 (0.11 g/100 mL, $CHCl_3$); ^1H NMR (600 MHz, $CDCl_3$): δ = 10.13 (d, J = 2.0 Hz, 1H), 6.19 (d, J = 11.2 Hz, 1H), 4.90 (m, 2H), 3.95 (tdd, J = 10.9, 4.1, 2.0 Hz, 1H), 3.50 (d, J = 10.7 Hz, 1H), 2.70 (ddd, J = 13.7, 11.3, 9.2 Hz, 1H), 2.45 (dt, J = 12.9, 5.1 Hz, 1H), 2.33 (dt, J = 15.5, 5.6 Hz, 1H), 2.13 (m, 3H), 1.96 (dd, J = 11.2, 8.3 Hz, 1H), 1.91 (m, 2H), 1.87 (dddd, J = 14.5, 6.2, 4.7, 1.5 Hz, 1H), 1.62 (t, J = 0.9 Hz, 3H), 1.51 (d, J = 1.2 Hz, 3H), 1.28 (m, 1H), 1.16 (s, 3H), 1.03 (s, 3H), 1.02 ppm (td, J = 9.7, 8.3 Hz, 1H); ^{13}C NMR (151 MHz, $CDCl_3$): δ = 192.7, 151.3, 139.2, 136.6, 134.3, 123.5, 120.3, 77.5, 39.4, 38.9, 35.7, 35.6, 28.9, 26.8, 25.4, 24.1, 22.0, 17.7, 16.1, 15.9 ppm; IR (film) $\tilde{\nu}$ = 3436, 2921, 2853, 1734, 1657, 1620, 1452, 1377, 1260, 1091, 1017, 985, 798 cm^{-1}; HRMS (ESI): m/z calcd. for $C_{20}H_{30}O_2$ [M^++Na]: 325.21380; found: 325.21422.

Compound 5-*epi*-4. Prepared analogously as a colourless oil (2.1 mg, 53%). $[\alpha]_D^{20}$ = −77.4 (0.06 g/100 mL, $CHCl_3$); ^1H NMR (600 MHz, $CDCl_3$): δ = 10.18 (s, 1H), 6.49 (dd, J = 11.1, 1.2 Hz, 1H), 4.91 (m, 2H), 4.81 (tt, J = 5.2, 2.8 Hz, 1H), 2.55 (m, 1H), 2.45 (dt, 1H), 2.31 (dt, 1H), 2.12 (m, 3H), 2.07 (dd, J = 11.1, 8.3 Hz, 1H), 1.95 (m, 3H), 1.88 (d, J = 5.4 Hz, 1H), 1.63 (d, J = 1.3 Hz, 3H), 1.53 (d, J = 1.2 Hz, 3H), 1.22 (m, 1H), 1.17 (s, 3H), 1.08 (m, 1H), 1.04 ppm (s, 3H); ^{13}C NMR (151 MHz, $CDCl_3$): δ = 190.9, 148.1, 140.9, 137.7, 134.8, 124.6, 117.7, 67.8, 39.6, 38.8, 35.3, 33.0, 28.9, 26.2, 25.0, 24.0, 23.5, 17.2, 16.5, 15.7 ppm; IR (film) $\tilde{\nu}$ = 3429, 2919, 2862, 1662, 1654, 1448, 1377, 1377, 1150, 1125, 1097, 1057, 1020, 800, 672 cm^{-1}; HRMS (ESI): m/z calcd. for $C_{20}H_{30}O_2$ [M^++Na]: 325.21380; found: 325. 21437.

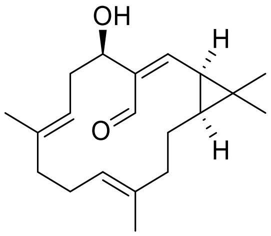

The reaction delivered a side product (ca. 10%) which was identified as aldehyde **S5** formed by protodestannation; analytical and spectral data of **S5**: $[\alpha]_D^{20}$ = −43.8 (0.21 g/100 mL, $CHCl_3$); ^1H NMR (400 MHz, $CDCl_3$): δ = 5.62 (ddd, J = 15.6, 3.5, 0.7 Hz, 1H), 5.41 (ddd, J = 15.5, 8.9, 1.8 Hz, 1H), 4.99 (m, 2H), 4.32 (s, 1H), 2.40 (m, 2H), 2.22 (dd, J = 13.7, 6.6 Hz, 1H), 2.13 (m, 3H), 2.00 (m, 1H), 1.91 (dt, J = 14.1, 7.2 Hz, 1H), 1.81 (dtd, J = 13.1, 6.5, 1.8 Hz, 1H), 1.64 (s, 3H), 1.60 (s, 3H), 1.43 (d, J = 7.2 Hz, 1H), 1.23 (m, 1H), 1.05 (s, 3H), 0.98 (s, 3H), 0.65 ppm (ddd, J =

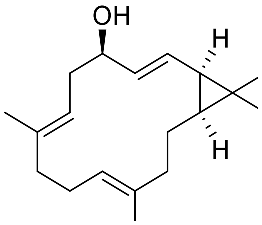

10.4, 8.7, 1.8 Hz, 1H); ^{13}C NMR (101 MHz, $CDCl_3$): δ = 136.9, 135.5, 132.5, 126.5, 124.0, 118.5, 71.0, 40.1, 38.8, 34.9, 30.9, 29.6, 28.8, 24.2, 24.0, 20.8, 17.0, 16.4, 15.8 ppm; IR (film) $\tilde{\nu}$ = 3367, 2923, 2857, 1719, 1666, 1453, 1376, 1260, 1071, 1019, 968, 872, 801, 735 cm^{-1}; HRMS (ESI): *m/z* calcd. for $C_{19}H_{30}O$ [M^++Na]: 297.21888; found: 297.21886.

Euphorhylonal A (*ent*-12). Prepared analogously as a colourless oil (2.7 mg, 61%). $[\alpha]_D^{20}$ = +77.3; $[\alpha]_D^{25}$ = +74.5 (0.11 g/100 mL, $CHCl_3$); ^{1}H NMR (600 MHz, $CDCl_3$): δ = 10.12 (d, *J* = 1.3 Hz, 1H), 5.98 (d, *J* = 11.4 Hz, 1H), 4.96 (t, *J* = 6.0 Hz, 1H), 4.86 (t, *J* = 6.9 Hz, 1H), 4.08 (td, *J* = 10.1, 9.6, 4.9 Hz, 1H), 3.55 (d, *J* = 9.9 Hz, 1H), 2.61 (m, 1H), 2.50 (m, 1H), 2.13 (m, 4H), 2.00 (m, 2H), 1.61 (m, 1H), 1.56 (s, 3H), 1.53 (s, 3H), 1.17 (s, 3H), 1.16 (s, 3H), 0.85 ppm (m, 1H); ^{13}C NMR (151 MHz, $CDCl_3$): δ = 192.1, 156.5, 137.2, 135.7, 132.6, 125.9, 120.8, 76.5, 39.1, 38.6, 37.7, 34.7, 29.1, 28.5, 24.2, 24.1, 22.9, 22.0, 15.9, 14.7 ppm; IR (film) $\tilde{\nu}$ = 3432, 2924, 2854, 1656, 1620, 1454, 1437, 1378, 1260, 1230, 1113, 1043, 1019, 965, 904 cm^{-1}. HRMS (ESI): *m/z* calcd. for $C_{20}H_{30}O_2$ [M^++Na]: 325.21380; found: 325.21378.

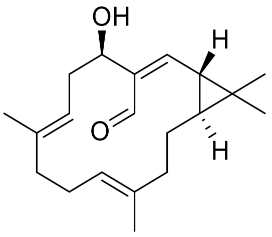

The reaction delivered a side product (< 10%) which was identified as aldehyde **S6** formed by protodestannation, which analyzed as follows: $[\alpha]_D^{20}$ = +52.5 (0.84 g/100 mL, $CHCl_3$); ^{1}H NMR (600 MHz, $CDCl_3$): δ = 5.38 (dd, *J* = 15.2, 8.3 Hz, 1H), 5.20 (dd, *J* = 15.2, 8.9 Hz, 1H), 5.06 (m, 1H), 5.00 (m, 1H), 4.16 (td, *J* = 8.9, 4.6 Hz, 1H), 2.45 (m, 1H), 2.13 (m, 7H), 1.92 (ddt, *J* = 14.6, 10.9, 3.8 Hz, 1H), 1.57 (s, 3H), 1.55 (s, 3H), 1.04 (s, 6H), 1.01 (m, 1H), 0.81 (dd, *J* = 8.9, 5.2 Hz, 1H), 0.37 ppm (ddd, *J* = 11.0, 5.1, 3.8 Hz, 1H); ^{13}C NMR (151 MHz, $CDCl_3$): δ = 135.5, 135.2, 133.0, 130.6, 125.7, 120.6, 74.1, 39.3, 38.4, 34.9, 32.7, 32.4, 24.5, 24.3, 22.8, 22.6, 21.7, 16.3, 14.8 ppm. IR (film) $\tilde{\nu}$ = 3370, 2956, 2922, 2871, 2855, 1664, 1455, 1377, 1292, 1252, 1182, 1151, 1075, 1023, 960, 878, 696, 675, 600, 519 cm^{-1}. HRMS (ESI): *m/z* calcd. for $C_{19}H_{30}O$ [M^++Na]: 297.21888; found: 297.21873.

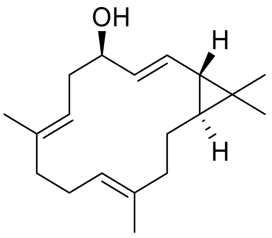

Compound 44. Prepared analogusly as a colourless oil (5.1 mg, 69%). $[\alpha]_D^{20}$ = +63.5; $[\alpha]_D^{25}$ = +98.7 °, (0.55 g/100 mL, $CHCl_3$); ^{1}H NMR (400 MHz, $CDCl_3$): δ = 10.17 (s, 1H), 6.23 (d, *J* = 11.4 Hz, 1H), 4.91 (td, *J* = 6.2, 3.2 Hz, 1H), 4.88 (m, 1H), 4.83 (dd, *J* = 7.5, 5.5 Hz, 1H), 2.47 (t, *J* = 7.5 Hz, 2H), 2.05 (m, 9H), 1.70 (dd, *J* = 11.4, 4.9 Hz, 1H), 1.57 (s, 3H), 1.52 (s, 3H), 1.18 (s, 3H), 1.17 (s, 3H), 0.82 ppm (ddd, *J* = 11.5, 5.0, 2.9 Hz, 1H); ^{13}C NMR (101 MHz, $CDCl_3$): δ = 190.9, 154.7, 138.3, 135.2, 132.9, 125.9, 120.6, 66.9, 38.8, 38.6, 37.8, 32.5, 29.2, 28.6, 24.3, 23.9, 22.9, 21.9, 15.4, 14.7 ppm. IR (film) $\tilde{\nu}$ = 3368, 2922, 2870, 2853, 1660, 1625, 1552, 1440, 1378, 1259, 1227,

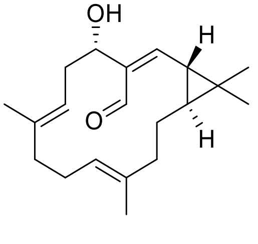

1141, 1065, 1031, 999, 963, 805, 688, 668 cm^{-1}. HRMS (ESI): *m/z* calcd. for $C_{20}H_{30}O_2$ [M^++Na]: 325.21380; found: 325.21354.

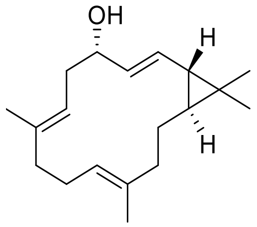

The reaction delivered a side product (< 10%) which was identified as aldehyde **S7** formed by protodestannation, which analysed as follows: $[\alpha]_D^{20}$ = −10.2 (0.42 g/100 mL, $CHCl_3$); ^{1}H NMR (600 MHz, $CDCl_3$): δ = 5.59 (ddd, *J* = 15.7, 4.7, 0.9 Hz, 1H), 5.48 (ddd, *J* = 15.7, 7.3, 1.4 Hz, 1H), 5.06 (m, 1H), 4.98 (m, 1H), 4.26 (m, 1H), 2.36 (m, 2H), 2.26 (dt, *J* = 11.9, 7.4 Hz, 1H), 2.12 (m, 4H), 2.03 (td, *J* = 12.5, 3.4 Hz, 1H), 1.92 (ddt, *J* = 15.0, 11.9, 3.2 Hz, 1H), 1.57 (s, 3H), 1.53 (s, 3H), 1.05 (s, 3H), 1.01 (s, 3H), 0.96 (m, 1H), 0.78 (dd, *J* = 7.3, 5.4 Hz, 1H), 0.41 ppm (ddd, *J* = 11.4, 5.3, 3.2 Hz, 1H); ^{13}C NMR (151 MHz, $CDCl_3$): δ = 135.4, 133.6, 131.7, 130.4, 125.6, 121.1, 70.6, 39.1, 38.6, 35.2, 33.0, 31.5, 24.8, 24.3, 22.9, 22.4, 21.7, 15.5, 14.8 ppm. IR (film) $\tilde{\nu}$ = 3345, 2919, 2850, 1729, 1668, 1453, 1377, 1287, 1258, 1105, 1083, 1018, 962, 881, 835 cm^{-1}. HRMS (ESI): *m/z* calcd. for $C_{19}H_{30}O$ [M^++Na]: 297.218884; found: 297.218930.

Yuexiandajisu A (*ent*-14). MeLi (1.6 M in Et_2O, 7.6 µL, 12.1 µmol) was added dropwise to a solution of alkenyl stannane **42** (3.1 mg, 5.5 µmol) in THF (1.6 mL) at −78 °C. The mixture was stirred at −78 °C for 10 min and for additional 15 min at RT before it was cooled again to −78°C. CO_2 was bubbled through the mixture for 5 min at −78 °C and for 30 min at RT. The reaction was quenched with a saturated aqueous NH_4Cl solution, and the aqueous layer was separated and extracted with *tert*-butyl methyl ether (3 × 5 mL). The combined organic phases were dried over $MgSO_4$ and concentrated, and the residue was purified by flash chromatography (hexane/EtOAc, 10:1 → EtOAc), followed by preparative HPLC (Eclipse Plus C18, 50 mm × 1.8 µm, ∅ 4.6 mm, methanol / 0.1% TFA in water = 80:20, 1.0 mL/min, 20.1 MPa, 308 K, UV, 254 nm) to yield the title compound as a colourless amorphous solid (0.9 mg, 51%). $[\alpha]_D^{30}$ = +171.3 (0.08 g/100 mL, EtOH); ^{1}H NMR (600 MHz, $CDCl_3$): δ = 5.71 (d, *J* = 11.0 Hz, 1H), 5.01 (d, *J* = 6.8 Hz, 1H), 4.87 (m, 1H), 4.16 (dd, *J* = 11.0, 5.2 Hz, 1H), 2.64 (m, 1H), 2.49 (m, 1H), 2.08 (m, 6H), 2.04 (dd, *J* = 11.1, 5.1 Hz, 1H), 1.95 (ddt, *J* = 15.1, 11.5, 3.8 Hz, 1H), 1.58 (s, 3H), 1.56 (s, 3H), 1.16 (m, 1H), 1.14 (s, 3H), 1.12 (s, 3H), 0.73 ppm (dt, *J* = 11.1, 4.5 Hz, 1H); ^{13}C NMR (151 MHz, $CDCl_3$): δ = 170.5, 153.2, 136.1, 133.0, 126.8, 125.4, 120.4, 78.1, 39.1, 38.6, 37.1, 34.6, 31.8, 27.9, 24.10, 24.12, 22.9, 22.0, 16.2, 14.8 ppm; IR (film) $\tilde{\nu}$ = 3401, 2923, 2853, 1675, 1437, 1408, 1377, 1263, 1244, 1190, 1142, 1114, 1029, 880, 804, 747, 601, 491 cm^{-1}; HRMS (ESI): *m/z* calcd. for $C_{20}H_{30}O_3$ [M^+]: 317.21222; found: 317.21231.

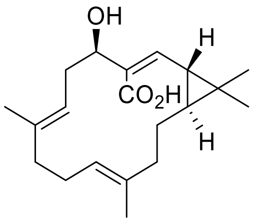

Comparison of the analytical and spectral data of Yuexiandajisu A as reported in the literature with those of synthetic *ent*-14

	**Yuexiandajisu A**		**synthetic *ent*-14**		**Yuexiandajisu A**		**synthetic *ent*-14**
$[\alpha]_D^{30}$	**+172°, c = 0.78**		**+171.3°, c = 0.04**				
	**^{1}H NMR** δ (ppm, *J* [Hz])				**^{13}C NMR** δ [ppm]		
	original	*reassigned*			*original*	*reassigned*	
**1**	0.75 (m)	0.75 (m)	0.73 (m)	**1**	37.3	37.3	37.1
**2**	2.06 (m)	2.06 (m)	2.04 (dd, *J* = 11.1, 5.1)	**2**	31.9	31.9	31.8
**3**	5.74 (d, 11.1)	5.74 (d, 11.1)	5.71 (d, 11.0)	**3**	153.5	153.5	153.2
**4**	-	-	-	**4**	127.0	127.0	126.8
**5**	4.15 (dd, 11.0, 5.3)	4.15 (dd, 11.0, 5.3)	4.16 (dd, 11.0, 5.2)	**5**	78.0	78.0	78.1
**6a**	2.48 (m)	2.48 (m)	2.49 (m)	**6**	34.7	34.7	34.6
**6b**	2.67 (m)	2.67 (m)	2.64 (m)	**7**	120.6	120.6	120.4
**7**	4.88 (dd, 7.2, 5.2)	4.88 (dd, 7.2, 5.2)	4.87 (m, 1H)	**8**	136.0	136.0	136.1
**8**	-	-	-	**9**	38.6	38.6	38.6
**9(a)**	2.04 (m)	2.04 (m)	2.02 (m)	**10**	39.1	24.1	24.1
**9b**	-	-	2.09 (m)	**11**	125.5	125.5	125.4
**10(a)**	2.12 (m)	2.12 (m)	2.13 (m)	**12**	132.9	132.9	133.0
**10b**	-	-		**13**	24.1	39.1	39.1
**11**	5.02 (t, br)	5.02 (t, br)	5.01 (d, 6.8)	**14**	24.1	24.1	24.1
**12**	-	-	-	**15**	27.9	27.9	27.9
**13a**	2.14 (m)	2.14 (m)	2.10 (m)	**16**	22.0	22.9	22.9
**13b**	-	-	2.17 (m)	**17**	22.9	22.0	22.0
**14a**	1.19 (m)	1.19 (m)	1.16 (m)	**18**	-	-	-
**14b**	1.95 (m)	1.95 (m)	1.95 (ddt, 15.1, 11.5, 3.8)	**19**	16.2	16.2	16.2
**15**	-	-	-	**20**	14.8	14.8	14.8
**16**	1.15 (s)	1.19 (s)	1.14 (s)	**21**	171.8	171.8	170.5
**17**	1.19 (s)	1.15 (s)	1.12 (s)				
**18**	-	-	-				
**19**	1.57 (s)	1.58 (s)	1.58 (s)				
**20**	1.58 (s)	1.57 (s)	1.56 (s)				

Comparison of the analytical and ^{1}H NMR data (δ, ppm; *J* [Hz]) of Euphorhylonal A and Pekinenin C with those of various synthetic samples

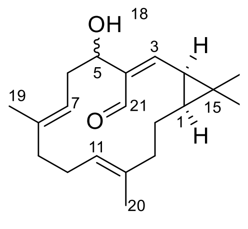

euphorhylonal A

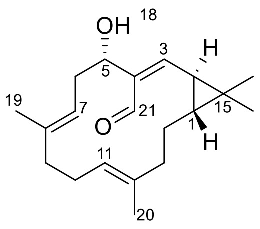

pekinenin C

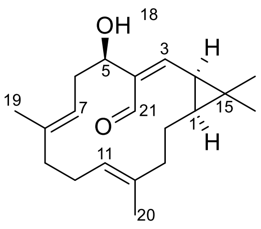

5-*epi*-4

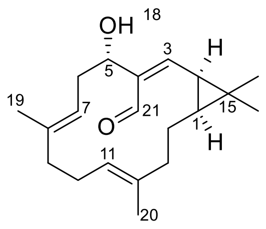

4

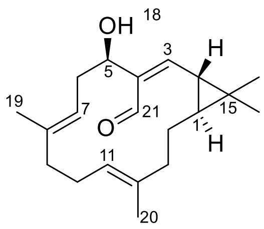

***ent*-12**

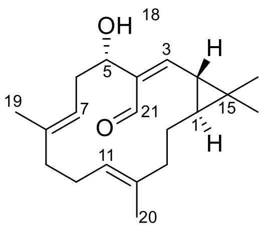

44

$[\alpha]_D$	**+90.5°, c = 0.3**	**−19°, c = 0.04**	**−77.4°, c = 0.28**	**−37,7°, c = 0.11**	**+77.3°, c = 0.11**	**+98.7°, c = 0.55**
**1**	0.84 (m)	0.82 (m)	1.08 (m)	1.02 (td, 9.7, 8.3)	0.82 (m)	0.82 (m)
**2**	1.61 (dd, 11.4, 4.9)	1.61 (m)	2.07 (dd, 11.1, 8.3)	1.96 (dd, 11.2, 8.3)	1.60 (dd, 11.4, 5.0)	1.70 (dd, 11.4, 4.9)
**3**	5.99 (d, 11.4)	5.97 (d, 11.5)	6.49 (dd, 11.1, 1.2)	6.19 (d, 11.2)	5.98 (d, 11.4)	6.23 (d, 11.4)
**4**	-	-	-	-	-	-
**5**	4.07 (dd, 6.5, 1)	4.08 (dd, 10.0, 4.5)	4.81 (m)	3.95 (tdd, 10.9, 4.1, 2.0)	4.08 (td)	4.83 (dd, 7.5, 5.5)
**6(a)**	2.51 (m)	2.49 (m)	2.46 (m)	2.45 (dt, 13.7)	2.49 (dt, 14.4, 5.2)	2.47 (t, 7.5)
**6b**	2.62 (m)	2.61 (m)	2.56 (m)	2.70 (ddd, 13.7, 11.3, 9.2)	2.61 (ddd, 14.3, 10.4, 8.7)	-
**7**	4.86 (t, 7.0)	4.86 (t, 7.0)	4.91 (m)	4.89 (m)	4.86 (dd, 7.7, 5.7)	4.92 (m)
**8**	-	-	-	-	-	-
**9(a)**	2.00 (m)	2.04 (m)	1.99 (m)	1.91 (m)	2.01 (m)	2.09 (m)
**9b**		2.09 (m)	2.11 (m)	2.14 (m)	2.11 (m)	2.11 (m)
**10(a)**	2.14 (m)	2.14 (m)	2.11 (m)	2.14 (m)	2.14 (m)	2.23 (m)
**10b**		-		2.09 (m)	-	1.98 (m)
**11**	4.96 (t, 6.6)	4.97 (t, 6.0)	4.92 (m)	4.91 (m)	4.96 (t, 6.0, 6.0)	4.88 (m)
**12**	-	-	-	-	-	-
**13(a)**	2.00 (m)	2.15 (m)	2.30 (m)	2.33 (dt, 15.5, 5.6)	2.15 (m)	2.18 (m)
**13b**		2.01 (m)	1.91 (m)	1.95 (m)	2.00 (m)	1.96 (m)
**14(a)**	2.14 (m)	1.13 (m)	1.23 (m)	1.28 (m)	1.14 (m)	1.09 (m)

**14b**		1.99 (m)	1.94 (m)	1.87 (dddd, 14.5, 6.2, 4.7, 1.5)	2.00 (m)	1.98 (m)
**15**	-	-	-	-	-	-
**16**	1.17 (s)	1.17 (s)	1.17 (s)	1.16 (s)	1.17 (s)	1.17 (s)
**17**	1.16 (s)	1.16 (s)	1.04 (s)	1.03 (s)	1.16 (s)	1.18 (s)
**18**	-	-	1.86 (s)	3.50 (d, 10.7)	3.55 (d, 9.9)	-
**19**	1.54 (s)	1.53 (s)	1.53 (s)	1.51 (d, 1.2)	1.53 (s)	1.53 (s)
**20**	1.57 (s)	1.57 (s)	1.63 (s)	1.62 (t, 0.9)	1.56 (s)	1.57 (s)
**21**	10.12 (d, 1.4)	10.11 (s)	10.18 (s)	10.13 (d, 2.0)	10.12 (d, 1.4)	10.17 (s)

Comparison of the ^{13}C NMR data (δ, ppm) of Euphorylonal A and Pekinenin C as reported in the Literature with those of various synthetic compounds

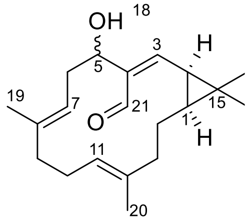

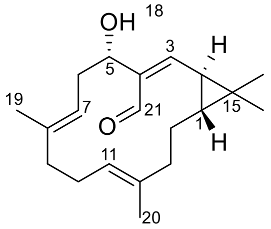

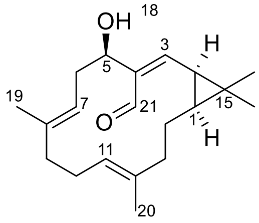

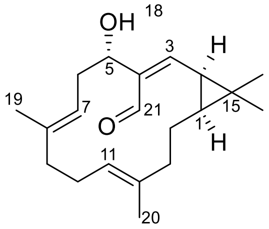

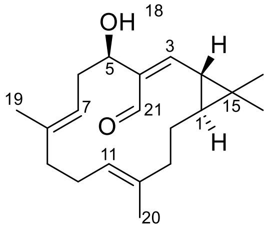

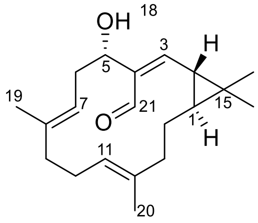

Nr	**euphorhylonal A**		**pekinenin C**	**5-*epi*-4**	**4**	***ent*-12**	**44**
	original	*reassigned*					
**1**	37.7	37.7	37.7	35.3	35.6	37.7	37.8
**2**	29.1	29.1	29.1	25.0	25.4	29.1	29.2
**3**	156.4	156.4	156.4	148.1	151.3	156.5	154.7
**4**	132.6	137.3	137.3	140.9	139.2	137.2	138.3
**5**	76.4	76.4	76.4	67.8	77.5	76.5	66.9
**6**	34.7	34.7	34.7	33.0	35.7	34.7	32.5
**7**	120.8	120.8	120.8	117.7	120.3	120.8	120.6
**8**	137.3	135.6	135.6	137.7	136.6	135.7	135.2
**9**	39.2	39.2	38.6	38.8	38.9	38.6	38.6
**10**	24.1	24.1	24.1	24.0	24.1	24.1	23.9
**11**	125.9	125.9	125.9	124.6	123.5	125.9	125.9
**12**	135.6	132.6	132.5	134.8	134.3	132.6	132.9
**13**	38.6	38.6	39.1	39.6	39.4	39.1	38.8
**14**	24.2	24.2	24.2	23.5	22.0	24.2	24.4
**15**	28.4	28.4	28.4	26.2	26.8	28.5	28.6
**16**	22.8	22.8	22.8	28.9	28.9	22.9	22.9
**17**	21.9	21.9	21.9	15.7	15.9	22.0	21.9
**18**	-	-	-	-	-	-	-
**19**	15.9	15.9	15.9	16.5	16.1	15.9	15.4
**20**	14.7	14.7	14.7	17.2	17.7	14.7	14.7
**21**	192.1	192.1	192.1	190.9	192.7	192.1	190.9

Mosher Ester Analyses

Mosher Ester S8. (*R*)-(−)-α-Methoxy-α-(trifluormethyl)phenylacetyl chloride (1.0 µL, 4.7 µmol) was added to a solution of DMAP (0.1 mg, 1.0 µmol), Et_3N (2.0 µL, 14.3 µmol) and propargylic alcohol **34** (1.3 mg, 4.7 µmol) in CH_2Cl_2 (0.2 mL). The mixture was stirred at RT for 1 h before it was diluted with Et_2O (1 mL) and washed with aqueous saturated NH_4Cl solution. The aqueous layer was separated and extracted with *tert*-butyl methyl ether (3 × 2 mL). The combined organic phases were dried over $MgSO_4$ and concentrated, and the residue was purified by flash chromatography (hexane/EtOAc, 20:1) to yield the title compound as a colourless oil (2.3 mg, 99%). $[\alpha]_D^{20}$ = +11.1 (0.18 g/100 mL, $CHCl_3$); ^{1}H NMR (600 MHz, $CDCl_3$): δ = 7.56 (m, 2H), 7.39 (m, 3H), 5.59 (ddd, *J* = 9.5, 4.1, 1.8 Hz, 1H), 5.05 (dddt, *J* = 7.9, 6.7, 5.4, 1.2 Hz, 2H), 3.57 (q, *J* = 1.1 Hz, 3H), 2.52 (m, 1H), 2.46 (ddd, *J* = 14.2, 9.5, 6.7 Hz, 1H), 2.15 (m, 2H), 2.01 (m, 4H), 1.81 (dddd, *J* = 13.6, 11.3, 6.6, 2.5 Hz, 1H), 1.59 (d, *J* = 1.4 Hz, 3H), 1.58 (d, *J* = 1.2 Hz, 3H), 1.18 (dd, *J* = 8.3, 1.9 Hz, 1H), 1.05 (m, 4H), 0.94 (s, 3H), 0.74 ppm (ddd, *J* = 10.9, 8.3, 2.5 Hz, 1H); ^{13}C NMR (151 MHz, $CDCl_3$): δ = 165.5, 137.8, 135.8, 132.1, 129.5, 128.3, 127.5, 123.9, 123.2 (q), 118.6, 86.6, 76.0, 67.1, 55.5, 39.6, 39.6, 33.4, 30.9, 27.4, 26.1, 23.8, 22.5, 17.6, 16.0, 15.8, 15.6 ppm ($\underline{C}_{q,\ sp3}$ signal is missing); IR (film) $\tilde{\nu}$ = 2946, 2927, 2853, 1750, 1452, 1268, 1251, 1170, 1122, 1081, 1016, 991, 968, 719, 697 cm^{-1}; HRMS (ESI): *m/z* calcd. for $C_{29}H_{35}F_3O_3$ [M^++Na]: 511.24305; found: 511.24310.

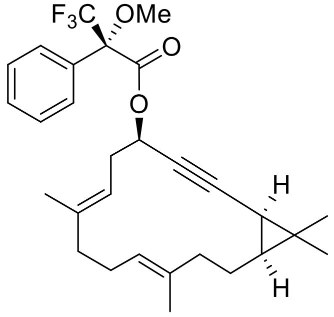

Mosher Ester S9. Prepared analogously using (*S*)-(+)-α-methoxy-α-(trifluormethyl)phenylacetyl chloride and compound **34**; colourless oil (2.1 mg, 78%). $[\alpha]_D^{20}$ = +94.1 (0.17 g/100 mL, $CHCl_3$); ^{1}H NMR (600 MHz, $CDCl_3$): δ = 7.57 (m, 2H), 7.40 (m, 3H), 5.59 (ddd, *J* = 9.6, 4.1, 1.8 Hz, 1H), 5.07 (m, 1H), 5.02 (tq, *J* = 6.5, 0.9 Hz, 1H), 3.60 (m, 3H), 2.45 (m, 1H), 2.38 (m, 1H), 2.17 (m, 1H), 2.11 (m, 1H), 2.02 (m, 4H), 1.84 (dddd, *J* = 13.5, 11.1, 6.5, 2.5 Hz, 1H), 1.60 (d, *J* = 1.4 Hz, 3H), 1.57 (q, *J* = 0.9 Hz, 3H), 1.21 (dd, *J* = 8.3, 1.8 Hz, 1H), 1.06 (m, 1H), 1.06 (s, 3H), 1.03 (s, 3H), 0.77 ppm (ddd, *J* = 10.9, 8.3, 2.5 Hz, 1H); ^{13}C NMR (151 MHz, $CDCl_3$): δ = 165.6, 137.9, 135.8, 132.4, 129.5, 128.3, 127.4, 123.9, 123.3 (q), 118.5, 86.6, 84.4 (q), 76.1, 66.9, 55.4, 39.6, 39.6, 33.3, 30.9, 27.4, 26.2, 23.8, 22.5, 17.7, 16.1, 15.8, 15.5 ppm; IR (film) $\tilde{\nu}$ = 2980, 2945, 2928, 2863, 1750, 1452, 1268, 1248, 1185, 1169, 1122, 1016, 991, 920, 717, 698 cm^{-1}; HRMS (ESI): *m/z* calcd. for $C_{29}H_{35}F_3O_3$ [M^++Na]: 511.24305; found: 511.24322.

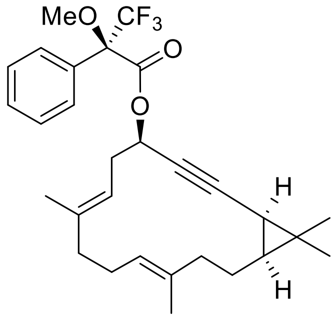

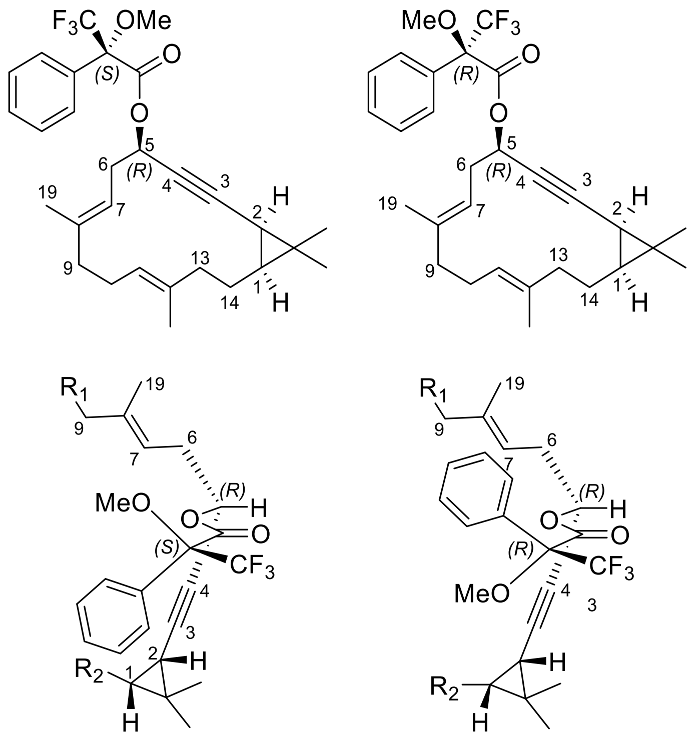

Position	δ_S (^{1}H, ppm)	δ_R (^{1}H, ppm)	$\Delta\delta^{SR} = \delta_S - \delta_R$
14a	1.81	1.84	−0.03
14b	1.03	1.07	−0.04
1	0.74	0.77	−0.03
2	1.18	1.21	−0.03
3	-	-	-
4	-	-	-
5	5.59	5.59	±0.00
6a	2.52	2.45	+0.07
6b	2.46	2.38	+0.08
7	5.04	5.02	+0.02
19	1.58	1.57	+0.01
9a	2.12	2.11	+0.01
9b	2.02	2.00	+0.02

Mosher Ester S10. Prepared analogously from compound **35** and (*R*)-(−)-α-methoxy-α-(trifluormethyl)phenylacetyl chloride (1.0 µL, 5.5 µmol) as a colourless oil (1.4 mg, 72%). $[\alpha]_D^{20}$ = −172.0 (0.05 g/100 mL, $CHCl_3$); ^1H NMR (600 MHz, $CDCl_3$): δ = 7.58 (m, 2H), 7.39 (m, 3H), 5.71 (ddd, *J* = 8.1, 3.8, 1.9 Hz, 1H), 5.14 (ddq, *J* = 8.6, 6.1, 1.3 Hz, 1H), 5.07 (ddq, *J* = 8.5, 5.9, 1.1 Hz, 1H), 3.61 (q, *J* = 1.2 Hz, 3H), 2.52 (dt, *J* = 14.2, 8.3 Hz, 1H), 2.33 (dm, *J* = 14.2 Hz, 1H), 2.11 (m, 3H), 1.98 (m, 3H), 1.81 (dddd, *J* = 13.8, 9.6, 6.0, 2.4 Hz, 1H), 1.62 (d, *J* = 1.2 Hz, 3H), 1.45 (t, *J* = 1.1 Hz, 3H), 1.19 (dd, *J* = 8.2, 1.9 Hz, 1H), 1.18 (m, 1H), 1.05 (s, 3H), 1.02 (s, 3H), 0.74 ppm (ddd, *J* = 10.5, 8.2, 2.4 Hz, 1H); ^{13}C NMR (151 MHz, $CDCl_3$): δ = 165.8, 138.0, 135.3, 132.7, 129.5, 128.3, 127.4, 124.1, 123.3 (q), 118.4, 86.8, 84.3 (q), 76.2, 66.8, 55.5, 39.6, 39.4, 32.9, 30.9, 27.4, 25.6, 24.0, 22.6, 18.0, 16.4, 16.2, 15.7 ppm; IR (film) $\tilde{\nu}$ = 2982, 2923, 2853, 1750, 1452, 1269, 1237, 1184, 1170, 1123, 1018, 992, 917, 844, 716, 697 cm^{-1}; HRMS (ESI): *m/z* calcd. for $C_{29}H_{35}F_3O_3$ [M^++Na]: 511.24305; found: 511.24320.

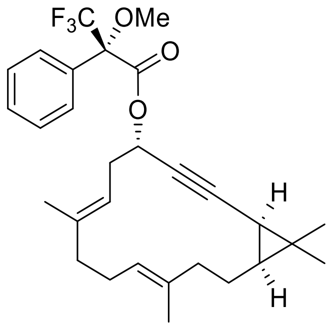

Mosher Ester S11. Prepared analogously from compound **35** and (*S*)-(+)-α-methoxy-α-(trifluormethyl)phenylacetyl chloride (1.0 µL, 5.5 µmol) as a colourless oil (1.3 mg, 72%). $[\alpha]_D^{20}$ = +15.0 (0.04 g/100 mL, $CHCl_3$); ^1H NMR (600 MHz, $CDCl_3$): δ = 7.55 (m, 2H), 7.39 (m, 3H), 5.65 (ddd, *J* = 8.4, 3.9, 1.9 Hz, 1H), 5.08 (m, 1H), 4.96 (t(m), *J* = 7.0 Hz, 1H), 3.57 (m, 3H), 2.61 (dt, *J* = 14.1, 8.4 Hz, 1H), 2.39 (dt, *J* = 14.2, 5.0 Hz, 1H), 2.11 (m, 3H), 2.08 (m, 1H), 1.99 (m, 1H), 1.90 (ddd, *J* = 14.3, 9.3, 5.8 Hz, 1H), 1.77 (dddd, *J* =13.7, 9.4, 6.0, 2.3 Hz, 1H), 1.59 (d, *J* = 1.3 Hz, 3H), 1.57 (t, *J* = 1.0 Hz, 3H), 1.16 (dd, *J* = 8.3, 1.9 Hz, 1H), 1.15 (m, 1H), 1.05 (s, 3H), 1.02 (s, 3H), 0.71 ppm (ddd, *J* = 10.5, 8.3, 2.3 Hz, 1H); ^{13}C NMR (151 MHz, $CDCl_3$): δ = 165.6, 138.1, 135.2, 132.2, 129.5, 128.3, 127.4, 123.9, 123.3 (q), 118.4, 86.6, 84.6 (q), 76.0, 67.0, 55.5, 39.6, 39.4, 33.0, 30.9, 27.4, 25.4, 24.0, 22.6, 18.1, 16.4, 16.2, 15.9 ppm; IR (film) $\tilde{\nu}$ = 2981, 2924, 2854, 1749, 1452, 1259, 1185, 1168, 1119, 1101, 1082, 1015, 992, 798, 717, 696 cm^{-1}; HRMS (ESI): *m/z* calcd. for $C_{29}H_{35}F_3O_3$ [M^++Na]: 511.24305; found: 511.24328.

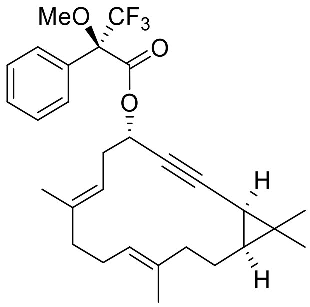

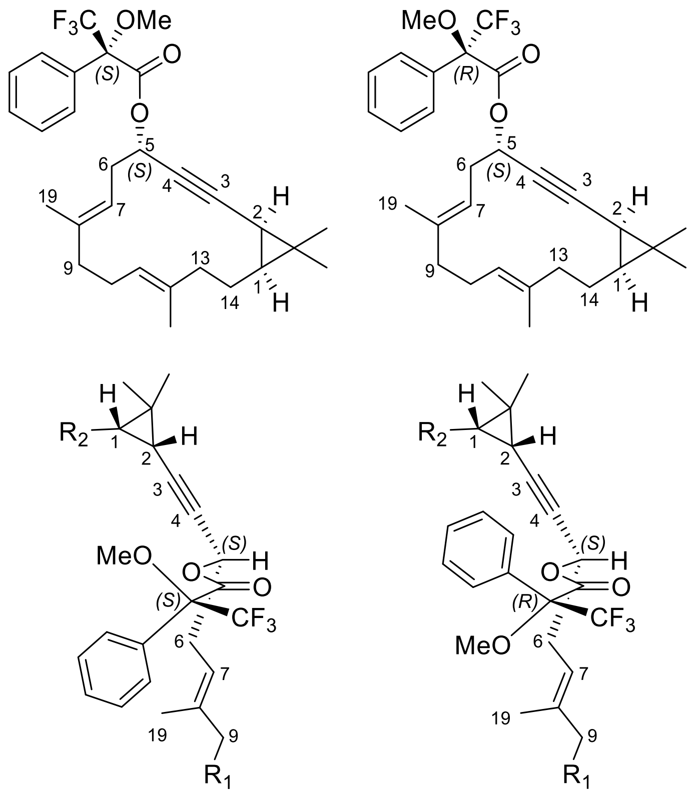

Position	δ_S (¹H, ppm)	δ_R (¹H, ppm)	$\Delta\delta^{SR} = \delta_S - \delta_R$
14a	1.81	1.77	+0.04
14b	1.18	1.15	+0.03
1	0.74	0.71	+0.03
2	1.19	1.16	+0.03
3	-	-	-
4	-	-	-
5	5.71	5.65	+0.06
6a	2.52	2.61	−0.09
6b	2.33	2.39	−0.06
7	5.07	5.08	−0.01
19	1.45	1.57	−0.12
9a	2.09	2.11	−0.02
9b	1.96	1.97	−0.01

Mosher Ester S12. Prepared analogously from side product **S5** and (*R*)-(−)-α-methoxy-α-(trifluormethyl)phenylacetyl chloride (1.7 µL, 2.4 mg, 8.2 µmol) as a colourless oil (2.4 mg, 4.9 µmol, 90%). $[\alpha]_D^{20}$ = −96.0 (0.05 g/100 mL, $CHCl_3$); ^{1}H NMR (600 MHz, $CDCl_3$): δ = 7.55 (dd, *J* = 7.3, 2.6 Hz, 2H), 7.39 (m, 3H), 5.62 (m, 1H), 5.49 (dd, *J* = 15.7, 4.0 Hz, 1H), 5.38 (ddd, *J* = 15.6, 8.8, 1.5 Hz, 1H), 5.00 (m, 1H), 4.94 (t, *J* = 6.9 Hz, 1H), 3.57 (d, *J* = 1.2 Hz, 3H), 2.50 (m, 2H), 2.18 (m, 3H), 2.06 (m, 1H), 1.96 (t, *J* = 11.5 Hz, 1H), 1.88 (dt, *J* = 14.1, 6.9 Hz, 1H), 1.76 (dtd, *J* = 13.9, 6.9, 1.9 Hz, 1H), 1.59 (s, 3H), 1.58 (s, 3H), 1.19 (t, *J* = 8.9 Hz, 1H), 1.01 (s, 3H), 0.93 (m, 1H), 0.83 (s, 3H), 0.63 ppm (ddd, *J* = 10.5, 8.7, 1.9 Hz, 1H); ^{13}C NMR (151 MHz, $CDCl_3$): δ = 165.6, 137.1, 135.4, 132.5, 129.8, 129.5, 128.3, 127.4, 126.0, 123.4, 118.4, 75.9, 55.4, 39.9, 39.1, 31.7, 31.2, 29.6, 28.7, 24.1, 23.6, 21.2, 16.7, 16.0, 15.6 ppm ($\underline{C}F_3$ and $\underline{C}_{q,\,sp3}$ signals are missing); IR (film) $\tilde{\nu}$ = 2952, 2918, 2850, 1745, 1452, 1383, 1261, 1185, 1169, 1121, 1106, 1081, 1019, 991, 965, 799, 717, 672 cm^{-1}; HRMS (ESI): *m*/*z* calcd. for $C_{29}H_{37}F_3O_3$ [M^++Na]: 513.25870; found: 513.25861.

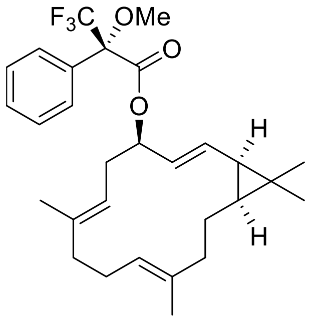

Mosher Ester S13. Prepared analogously from side product **S5** and (*S*)-(+)-α-methoxy-α-(trifluormethyl)phenylacetyl chloride (1.7 µL, 2.4 mg, 8.2 µmol) as a colourless oil (2.1 mg, 78%) $[\alpha]_D^{20}$ = −18.1 (0.52 g/@ mL, $CHCl_3$); ^{1}H NMR (600 MHz, $CDCl_3$): δ = 7.54 (m, 2H), 7.39 (m, 3H), 5.60 (dd, *J* = 7.7, 3.7 Hz, 1H), 5.56 (dd, *J* = 15.4, 4.0 Hz, 1H), 5.49 (ddd, *J* = 15.4, 8.6, 1.1 Hz, 1H), 4.93 (m, 2H), 3.56 (d, *J* = 1.2 Hz, 3H), 2.47 (q, *J* = 7.3 Hz, 1H), 2.41 (ddd, *J* = 14.8, 7.9, 3.1 Hz, 1H), 2.20 (dd, *J* = 14.2, 6.9 Hz, 1H), 2.12 (m, 2H), 2.06 (m, 1H), 1.93 (t, *J* = 10.4 Hz, 1H), 1.88 (dt, *J* = 14.0, 6.9 Hz, 1H), 1.79 (dtd, *J* = 13.9, 7.0, 2.0 Hz, 1H), 1.59 (s, 3H), 1.57 (s, 3H), 1.23 (t, *J* = 8.7 Hz, 1H), 1.04 (s, 3H), 0.95 (td, *J* = 7.1, 3.4 Hz, 1H), 0.91 (s, 3H), 0.66 ppm (ddd, *J* = 10.7, 8.7, 1.9 Hz, 1H); ^{13}C NMR (151 MHz, $CDCl_3$): δ = 165.7, 137.0, 135.4, 132.4, 130.3, 129.5, 128.4, 127.4, 126.0, 123.4, 118.4, 76.0, 55.3, 39.9, 39.1, 31.4, 31.2, 29.7, 29.6, 28.7, 24.1, 23.8, 16.7, 15.9, 15.8 ppm ($\underline{C}F_3$ and $\underline{C}_{q,\,sp3}$ signals are missing); IR (film) $\tilde{\nu}$ = 2962, 2916, 2850, 1749, 1446, 1412, 1258, 1078, 1010, 684, 789, 700, 662, 466 cm^{-1}; HRMS (ESI): *m*/*z* calcd. for $C_{29}H_{37}F_3O_3$ [M^++Na]: 513.25870; found: 513.25910.

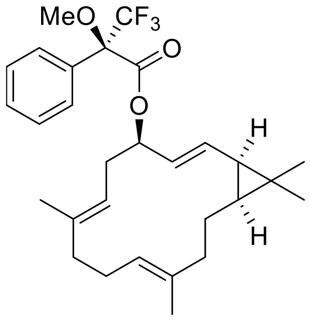

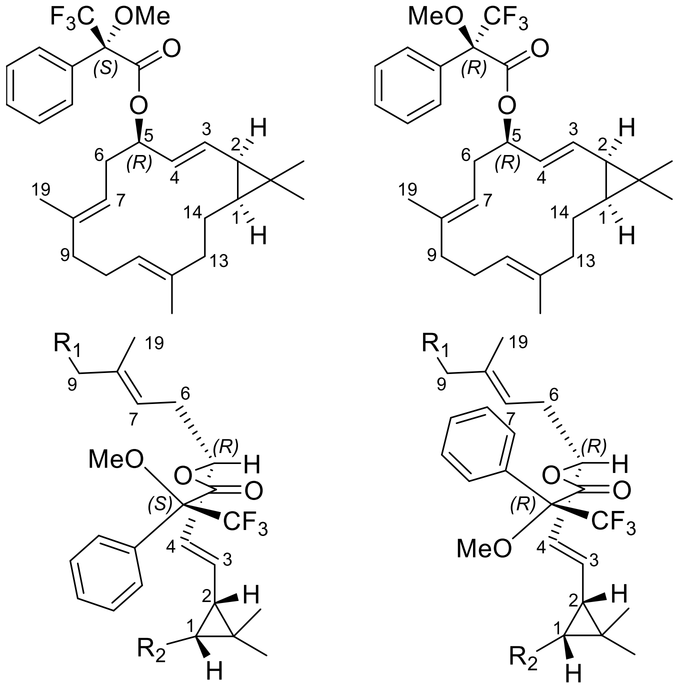

Positio n	δ_S (^{1}H, ppm)	δ_R (^{1}H, ppm)	$\Delta\delta^{SR} = \delta_S - \delta_R$
14a	0.93	0.95	−0.02
14b	1.76	1.79	−0.03
1	0.63	0.66	−0.03
2	1.19	1.23	−0.04
3	5.38	5.49	−0.11
4	5.49	5.56	−0.07
5	5.62	5.60	+0.02
6a	2.51	2.47	+0.04
6b	2.50	2.41	+0.09
7	4.99	4.93	+0.06
19	1.58	1.57	+0.01
9a	1.96	1.93	+0.03
9b	2.13	2.12	+0.01

Mosher Ester S14. Prepared analogously from compound **40** and (*R*)-(−)-α-methoxy-α-(trifluormethyl)phenylacetyl chloride as a colourless oil (2.6 mg, 97%). $[\alpha]_D^{20}$ = −57.6 (0.25 g/100 mL, CHCl₃); ¹H NMR (600 MHz, CDCl₃): δ = 7.56 (m, 2H), 7.40 (m, 3H), 5.59 (ddd, *J* = 6.7, 4.3, 1.2 Hz, 1H), 5.18 (td, *J* = 7.3, 6.9, 1.4 Hz, 1H), 5.11 (m, 1H), 3.57 (d, *J* = 1.1 Hz, 3H), 2.50 (m, 2H), 2.21 (m, 1H), 2.17 (m, 1H), 2.14 (m, 3H), 2.07 (td, *J* = 13.1, 12.1, 3.2 Hz, 1H), 1.85 (ddt, *J* = 14.1, 10.9, 3.1 Hz, 1H), 1.57 (s, 3H), 1.55 (s, 3H), 1.12 (s, 3H), 1.03 (s, 3H), 0.93 (m, 1H), 0.64 (m, 1H), 0.61 ppm (dd, *J* = 5.3, 1.2 Hz, 1H); ¹³C NMR (151 MHz, CDCl₃): δ = 165.6, 138.0, 133.1, 132.2, 129.5, 128.3, 127.6, 126.1, 118.1, 89.9, 73.1, 67.2, 55.5, 39.1, 38.7, 34.3, 33.1, 24.7, 24.5, 23.4, 23.4, 20.4, 19.2, 15.7, 15.1 ppm (CF₃ and $C_{q,\ sp3}$ signals are missing); IR (film) $\tilde{\nu}$ = 2972, 2923, 2852, 2239, 1750, 1497, 1451, 1379, 1270, 1250, 1185, 1169, 1122, 1081, 1017, 991, 965, 920, 882, 831, 764, 718, 696 cm^{-1}; HRMS (ESI): *m/z* calcd. for $C_{29}H_{35}F_3O_3$ [M^++Na]: 511.24305; found: 511.24336.

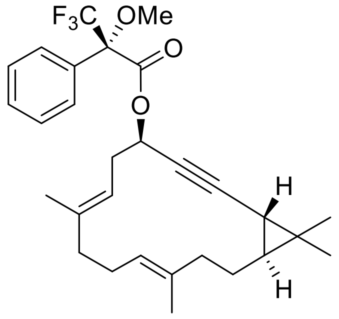

Mosher Ester S15. Prepared analogously from compound **40** and (*S*)-(+)-α-methoxy-α-(trifluormethyl)phenylacetyl chloride as a colourless oil (2.1 mg, 78%). $[\alpha]_D^{20}$ = −6.1 (0.18 g/100 mL, CHCl₃); ¹H NMR (600 MHz, CDCl₃): δ = 7.59 (dd, *J* = 6.8, 3.0 Hz, 2H), 7.40 (m, 3H), 5.61 (ddd, *J* = 5.9, 4.7, 1.1 Hz, 1H), 5.18 (m, 2H), 3.61 (d, *J* = 1.1 Hz, 3H), 2.44 (dd, *J* = 7.7, 5.0 Hz, 2H), 2.25 (dd, *J* = 10.9, 5.1 Hz, 1H), 2.16 (m, 4H), 2.10 (ddd, *J* = 13.1, 10.8, 3.2 Hz, 1H), 1.86 (ddt, *J* = 14.2, 11.1, 3.0 Hz, 1H), 1.59 (s, 3H), 1.52 (s, 3H), 1.12 (s, 3H), 1.04 (s, 3H), 0.93 (dddd, *J* = 14.5, 11.4, 6.1, 3.2 Hz, 1H), 0.67 (m, 1H), 0.64 ppm (dd, *J* = 5.3, 1.1 Hz, 1H); ¹³C NMR (151 MHz, CDCl₃): δ = 165.7, 138.0, 133.2, 132.5, 129.5, 128.3, 127.5, 126.1, 118.2, 90.0, 73.2, 67.1, 55.4, 39.1, 38.7, 34.3, 32.9, 24.8, 24.4, 23.6, 23.4, 20.4, 19.2, 15.5, 15.1 ppm (CF₃ and $C_{q,\ sp3}$ signals are missing); IR (film) $\tilde{\nu}$ = 2971, 2924, 2849, 2236, 1751, 1496, 1452, 1379, 1270, 1250, 1239, 1185,1169, 1123, 1081, 1018, 992, 964, 717 cm^{-1}; HRMS (ESI): *m/z* calcd. for $C_{29}H_{35}F_3O_3$ [M^++Na]: 511.24305; found: 511.24328.

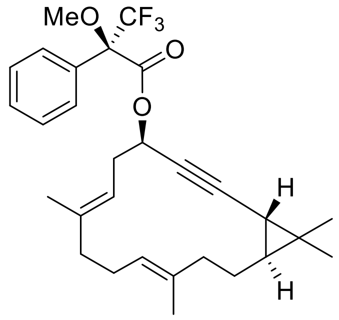

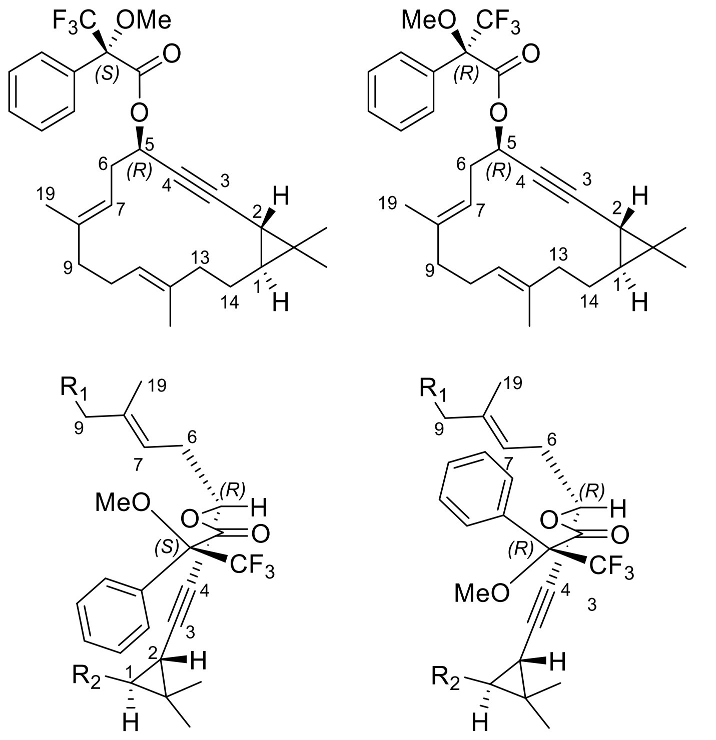

Position	δ_S (^{1}H, ppm)	δ_R (^{1}H, ppm)	$\Delta\delta^{SR} = \delta_S - \delta_R$
14a	0.93	0.93	±0.00
14b	1.85	1.86	−0.01
1	0.64	0.67	−0.03
2	0.61	0.64	−0.03
3	-	-	-
4	-	-	-
5	5.59	5.61	−0.02
6	2.50	2.44	+0.06
7	5.18	5.17	+0.01
19	1.55	1.52	+0.03
9	2.14	2.14	±0.00

Mosher Ester S16. Prepared analogously from compound **41** and (*R*)-(−)-α-methoxy-α-(trifluormethyl)phenylacetyl chloride as a colourless oil (2.2 mg, 62%). $[\alpha]_D^{20}$ = +92.4 (0.17 g/100 mL, CHCl$_3$); ^{1}H NMR (600 MHz, CDCl$_3$): δ = 7.57 (dd, *J* = 7.7, 2.0 Hz, 2H), 7.39 (m, 3H), 5.57 (ddd, *J* = 10.5, 4.9, 2.3 Hz, 1H), 5.14 (d, *J* = 7.6 Hz, 1H), 5.11 (m, 1H), 3.60 (d, *J* = 1.1 Hz, 3H), 2.48 (m, 1H), 2.35 (m, 2H), 2.18 (m, 4H), 2.08 (td, *J* = 12.5, 3.2 Hz, 1H), 1.87 (ddt, *J* = 15.0, 12.1, 2.9 Hz, 1H), 1.59 (s, 3H), 1.55 (s, 3H), 1.13 (s, 3H), 1.05 (s, 3H), 0.90 (m, 1H), 0.68 (m, 1H), 0.67 ppm (m, 1H); ^{13}C NMR (151 MHz, CDCl$_3$): δ = 165.7, 138.2, 133.3, 132.5, 129.5, 128.3, 127.4, 126.0, 118.3, 90.1, 74.0, 66.7, 55.5, 38.8, 38.4, 34.4, 33.4, 24.8, 24.3, 24.2, 23.5, 20.4, 19.1, 15.3, 15.0 ppm ($\underline{C}F_3$ and $\underline{C}_{q,\,sp3}$ signals are missing); IR (film) $\tilde{\nu}$ = 2923, 2853, 2243, 1191, 1170, 1122, 1082, 116, 991, 921, 909, 718, 698 cm^{-1}; HRMS (ESI): *m/z* calcd. for $C_{29}H_{35}F_3O_3$ [M^++Na]: 511.24305; found: 511.24323.

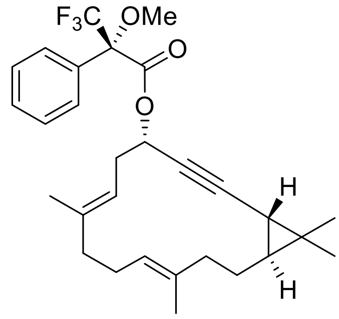

Mosher Ester S17. Prepared analogously from compound **41** and (*S*)-(+)-α-methoxy-α-(trifluormethyl)phenylacetyl chloride as a colourless oil (5.3 mg, 72%). $[\alpha]_D^{20}$ = −101.7 (0.53 g/100 mL, CHCl$_3$); ^{1}H NMR (600 MHz, CDCl$_3$): δ = 7.56 (dd, *J* = 7.4, 2.2 Hz, 2H), 7.40 (m, 3H), 5.55 (ddd, *J* = 10.4, 5.0, 2.1 Hz, 1H), 5.13 (m, 2H), 3.56 (d, *J* = 1.1 Hz, 3H), 2.54 (m, 1H), 2.46 (m, 1H), 2.33 (dq, *J* = 15.9, 7.5 Hz, 1H), 2.19 (t, *J* = 5.1 Hz, 2H), 2.14 (br-s, 2H), 2.07 (td, *J* = 12.6, 3.3 Hz, 1H), 1.85 (ddd, *J* = 12.2, 10.2, 3.1 Hz, 1H), 1.58 (s, 3H), 1.56 (s, 3H), 1.04 (s, 3H), 1.04 (s, 3H), 0.89 (m, 1H), 0.65 (m, 1H), 0.64 ppm (s, 1H); ^{13}C NMR (151 MHz, CDCl$_3$): δ = 165.6, 138.2, 133.3, 132.2, 129.5, 128.3, 127.6, 125.9, 118.3, 90.0, 73.8, 67.0, 55.5, 38.8, 38.5, 34.4, 33.5, 24.8, 24.3, 24.2, 23.4, 20.4, 19.0, 15.3, 15.0 ppm ($\underline{C}F_3$ and $\underline{C}_{q,\,sp3}$ signals are missing); IR (film) $\tilde{\nu}$ = 2924, 2852, 2236, 1749, 1452, 1379, 1352, 1252, 1185, 1168, 1121, 990, 964, 920, 871, 801, 764, 720, 696 cm^{-1}; HRMS (ESI): *m/z* calcd. for $C_{29}H_{35}F_3O_3$ [M^++Na]: 511.24305; found: 511.24327.

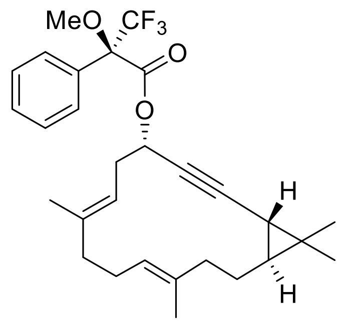

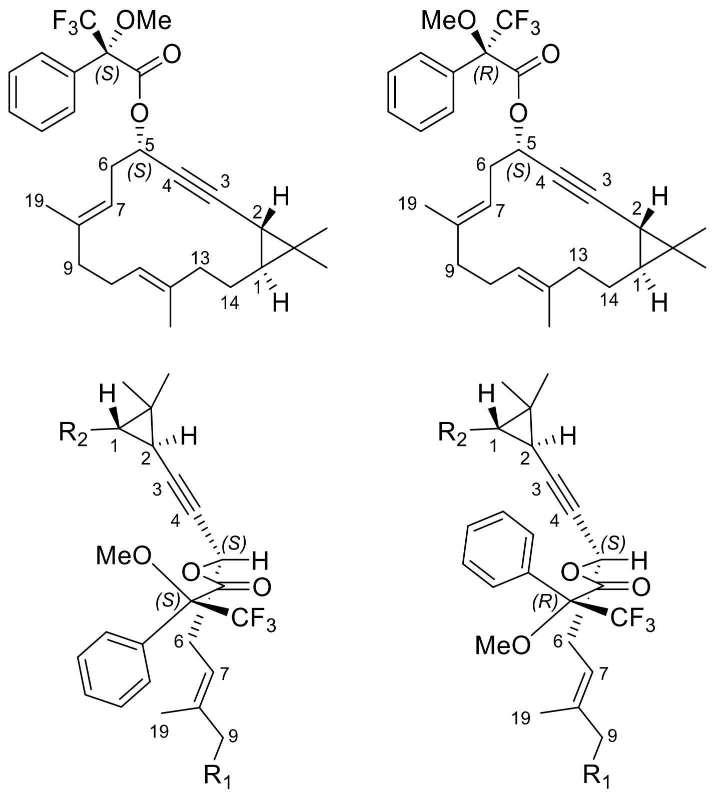

Position	δ_S (^{1}H, ppm)	δ_R (^{1}H, ppm)	$\Delta\delta^{SR} = \delta_S - \delta_R$
14a	0.91	0.89	+0.02
14b	1.87	1.85	+0.02
1	0.68	0.65	+0.03
2	0.67	0.64	+0.03
3	-	-	-
4	-	-	-
5	5.57	5.55	+0.02
6a	2.36	2.46	−0.10
6b	2.48	2.54	−0.06
7	5.11	5.12	−0.01
19	1.55	1.56	−0.01
9	2.19	2.19	±0.00

Mosher Ester S18. Prepared analogously from side product **S6** and (*R*)-(−)-α-methoxy-α-(trifluormethyl)phenylacetyl chloride as a colourless oil (2,3 mg, 4.7 µmol, 85%). $[\alpha]_D^{20}$ = +92.5 (0.16 g/100 mL, $CHCl_3$); ^{1}H NMR (600 MHz, $CDCl_3$): δ = 7.52 (m, 2H), 7.39 (m, 3H), 5.48 (ddt, *J* = 12.5, 8.3, 4.1 Hz, 1H), 5.36 (dd, *J* = 15.2, 8.7 Hz, 1H), 5.27 (dd, *J* = 15.3, 8.2 Hz, 1H), 5.00 (m, 2H), 3.58 (d, *J* = 1.2 Hz, 3H), 2.53 (dt, *J* = 11.8, 5.7 Hz, 1H), 2.37 (dt, *J* = 14.0, 8.4 Hz, 1H), 2.14 (m, 1H), 2.09 (t, *J* = 4.8 Hz, 2H), 2.03 (m, 1H), 1.91 (ddt, *J* = 14.5, 11.1, 3.6 Hz, 1H), 1.57 (s, 3H), 1.55 (s, 3H), 1.04 (m, 7H), 0.78 (dd, *J* = 8.6, 5.2 Hz, 1H), 0.40 ppm (m, 1H); ^{13}C NMR (151 MHz, $CDCl_3$): δ = 165.7, 138.7, 137.2, 133.3, 132.5, 129.4, 128.3, 127.5, 125.6, 124.8, 119.1, 78.3, 55.4, 39.2, 38.6, 32.74, 32.69, 31.8, 24.4, 24.3, 23.1, 22.7, 21.7, 16.2, 14.8 ppm ($\underline{C}F_3$ and $\underline{C}_{q,\,sp3}$ signals are missing); IR (film) $\tilde{\nu}$ = 2924, 2853, 1744, 1663, 1497, 1452, 1378, 1270, 1259, 1290, 1169, 1121, 1081, 1018, 991, 963, 919, 720, 697 cm^{-1}. HRMS (ESI): *m/z* calcd. for $C_{29}H_{37}F_3O_3$ [M^++Na]: 513.25870; found: 513.25917.

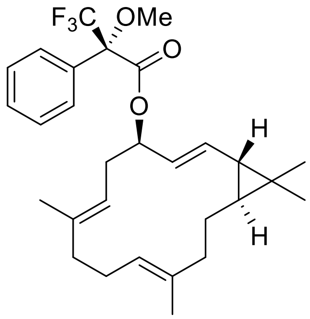

Mosher Ester S19. Prepared analogously from side product **S6** and (*S*)-(+)-α-methoxy-α-(trifluormethyl)phenylacetyl chloride as a colourless oil (1.9 mg, 3.9 µmol, 71%). $[\alpha]_D^{20}$ = +56.7 (0.03 g/100 mL, $CHCl_3$); ^{1}H NMR (600 MHz, $CDCl_3$): δ = 7.54 (m, 2H), 7.39 (m, 3H), 5.49 (m, 1H), 5.40 (m, 2H), 5.02 (t, *J* = 6.0 Hz, 1H), 4.97 (t, *J* = 7.1 Hz, 1H), 3.57 (d, *J* = 1.2 Hz, 3H), 2.48 (dt, *J* = 12.1, 5.6 Hz, 1H), 2.27 (m, 1H), 2.07 (m, 6H), 1.92 (m, 1H), 1.56 (s, 6H), 1.04 (s, 3H), 1.01 (m, 4H), 0.82 (m, 1H), 0.41 ppm (dt, *J* = 11.2, 4.4 Hz, 1H); ^{13}C NMR (151 MHz, $CDCl_3$): δ = 165.8, 138.7, 137.1, 133.2, 132.7, 129.5, 128.3, 127.4, 125.6, 125.0, 119.1, 78.2, 55.3, 39.2, 38.6, 32.8, 32.6, 31.6, 24.4, 24.3, 23.1, 22.7, 21.7, 16.1, 14.8 ppm ($\underline{C}F_3$ and $\underline{C}_{q,\,sp3}$ signals are missing); IR (film) $\tilde{\nu}$ = 2923, 2852, 1745, 1452, 1378, 1270, 1259, 1180, 1169, 1122, 1081, 1020, 992, 964, 919, 719 cm^{-1}. HRMS (ESI): *m/z* calcd. for $C_{29}H_{37}F_3O_3$ [M^++Na]: 513.25870; found: 513.25897.

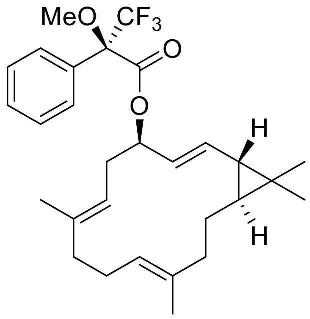

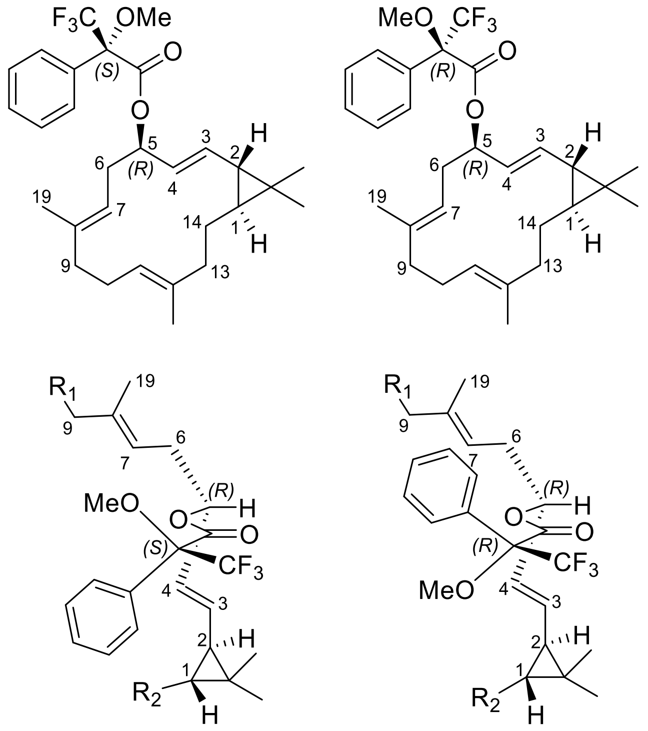

Position	δ_S (^{1}H, ppm)	δ_R (^{1}H, ppm)	$\Delta\delta^{SR} = \delta_S - \delta_R$
14a	1.00	1.01	−0.01
14b	1.91	1.92	−0.01
1	0.40	0.41	−0.01
2	0.78	0.81	−0.03
3	5.35	5.40	−0.05
4	5.28	5.39	−0.11
5	5.48	5.49	−0.01
6a	2.38	2.27	+0.11
6b	2.54	2.48	+0.06
7	5.01	4.97	+0.04
19	1.57	1.56	+0.01
9	2.10	2.08	+0.02

Mosher Ester S20. Prepared analogously from side product **S7** and (*R*)-(−)-α-methoxy-α-(trifluormethyl)phenylacetyl chloride as a colourless oil (2.4 mg, 4.9 µmol, 90%). $[\alpha]_D^{20}$ = −14.6 (0.35 g/100 mL, $CHCl_3$); ^1H NMR (600 MHz, $CDCl_3$): δ = 7.53 (m, 2H), 7.38 (m, 3H), 5.62 (dd, *J* = 15.8, 8.2 Hz, 1H), 5.53 (m, 1H), 5.47 (dd, *J* = 15.6, 6.1 Hz, 1H), 4.98 (dd, *J* = 9.6, 5.0 Hz, 1H), 4.94 (m, 1H), 3.55 (d, *J* = 1.2 Hz, 3H), 2.54 (dt, *J* = 14.1, 9.6 Hz, 1H), 2.31 (d, *J* = 15.3 Hz, 1H), 2.25 (m, 1H), 2.10 (m, 4H), 2.00 (td, *J* = 12.4, 3.3 Hz, 1H), 1.92 (m, 1H), 1.55 (s, 3H), 1.51 (s, 3H), 1.06 (s, 3H), 0.97 (m, 4H), 0.79 (dd, *J* = 8.0, 5.4 Hz, 1H), 0.46 ppm (ddd, *J* = 11.4, 5.4, 3.5 Hz, 1H); ^{13}C NMR (151 MHz, $CDCl_3$): δ = 165.9, 137.0, 136.4, 133.6, 132.6, 129.5, 127.4, 125.5, 123.4, 119.7, 75.3, 55.4, 39.0, 38.6, 32.7, 32.0, 31.3, 29.7, 24.6, 24.3, 24.3, 22.3, 21.8, 15.5, 14.8 ppm ($\underline{C}F_3$ and $\underline{C}_{q,\ sp3}$ signals are missing); IR (film) $\tilde{\nu}$ = 2961, 2923, 2852, 1744, 1662, 1451, 1378, 1258, 1184, 1168, 1081, 1012, 865, 791, 719, 697, 661 cm^{-1}; HRMS (ESI): *m/z* calcd. for $C_{29}H_{37}F_3O_3$ [M^++Na]: 513.25870; found: 513.25889.

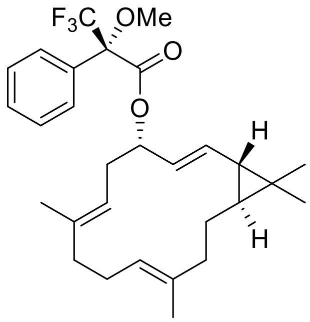

Mosher Ester S21. Prepared analogously from side product **S7** and (*S*)-(+)-α-methoxy-α-(trifluormethyl)phenylacetyl chloride as a colourless oil (2.2 mg, 4.5 µmol, 82%). $[\alpha]_D^{20}$ = −108.6 (0.14 g/100 mL, $CHCl_3$); ^1H NMR (600 MHz, $CDCl_3$): δ = 7.52 (m, 2H), 7.38 (m, 3H), 5.52 (m, 2H), 5.42 (dd, *J* = 15.7, 5.8 Hz, 1H), 5.02 (t, *J* = 7.3 Hz, 1H), 4.93 (m, 1H), 3.56 (d, *J* = 1.2 Hz, 3H), 2.59 (dt, *J* = 14.0, 9.4 Hz, 1H), 2.40 (m, 1H), 2.26 (m, 1H), 2.16 (m, 1H), 2.11 (m, 3H), 2.00 (td, *J* = 12.2, 3.4 Hz, 1H), 1.91 (m, 1H), 1.55 (s, 3H), 1.52 (s, 3H), 1.04 (s, 3H), 0.96 (m, 1H), 0.88 (s, 3H), 0.76 (dd, *J* = 7.9, 5.4 Hz, 1H), 0.41 ppm (ddd, *J* = 11.4, 5.4, 3.5 Hz, 1H); ^{13}C NMR (151 MHz, $CDCl_3$): δ = 165.7, 136.6, 136.4, 133.6, 132.5, 129.4, 128.3, 127.4, 125.4, 123.5, 119.6, 75.3, 55.4, 39.0, 38.6, 32.7, 31.9, 31.6, 24.6, 24.3, 24.1, 22.2, 21.7, 15.5, 14.7 ppm ($\underline{C}F_3$ and $\underline{C}_{q,\ sp3}$ signals are missing); IR (film) $\tilde{\nu}$ = 2922, 2851, 1744, 1665, 1452, 1378, 1259, 1185, 1168, 1119, 1102, 1082, 1018, 992, 963, 799, 719 cm^{-1}; HRMS (ESI): *m/z* calcd. for $C_{29}H_{37}F_3O_3$ [M^++Na]: 513.25870; found: 513.25883.

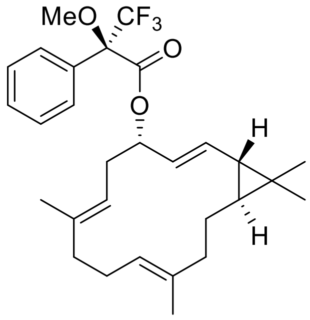

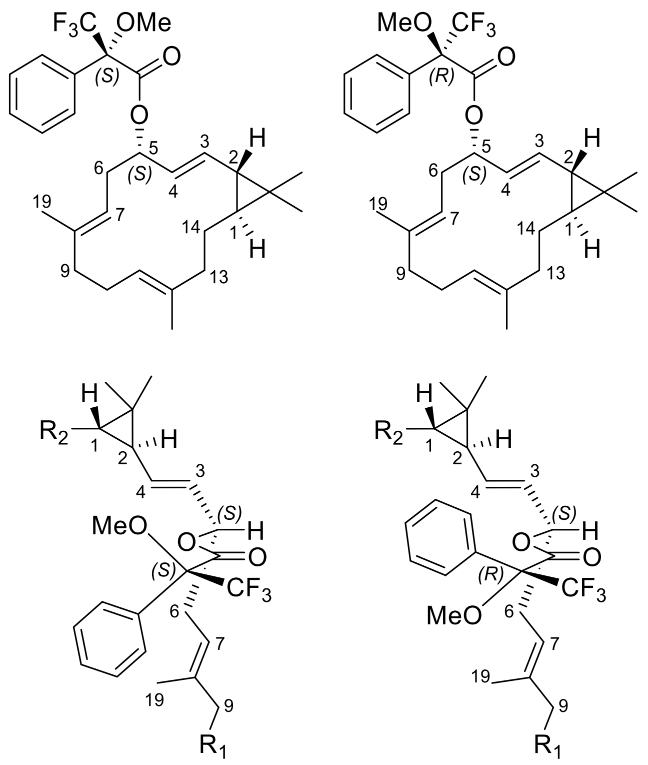

Position	δ_S (^{1}H, ppm)	δ_R (^{1}H, ppm)	$\Delta\delta^{SR} = \delta_S - \delta_R$
14a	0.97	0.95	+0.02
14b	1.92	1.91	+0.01
1	0.46	0.41	+0.05
2	0.80	0.76	+0.04
3	5.62	5.52	+0.10
4	5.47	5.43	+0.04
5	5.53	5.53	±0.00
6a	2.32	2.40	−0.08
6b	2.54	2.59	−0.05
7	4.98	5.02	−0.04
19	1.51	1.53	−0.02
9a	2.08	2.11	−0.03
9b	2.15	2.16	−0.01

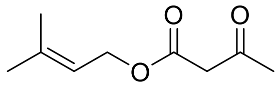

S1

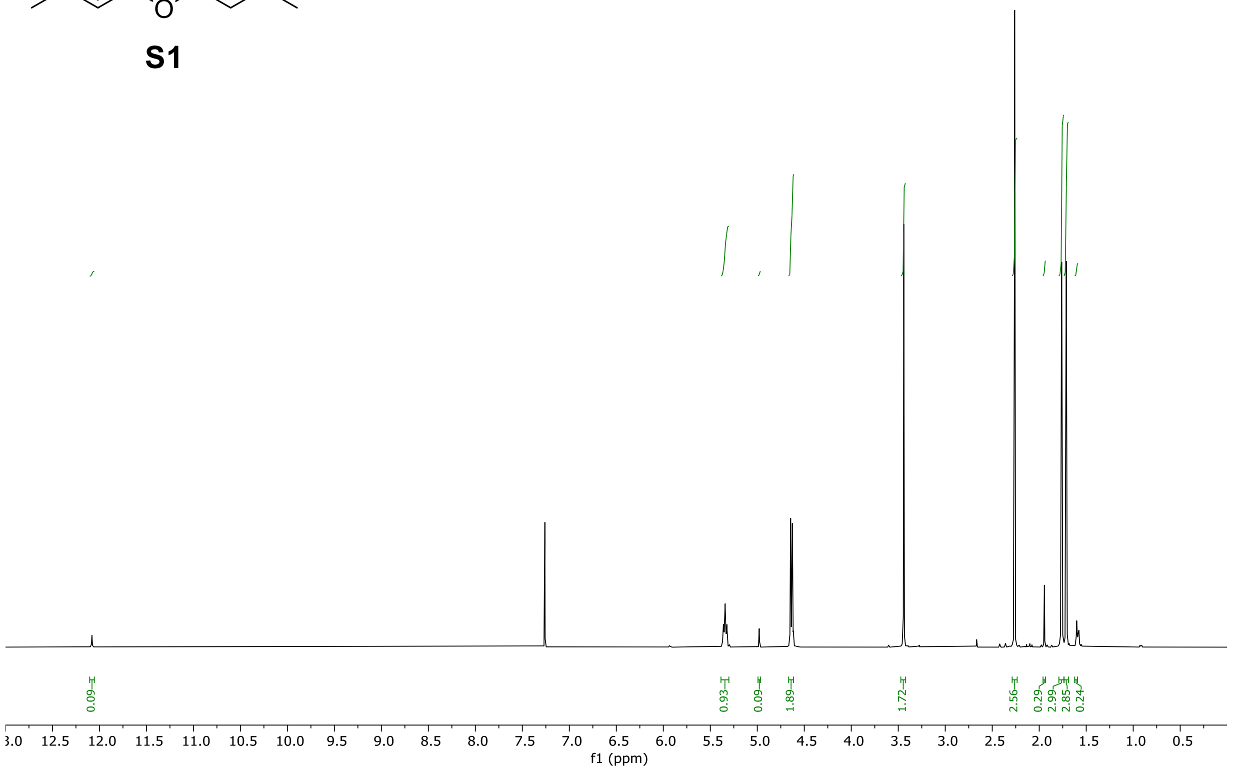

0.09
0.93
0.09
1.89
1.72
2.56
0.29
2.99
2.85
0.24
3.0 12.5 12.0 11.5 11.0 10.5 10.0 9.5 9.0 8.5 8.0 7.5 7.0 6.5 6.0 5.5 5.0 4.5 4.0 3.5 3.0 2.5 2.0 1.5 1.0 0.5
f1 (ppm)

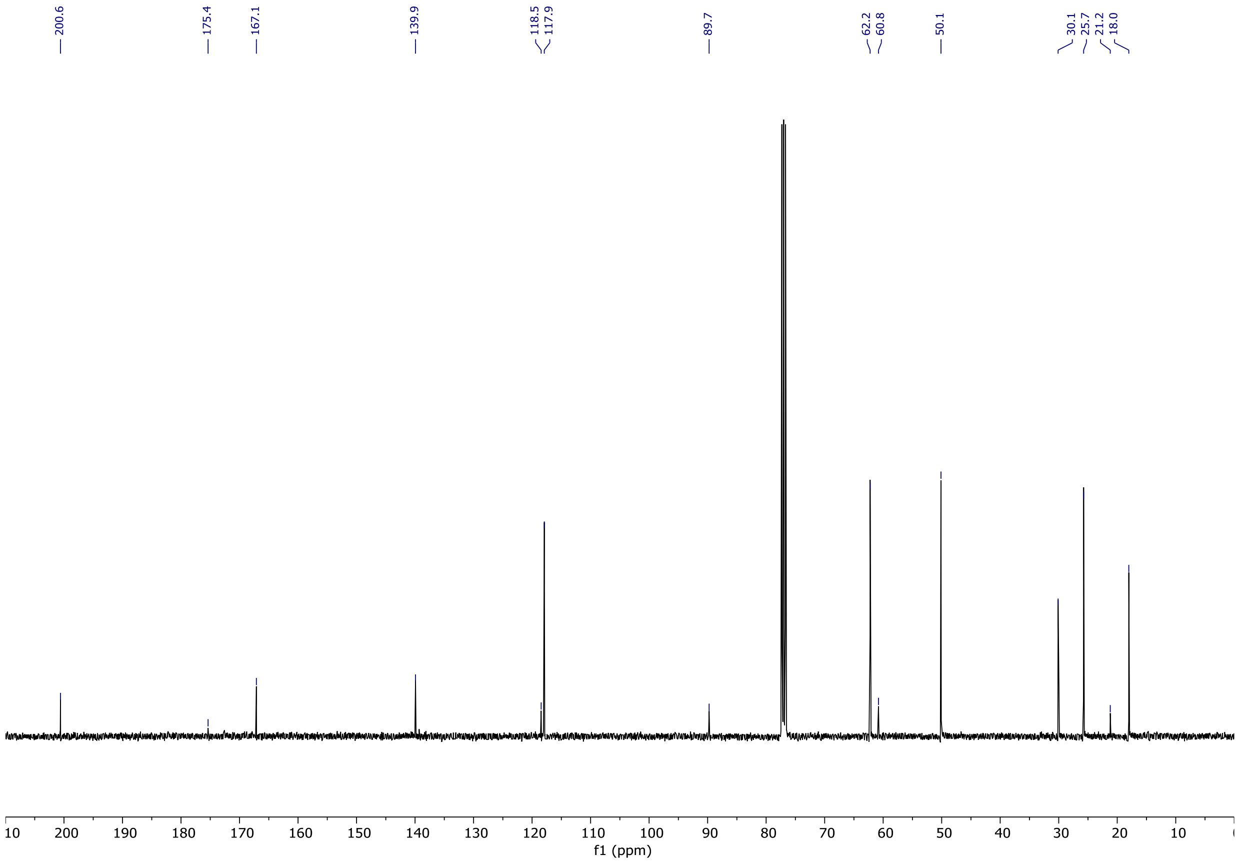

200.6
175.4
167.1
139.9
118.5
117.9
89.7
62.2
60.8
50.1
30.1
25.7
21.2
18.0
10 200 190 180 170 160 150 140 130 120 110 100 90 80 70 60 50 40 30 20 10
f1 (ppm)

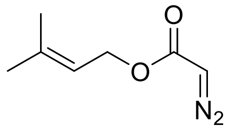
O
O
N2

15

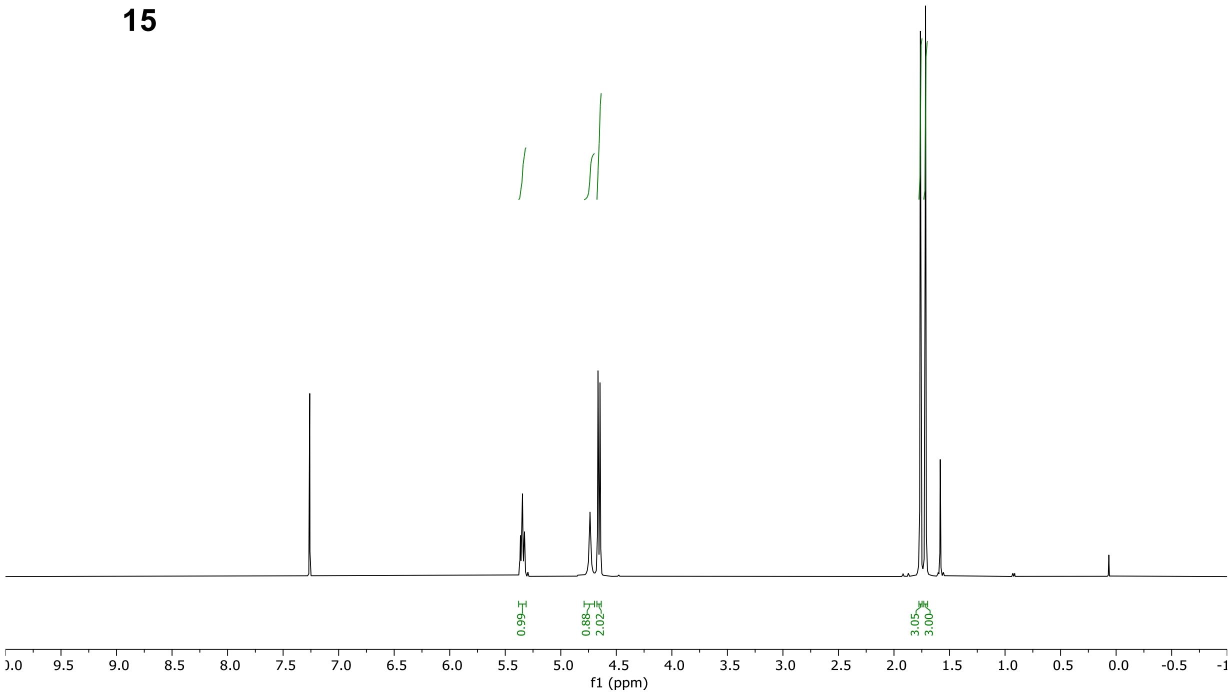
0.99
0.88
2.02
3.05
3.00
f1 (ppm)

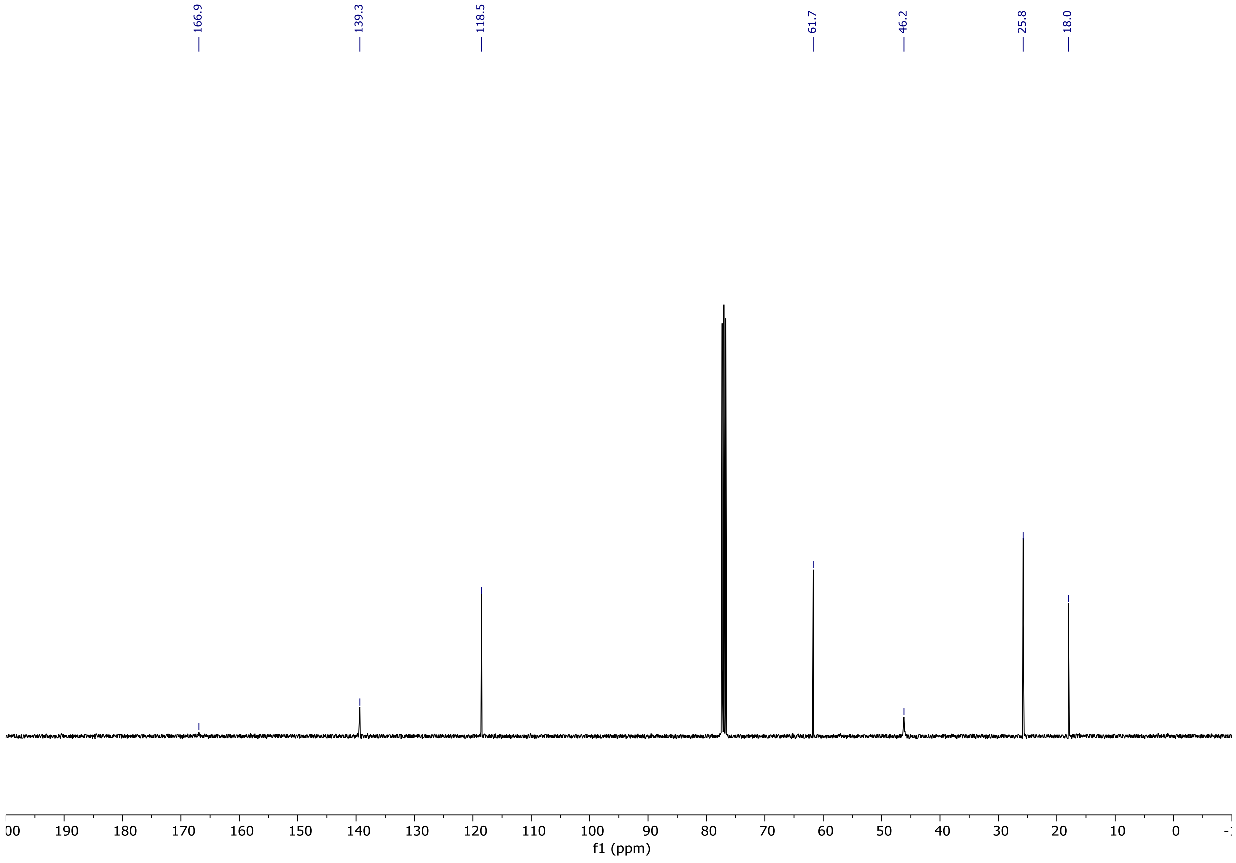
166.9
139.3
118.5
61.7
46.2
25.8
18.0
f1 (ppm)

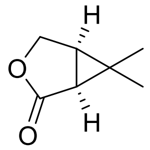

16

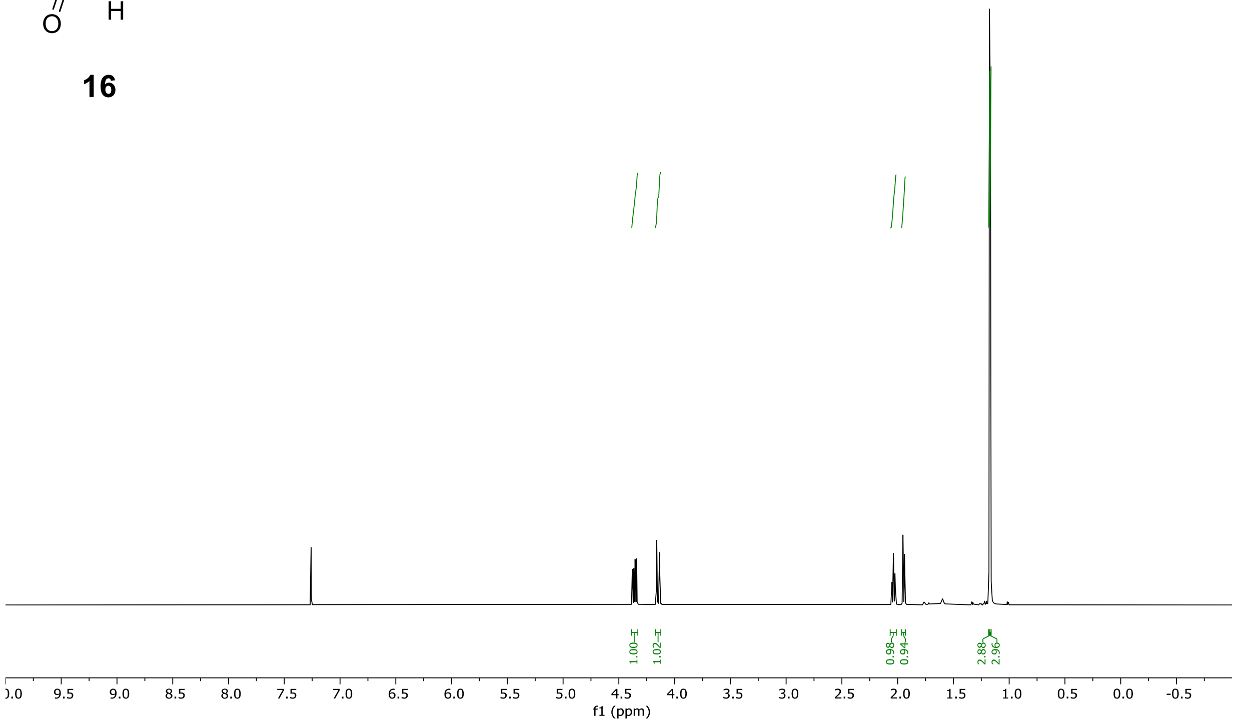

1.00
1.02
0.98
0.94
2.88
2.96
0.0
9.5
9.0
8.5
8.0
7.5
7.0
6.5
6.0
5.5
5.0
4.5
4.0
3.5
3.0
2.5
2.0
1.5
1.0
0.5
0.0
-0.5
f1 (ppm)

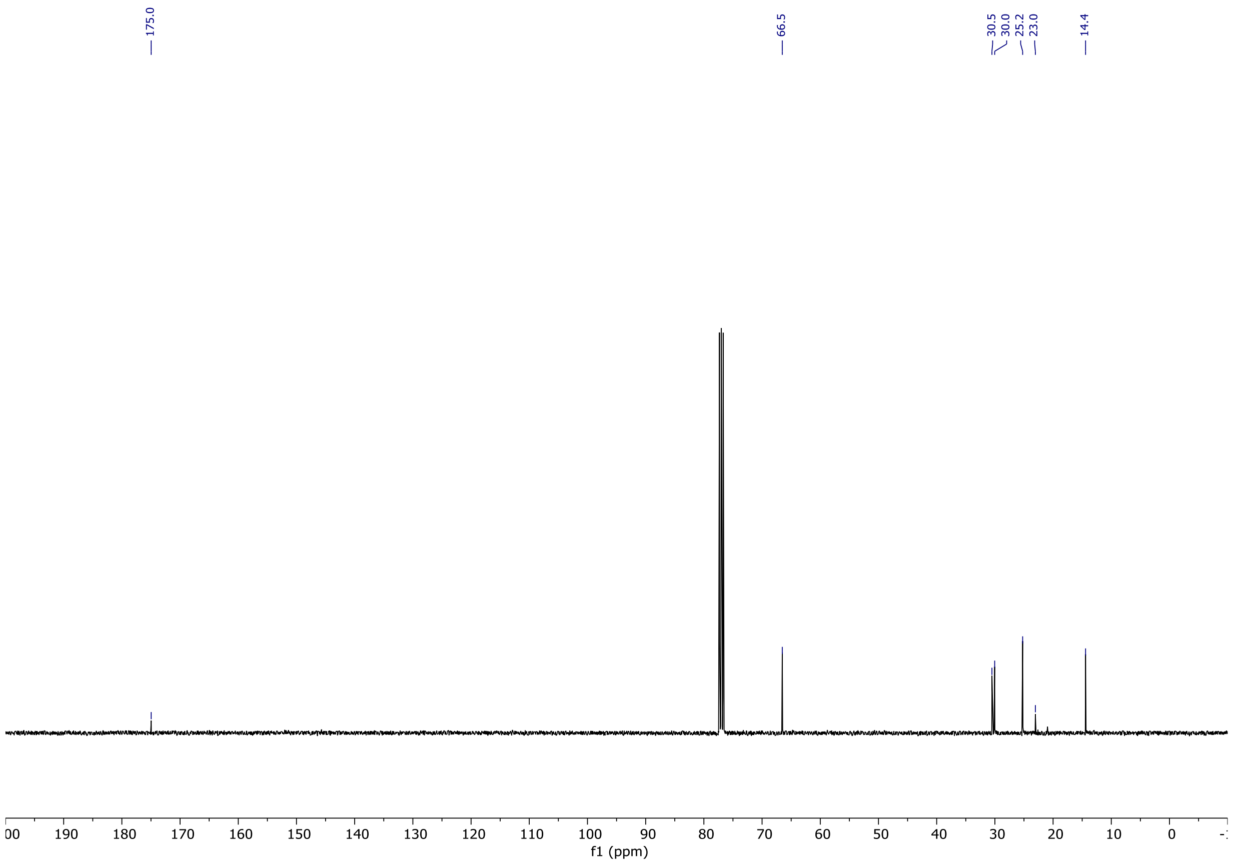

175.0
66.5
30.5
30.0
25.2
23.0
14.4
190
180
170
160
150
140
130
120
110
100
90
80
70
60
50
40
30
20
10
0
f1 (ppm)

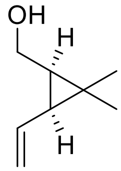
OH
H
H

17

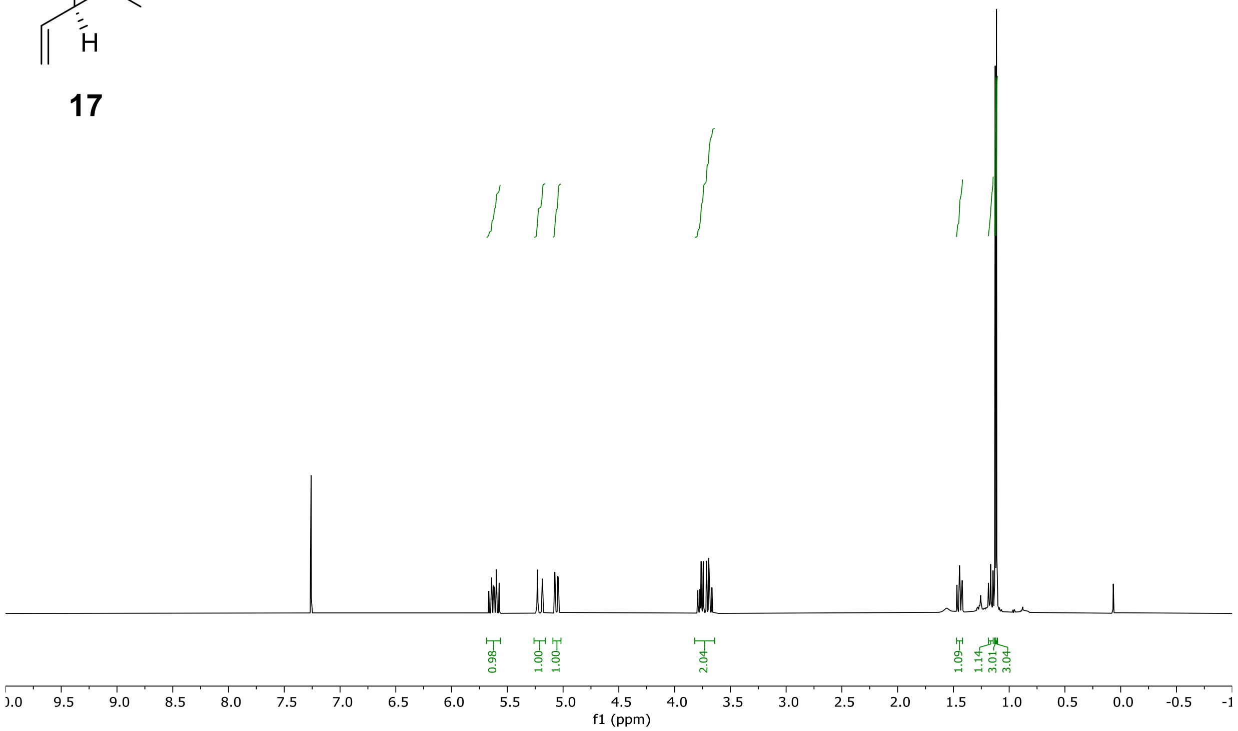
0.98
1.00
1.00
2.04
1.09
1.14
3.01
3.04
f1 (ppm)

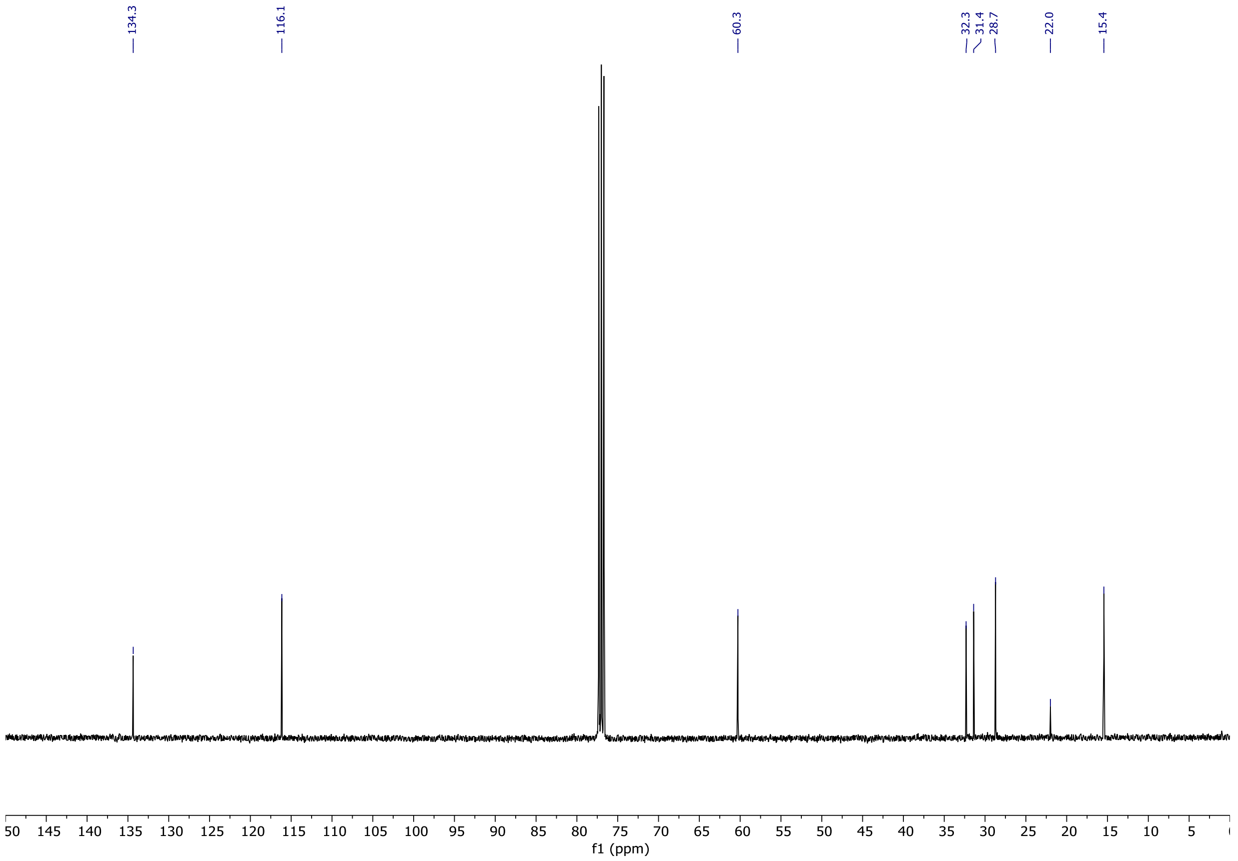
134.3
116.1
60.3
32.3
31.4
28.7
22.0
15.4
f1 (ppm)

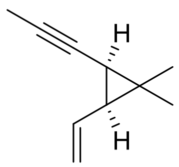
H
H

19

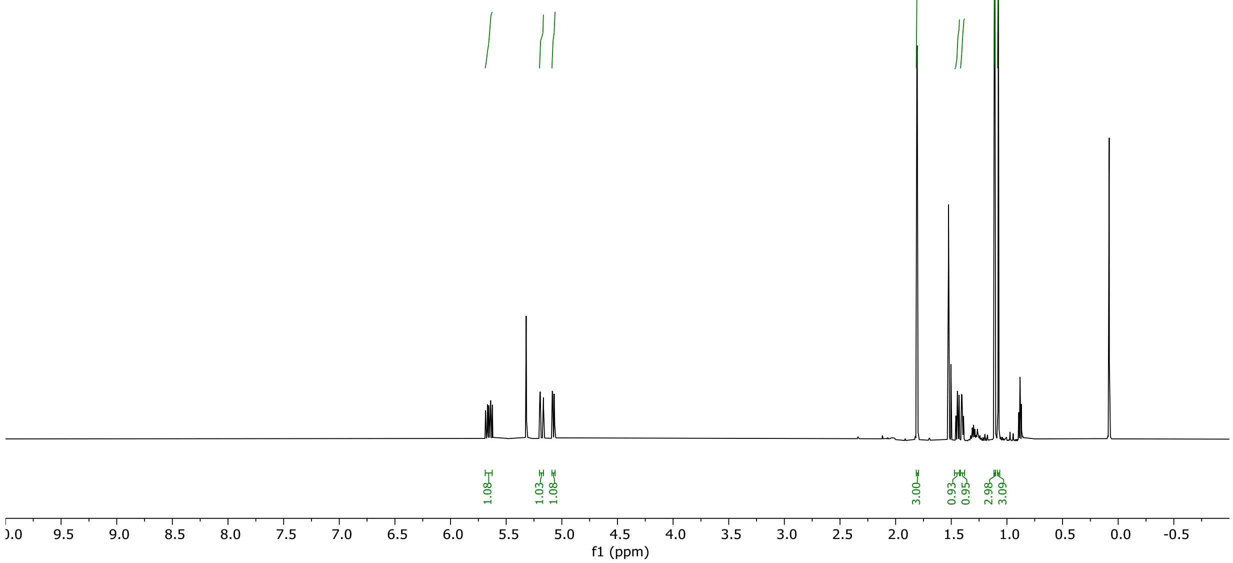
1.08
1.03
1.08
3.00
0.93
0.95
2.98
3.09
f1 (ppm)

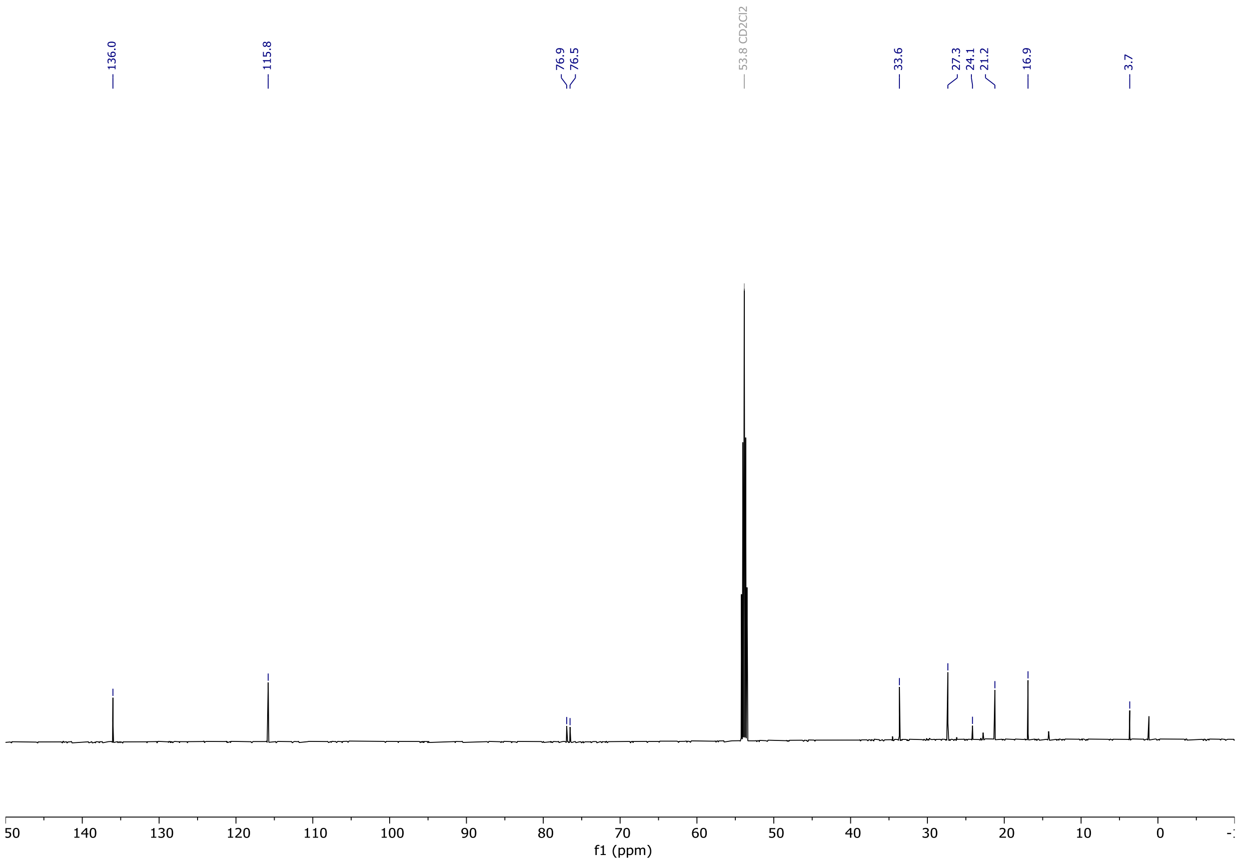
136.0
115.8
76.9
76.5
53.8 CD2Cl2
33.6
27.3
24.1
21.2
16.9
3.7
f1 (ppm)

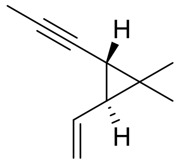

20

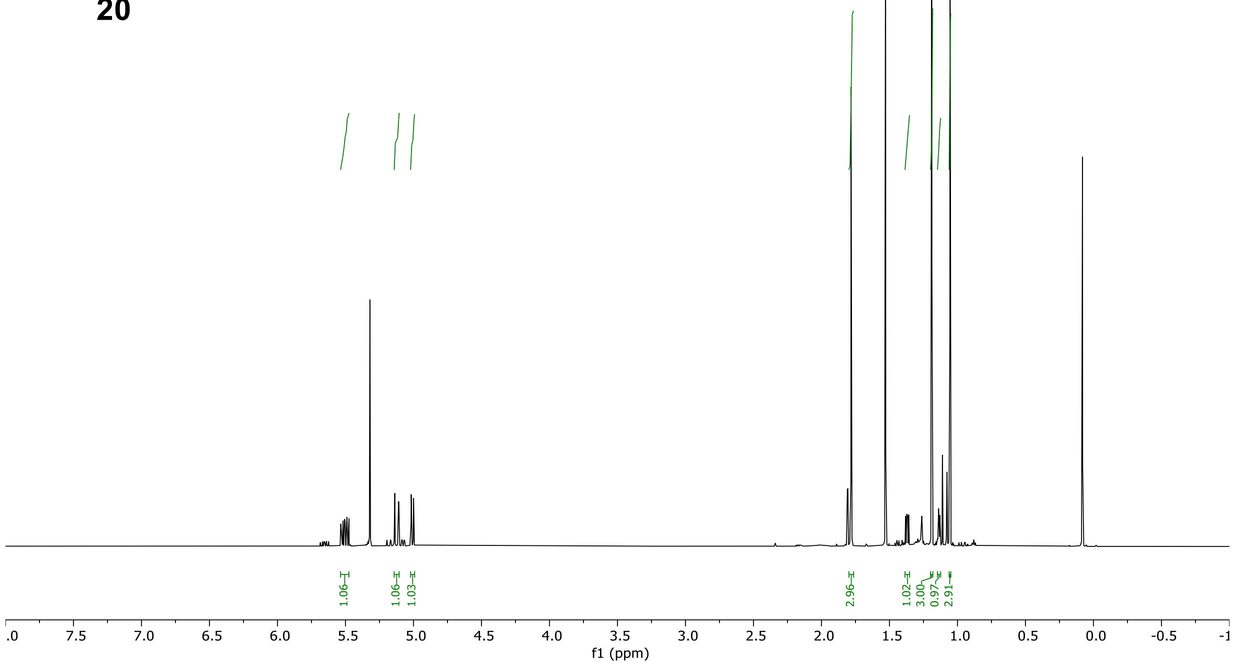
1.06
1.06
1.03
2.96
1.02
3.00
0.97
2.91
f1 (ppm)

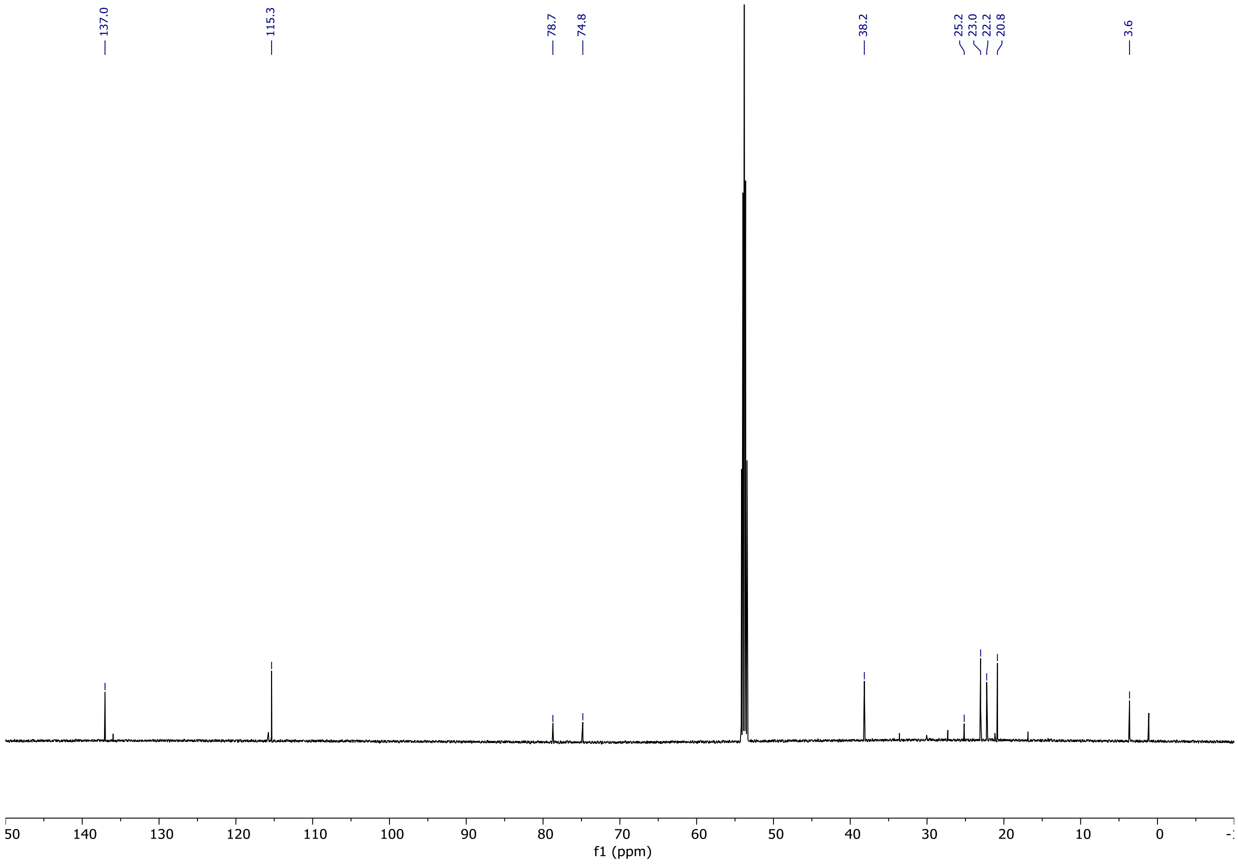
137.0
115.3
78.7
74.8
38.2
25.2
23.0
22.2
20.8
3.6
f1 (ppm)

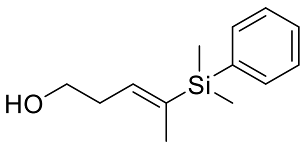
HO
Si

24

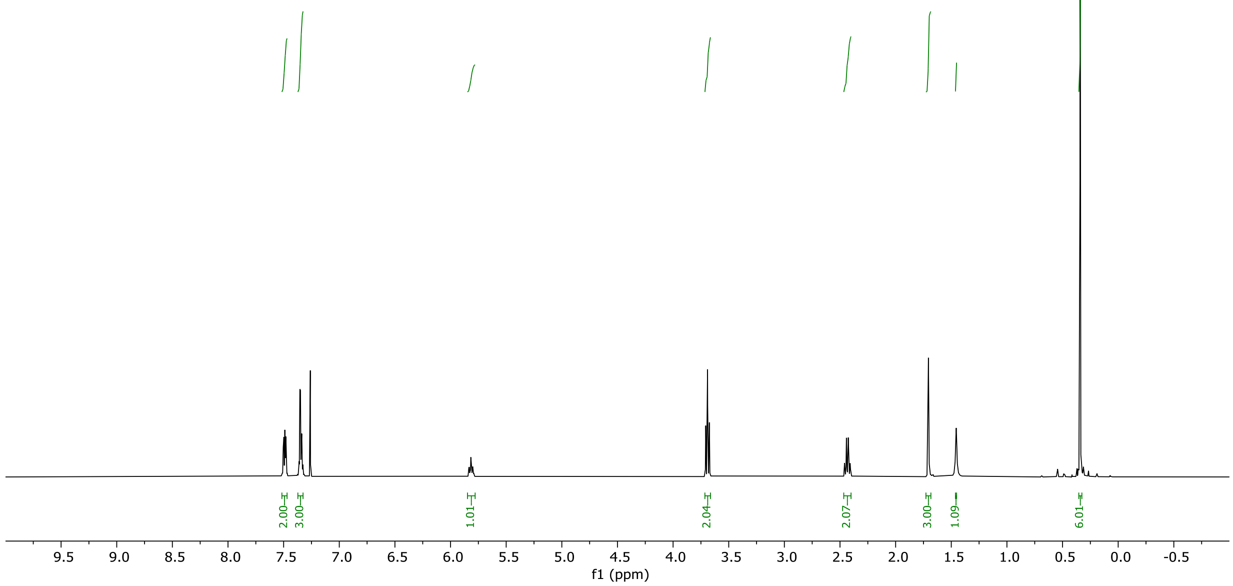
2.00
3.00
1.01
2.04
2.07
3.00
1.09
6.01
9.5 9.0 8.5 8.0 7.5 7.0 6.5 6.0 5.5 5.0 4.5 4.0 3.5 3.0 2.5 2.0 1.5 1.0 0.5 0.0 -0.5
f1 (ppm)

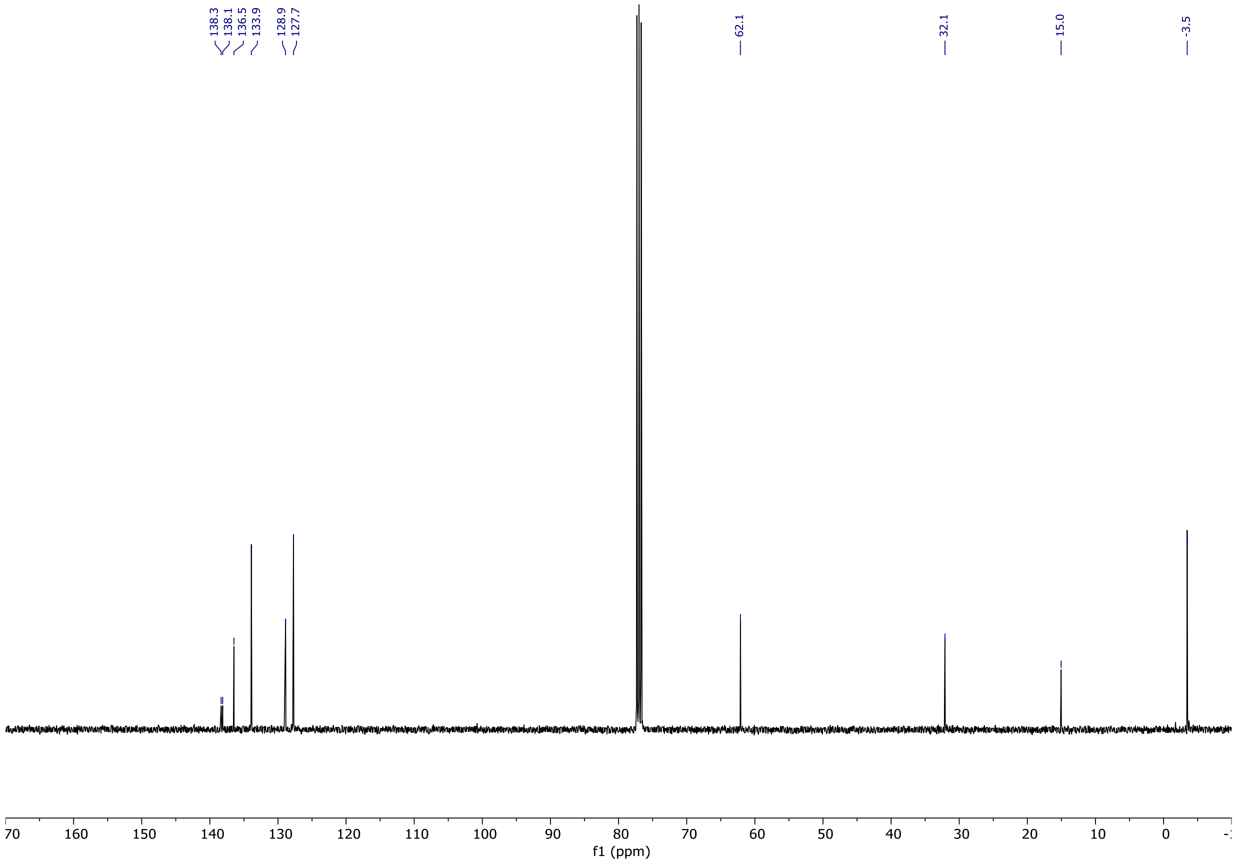
138.3
138.1
136.5
133.9
128.9
127.7
62.1
32.1
15.0
-3.5
160 150 140 130 120 110 100 90 80 70 60 50 40 30 20 10 0
f1 (ppm)

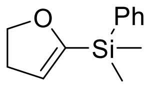
Ph
O
Si

23

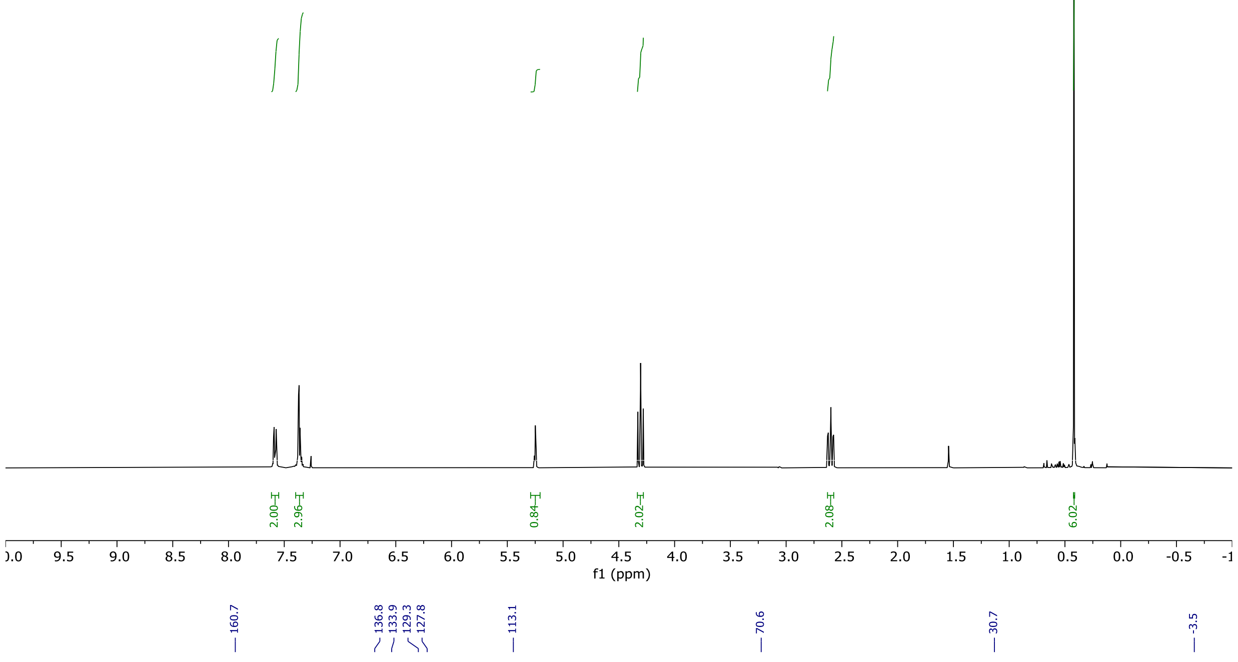
2.00
2.96
0.84
2.02
2.08
6.02
f1 (ppm)
160.7
136.8
133.9
129.3
127.8
113.1
70.6
30.7
-3.5

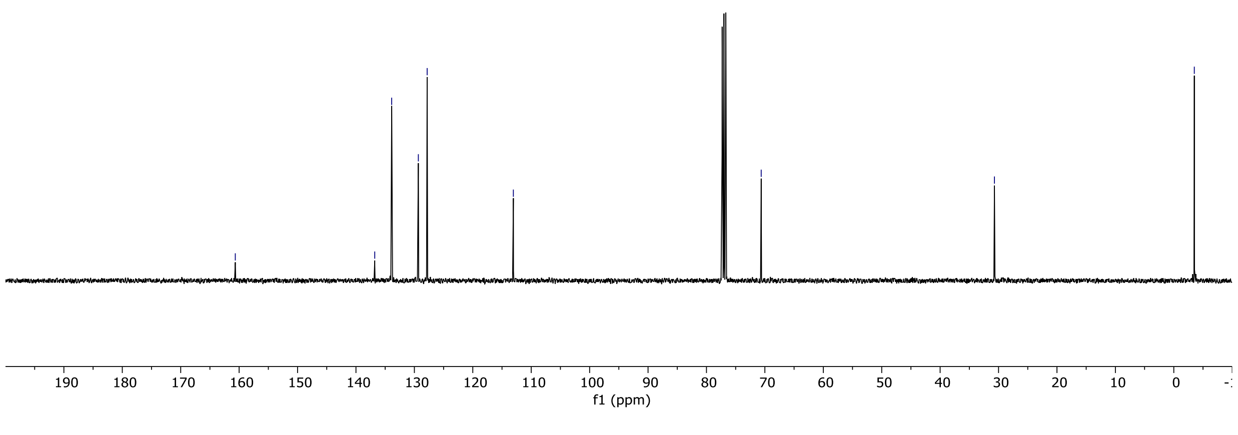
f1 (ppm)

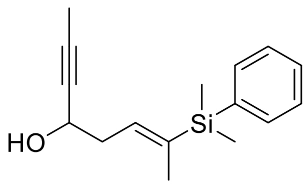
HO
Si

26

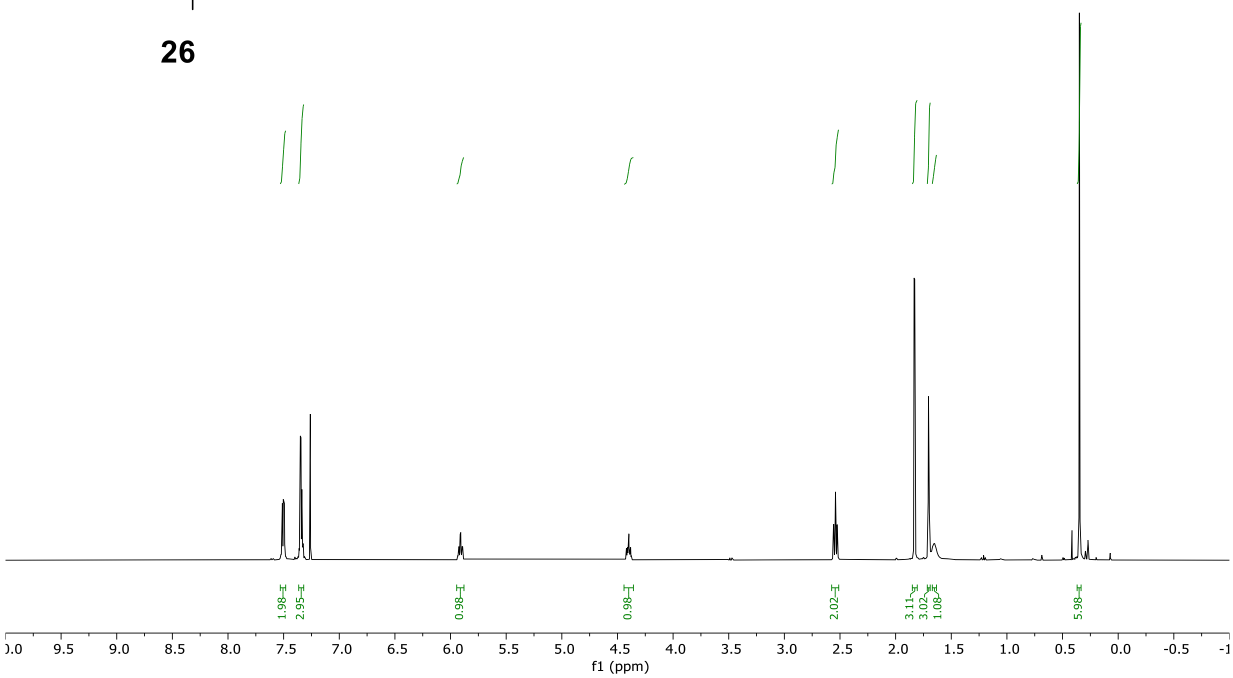
1.98
2.95
0.98
0.98
2.02
3.11
3.02
1.08
5.98
f1 (ppm)

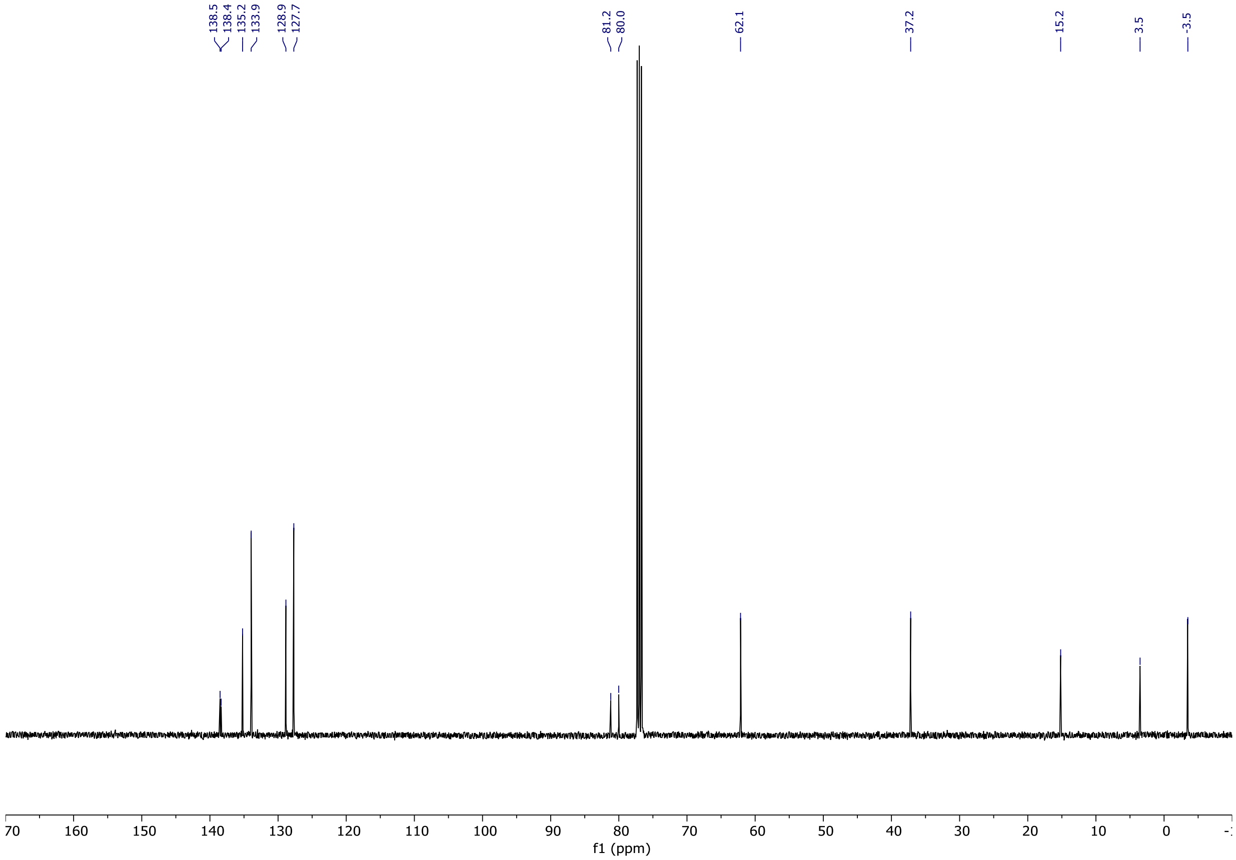
138.5
138.4
135.2
133.9
128.9
127.7
81.2
80.0
62.1
37.2
15.2
3.5
-3.5
f1 (ppm)

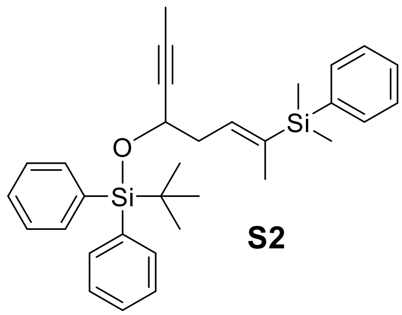
O
Si
Si

S2

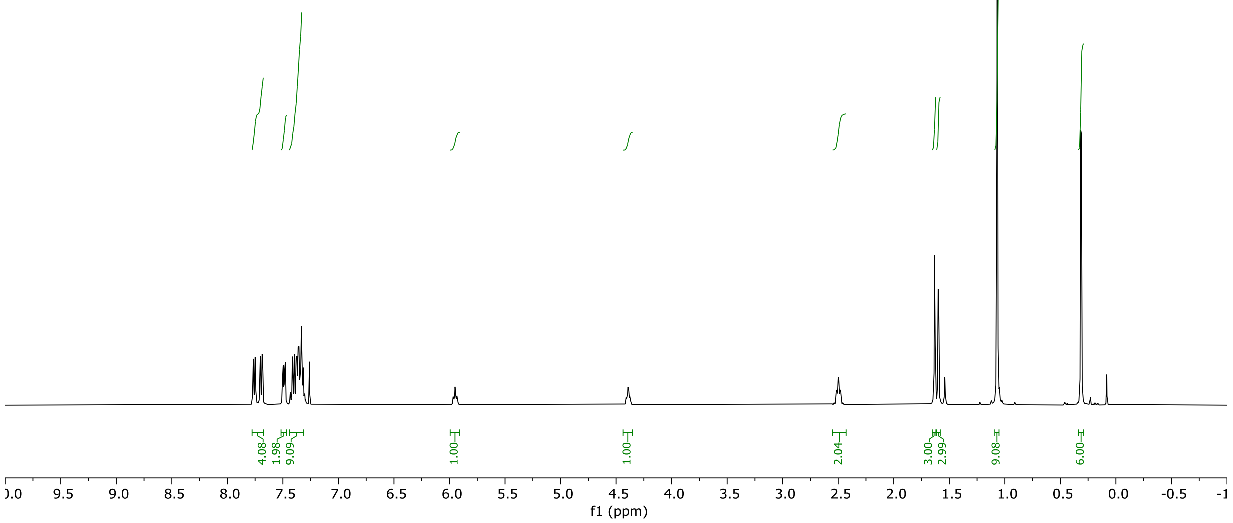
4.08
1.98
9.09
1.00
1.00
2.04
3.00
2.99
9.08
6.00
f1 (ppm)

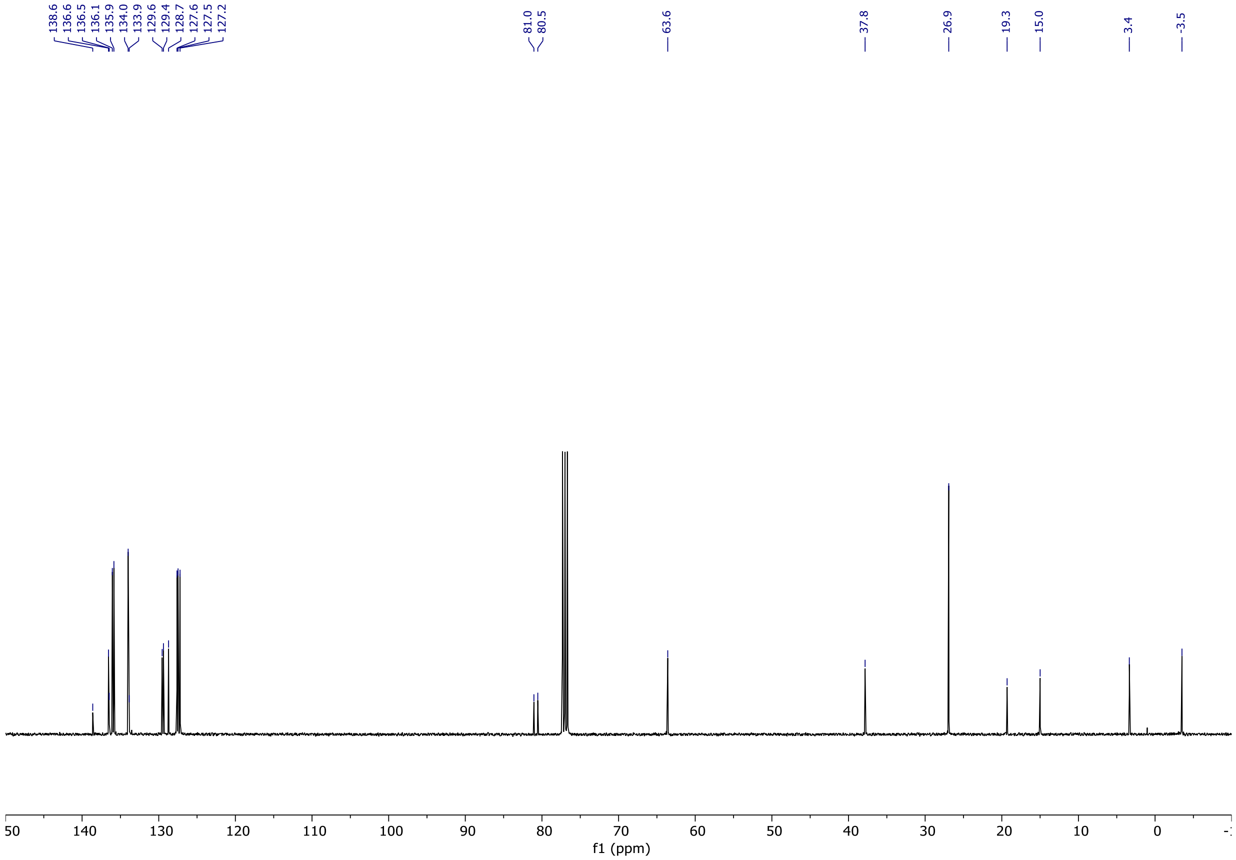
138.6
136.6
136.5
136.1
135.9
134.0
133.9
129.6
129.4
128.7
127.6
127.5
127.2
81.0
80.5
63.6
37.8
26.9
19.3
15.0
3.4
-3.5
f1 (ppm)

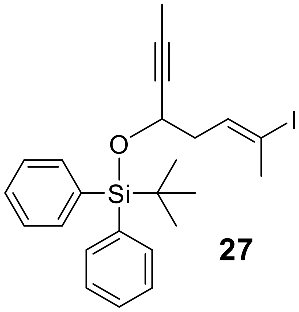

O
Si
I

27

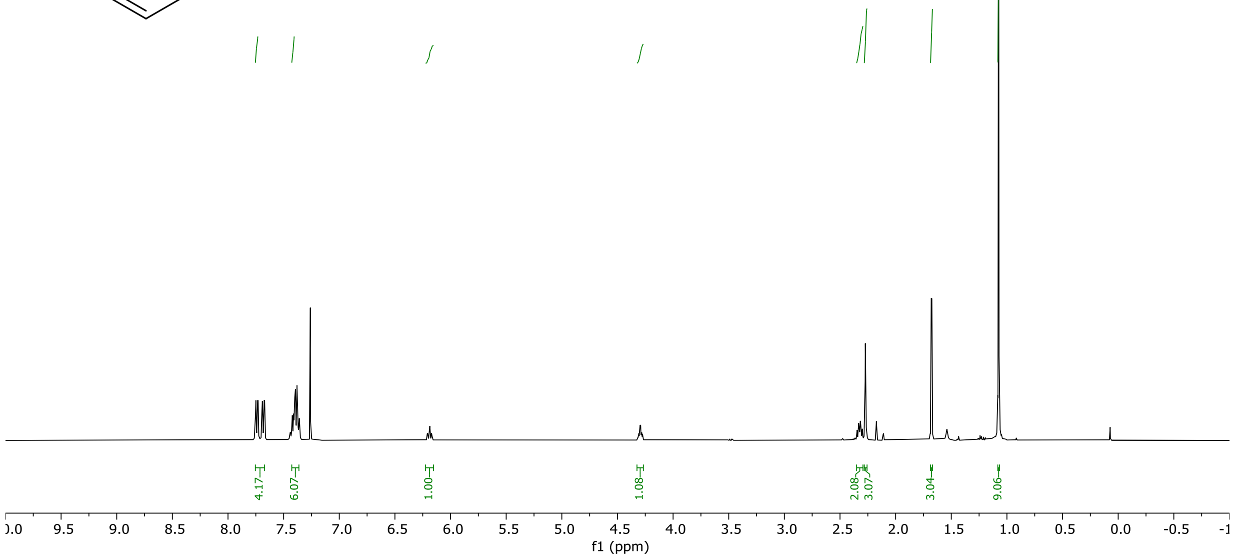

4.17
6.07
1.00
1.08
2.08
3.07
3.04
9.06
f1 (ppm)

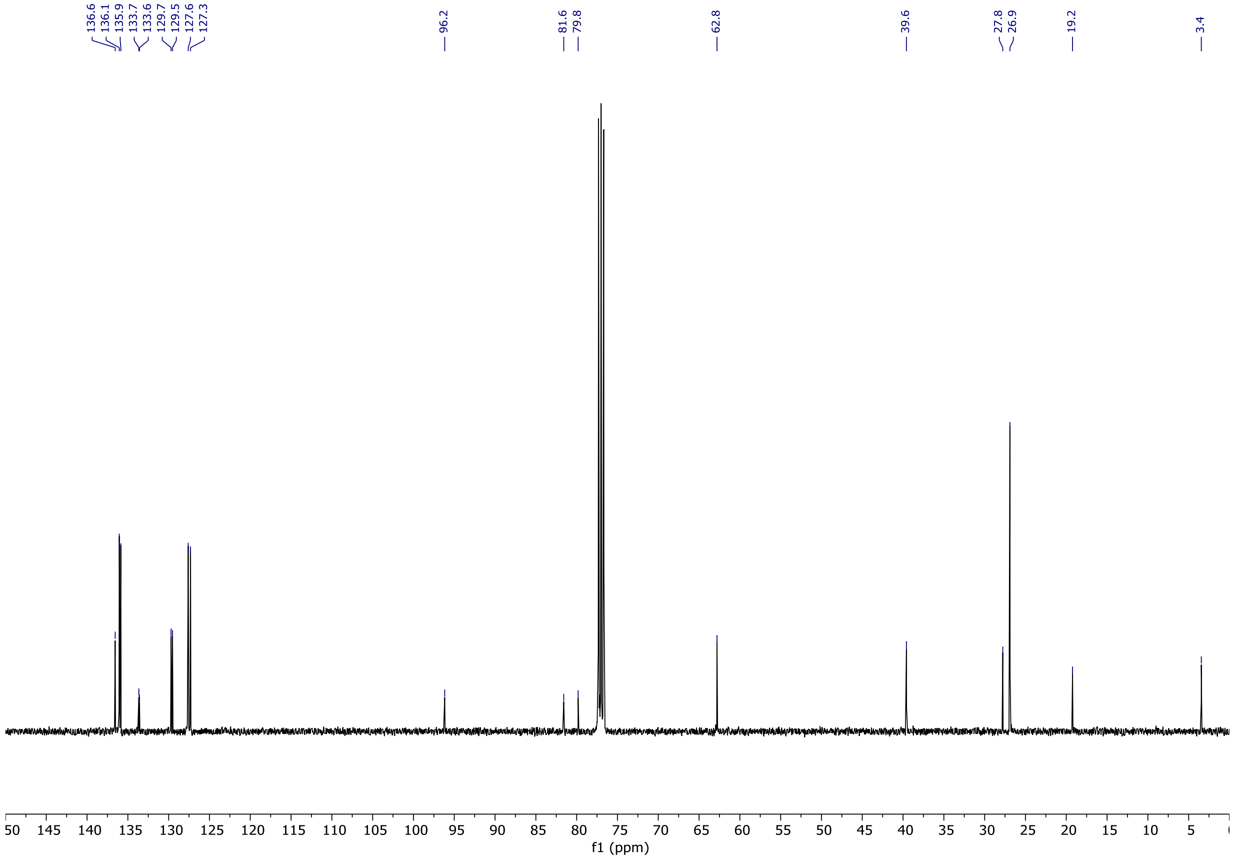

136.6
136.1
135.9
133.7
133.6
129.7
129.5
127.6
127.3
96.2
81.6
79.8
62.8
39.6
27.8
26.9
19.2
3.4
f1 (ppm)

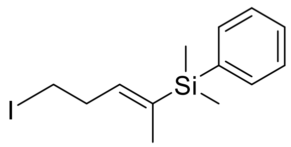
I
Si

25

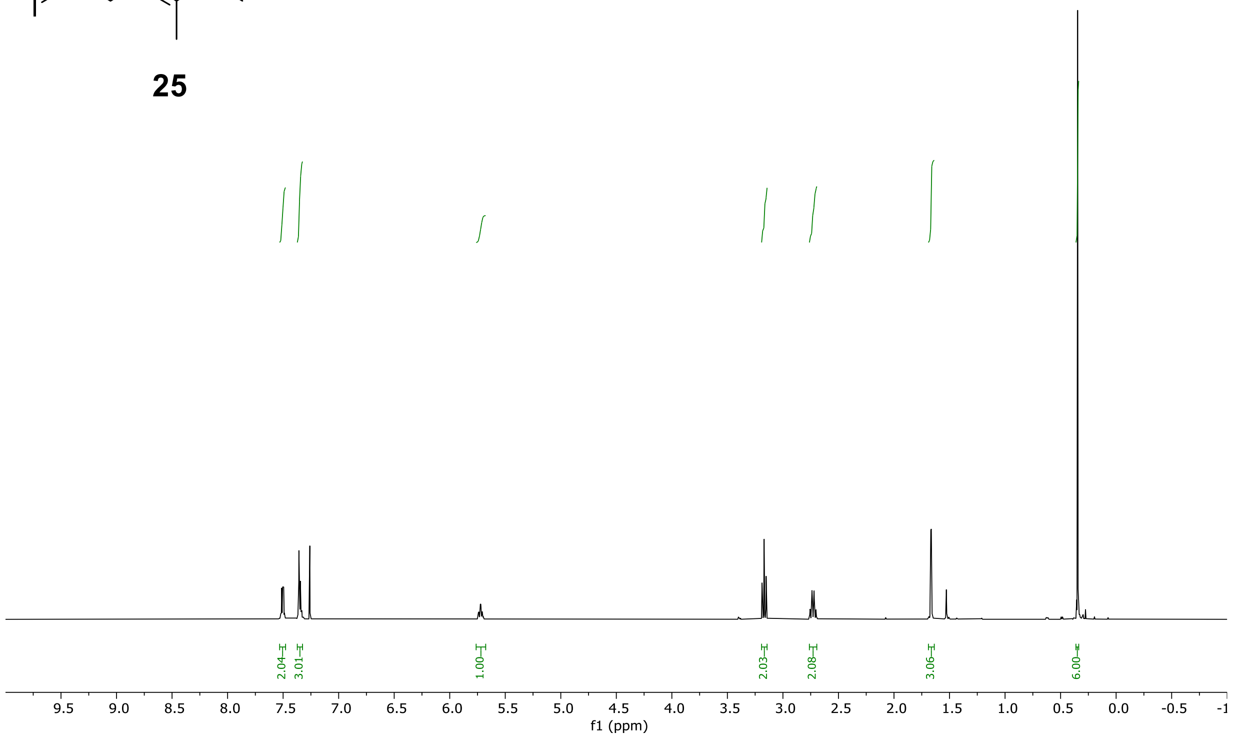
2.04
3.01
1.00
2.03
2.08
3.06
6.00
9.5 9.0 8.5 8.0 7.5 7.0 6.5 6.0 5.5 5.0 4.5 4.0 3.5 3.0 2.5 2.0 1.5 1.0 0.5 0.0 -0.5
f1 (ppm)

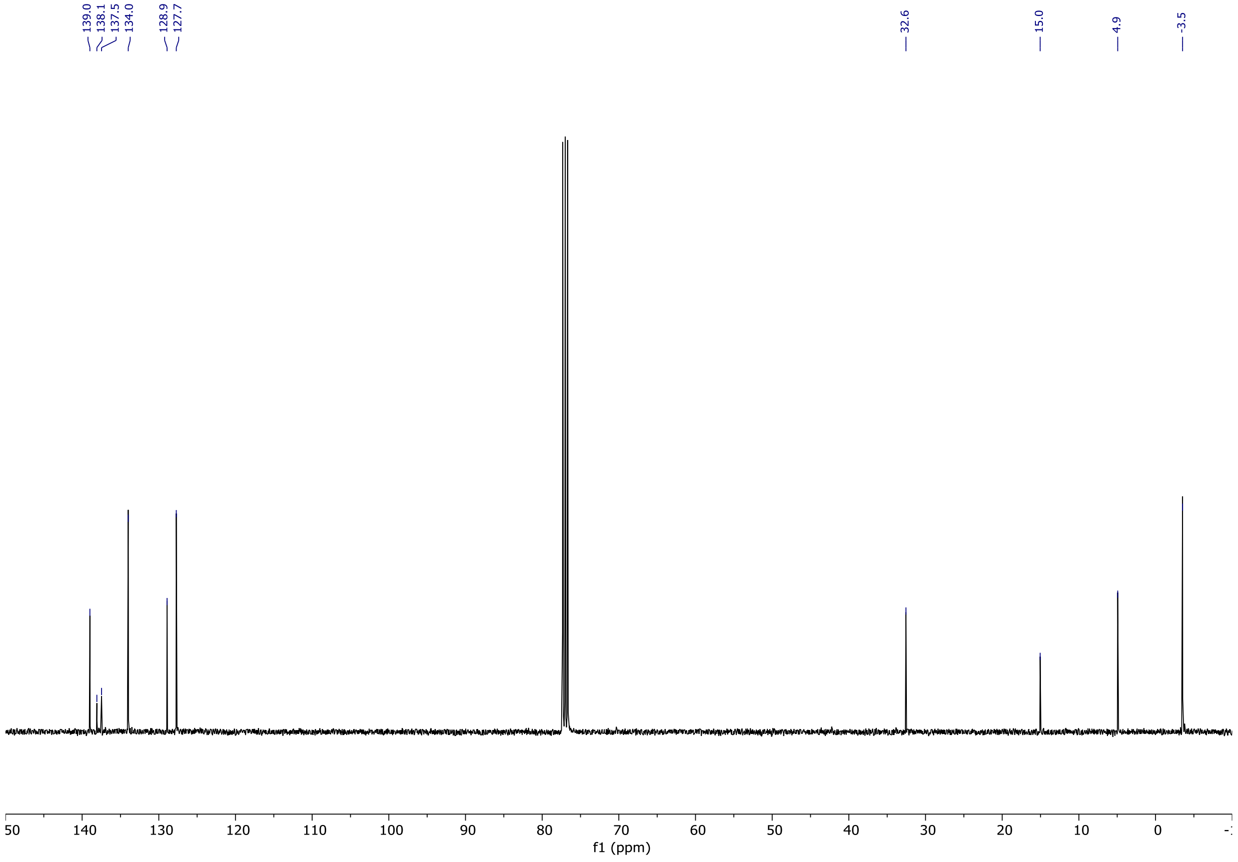
139.0
138.1
137.5
134.0
128.9
127.7
32.6
15.0
4.9
-3.5
140 130 120 110 100 90 80 70 60 50 40 30 20 10 0
f1 (ppm)

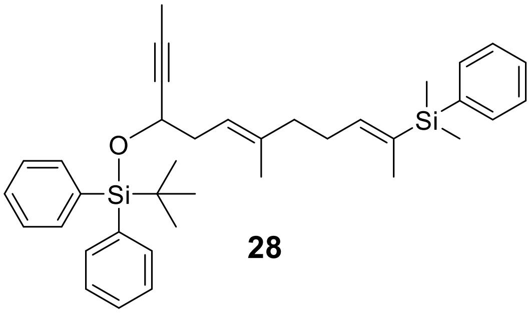

28

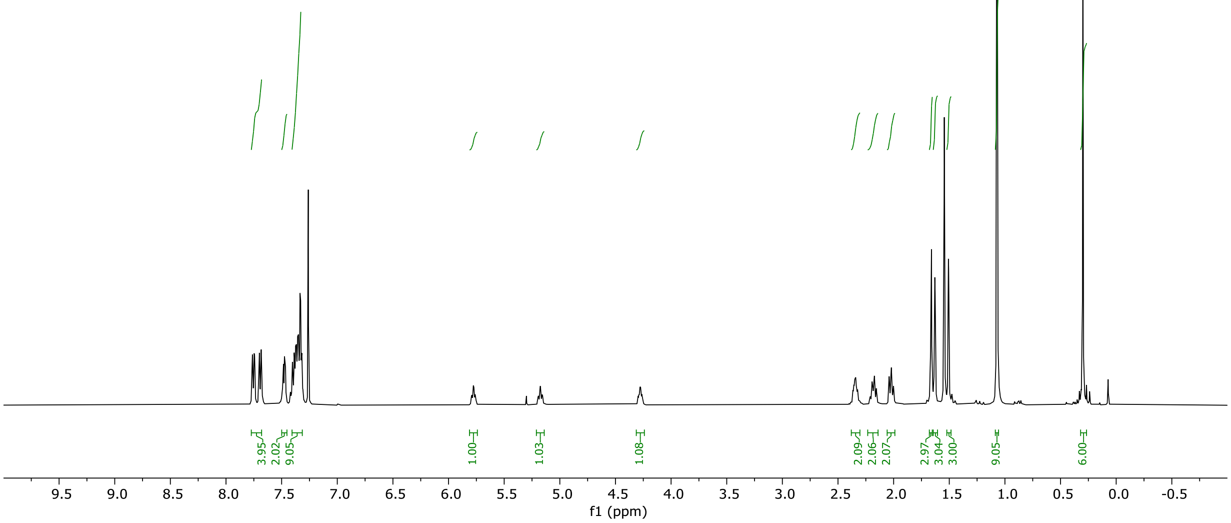
3.95
2.02
9.05
1.00
1.03
1.08
2.09
2.06
2.07
2.97
3.04
3.00
9.05
6.00
f1 (ppm)

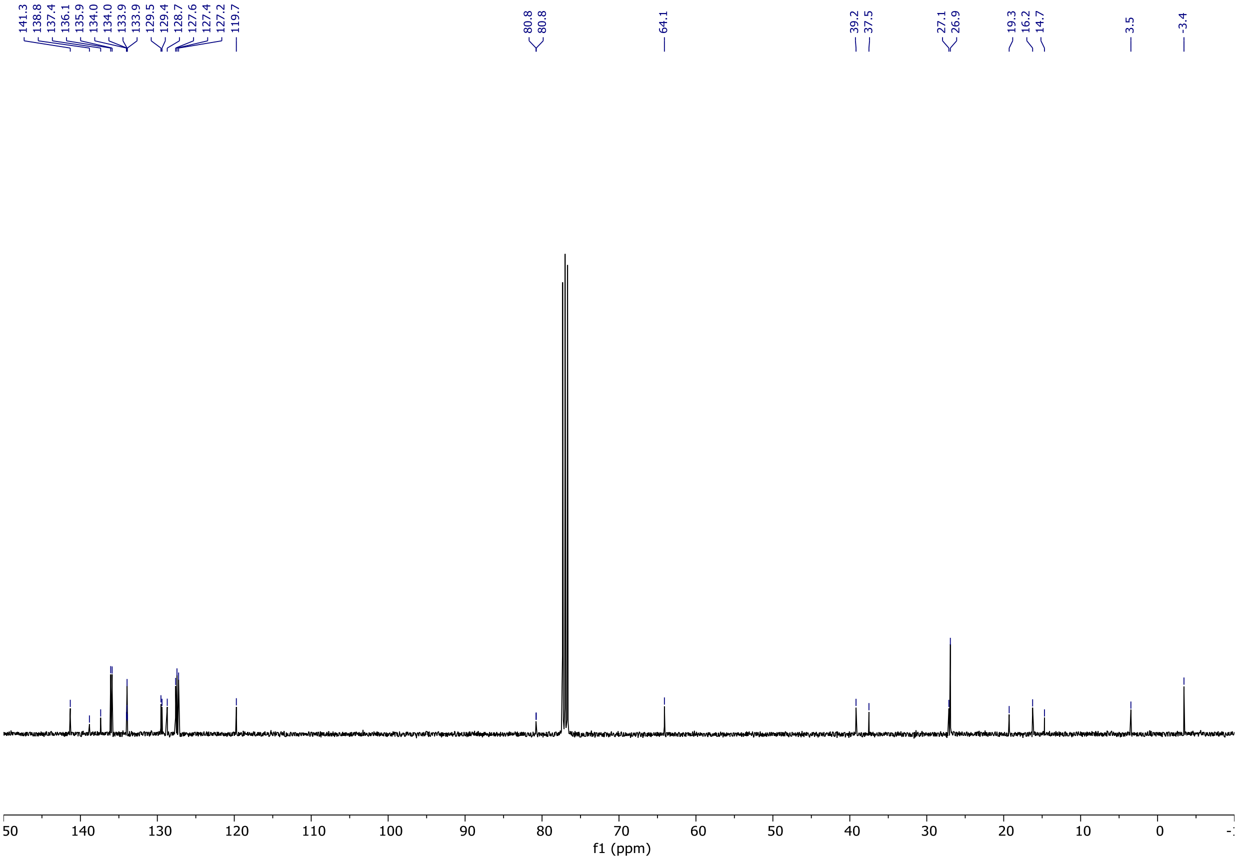
141.3
138.8
137.4
136.1
135.9
134.0
134.0
133.9
133.9
129.5
129.4
128.7
127.6
127.4
127.2
119.7
80.8
80.8
64.1
39.2
37.5
27.1
26.9
19.3
16.2
14.7
3.5
-3.4
f1 (ppm)

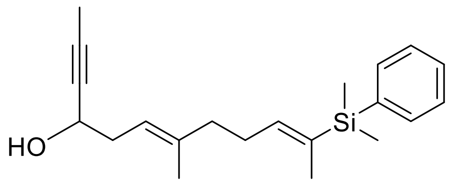
HO
Si

29

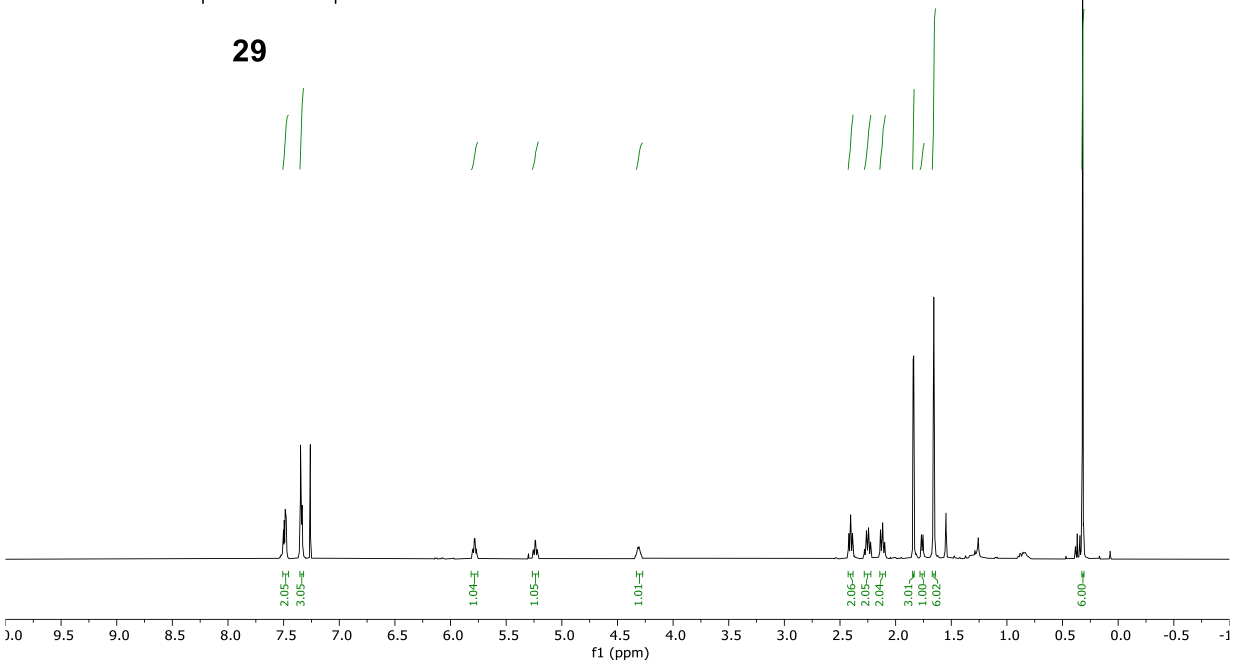
2.05
3.05
1.04
1.05
1.01
2.06
2.05
2.04
3.01
1.00
6.02
6.00
9.5 9.0 8.5 8.0 7.5 7.0 6.5 6.0 5.5 5.0 4.5 4.0 3.5 3.0 2.5 2.0 1.5 1.0 0.5 0.0 -0.5
f1 (ppm)

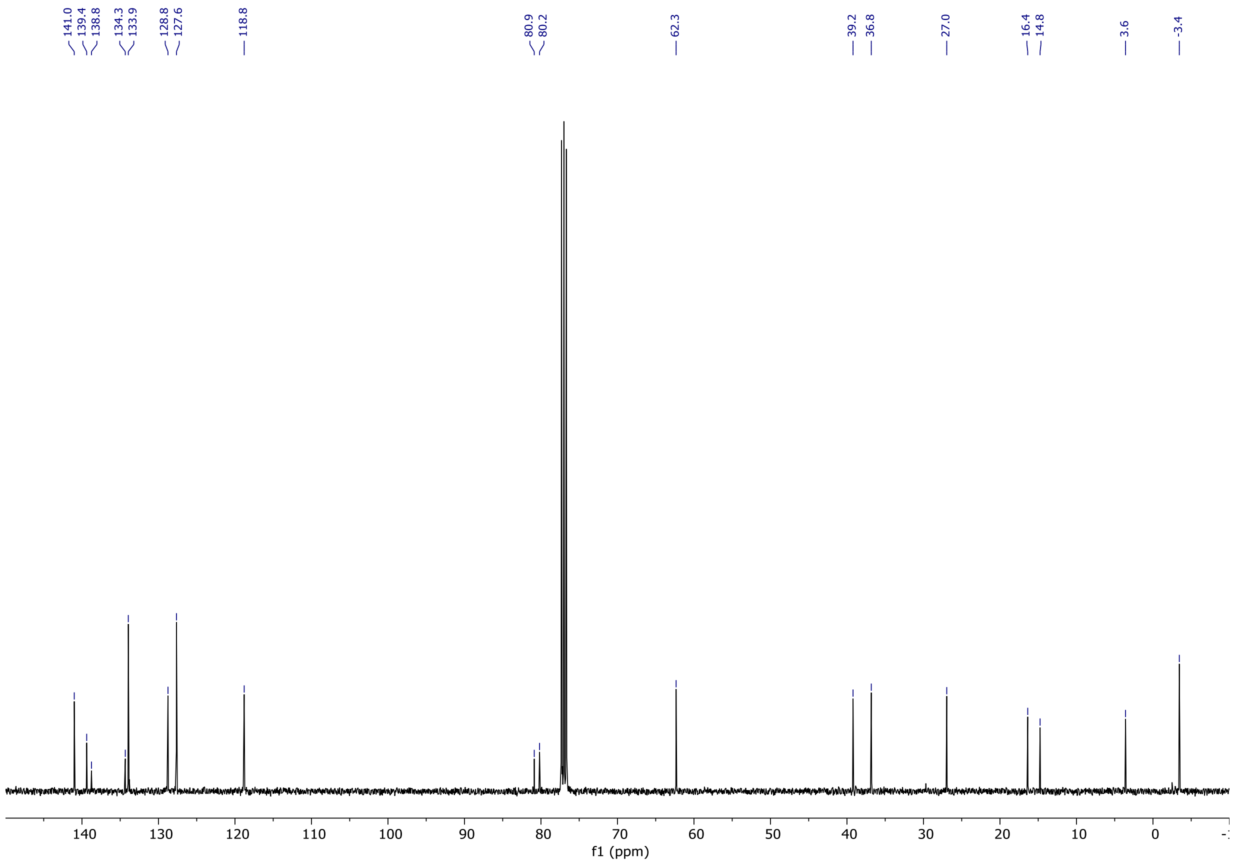
141.0
139.4
138.8
134.3
133.9
128.8
127.6
118.8
80.9
80.2
62.3
39.2
36.8
27.0
16.4
14.8
3.6
-3.4
140 130 120 110 100 90 80 70 60 50 40 30 20 10 0
f1 (ppm)

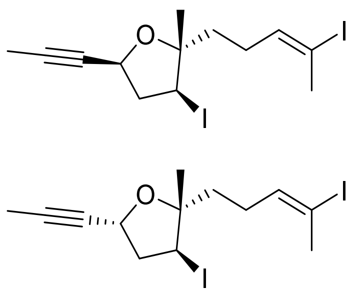

30

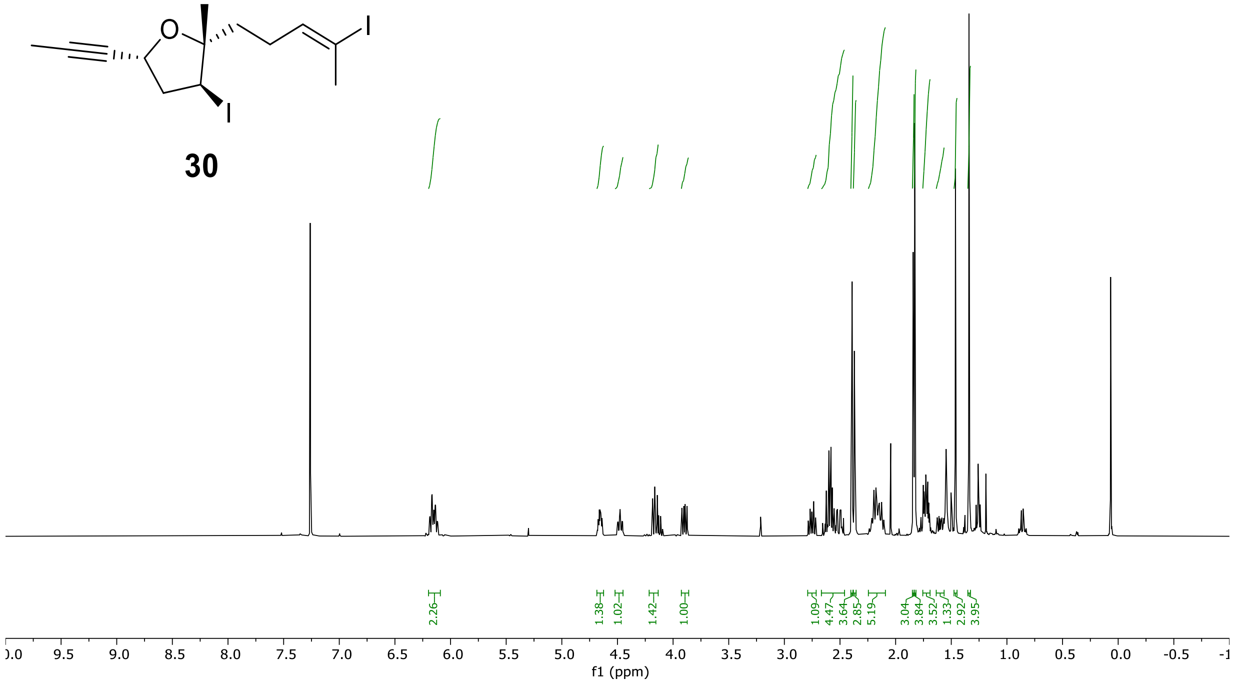

2.26
1.38
1.02
1.42
1.00
1.09
4.47
3.64
2.85
5.19
3.04
3.84
3.52
1.33
2.92
3.95
f1 (ppm)

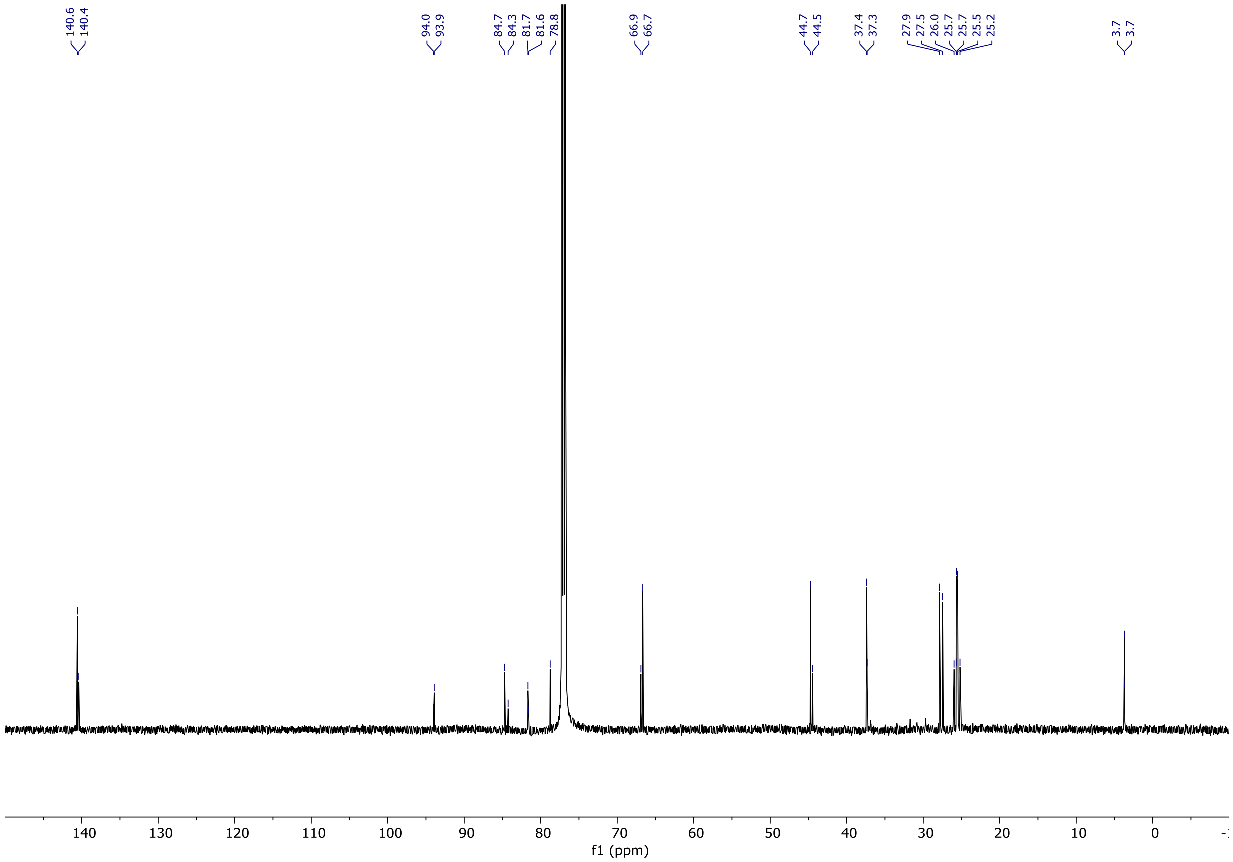

140.6
140.4
94.0
93.9
84.7
84.3
81.7
81.6
78.8
66.9
66.7
44.7
44.5
37.4
37.3
27.9
27.5
26.0
25.7
25.7
25.5
25.2
3.7
3.7
f1 (ppm)

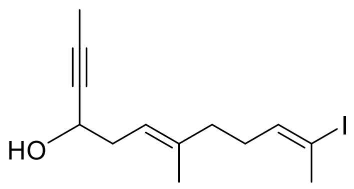
HO
I

31

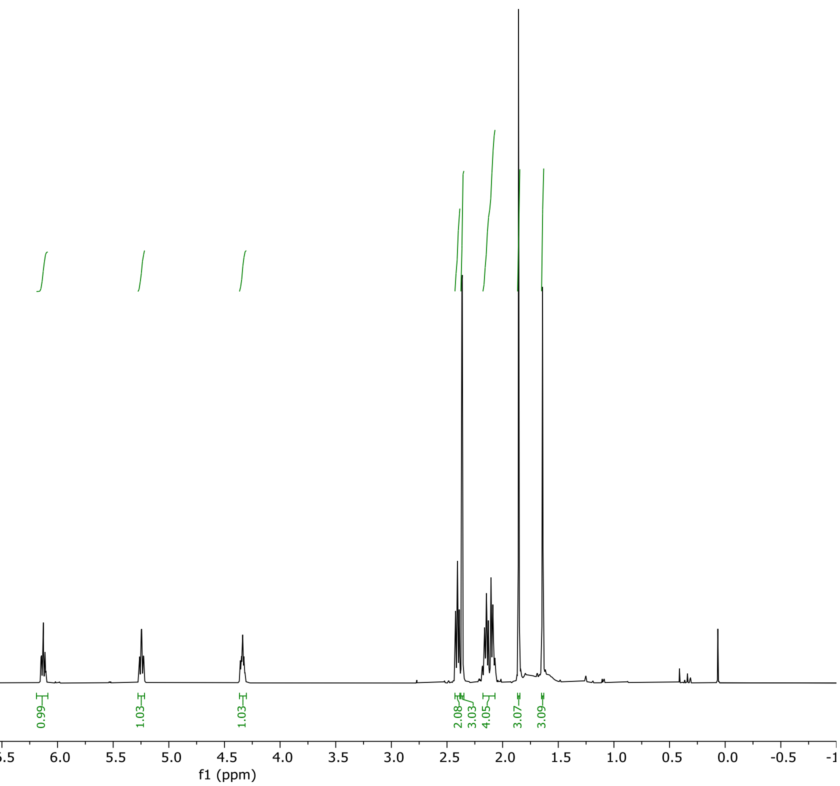
0.99
1.03
1.03
2.08
3.03
4.05
3.07
3.09
f1 (ppm)

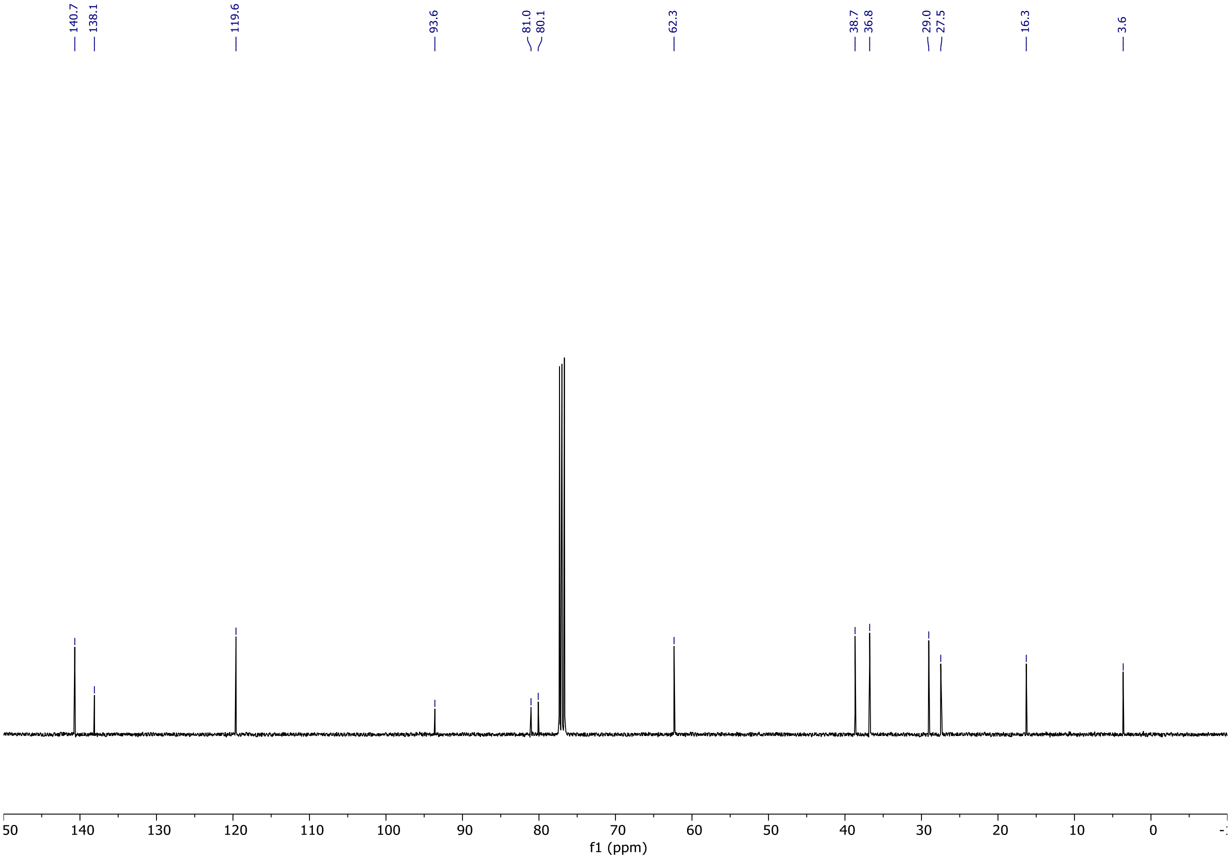
140.7
138.1
119.6
93.6
81.0
80.1
62.3
38.7
36.8
29.0
27.5
16.3
3.6
f1 (ppm)

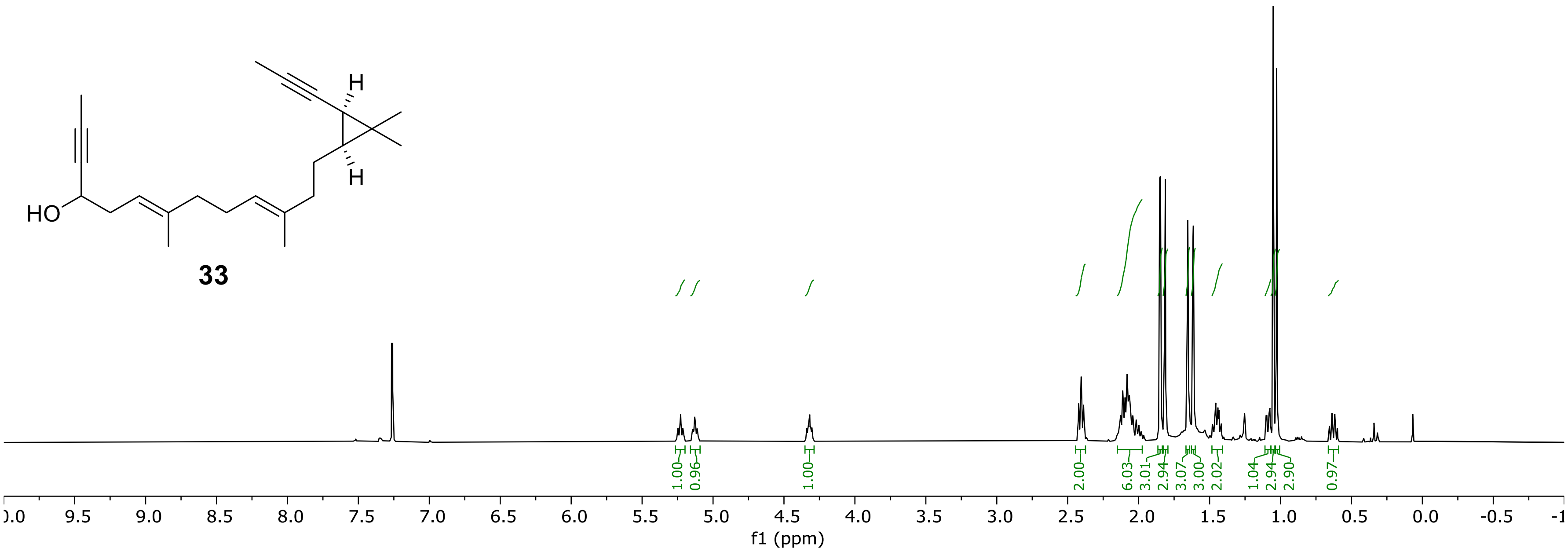
HO
H
H
1.00
0.96
1.00
2.00
6.03
3.01
2.94
3.07
3.00
2.02
1.04
2.94
2.90
0.97
f1 (ppm)

33

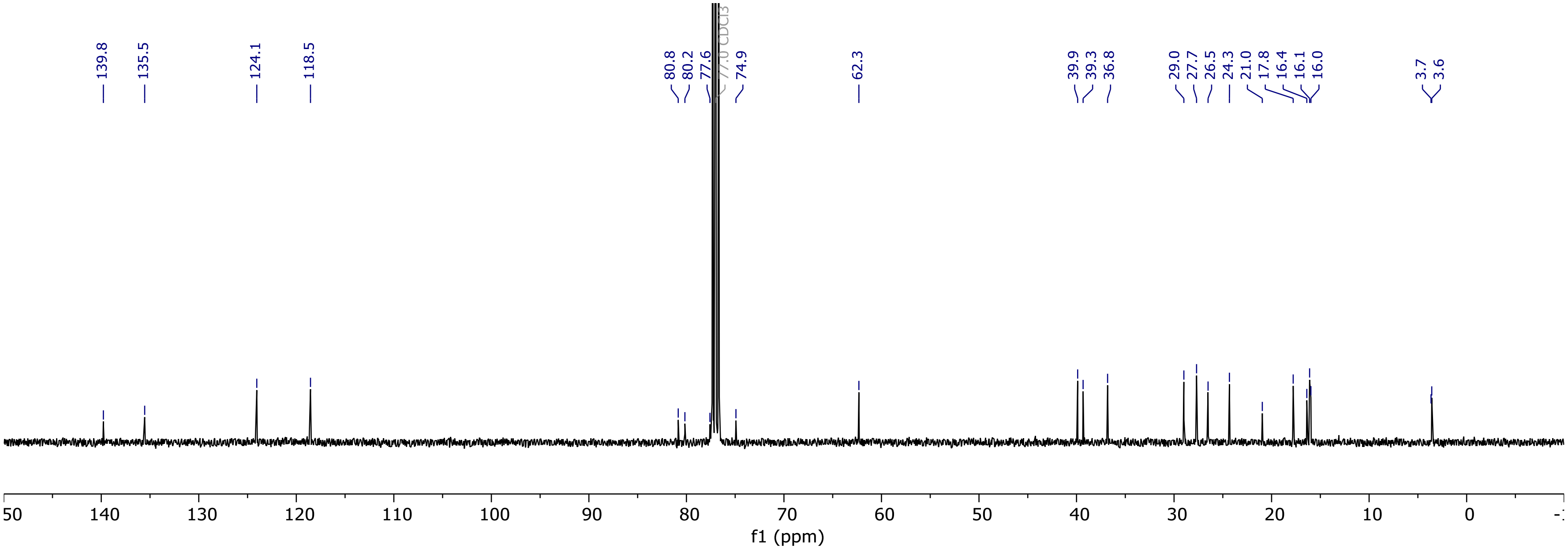
139.8
135.5
124.1
118.5
80.8
80.2
77.6
77.0 CDCl3
74.9
62.3
39.9
39.3
36.8
29.0
27.7
26.5
24.3
21.0
17.8
16.4
16.1
16.0
3.7
3.6
f1 (ppm)

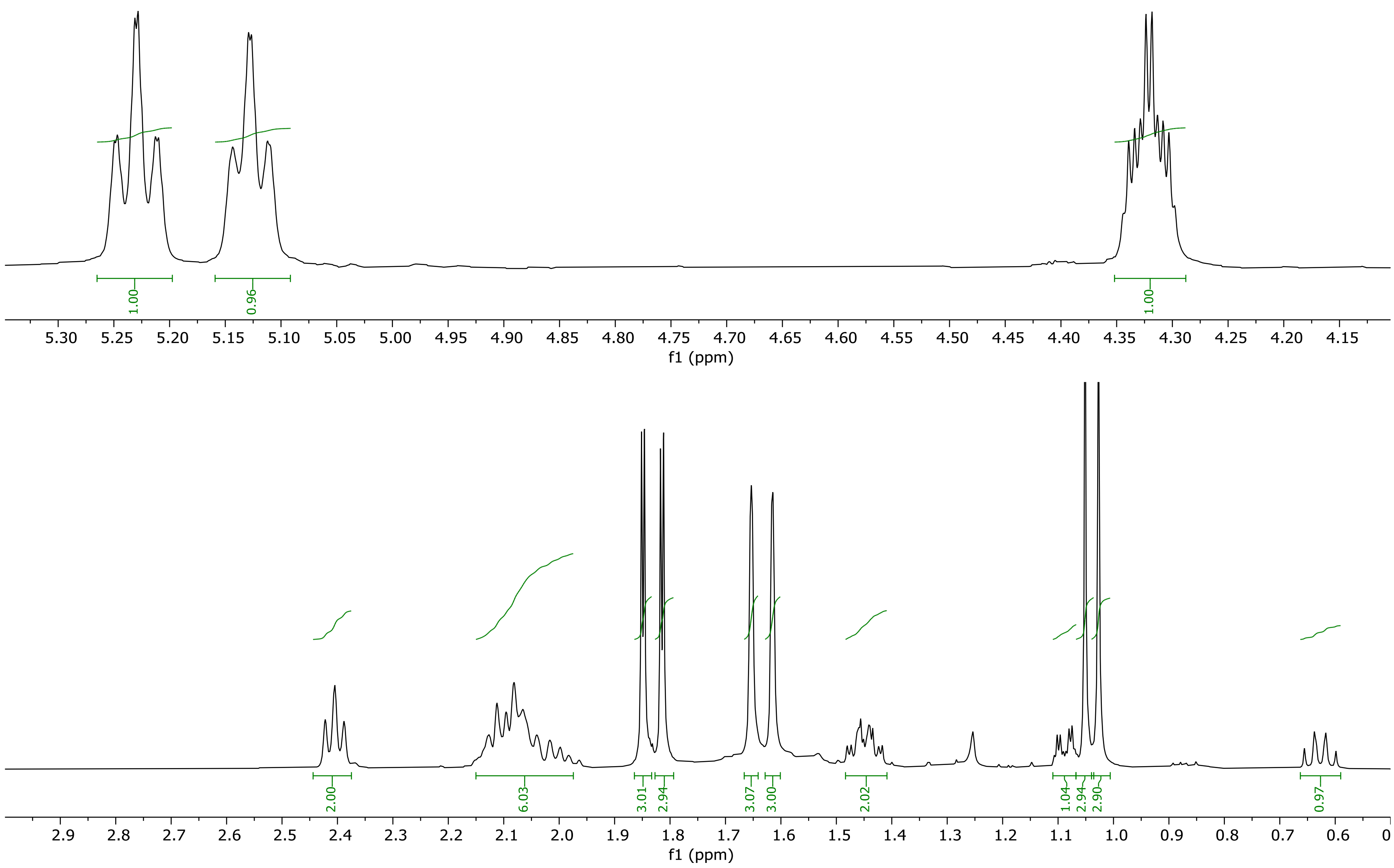

1.00
0.96
1.00
5.30 5.25 5.20 5.15 5.10 5.05 5.00 4.95 4.90 4.85 4.80 4.75 4.70 4.65 4.60 4.55 4.50 4.45 4.40 4.35 4.30 4.25 4.20 4.15
f1 (ppm)
2.00
6.03
3.01
2.94
3.07
3.00
2.02
1.04
2.94
2.90
0.97
2.9 2.8 2.7 2.6 2.5 2.4 2.3 2.2 2.1 2.0 1.9 1.8 1.7 1.6 1.5 1.4 1.3 1.2 1.1 1.0 0.9 0.8 0.7 0.6 0
f1 (ppm)

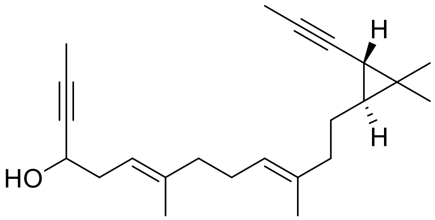

39

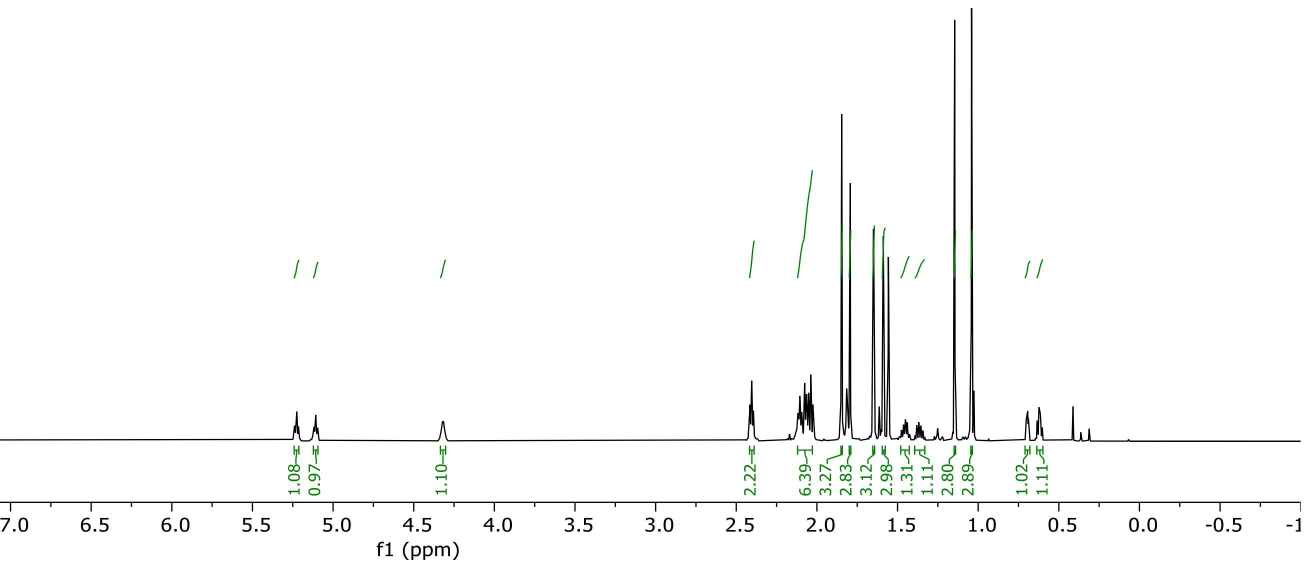

1.08
0.97
1.10
2.22
6.39
3.27
2.83
3.12
2.98
1.31
1.11
2.80
2.89
1.02
1.11
f1 (ppm)

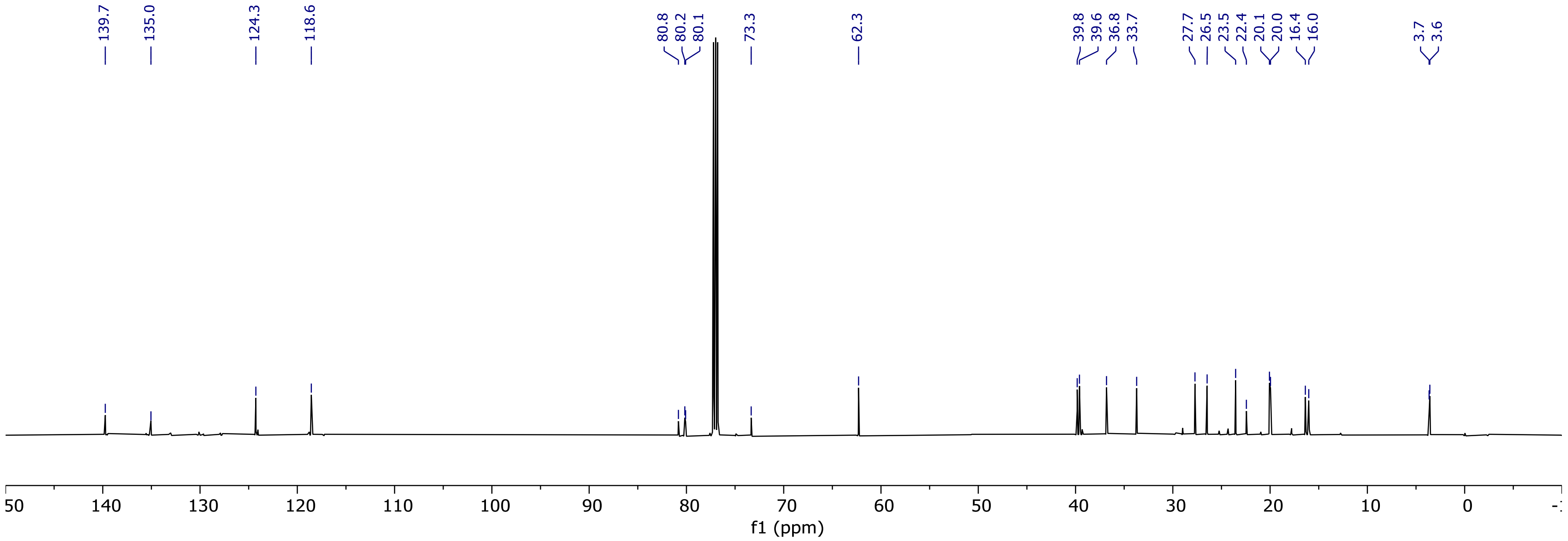

139.7
135.0
124.3
118.6
80.8
80.2
80.1
73.3
62.3
39.8
39.6
36.8
33.7
27.7
26.5
23.5
22.4
20.1
20.0
16.4
16.0
3.7
3.6
f1 (ppm)

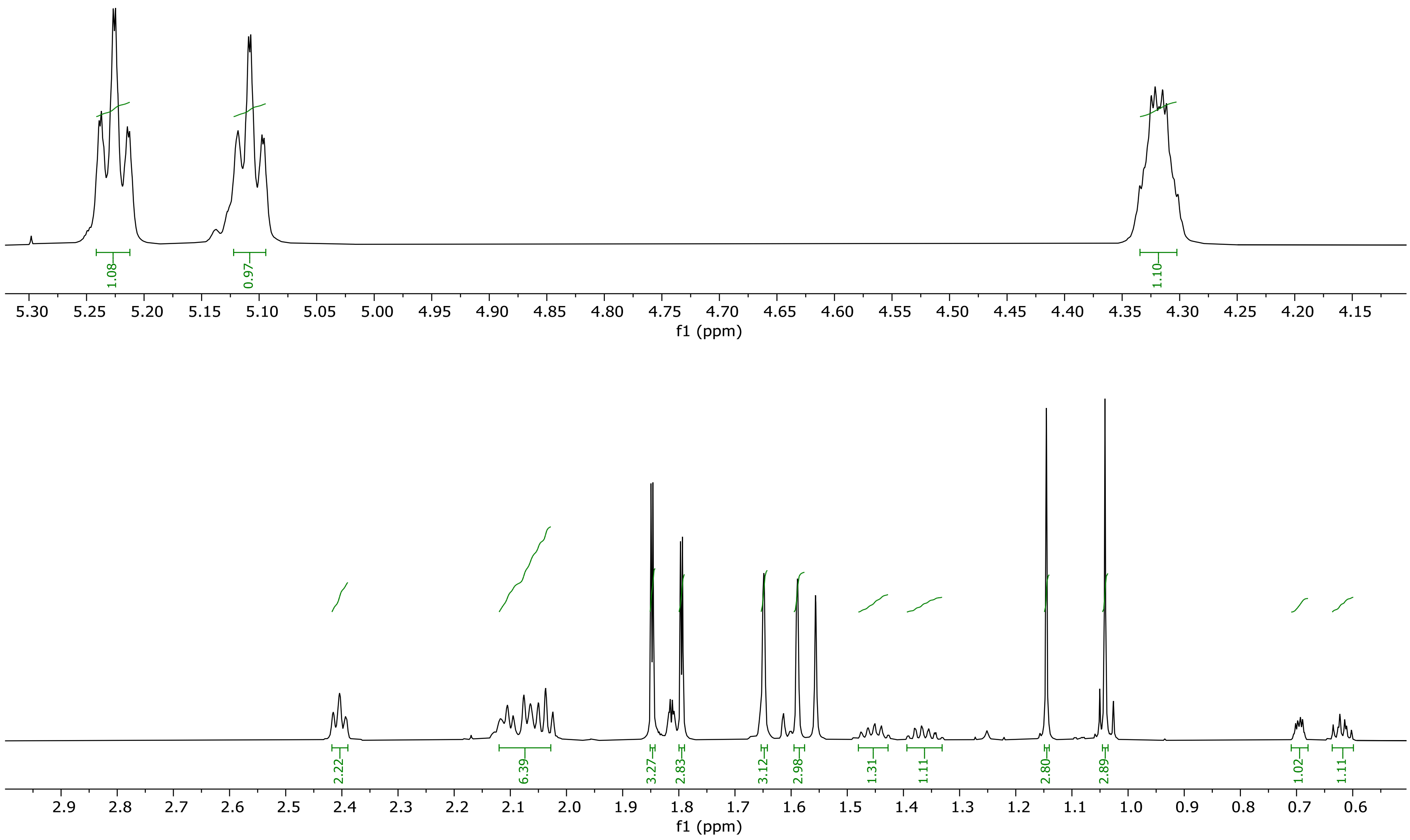

1.08
0.97
1.10
5.30 5.25 5.20 5.15 5.10 5.05 5.00 4.95 4.90 4.85 4.80 4.75 4.70 4.65 4.60 4.55 4.50 4.45 4.40 4.35 4.30 4.25 4.20 4.15
f1 (ppm)
2.22
6.39
3.27
2.83
3.12
2.98
1.31
1.11
2.80
2.89
1.02
1.11
2.9 2.8 2.7 2.6 2.5 2.4 2.3 2.2 2.1 2.0 1.9 1.8 1.7 1.6 1.5 1.4 1.3 1.2 1.1 1.0 0.9 0.8 0.7 0.6
f1 (ppm)

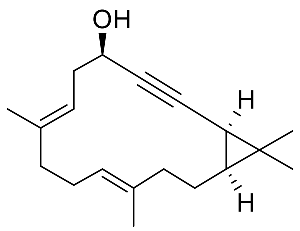
OH
H
H

34

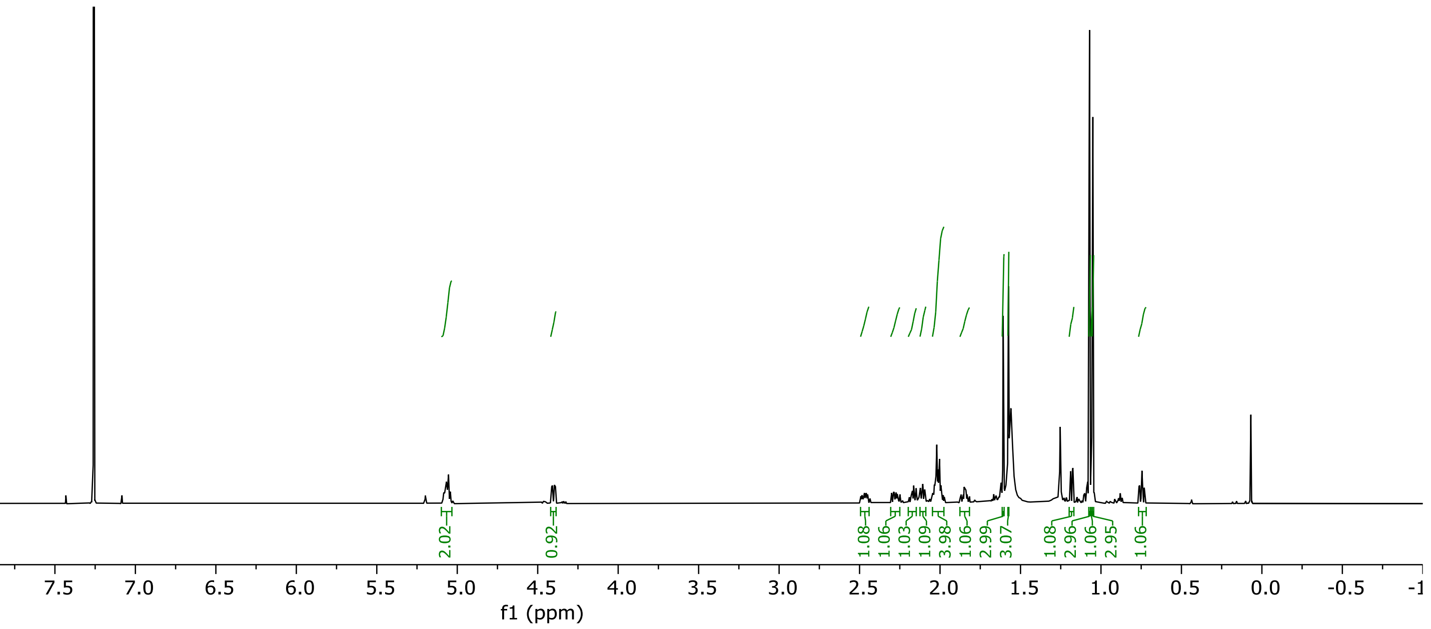
2.02
0.92
1.08
1.06
1.03
1.09
3.98
1.06
2.99
3.07
1.08
2.96
1.06
2.95
1.06
9.5 9.0 8.5 8.0 7.5 7.0 6.5 6.0 5.5 5.0 4.5 4.0 3.5 3.0 2.5 2.0 1.5 1.0 0.5 0.0 -0.5
f1 (ppm)

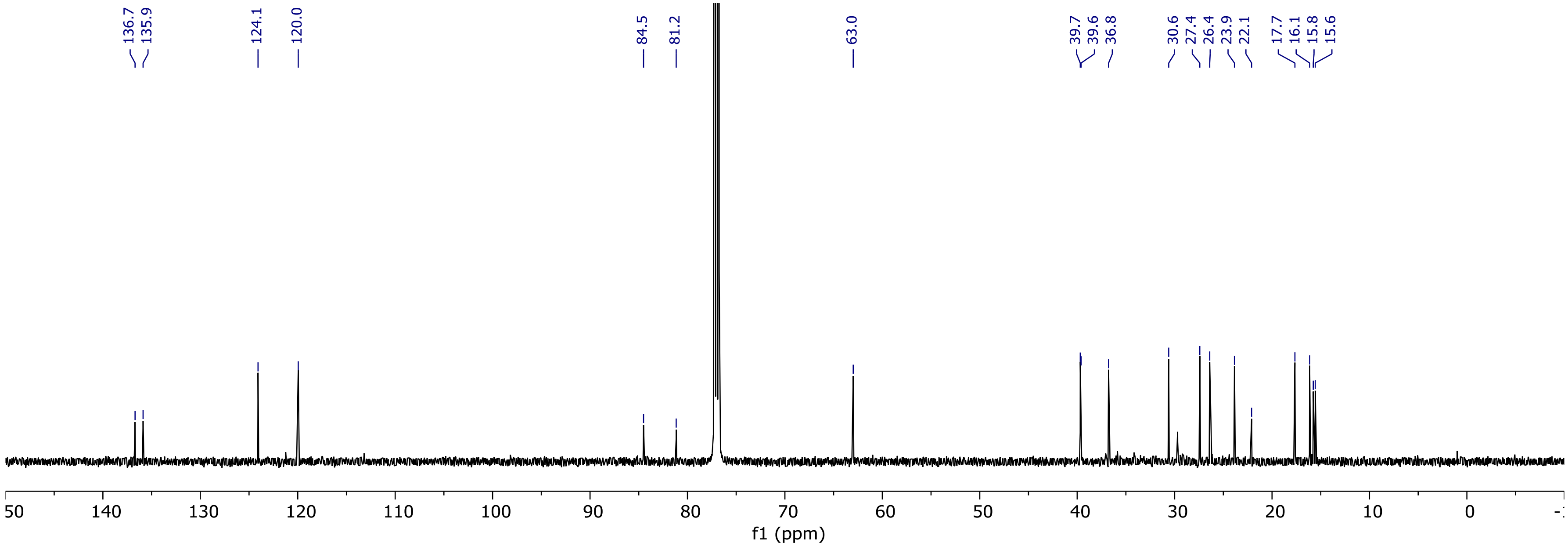
136.7
135.9
124.1
120.0
84.5
81.2
63.0
39.7
39.6
36.8
30.6
27.4
26.4
23.9
22.1
17.7
16.1
15.8
15.6
140 130 120 110 100 90 80 70 60 50 40 30 20 10 0
f1 (ppm)

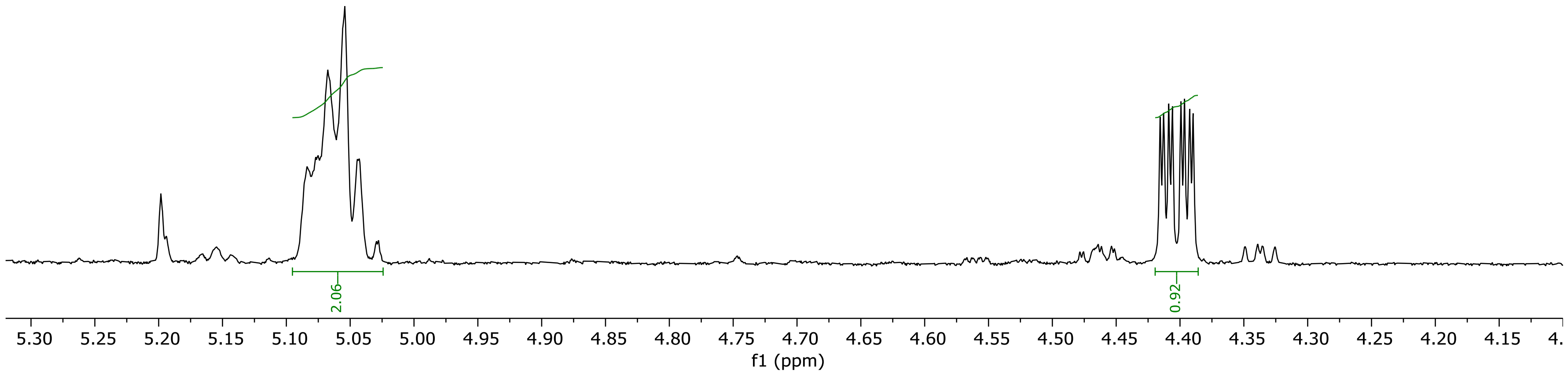
2.06
0.92
5.30 5.25 5.20 5.15 5.10 5.05 5.00 4.95 4.90 4.85 4.80 4.75 4.70 4.65 4.60 4.55 4.50 4.45 4.40 4.35 4.30 4.25 4.20 4.15 4.
f1 (ppm)

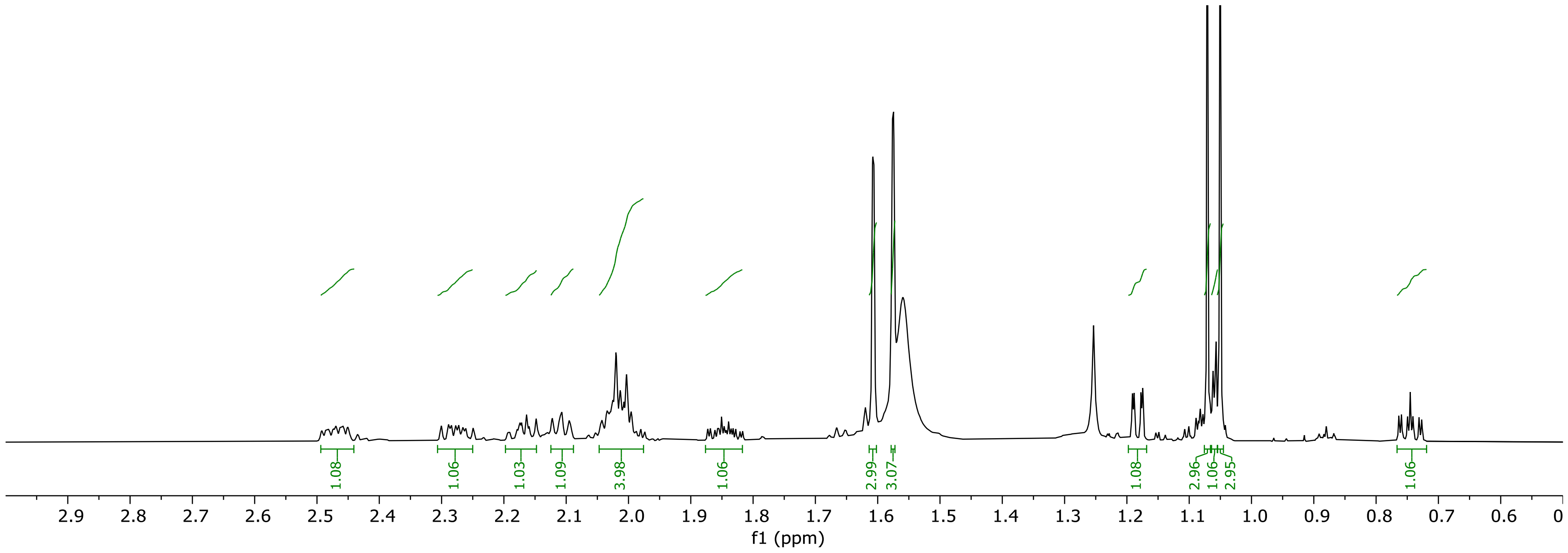
1.08
1.06
1.03
1.09
3.98
1.06
2.99
3.07
1.08
2.96
1.06
2.95
1.06
2.9 2.8 2.7 2.6 2.5 2.4 2.3 2.2 2.1 2.0 1.9 1.8 1.7 1.6 1.5 1.4 1.3 1.2 1.1 1.0 0.9 0.8 0.7 0.6 0
f1 (ppm)

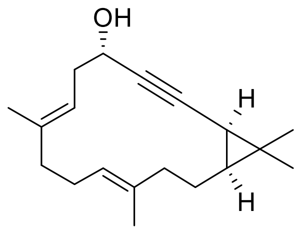
OH
H
H

35

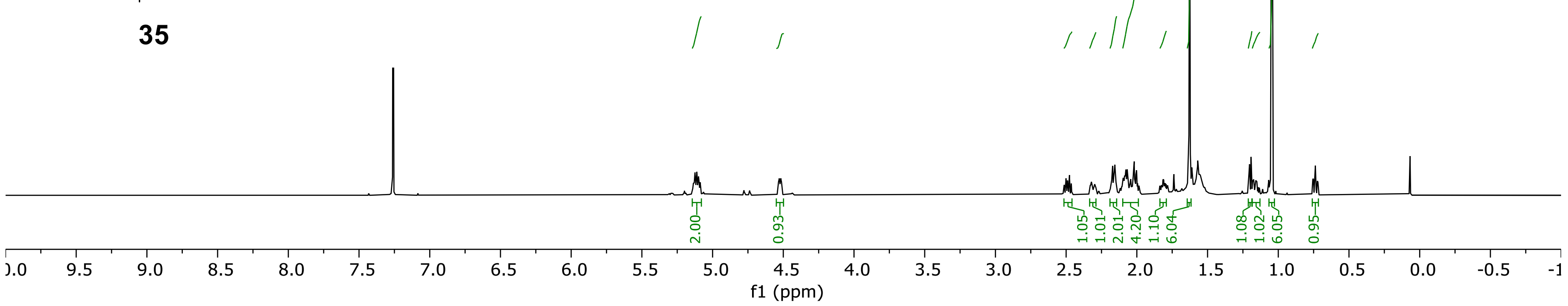
2.00
0.93
1.05
1.01
2.01
4.20
1.10
6.04
1.08
1.02
6.05
0.95
f1 (ppm)

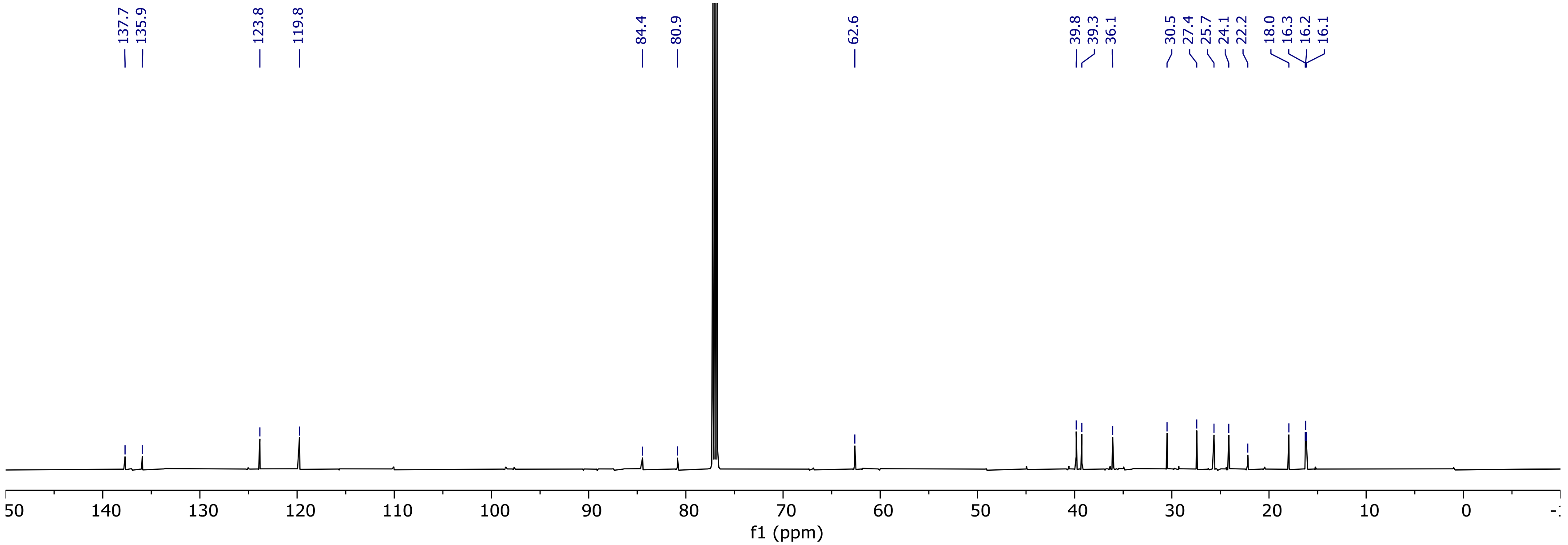
137.7
135.9
123.8
119.8
84.4
80.9
62.6
39.8
39.3
36.1
30.5
27.4
25.7
24.1
22.2
18.0
16.3
16.2
16.1
f1 (ppm)

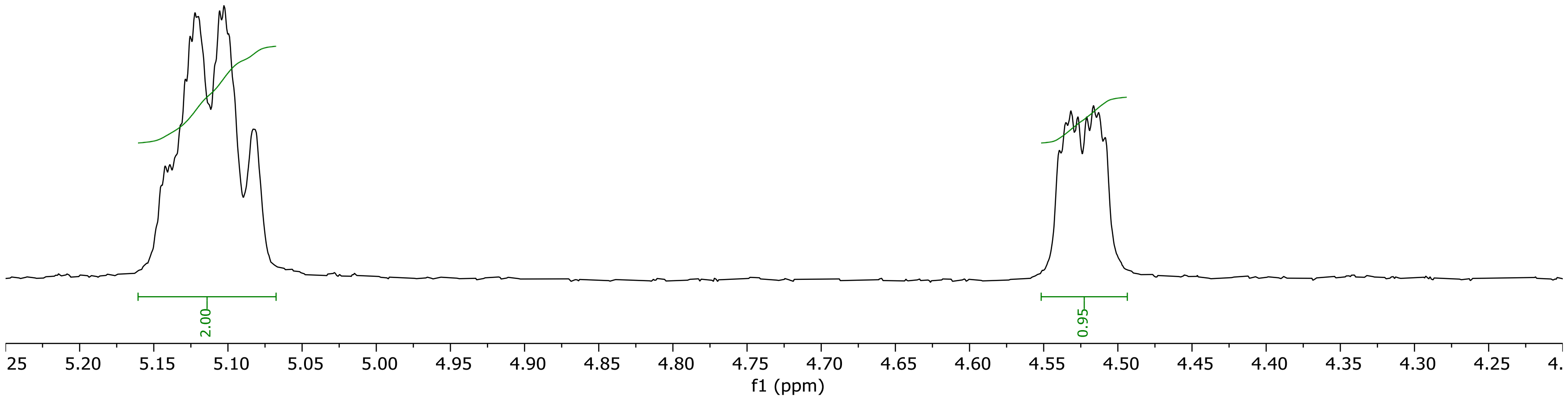
2.00
0.95
5.25
5.20
5.15
5.10
5.05
5.00
4.95
4.90
4.85
4.80
4.75
4.70
4.65
4.60
4.55
4.50
4.45
4.40
4.35
4.30
4.25
4.
f1 (ppm)

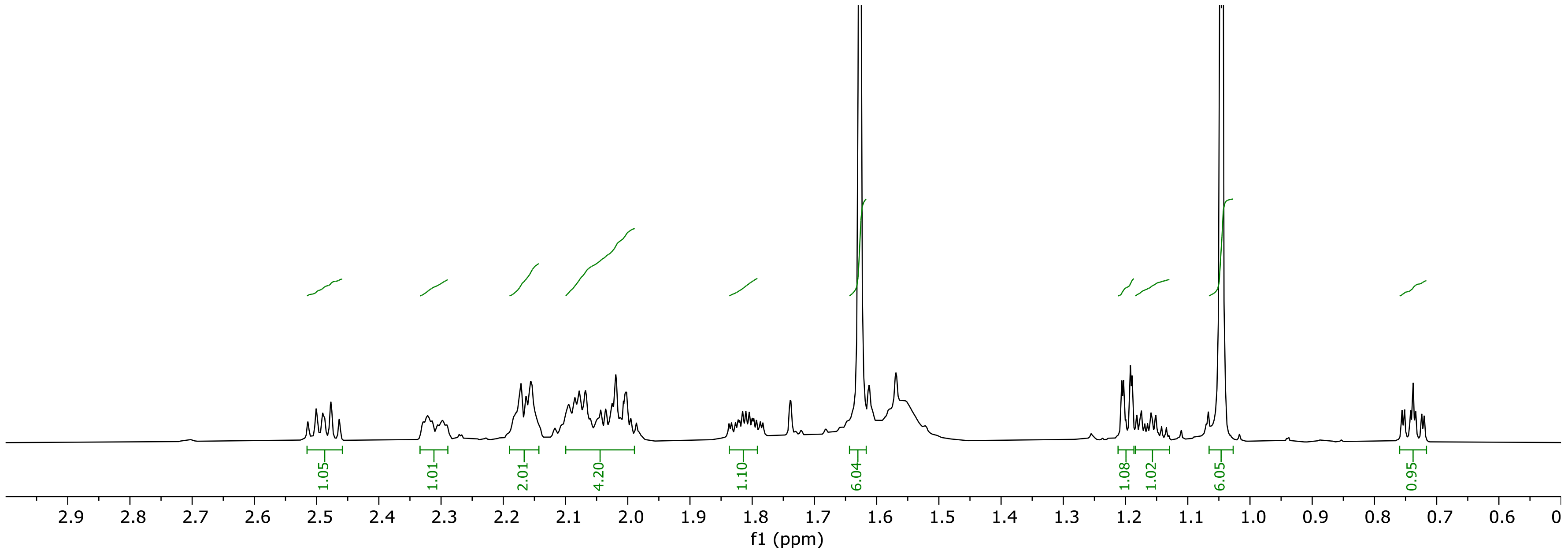
1.05
1.01
2.01
4.20
1.10
6.04
1.08
1.02
6.05
0.95
2.9
2.8
2.7
2.6
2.5
2.4
2.3
2.2
2.1
2.0
1.9
1.8
1.7
1.6
1.5
1.4
1.3
1.2
1.1
1.0
0.9
0.8
0.7
0.6
0
f1 (ppm)

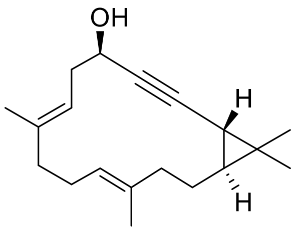
OH
H
H

40

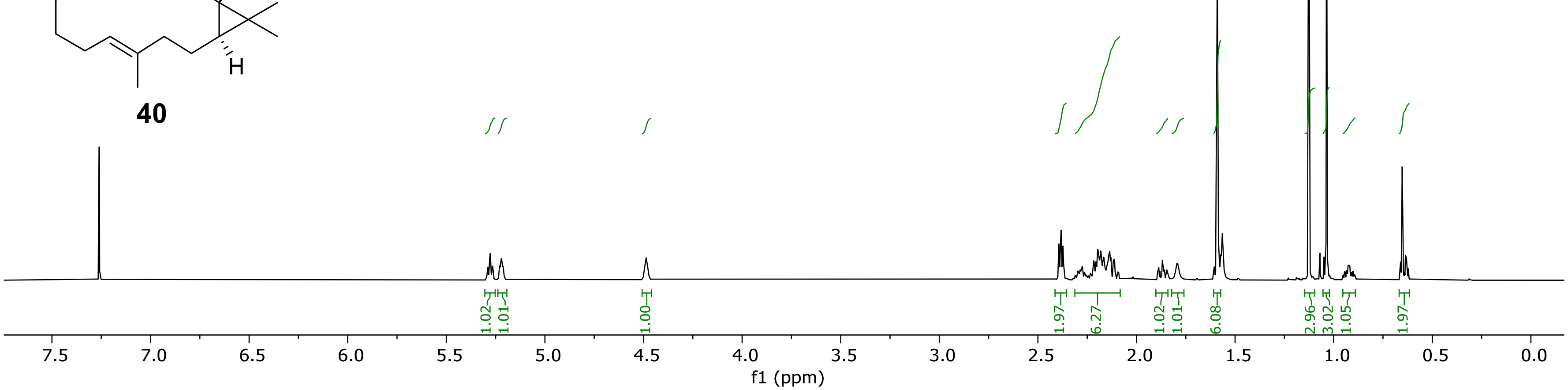
1.02
1.01
1.00
1.97
6.27
1.02
1.01
6.08
2.96
3.02
1.05
1.97
7.5
7.0
6.5
6.0
5.5
5.0
4.5
4.0
3.5
3.0
2.5
2.0
1.5
1.0
0.5
0.0
f1 (ppm)

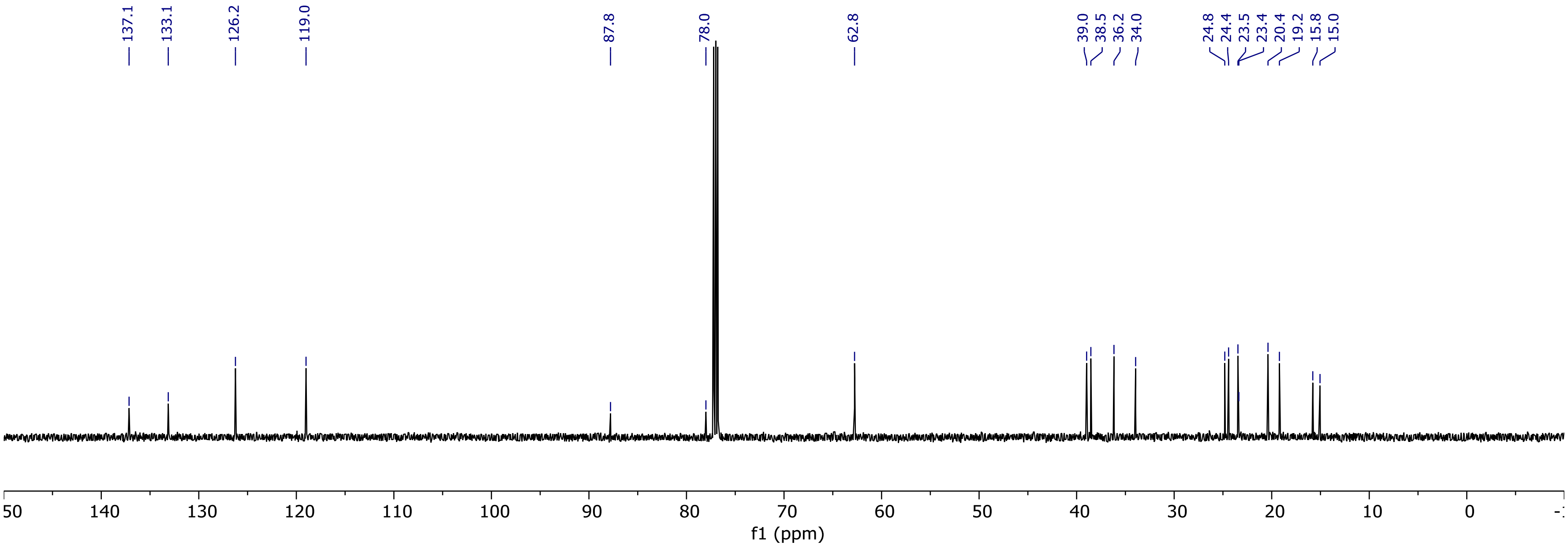
137.1
133.1
126.2
119.0
87.8
78.0
62.8
39.0
38.5
36.2
34.0
24.8
24.4
23.5
23.4
20.4
19.2
15.8
15.0
140
130
120
110
100
90
80
70
60
50
40
30
20
10
0
f1 (ppm)

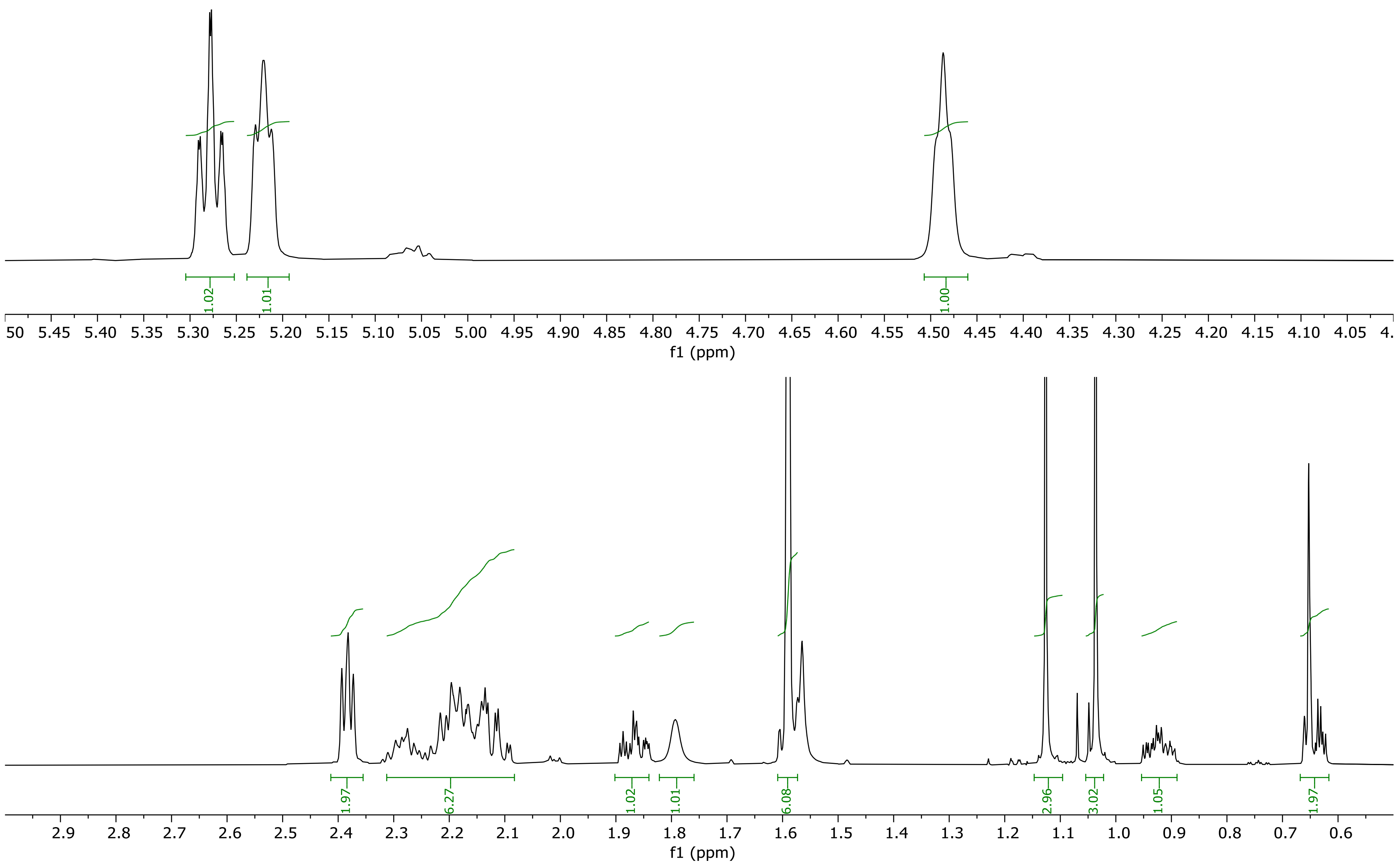

1.02
1.01
1.00
50 5.45 5.40 5.35 5.30 5.25 5.20 5.15 5.10 5.05 5.00 4.95 4.90 4.85 4.80 4.75 4.70 4.65 4.60 4.55 4.50 4.45 4.40 4.35 4.30 4.25 4.20 4.15 4.10 4.05 4.
f1 (ppm)
1.97
6.27
1.02
1.01
6.08
2.96
3.02
1.05
1.97
2.9 2.8 2.7 2.6 2.5 2.4 2.3 2.2 2.1 2.0 1.9 1.8 1.7 1.6 1.5 1.4 1.3 1.2 1.1 1.0 0.9 0.8 0.7 0.6
f1 (ppm)

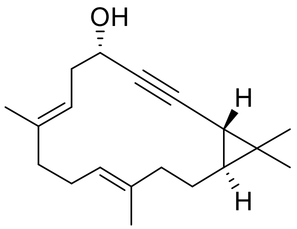
OH
H
H

41

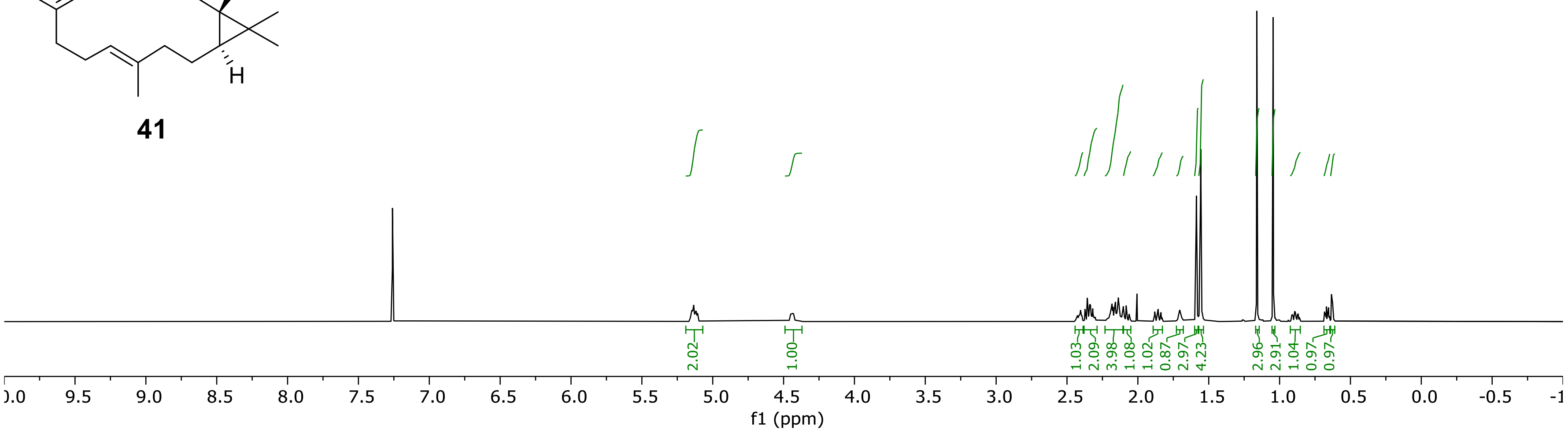
2.02
1.00
1.03
2.09
3.98
1.08
1.02
0.87
2.97
4.23
2.96
2.91
1.04
0.97
0.97
f1 (ppm)

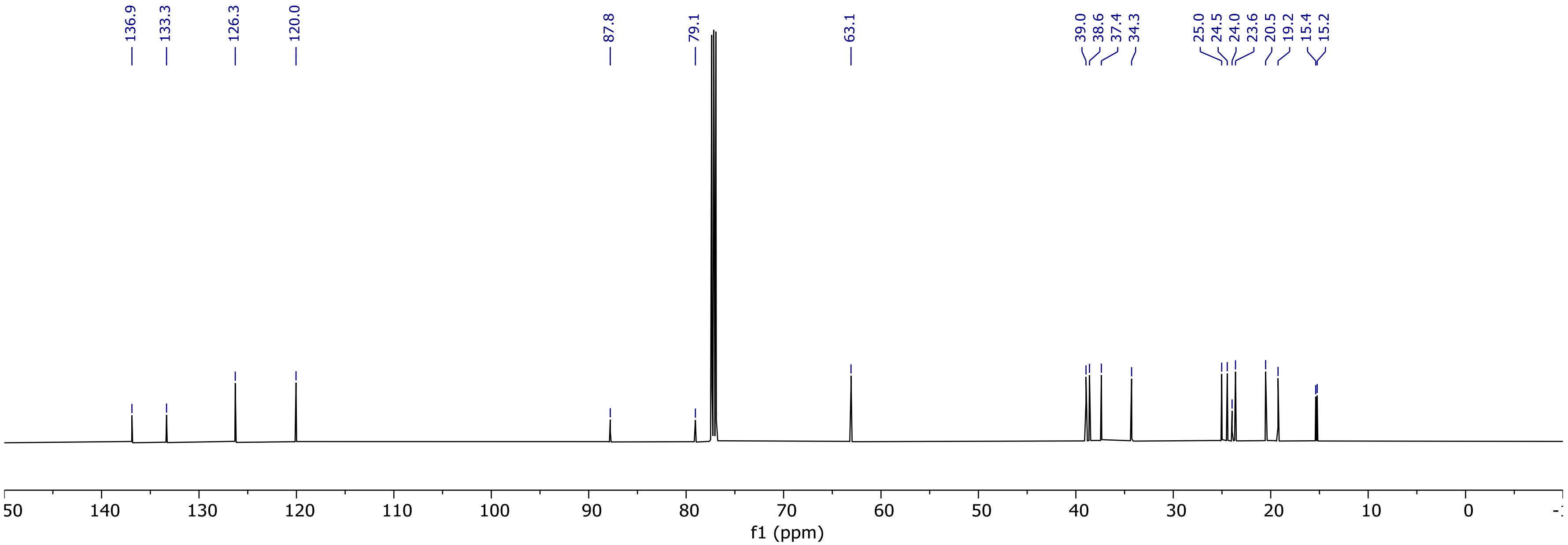
136.9
133.3
126.3
120.0
87.8
79.1
63.1
39.0
38.6
37.4
34.3
25.0
24.5
24.0
23.6
20.5
19.2
15.4
15.2
f1 (ppm)

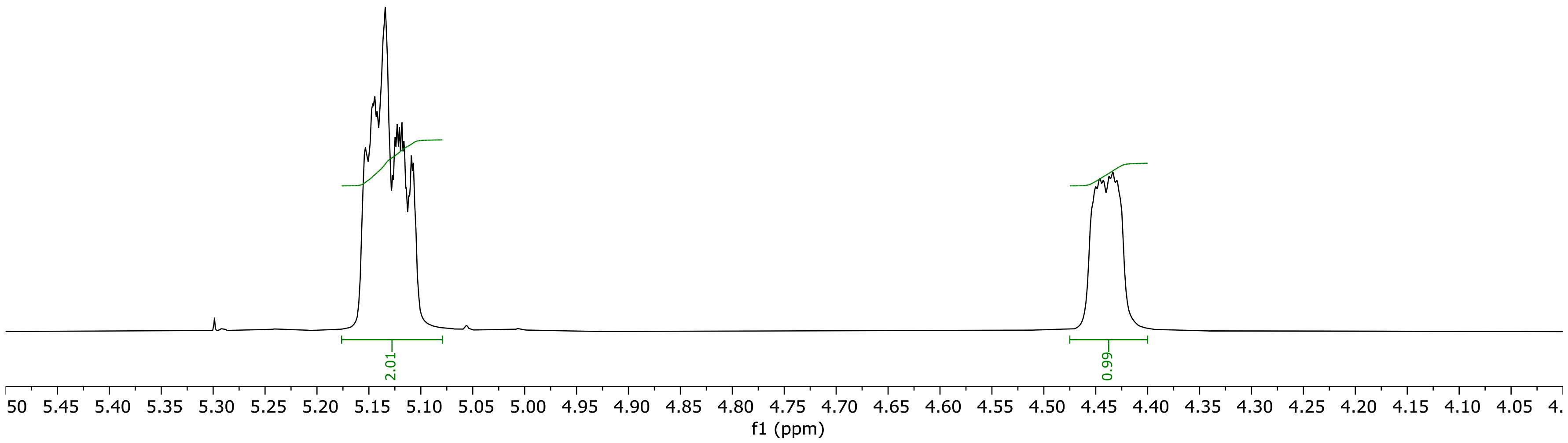
2.01
0.99
50 5.45 5.40 5.35 5.30 5.25 5.20 5.15 5.10 5.05 5.00 4.95 4.90 4.85 4.80 4.75 4.70 4.65 4.60 4.55 4.50 4.45 4.40 4.35 4.30 4.25 4.20 4.15 4.10 4.05 4.
f1 (ppm)

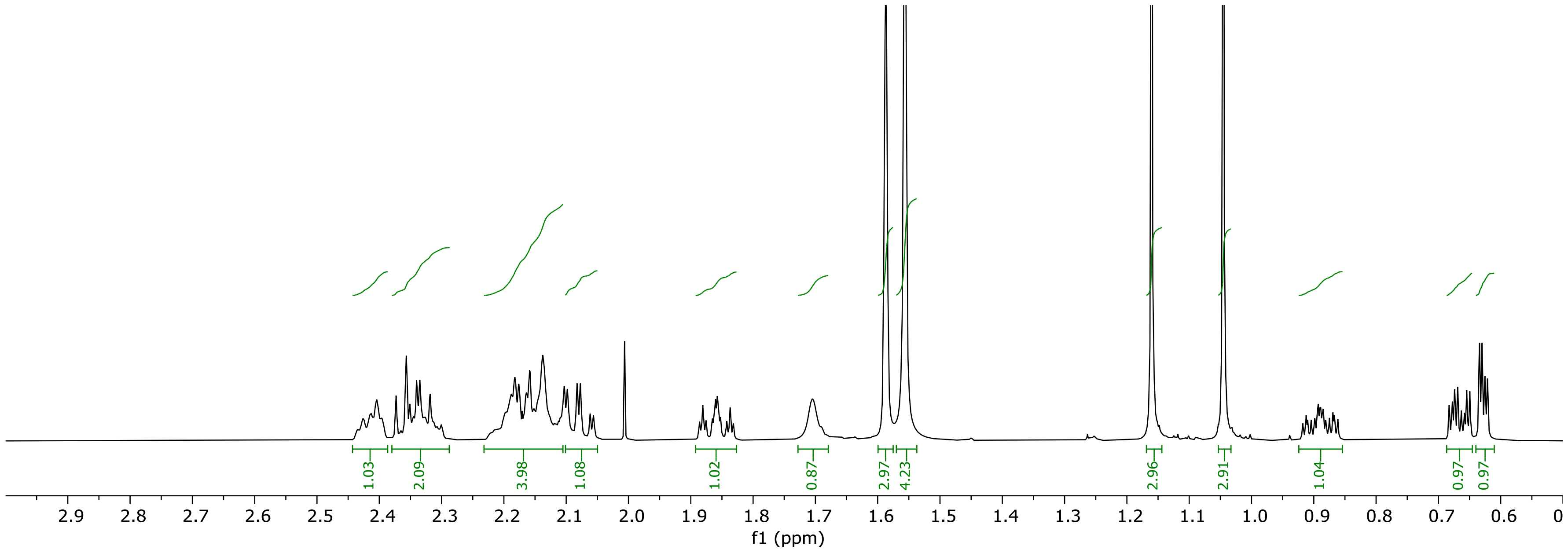
1.03
2.09
3.98
1.08
1.02
0.87
2.97
4.23
2.96
2.91
1.04
0.97
0.97
2.9 2.8 2.7 2.6 2.5 2.4 2.3 2.2 2.1 2.0 1.9 1.8 1.7 1.6 1.5 1.4 1.3 1.2 1.1 1.0 0.9 0.8 0.7 0.6 0
f1 (ppm)

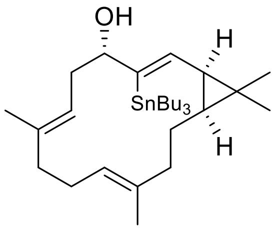
OH
H
SnBu3
H

36

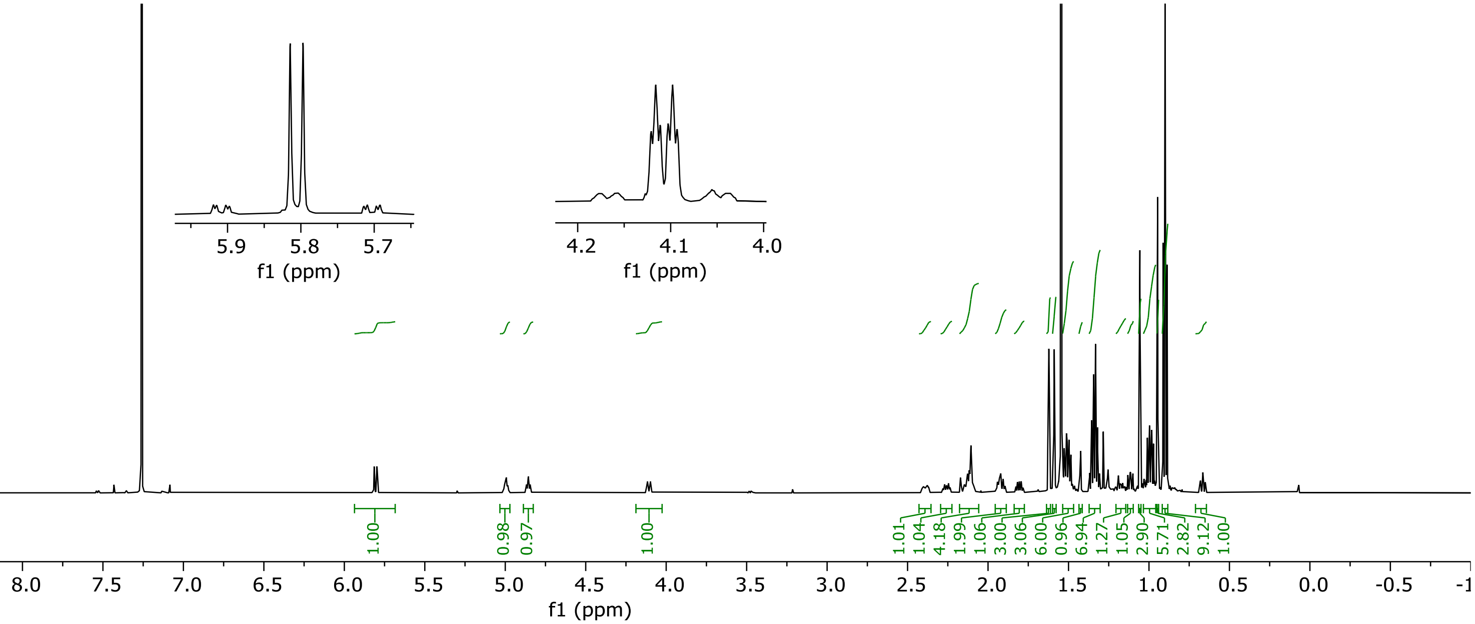
5.9
5.8
5.7
f1 (ppm)
4.2
4.1
4.0
f1 (ppm)
1.00
0.98
0.97
1.00
1.01
1.04
4.18
1.99
1.06
3.00
3.06
6.00
0.96
6.94
1.27
1.05
2.90
5.71
2.82
9.12
1.00
f1 (ppm)

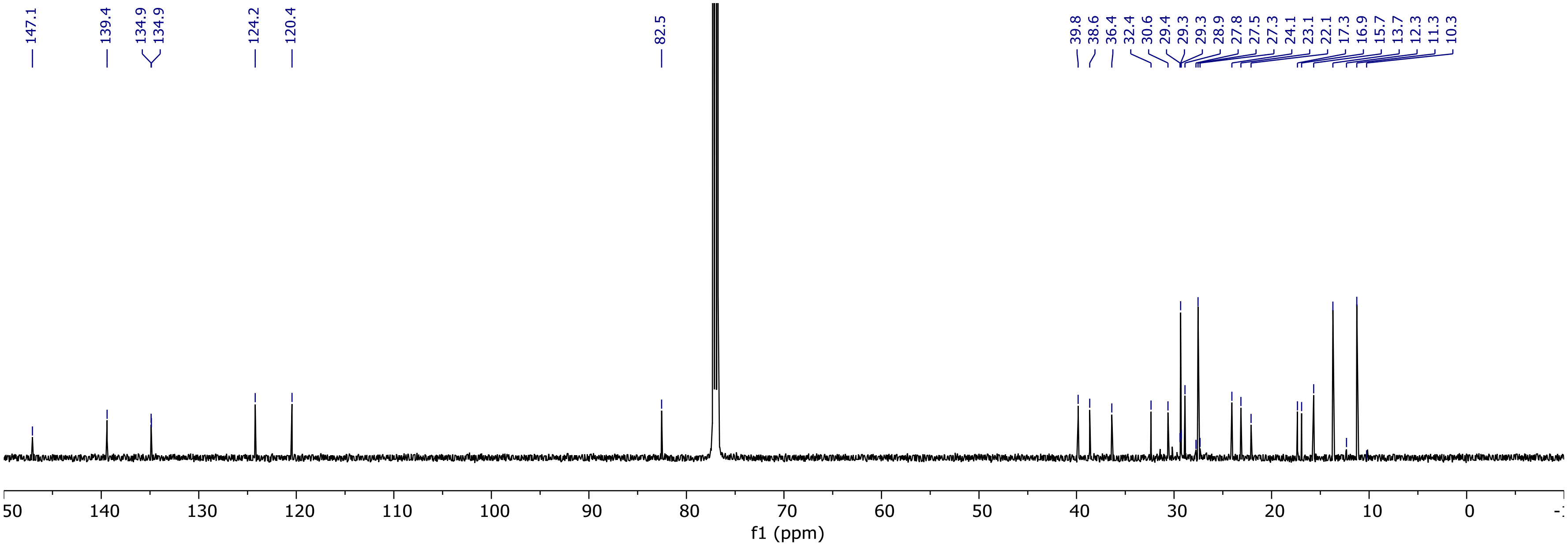
147.1
139.4
134.9
134.9
124.2
120.4
82.5
39.8
38.6
36.4
32.4
30.6
29.4
29.3
29.3
28.9
27.8
27.5
27.3
24.1
23.1
22.1
17.3
16.9
15.7
13.7
12.3
11.3
10.3
f1 (ppm)

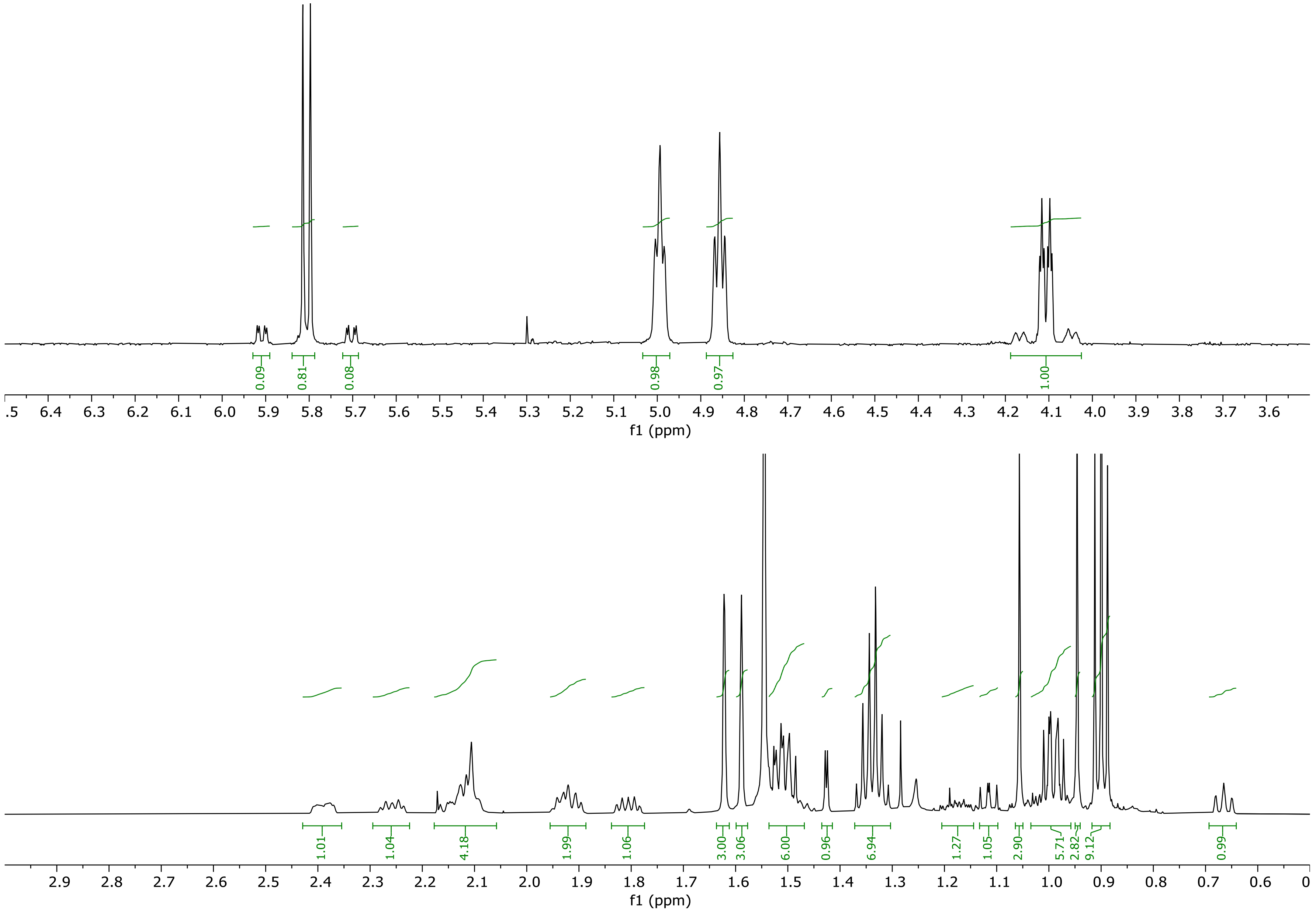
0.09
0.81
0.08
0.98
0.97
1.00
f1 (ppm)
1.01
1.04
4.18
1.99
1.06
3.00
3.06
6.00
0.96
6.94
1.27
1.05
2.90
5.71
2.82
9.12
0.99
f1 (ppm)

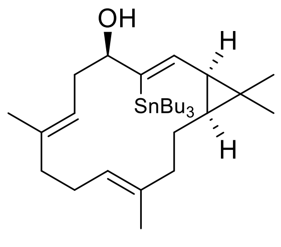
OH
H
SnBu3
H

S2

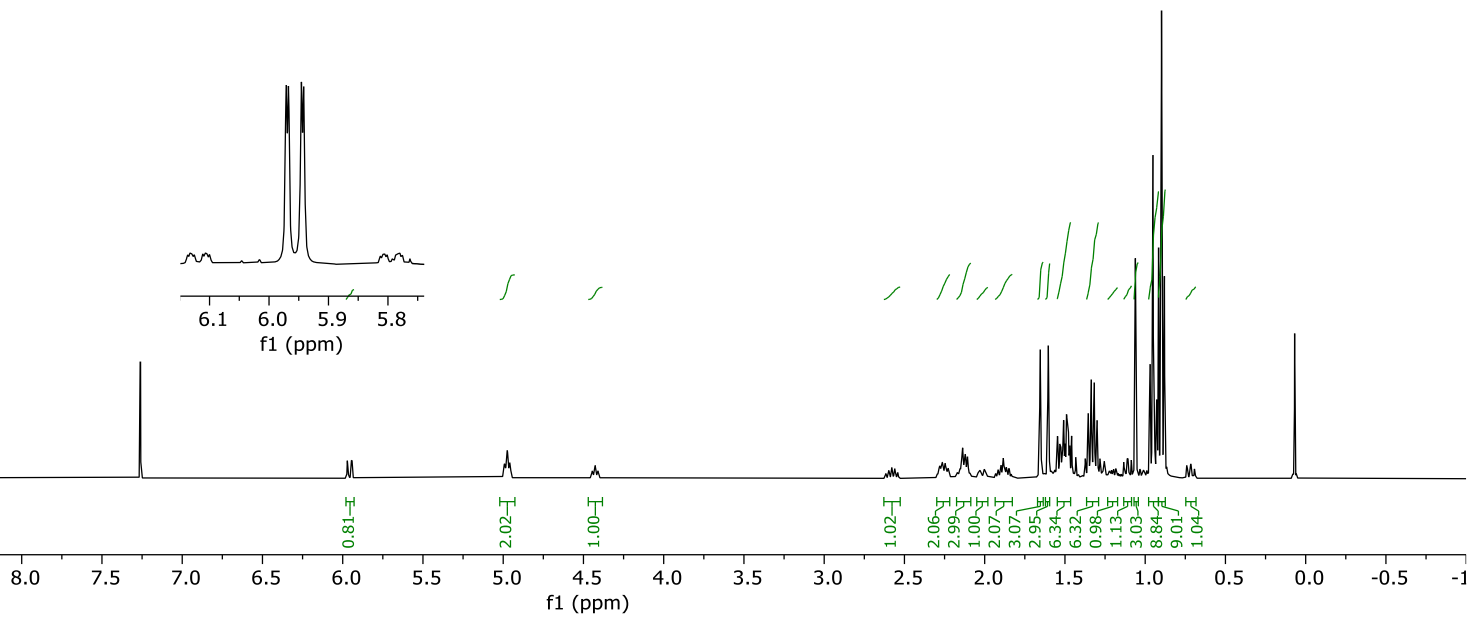
6.1 6.0 5.9 5.8
f1 (ppm)
0.81
2.02
1.00
1.02
2.06
2.99
1.00
2.07
3.07
2.95
6.34
6.32
0.98
1.13
3.03
8.84
9.01
1.04
0.0 9.5 9.0 8.5 8.0 7.5 7.0 6.5 6.0 5.5 5.0 4.5 4.0 3.5 3.0 2.5 2.0 1.5 1.0 0.5 0.0 -0.5 -1
f1 (ppm)

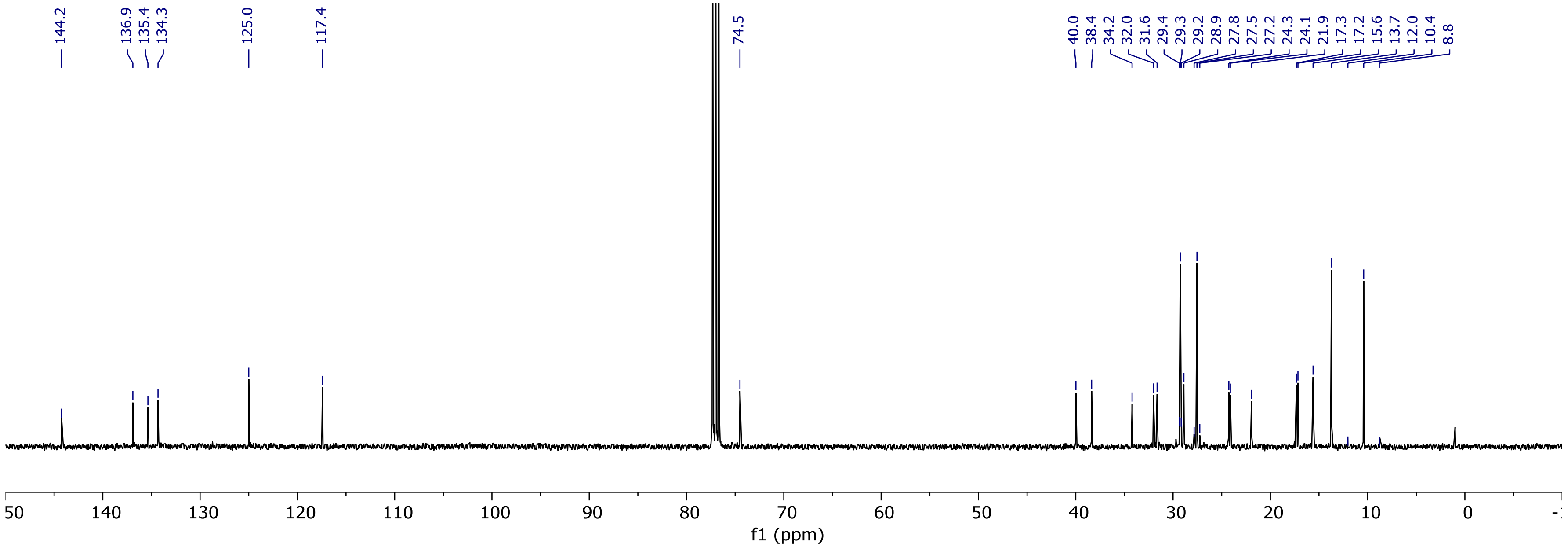
144.2
136.9
135.4
134.3
125.0
117.4
74.5
40.0
38.4
34.2
32.0
31.6
29.4
29.3
29.2
28.9
27.8
27.5
27.2
24.3
24.1
21.9
17.3
17.2
15.6
13.7
12.0
10.4
8.8
50 140 130 120 110 100 90 80 70 60 50 40 30 20 10 0
f1 (ppm)

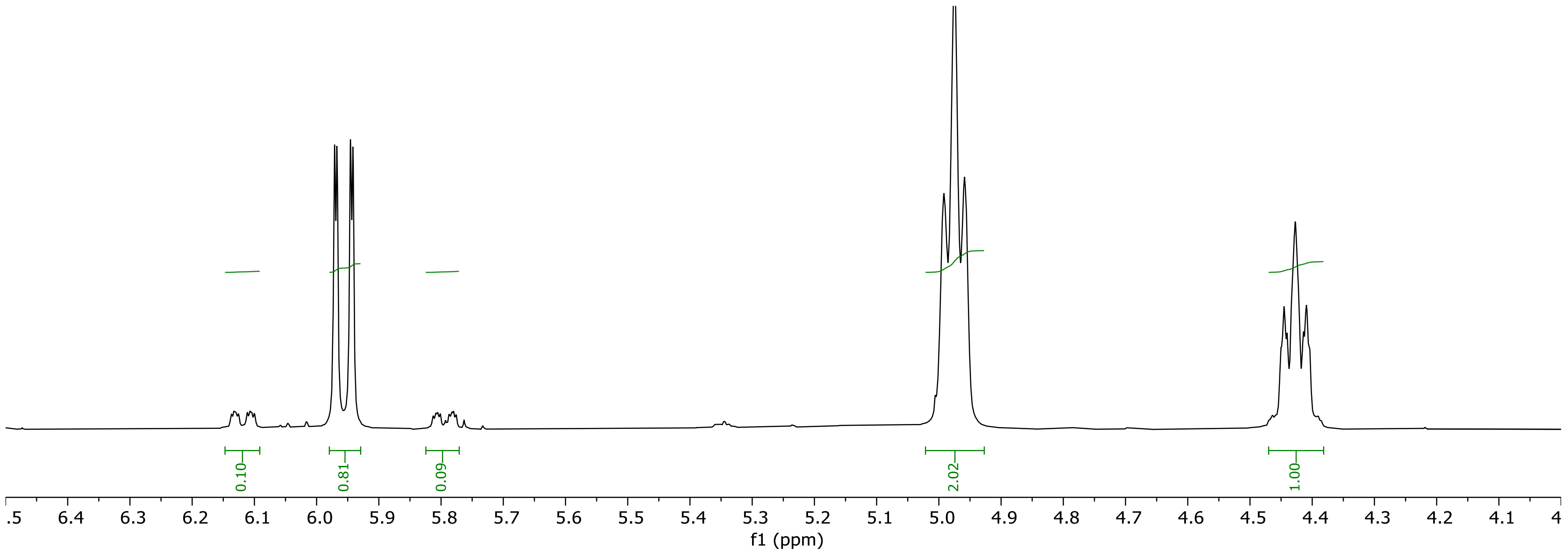

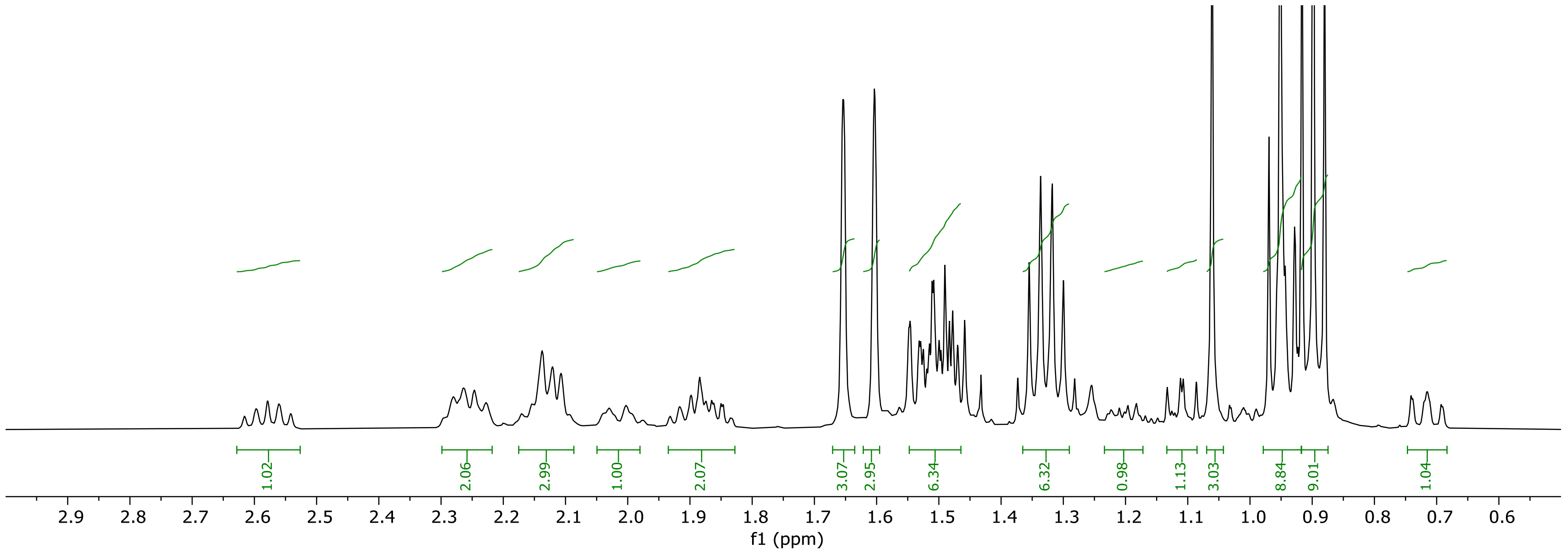

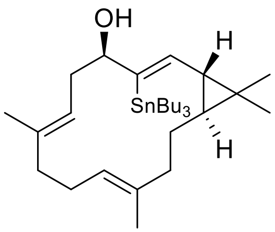
OH
H
SnBu3
H

42

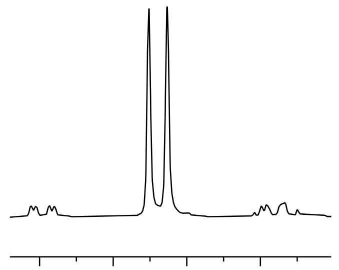

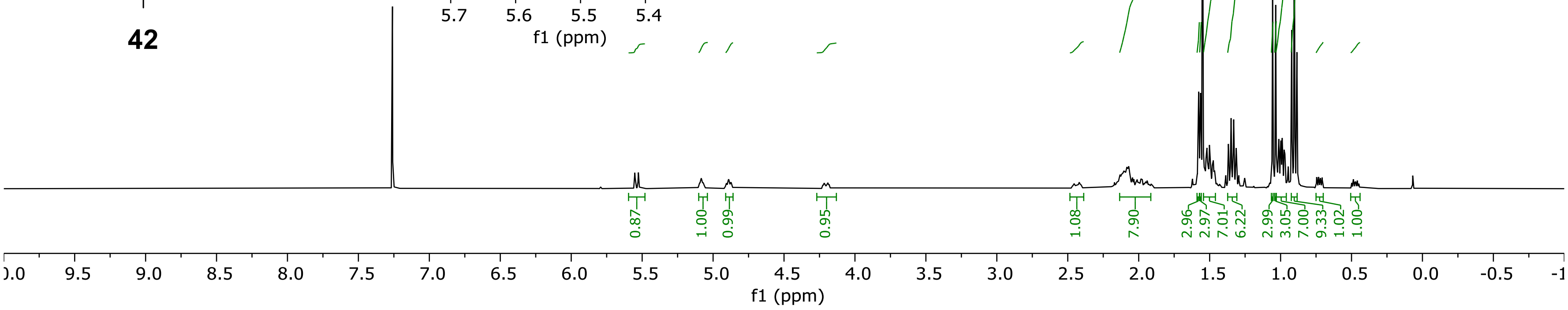
5.7
5.6
5.5
5.4
f1 (ppm)
0.87
1.00
0.99
0.95
1.08
7.90
2.96
2.97
7.01
6.22
2.99
3.05
7.00
9.33
1.02
1.00
9.5
9.0
8.5
8.0
7.5
7.0
6.5
6.0
5.5
5.0
4.5
4.0
3.5
3.0
2.5
2.0
1.5
1.0
0.5
0.0
-0.5
f1 (ppm)

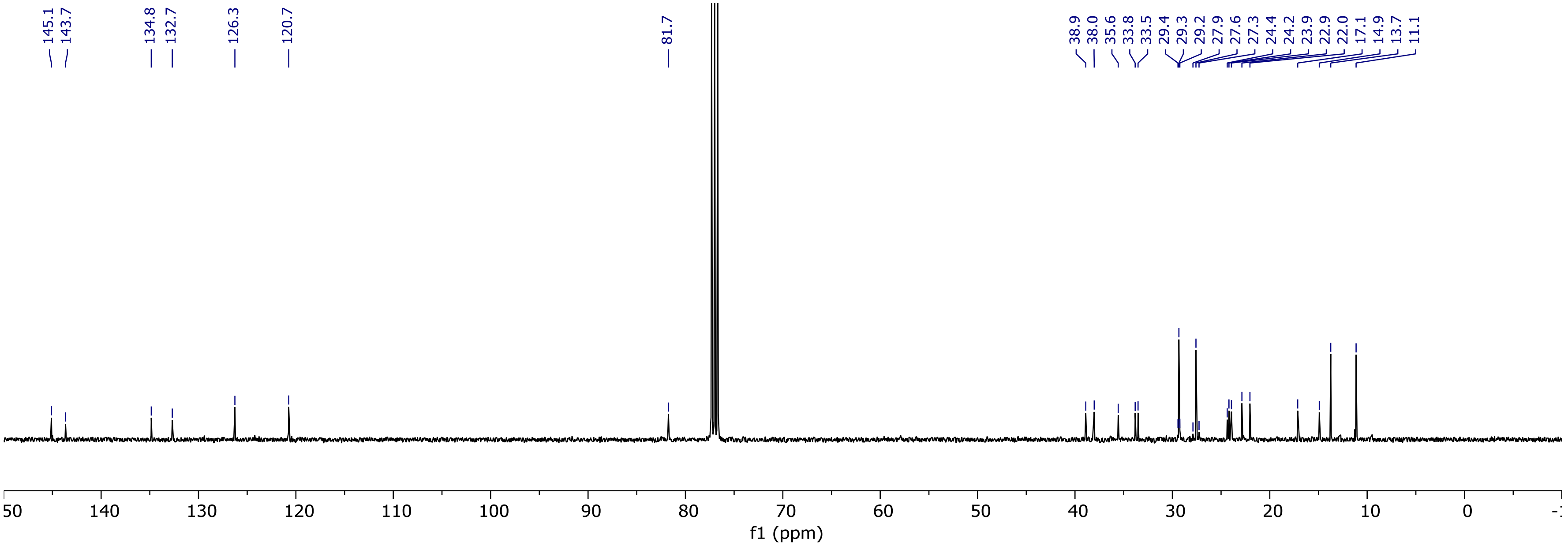
145.1
143.7
134.8
132.7
126.3
120.7
81.7
38.9
38.0
35.6
33.8
33.5
29.4
29.3
29.2
27.9
27.6
27.3
24.4
24.2
23.9
22.9
22.0
17.1
14.9
13.7
11.1
140
130
120
110
100
90
80
70
60
50
40
30
20
10
0
f1 (ppm)

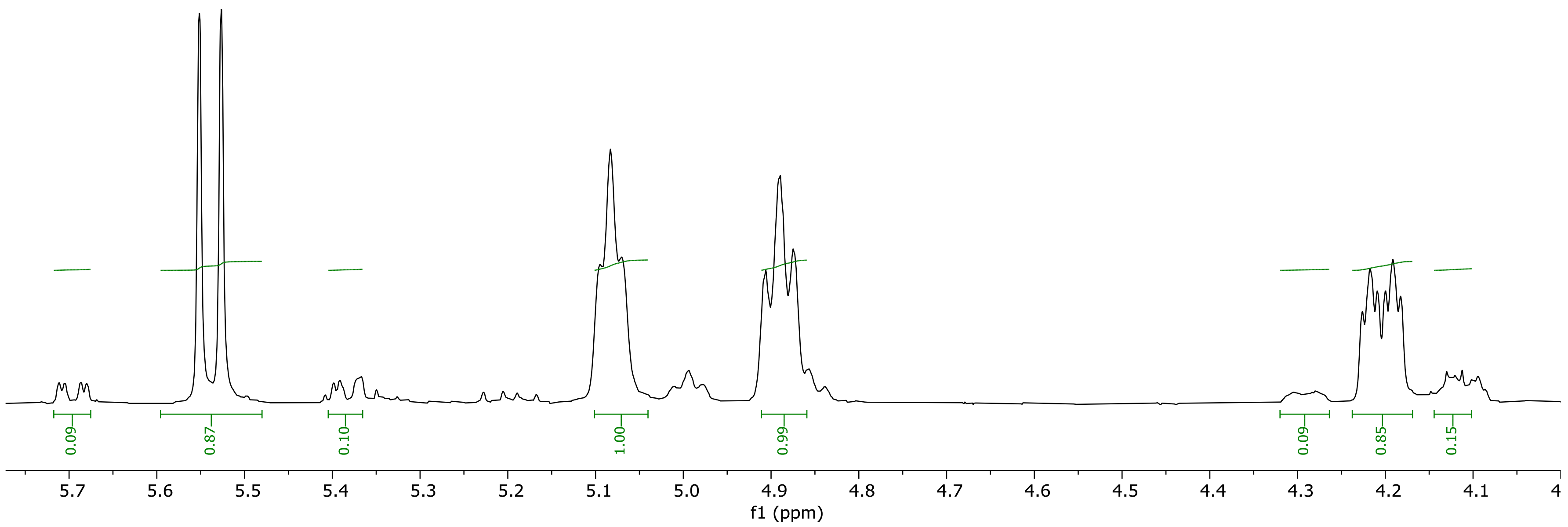
0.09
0.87
0.10
1.00
0.99
0.09
0.85
0.15
5.7
5.6
5.5
5.4
5.3
5.2
5.1
5.0
4.9
4.8
4.7
4.6
4.5
4.4
4.3
4.2
4.1
4
f1 (ppm)

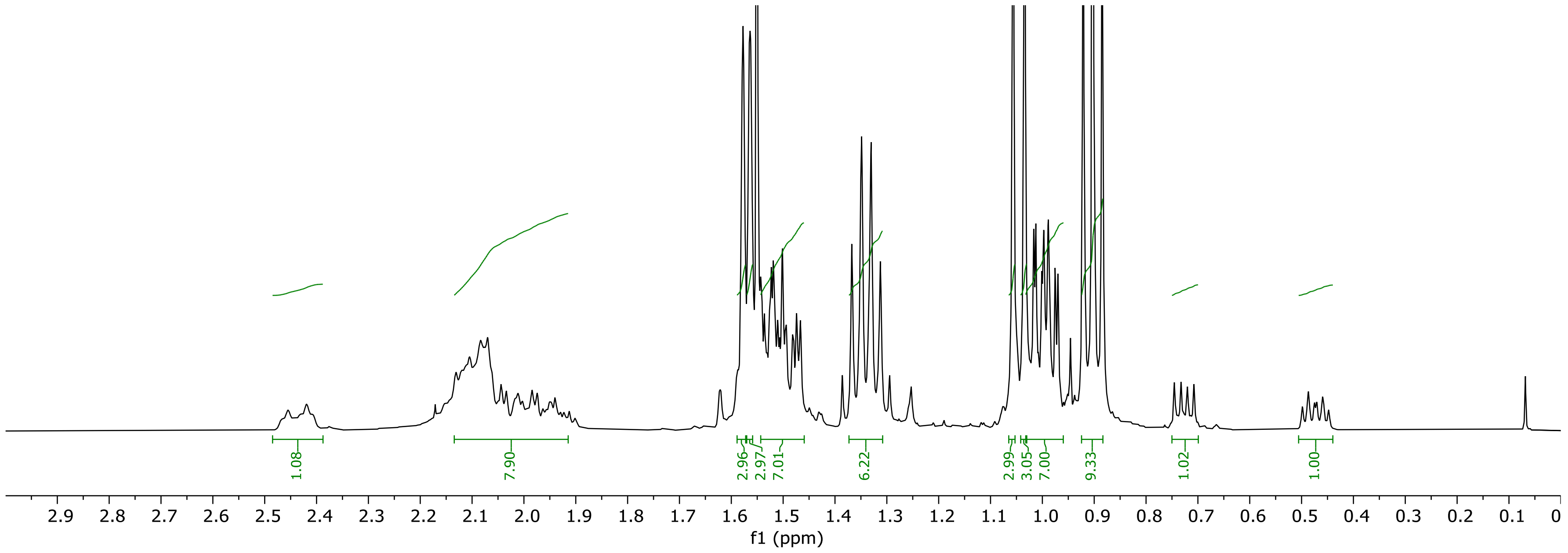
1.08
7.90
2.96
2.97
7.01
6.22
2.99
3.05
7.00
9.33
1.02
1.00
2.9
2.8
2.7
2.6
2.5
2.4
2.3
2.2
2.1
2.0
1.9
1.8
1.7
1.6
1.5
1.4
1.3
1.2
1.1
1.0
0.9
0.8
0.7
0.6
0.5
0.4
0.3
0.2
0.1
0
f1 (ppm)

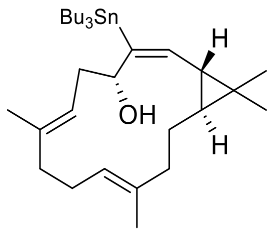
Bu3Sn
H
OH
H

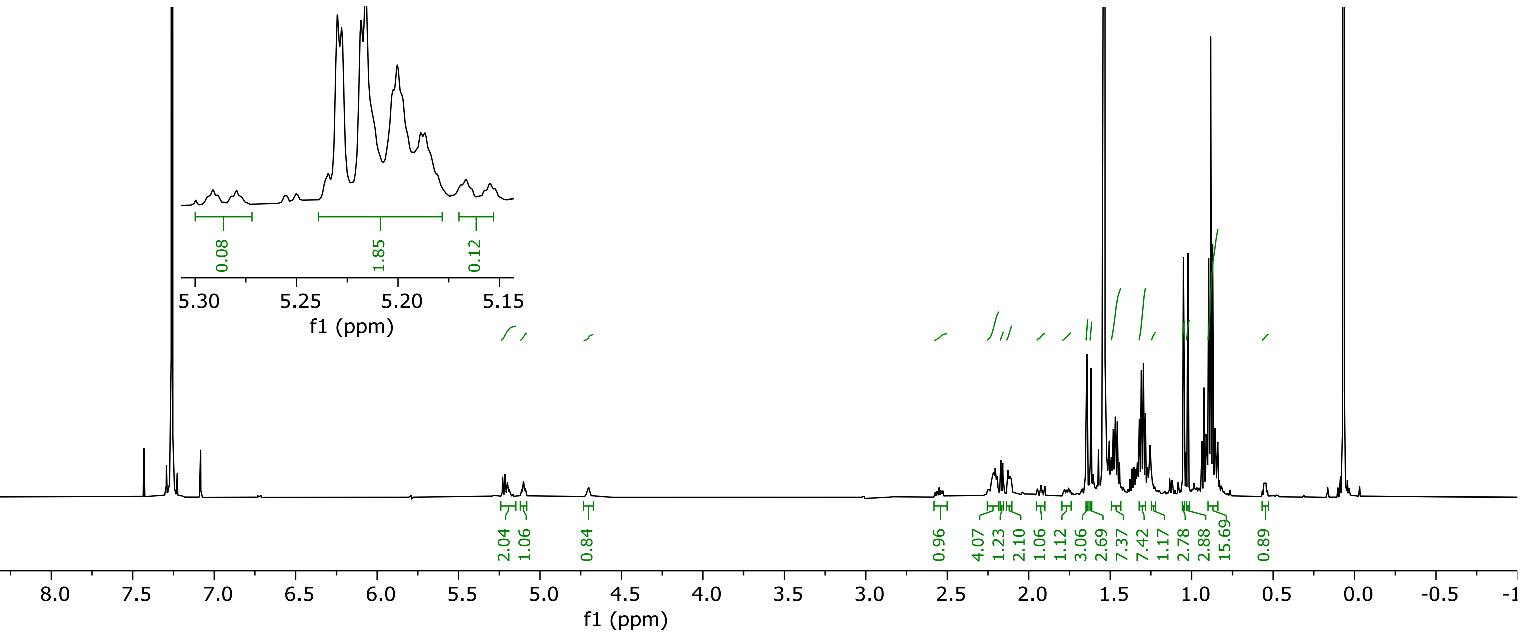
0.08
1.85
0.12
5.30 5.25 5.20 5.15
f1 (ppm)
2.04 1.06 0.84 0.96 4.07 1.23 2.10 1.06 1.12 3.06 2.69 7.37 7.42 1.17 2.78 2.88 15.69 0.89
f1 (ppm)

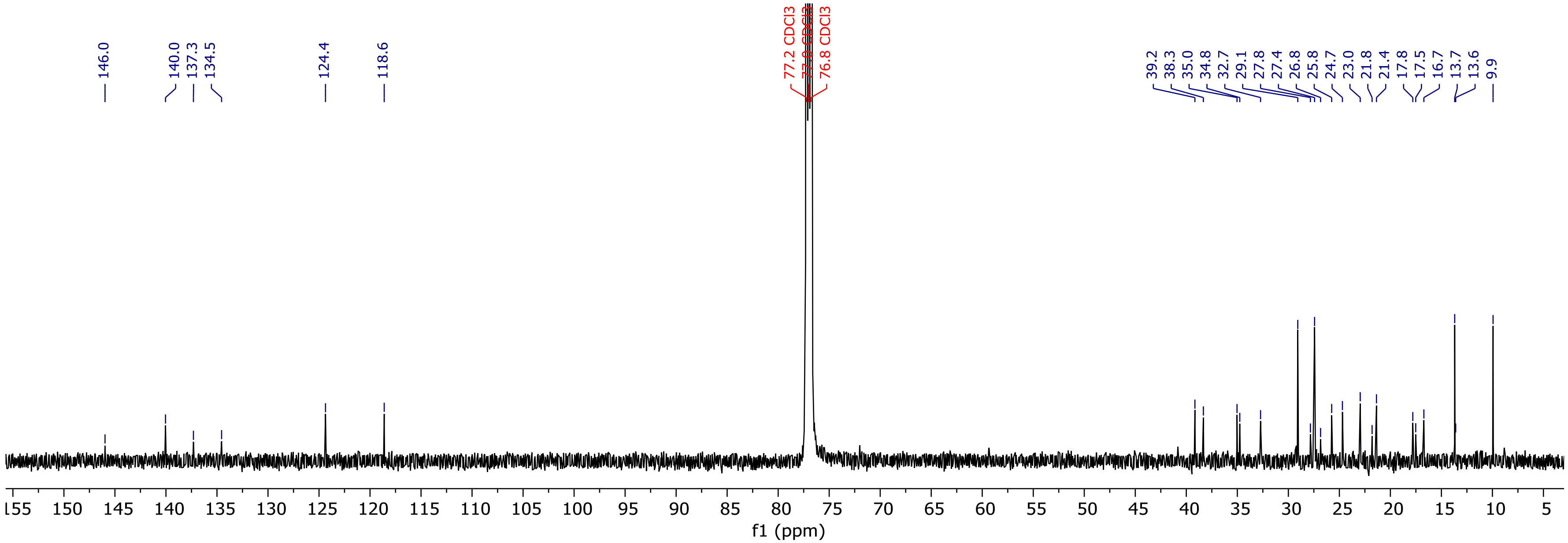
146.0
140.0
137.3
134.5
124.4
118.6
77.2 CDCl3
77.0 CDCl3
76.8 CDCl3
39.2
38.3
35.0
34.8
32.7
29.1
27.8
27.4
26.8
25.8
24.7
23.0
21.8
21.4
17.8
17.5
16.7
13.7
13.6
9.9
f1 (ppm)

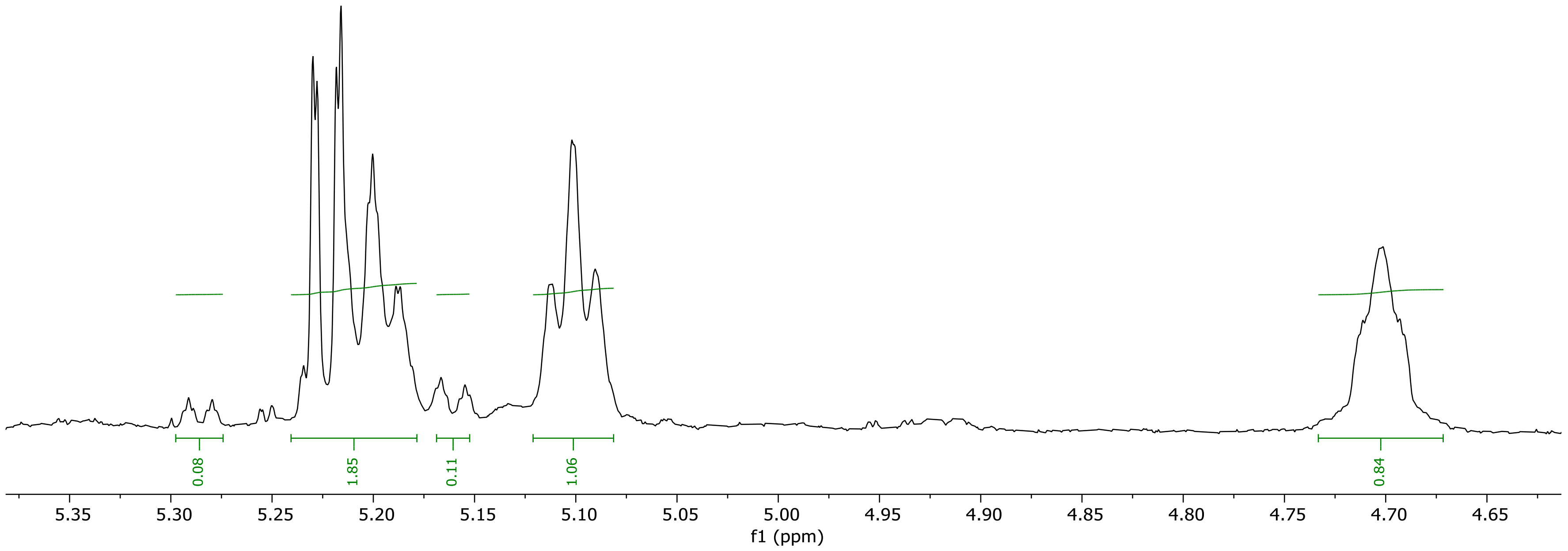
0.08
1.85
0.11
1.06
0.84
5.35
5.30
5.25
5.20
5.15
5.10
5.05
5.00
4.95
4.90
4.85
4.80
4.75
4.70
4.65
f1 (ppm)

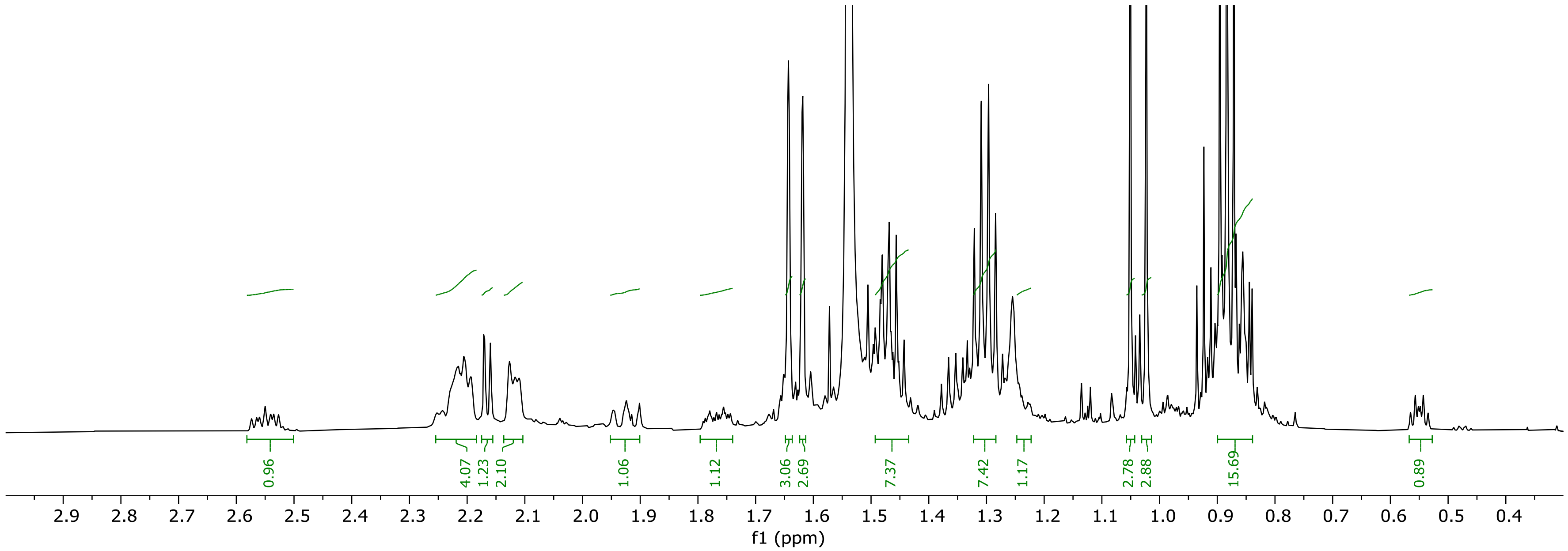
0.96
4.07
1.23
2.10
1.06
1.12
3.06
2.69
7.37
7.42
1.17
2.78
2.88
15.69
0.89
2.9
2.8
2.7
2.6
2.5
2.4
2.3
2.2
2.1
2.0
1.9
1.8
1.7
1.6
1.5
1.4
1.3
1.2
1.1
1.0
0.9
0.8
0.7
0.6
0.5
0.4
f1 (ppm)

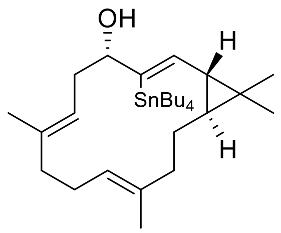
OH
H
SnBu4
H

43

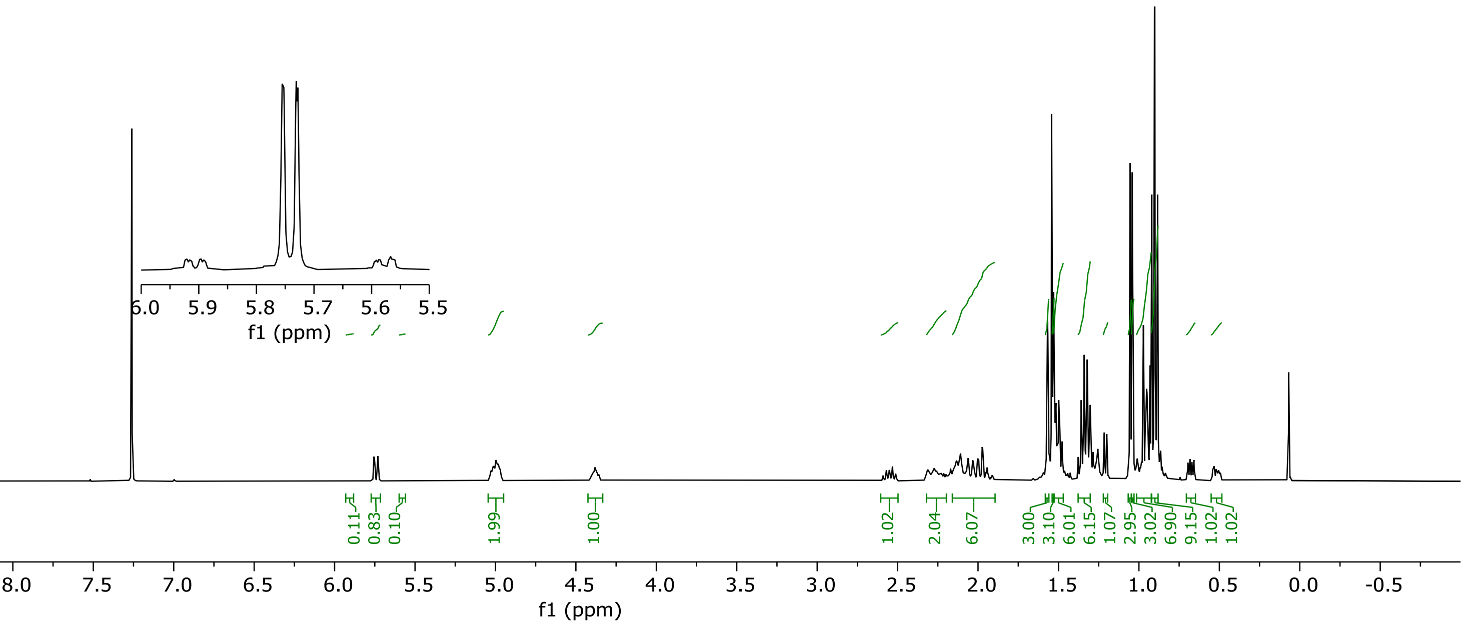
6.0
5.9
5.8
5.7
5.6
5.5
f1 (ppm)
0.11
0.83
0.10
1.99
1.00
1.02
2.04
6.07
3.00
3.10
6.01
6.15
1.07
2.95
3.02
6.90
9.15
1.02
1.02
9.5
9.0
8.5
8.0
7.5
7.0
6.5
6.0
5.5
5.0
4.5
4.0
3.5
3.0
2.5
2.0
1.5
1.0
0.5
0.0
-0.5
f1 (ppm)

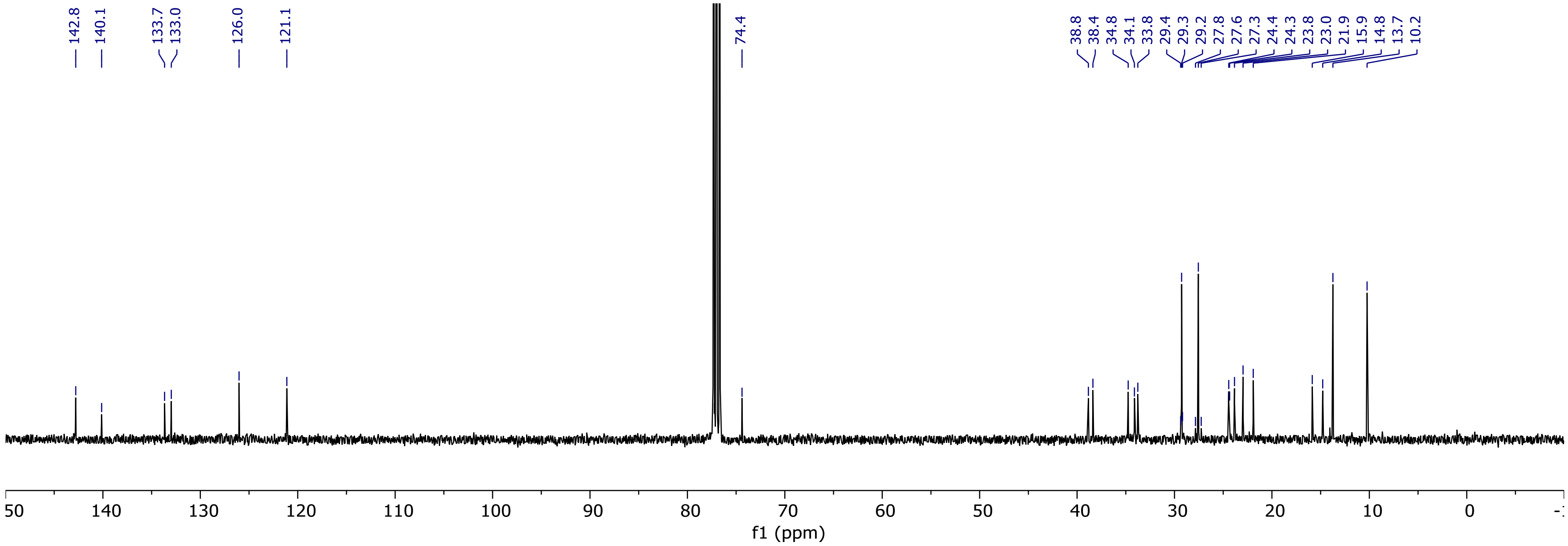
142.8
140.1
133.7
133.0
126.0
121.1
74.4
38.8
38.4
34.8
34.1
33.8
29.4
29.3
29.2
27.8
27.6
27.3
24.4
24.3
23.8
23.0
21.9
15.9
14.8
13.7
10.2
140
130
120
110
100
90
80
70
60
50
40
30
20
10
0
f1 (ppm)

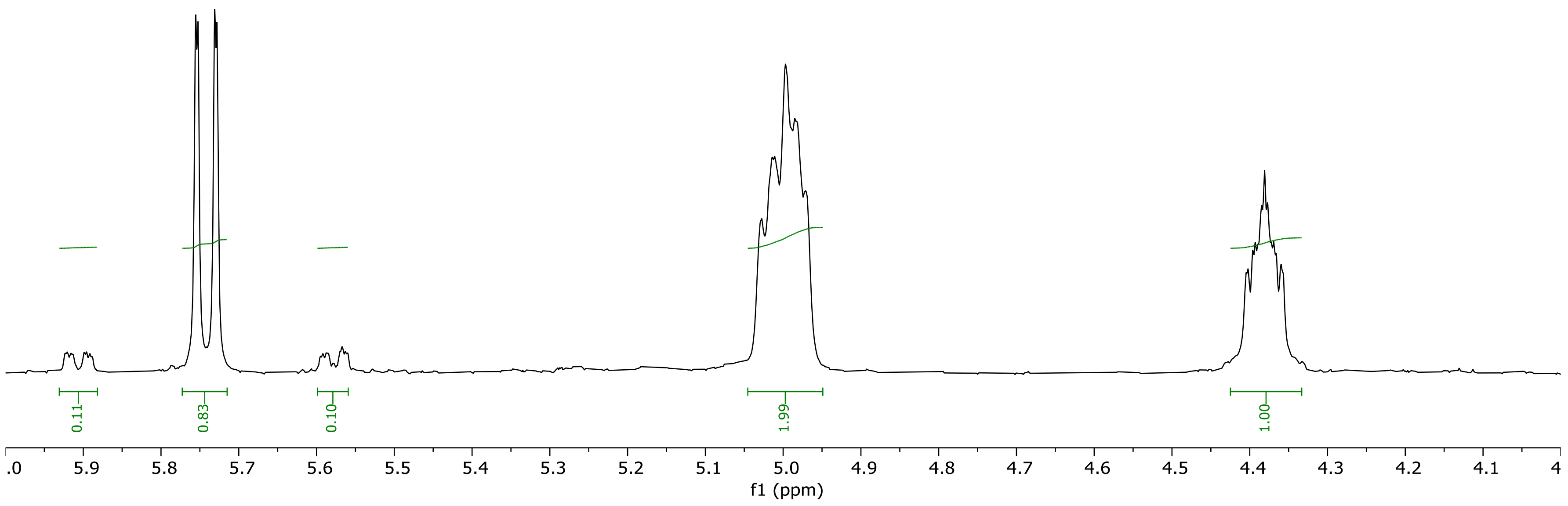

0.11
0.83
0.10
1.99
1.00
.0
5.9
5.8
5.7
5.6
5.5
5.4
5.3
5.2
5.1
5.0
4.9
4.8
4.7
4.6
4.5
4.4
4.3
4.2
4.1
4
f1 (ppm)

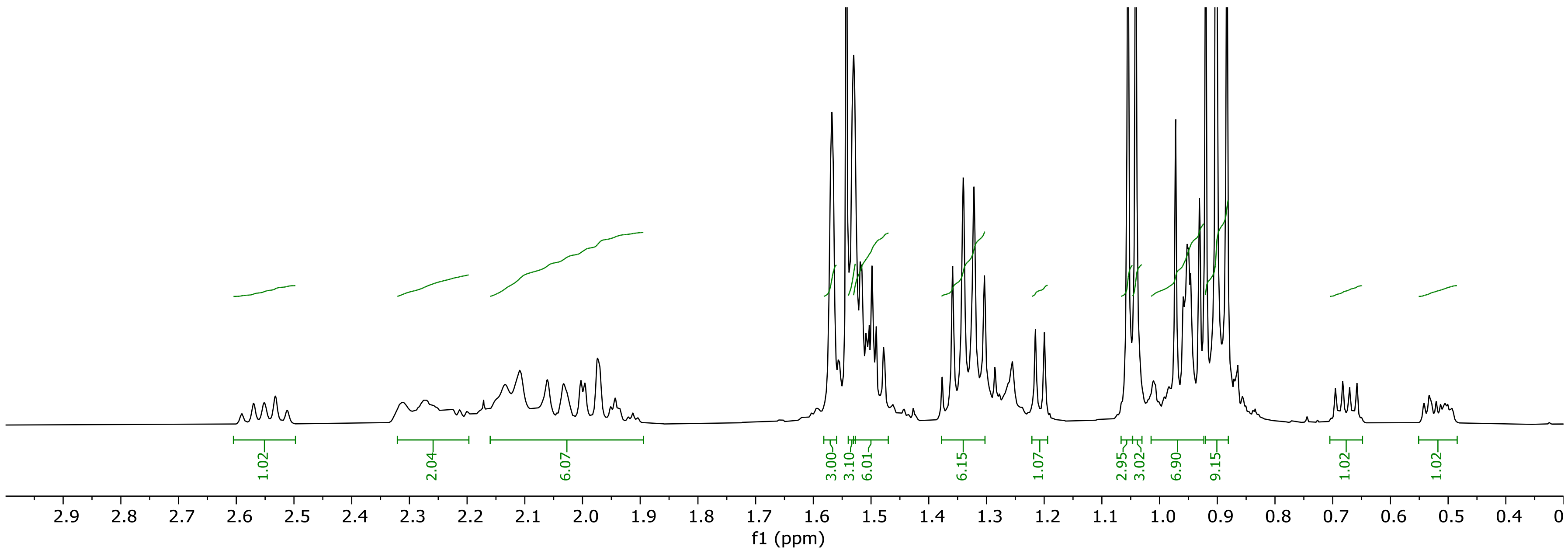

1.02
2.04
6.07
3.00
3.10
6.01
6.15
1.07
2.95
3.02
6.90
9.15
1.02
1.02
2.9
2.8
2.7
2.6
2.5
2.4
2.3
2.2
2.1
2.0
1.9
1.8
1.7
1.6
1.5
1.4
1.3
1.2
1.1
1.0
0.9
0.8
0.7
0.6
0.5
0.4
0
f1 (ppm)

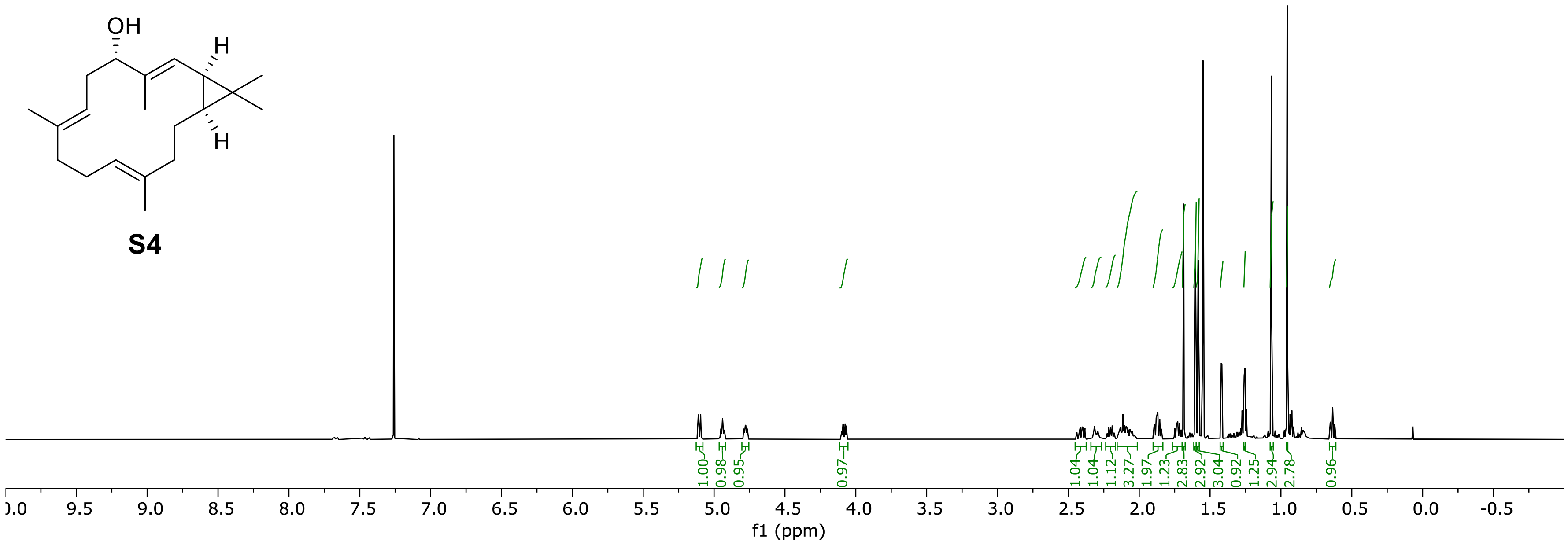
OH
H
H
S4
1.00
0.98
0.95
0.97
1.04
1.04
1.12
3.27
1.97
1.23
2.83
2.92
3.04
0.92
1.25
2.94
2.78
0.96
f1 (ppm)

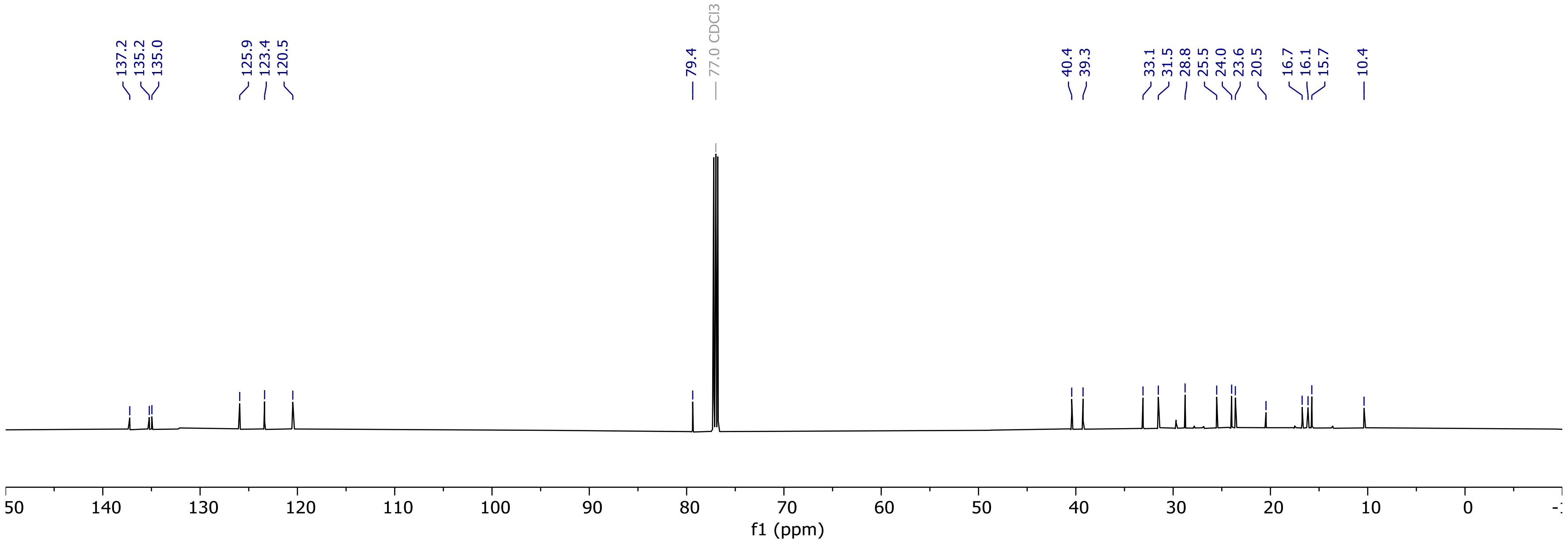
137.2
135.2
135.0
125.9
123.4
120.5
79.4
77.0 CDCl3
40.4
39.3
33.1
31.5
28.8
25.5
24.0
23.6
20.5
16.7
16.1
15.7
10.4
f1 (ppm)

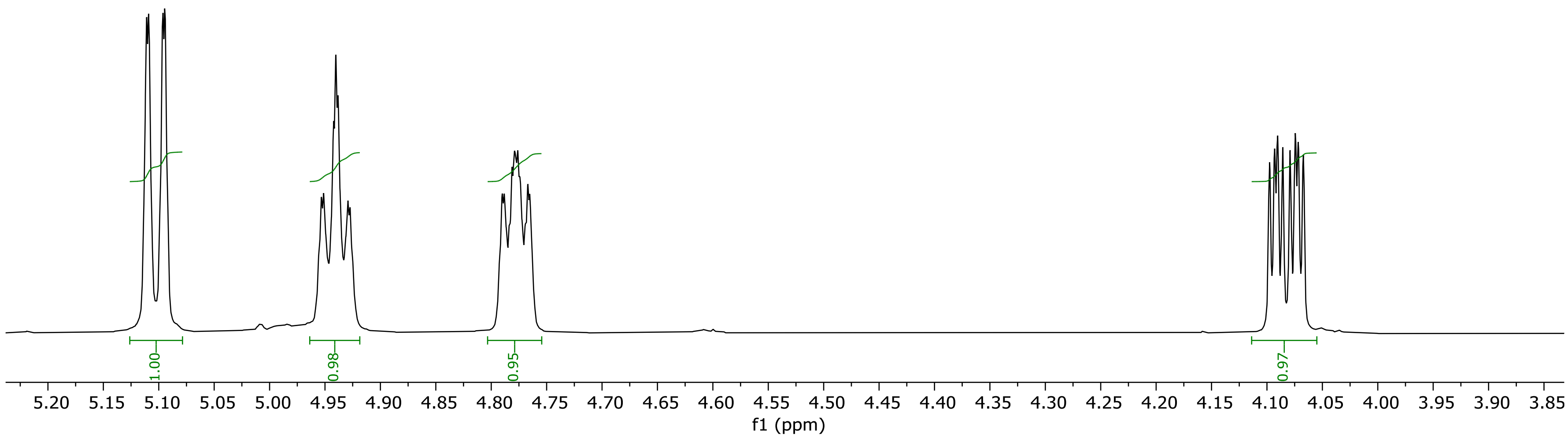
1.00
0.98
0.95
0.97
5.20
5.15
5.10
5.05
5.00
4.95
4.90
4.85
4.80
4.75
4.70
4.65
4.60
4.55
4.50
4.45
4.40
4.35
4.30
4.25
4.20
4.15
4.10
4.05
4.00
3.95
3.90
3.85
f1 (ppm)

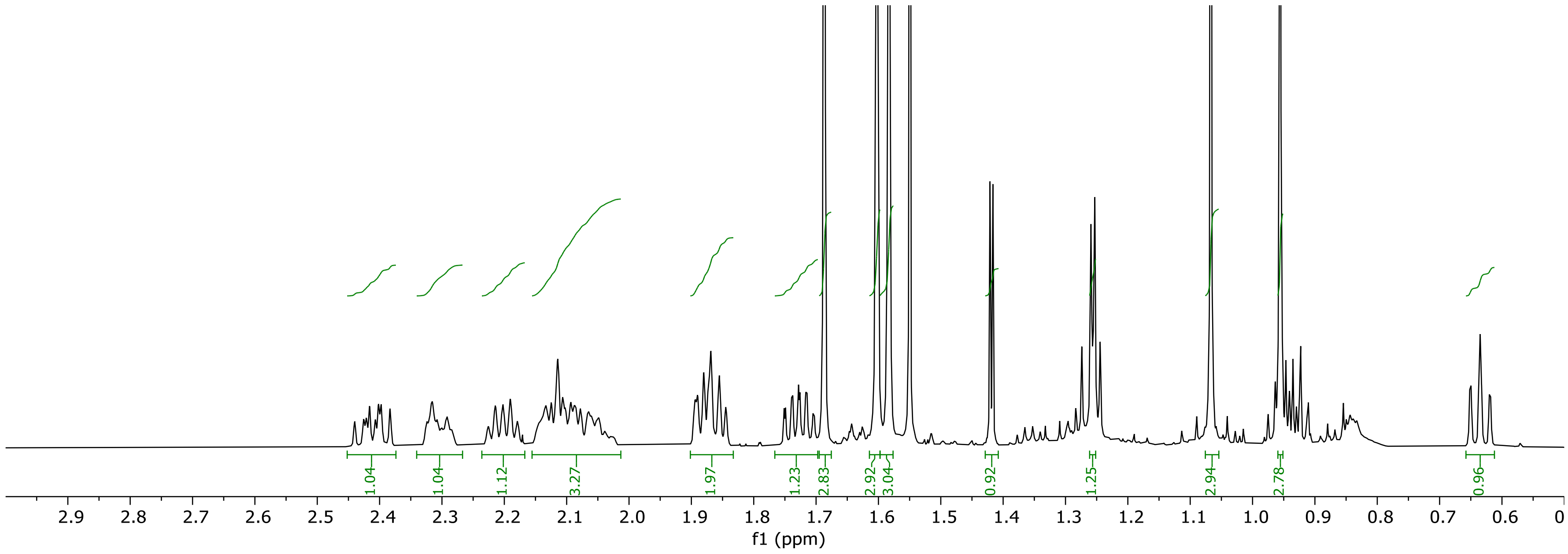
1.04
1.04
1.12
3.27
1.97
1.23
2.83
2.92
3.04
0.92
1.25
2.94
2.78
0.96
2.9
2.8
2.7
2.6
2.5
2.4
2.3
2.2
2.1
2.0
1.9
1.8
1.7
1.6
1.5
1.4
1.3
1.2
1.1
1.0
0.9
0.8
0.7
0.6
0
f1 (ppm)

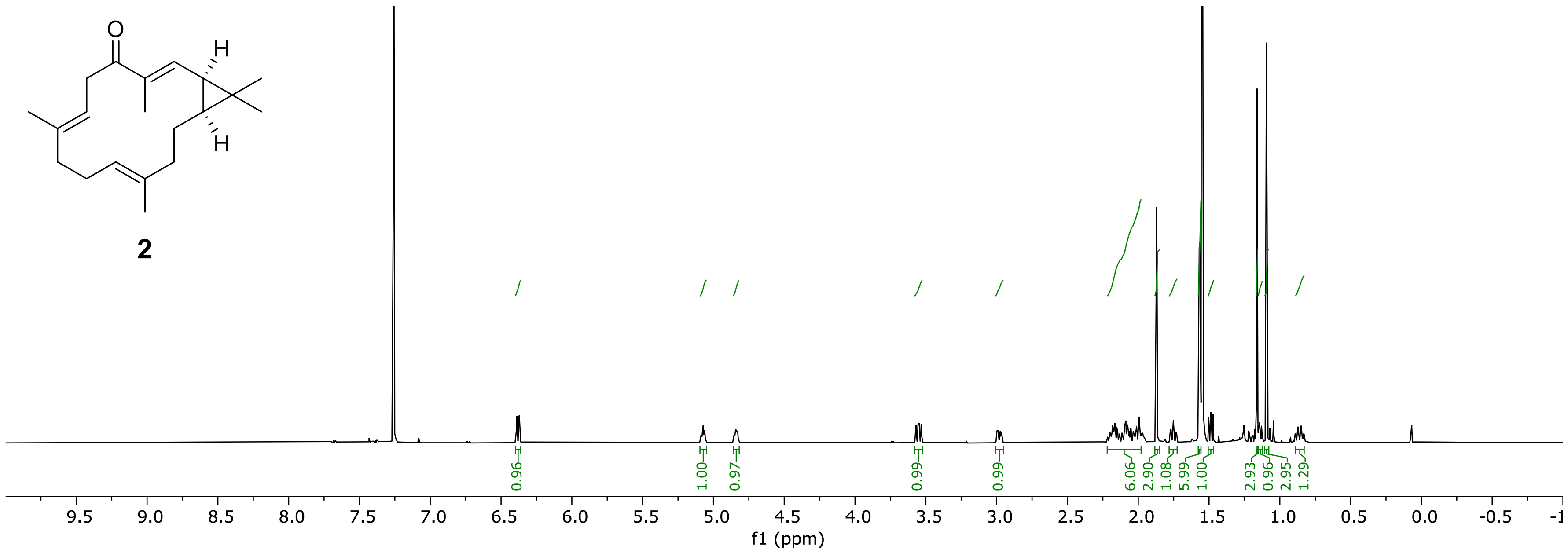
O
H
H
2
0.96
1.00
0.97
0.99
0.99
6.06
2.90
1.08
5.99
1.00
2.93
0.96
2.95
1.29
9.5
9.0
8.5
8.0
7.5
7.0
6.5
6.0
5.5
5.0
4.5
4.0
3.5
3.0
2.5
2.0
1.5
1.0
0.5
0.0
-0.5
f1 (ppm)

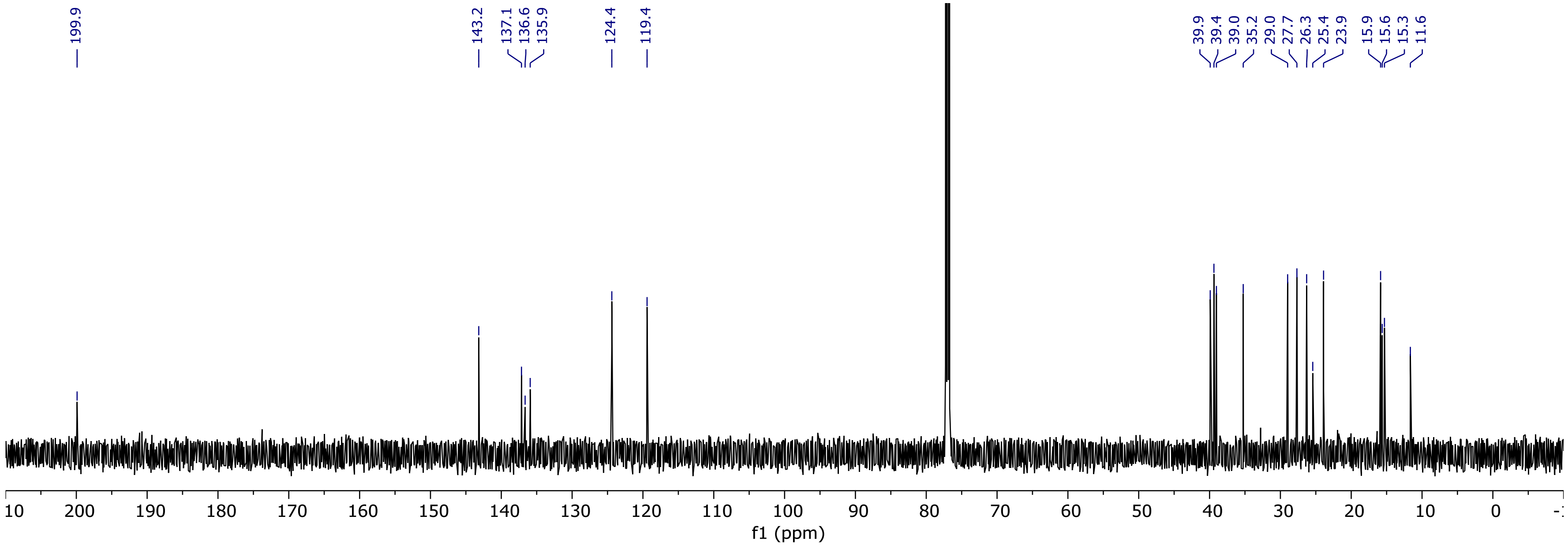
199.9
143.2
137.1
136.6
135.9
124.4
119.4
39.9
39.4
39.0
35.2
29.0
27.7
26.3
25.4
23.9
15.9
15.6
15.3
11.6
200
190
180
170
160
150
140
130
120
110
100
90
80
70
60
50
40
30
20
10
0
f1 (ppm)

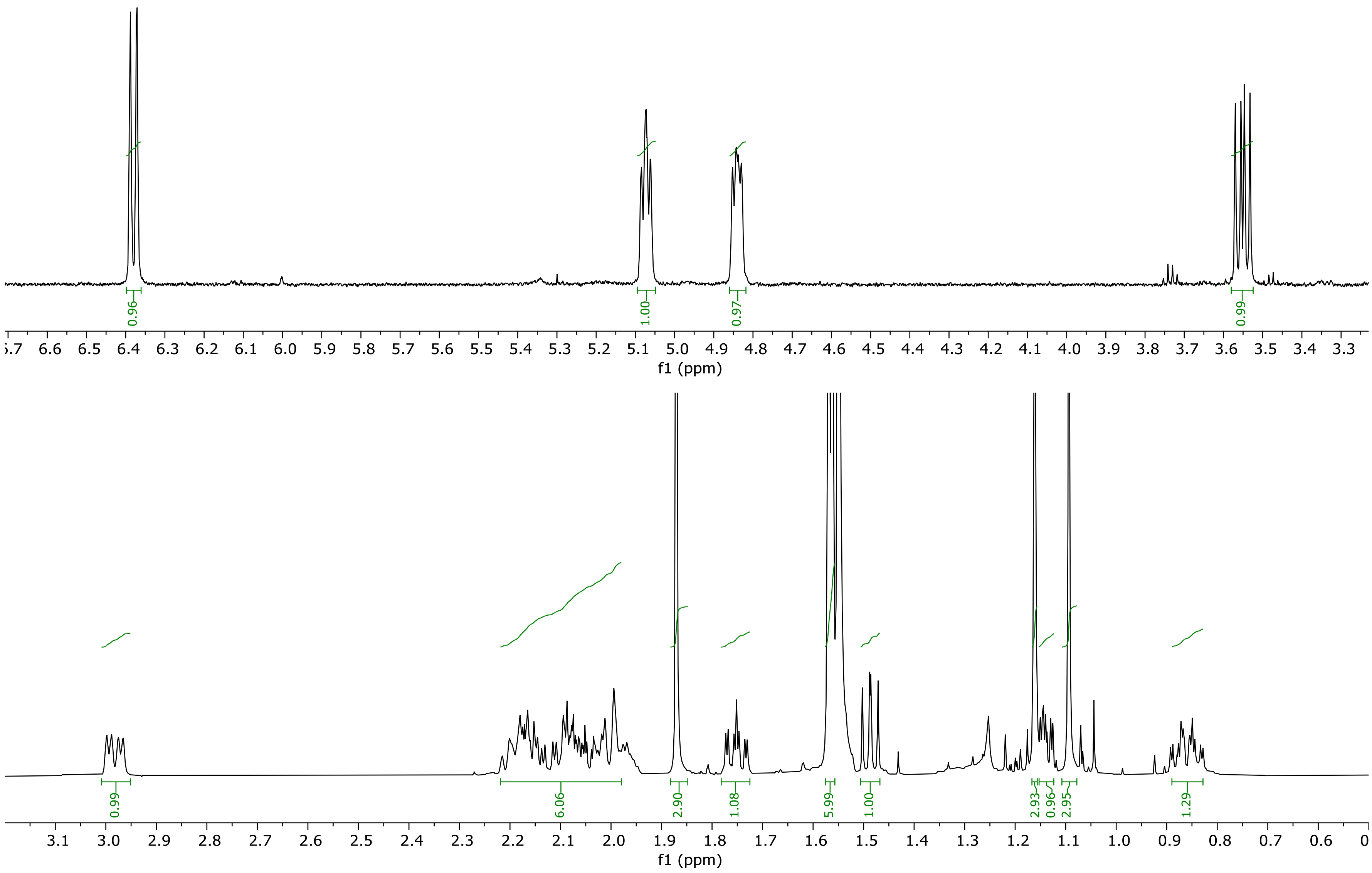

0.96
1.00
0.97
0.99
6.7 6.6 6.5 6.4 6.3 6.2 6.1 6.0 5.9 5.8 5.7 5.6 5.5 5.4 5.3 5.2 5.1 5.0 4.9 4.8 4.7 4.6 4.5 4.4 4.3 4.2 4.1 4.0 3.9 3.8 3.7 3.6 3.5 3.4 3.3
f1 (ppm)
0.99
6.06
2.90
1.08
5.99
1.00
2.93
0.96
2.95
1.29
3.1 3.0 2.9 2.8 2.7 2.6 2.5 2.4 2.3 2.2 2.1 2.0 1.9 1.8 1.7 1.6 1.5 1.4 1.3 1.2 1.1 1.0 0.9 0.8 0.7 0.6 0
f1 (ppm)

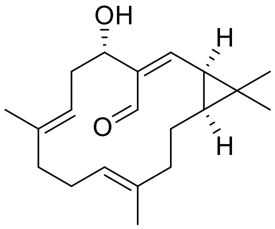

OH
H
O
H

4

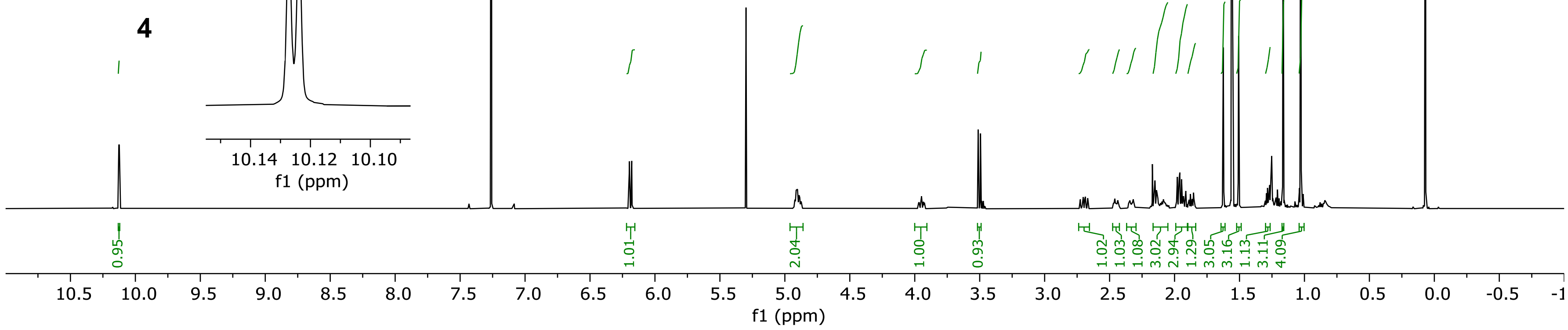

10.14 10.12 10.10
f1 (ppm)
0.95
1.01
2.04
1.00
0.93
1.02
1.03
1.08
3.02
2.94
1.29
3.05
3.16
1.13
3.11
4.09
10.5 10.0 9.5 9.0 8.5 8.0 7.5 7.0 6.5 6.0 5.5 5.0 4.5 4.0 3.5 3.0 2.5 2.0 1.5 1.0 0.5 0.0 -0.5
f1 (ppm)

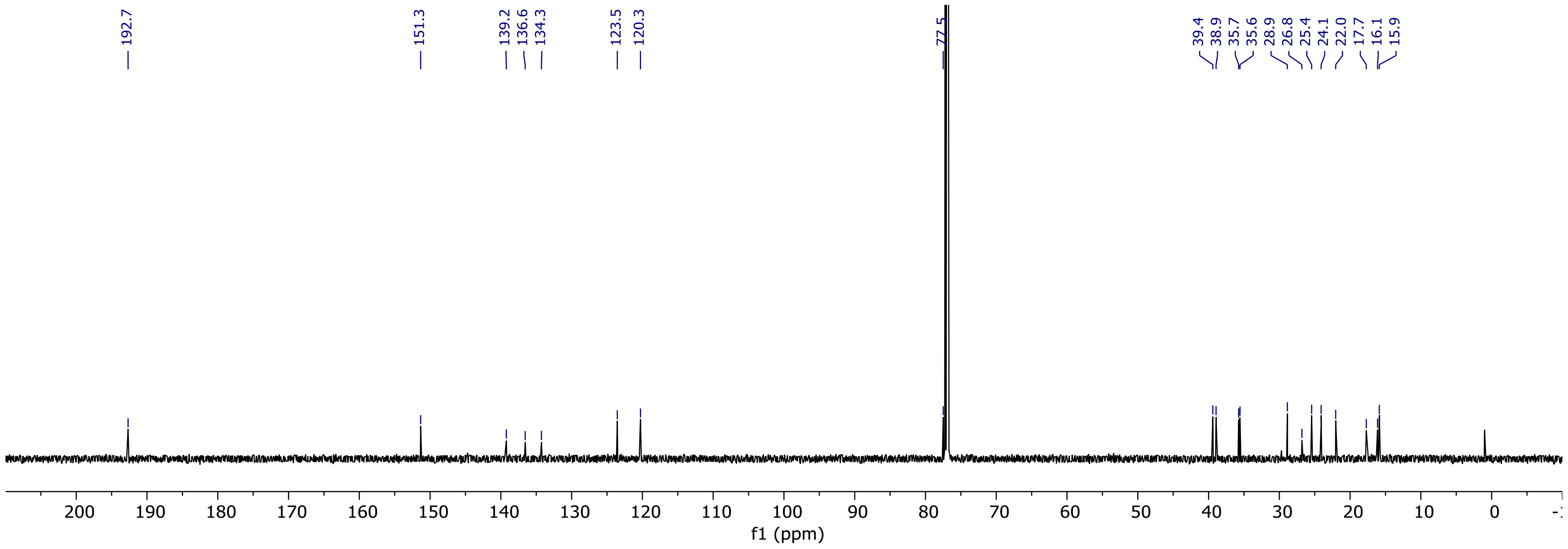

192.7
151.3
139.2
136.6
134.3
123.5
120.3
77.5
39.4
38.9
35.7
35.6
28.9
26.8
25.4
24.1
22.0
17.7
16.1
15.9
200 190 180 170 160 150 140 130 120 110 100 90 80 70 60 50 40 30 20 10 0
f1 (ppm)

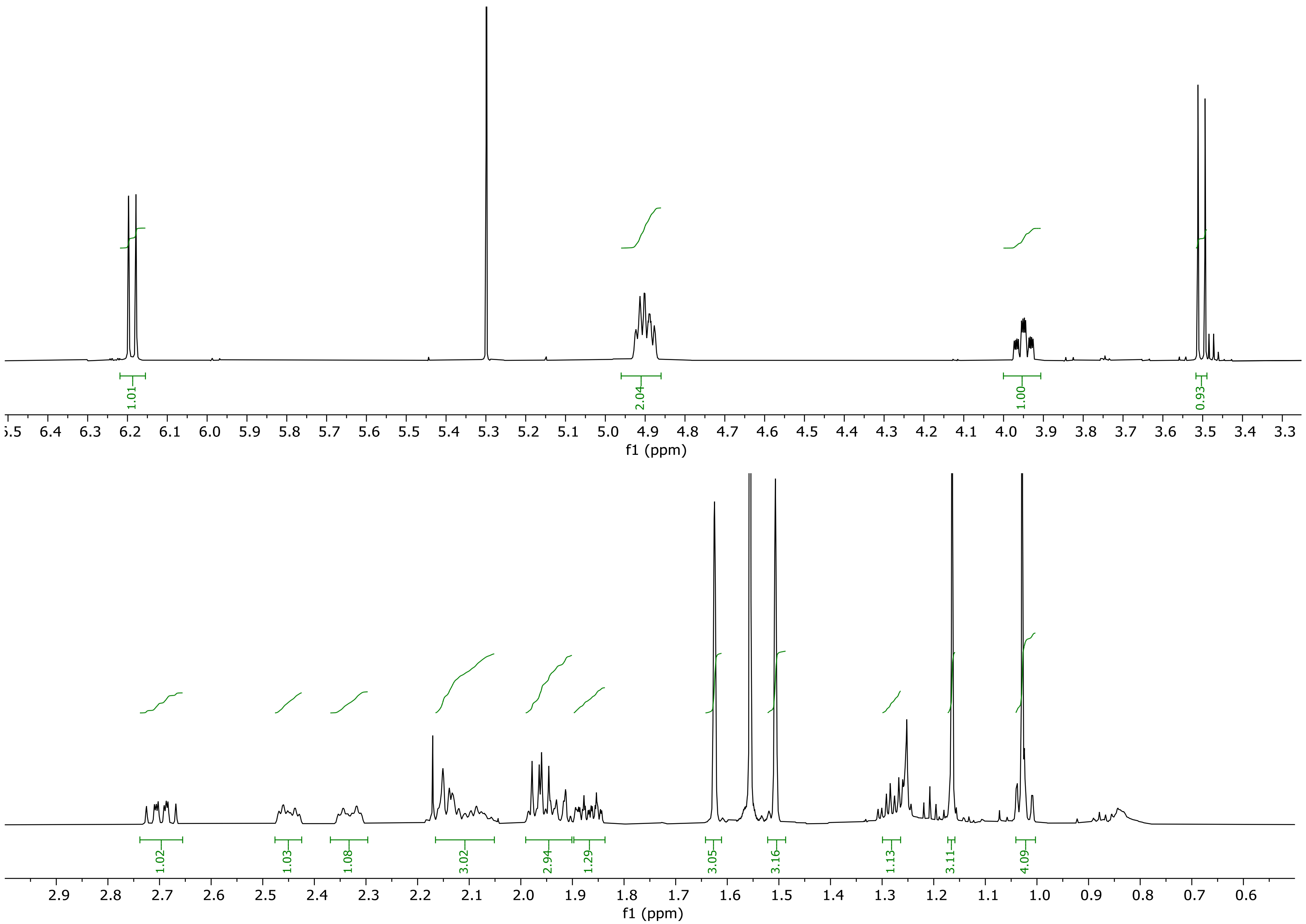
1.01
2.04
1.00
0.93
6.5 6.4 6.3 6.2 6.1 6.0 5.9 5.8 5.7 5.6 5.5 5.4 5.3 5.2 5.1 5.0 4.9 4.8 4.7 4.6 4.5 4.4 4.3 4.2 4.1 4.0 3.9 3.8 3.7 3.6 3.5 3.4 3.3
f1 (ppm)
1.02
1.03
1.08
3.02
2.94
1.29
3.05
3.16
1.13
3.11
4.09
2.9 2.8 2.7 2.6 2.5 2.4 2.3 2.2 2.1 2.0 1.9 1.8 1.7 1.6 1.5 1.4 1.3 1.2 1.1 1.0 0.9 0.8 0.7 0.6
f1 (ppm)

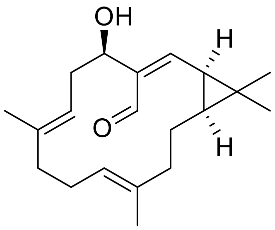

OH
H
O
H

5-*epi*-4

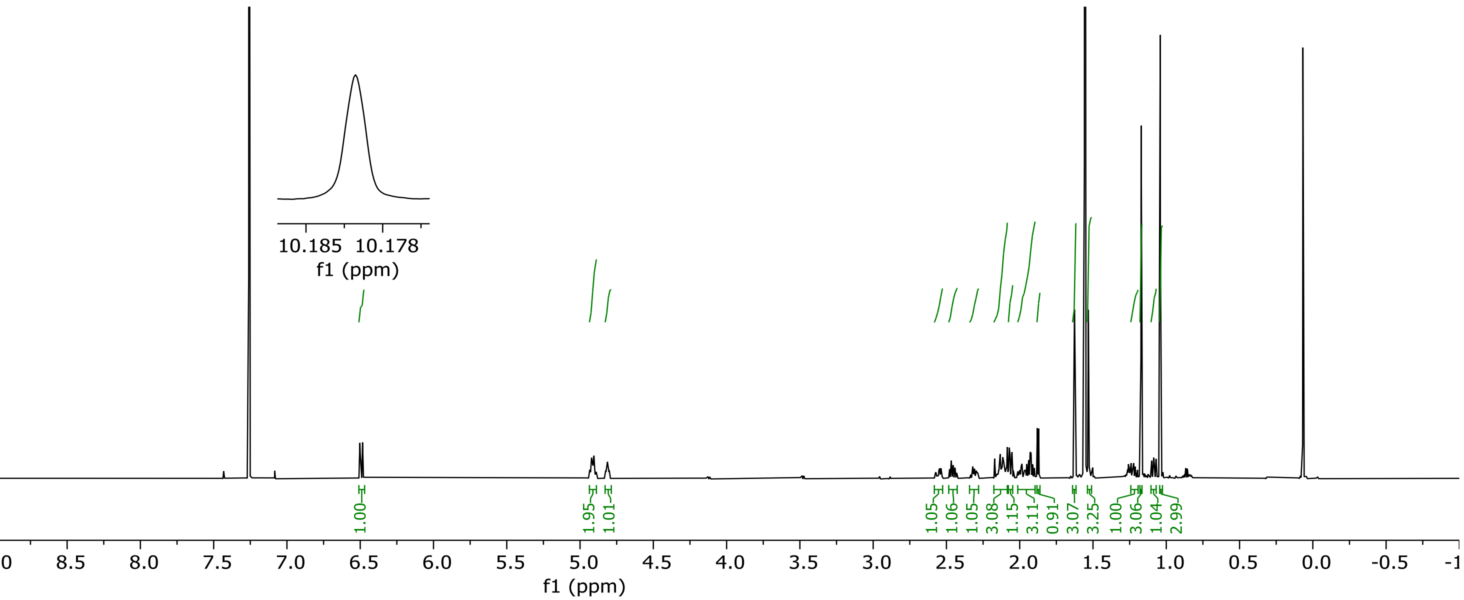

10.185 10.178
f1 (ppm)
0.94
1.00
1.95
1.01
1.05
1.06
1.05
3.08
1.15
3.11
0.91
3.07
3.25
1.00
3.06
1.04
2.99
10.5 10.0 9.5 9.0 8.5 8.0 7.5 7.0 6.5 6.0 5.5 5.0 4.5 4.0 3.5 3.0 2.5 2.0 1.5 1.0 0.5 0.0 -0.5
f1 (ppm)

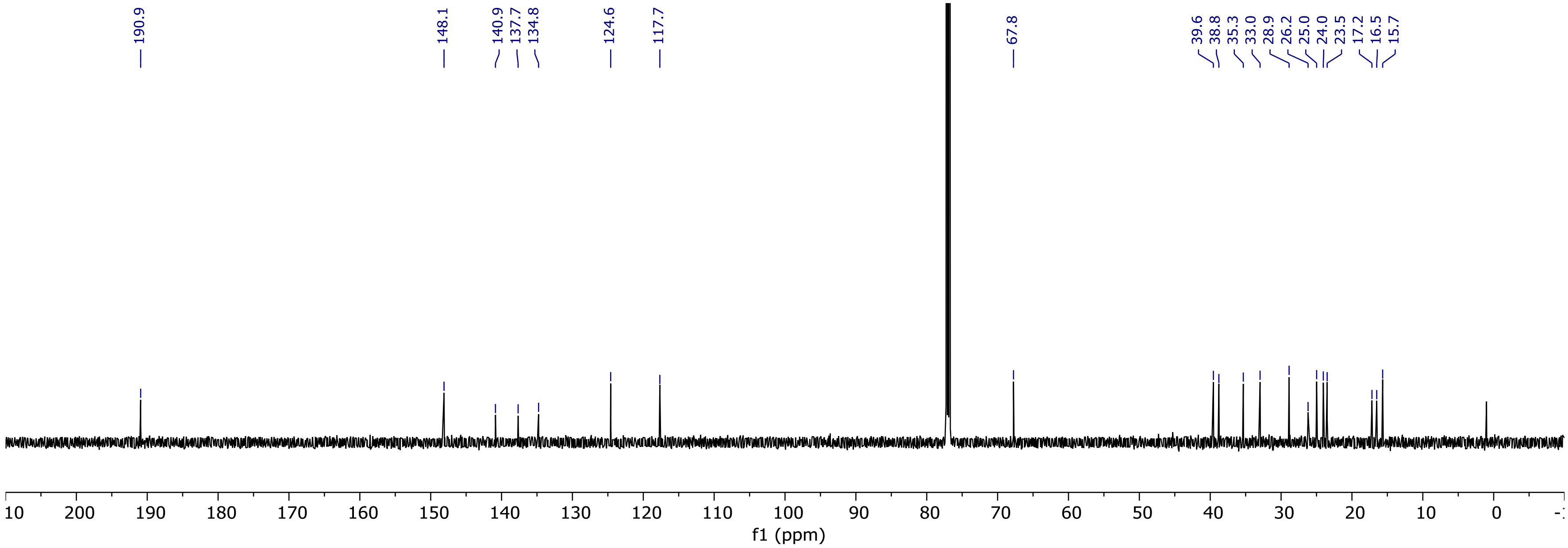

190.9
148.1
140.9
137.7
134.8
124.6
117.7
67.8
39.6
38.8
35.3
33.0
28.9
26.2
25.0
24.0
23.5
17.2
16.5
15.7
200 190 180 170 160 150 140 130 120 110 100 90 80 70 60 50 40 30 20 10 0
f1 (ppm)

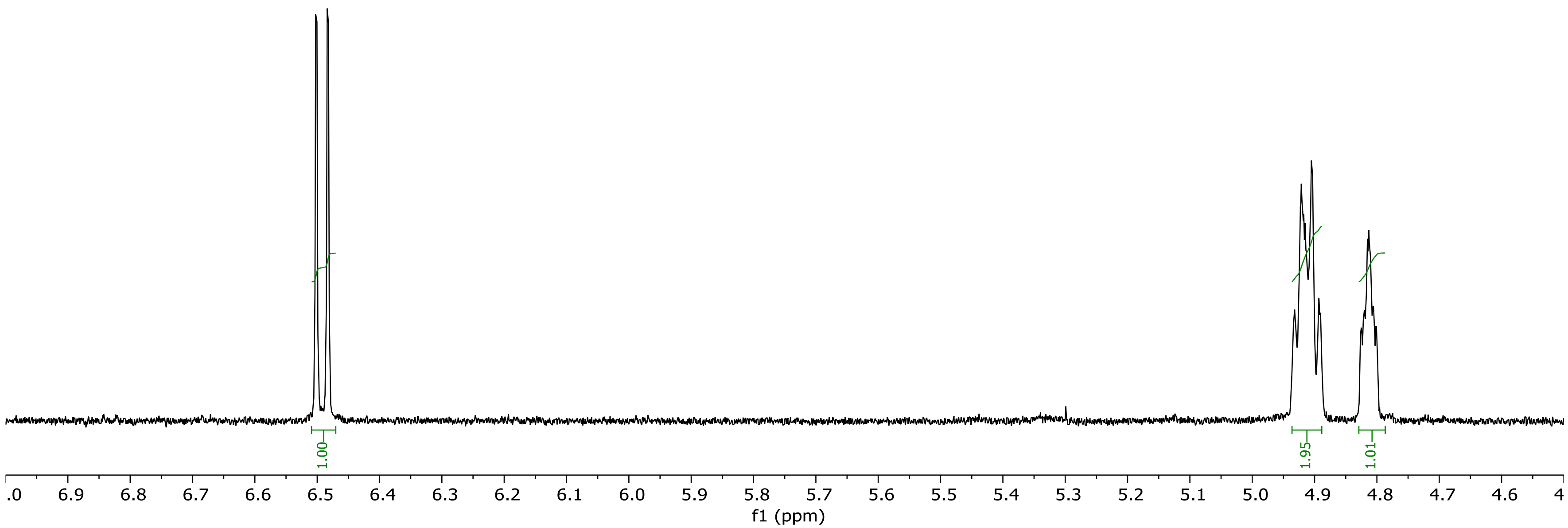
1.00
1.95
1.01
.0 6.9 6.8 6.7 6.6 6.5 6.4 6.3 6.2 6.1 6.0 5.9 5.8 5.7 5.6 5.5 5.4 5.3 5.2 5.1 5.0 4.9 4.8 4.7 4.6 4
f1 (ppm)

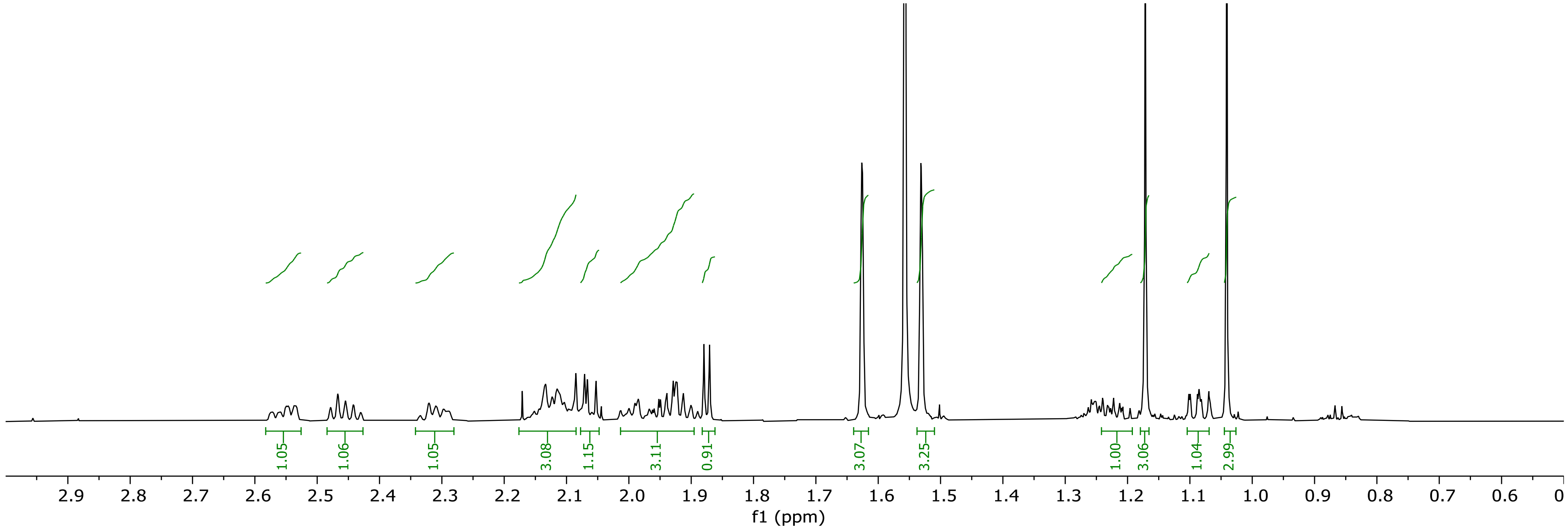
1.05
1.06
1.05
3.08
1.15
3.11
0.91
3.07
3.25
1.00
3.06
1.04
2.99
2.9 2.8 2.7 2.6 2.5 2.4 2.3 2.2 2.1 2.0 1.9 1.8 1.7 1.6 1.5 1.4 1.3 1.2 1.1 1.0 0.9 0.8 0.7 0.6 0
f1 (ppm)

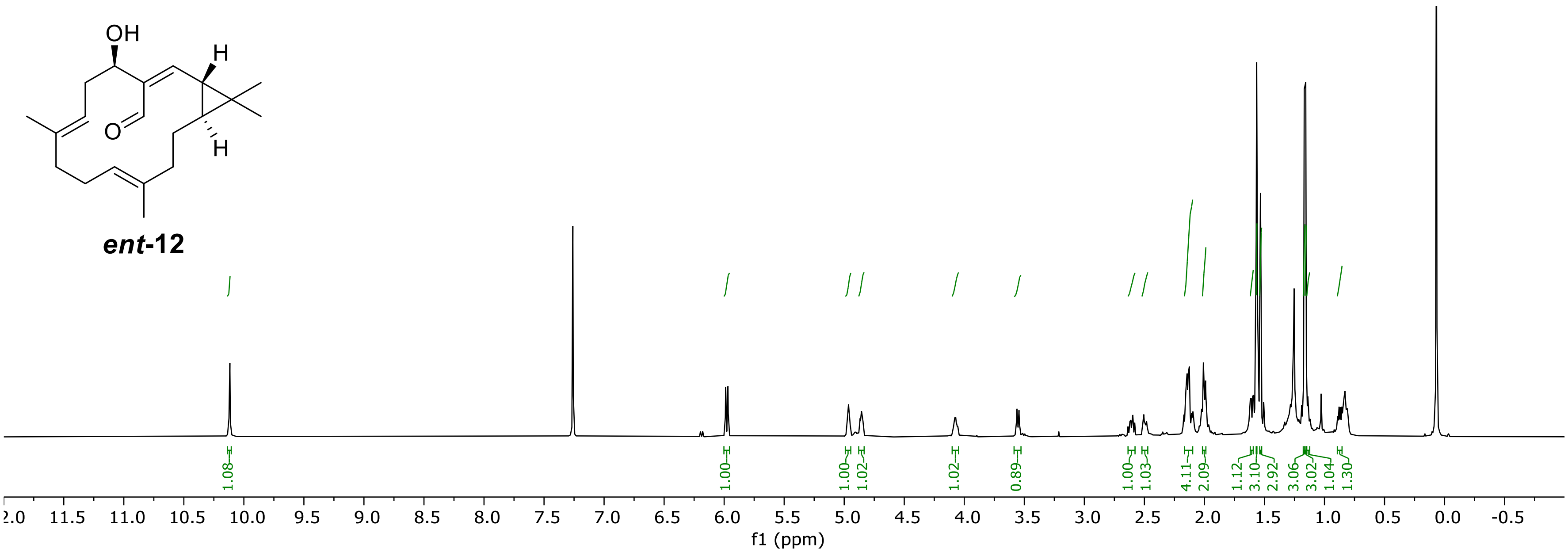
OH
H
O
H
1.08
1.00
1.00
1.02
1.02
0.89
1.00
1.03
4.11
2.09
1.12
3.10
2.92
3.06
3.02
1.04
1.30
f1 (ppm)

***ent*-12**

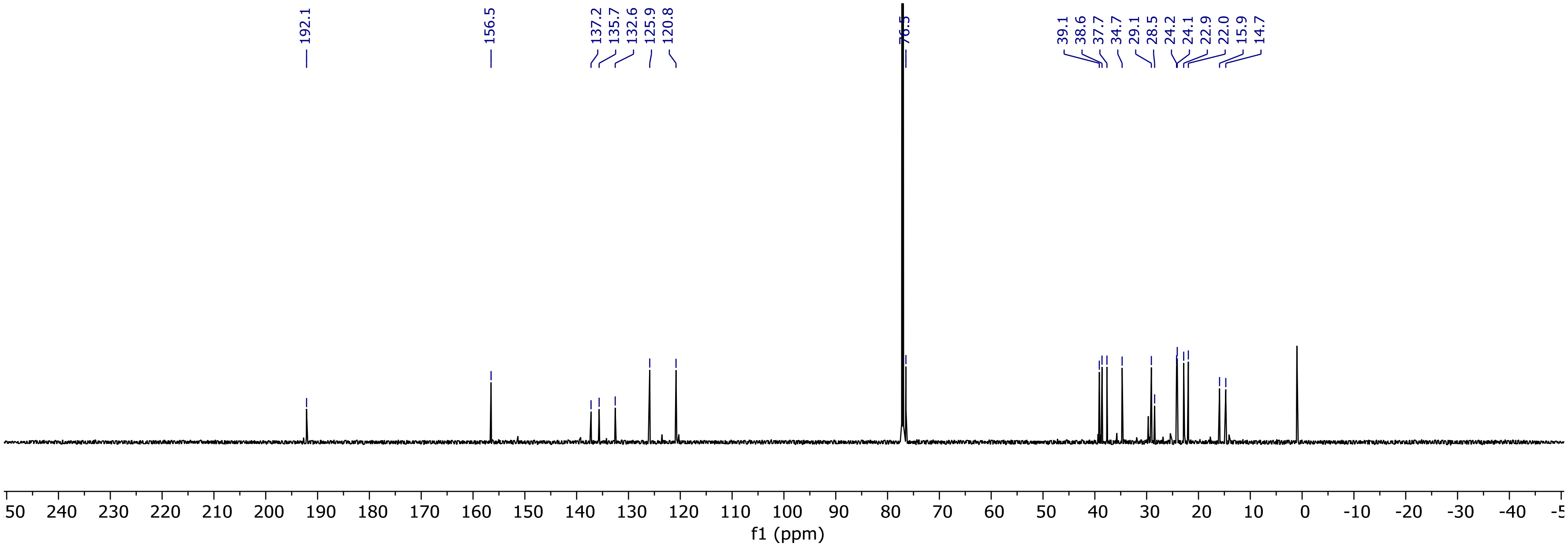
192.1
156.5
137.2
135.7
132.6
125.9
120.8
76.5
39.1
38.6
37.7
34.7
29.1
28.5
24.2
24.1
22.9
22.0
15.9
14.7
f1 (ppm)

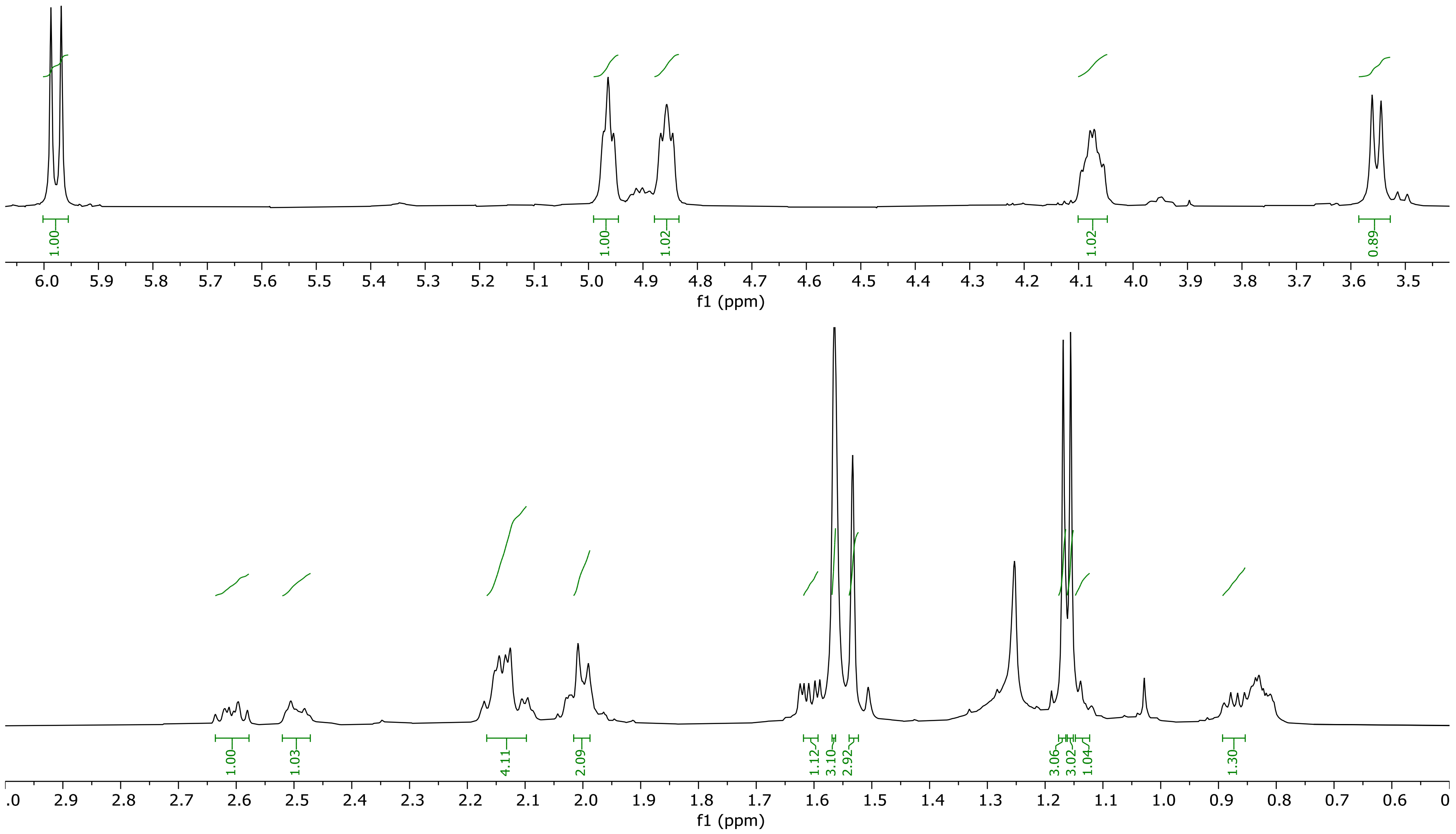
1.00
1.00
1.02
1.02
0.89
6.0 5.9 5.8 5.7 5.6 5.5 5.4 5.3 5.2 5.1 5.0 4.9 4.8 4.7 4.6 4.5 4.4 4.3 4.2 4.1 4.0 3.9 3.8 3.7 3.6 3.5
f1 (ppm)
1.00
1.03
4.11
2.09
1.12
3.10
2.92
3.06
3.02
1.04
1.30
.0 2.9 2.8 2.7 2.6 2.5 2.4 2.3 2.2 2.1 2.0 1.9 1.8 1.7 1.6 1.5 1.4 1.3 1.2 1.1 1.0 0.9 0.8 0.7 0.6 0
f1 (ppm)

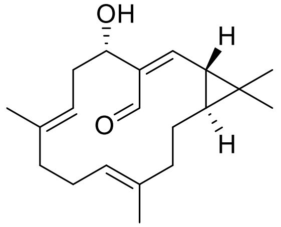
OH
H
O
H

44

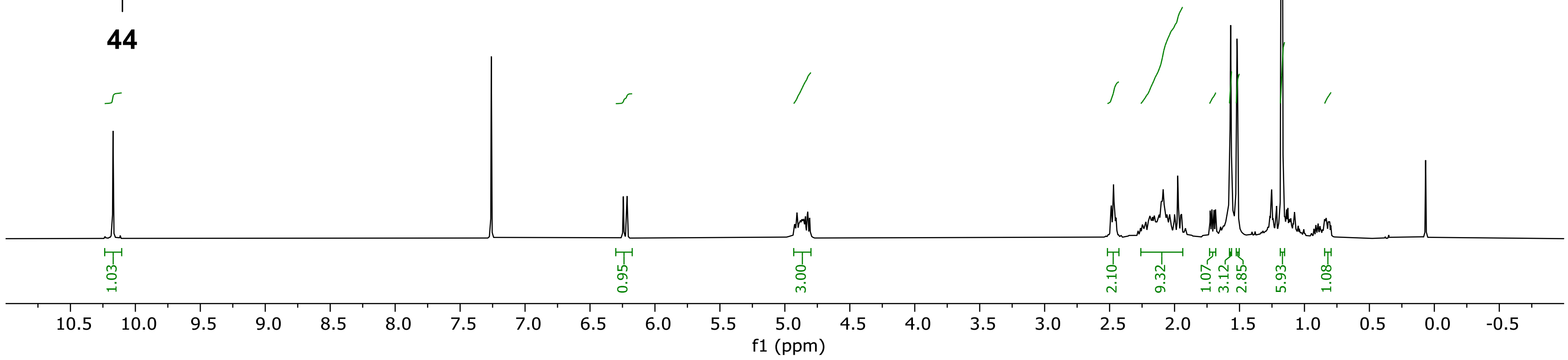
1.03
0.95
3.00
2.10
9.32
1.07
3.12
2.85
5.93
1.08
f1 (ppm)

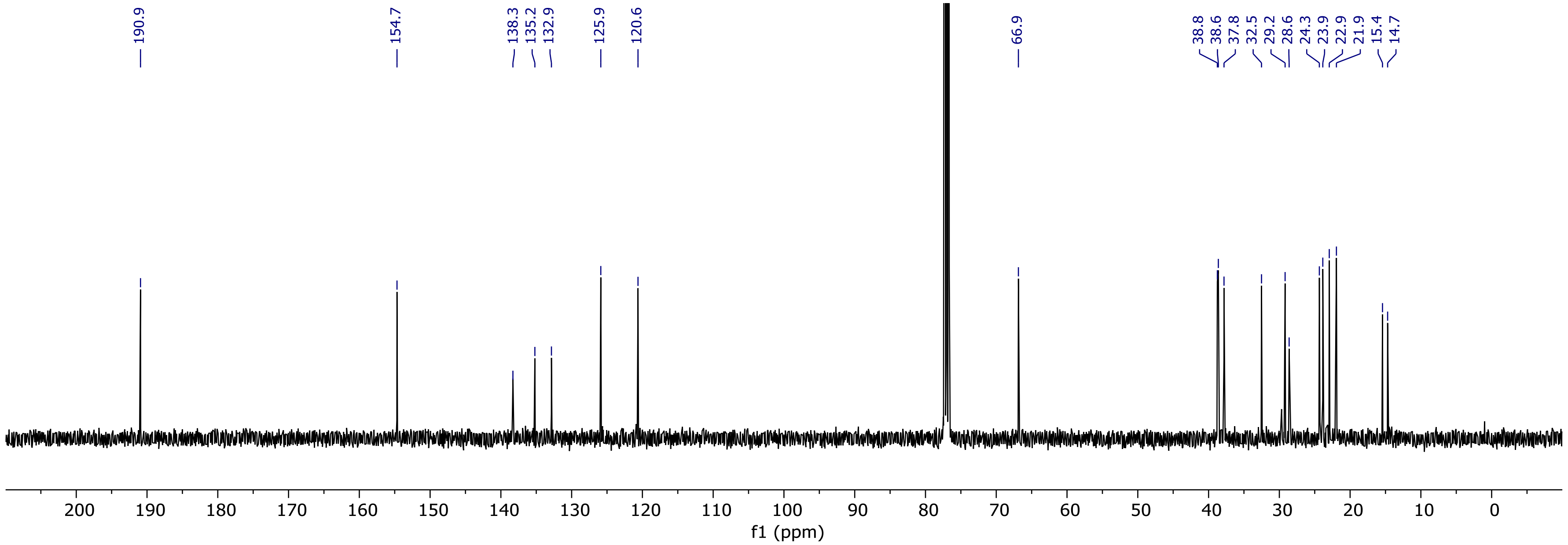
190.9
154.7
138.3
135.2
132.9
125.9
120.6
66.9
38.8
38.6
37.8
32.5
29.2
28.6
24.3
23.9
22.9
21.9
15.4
14.7
f1 (ppm)

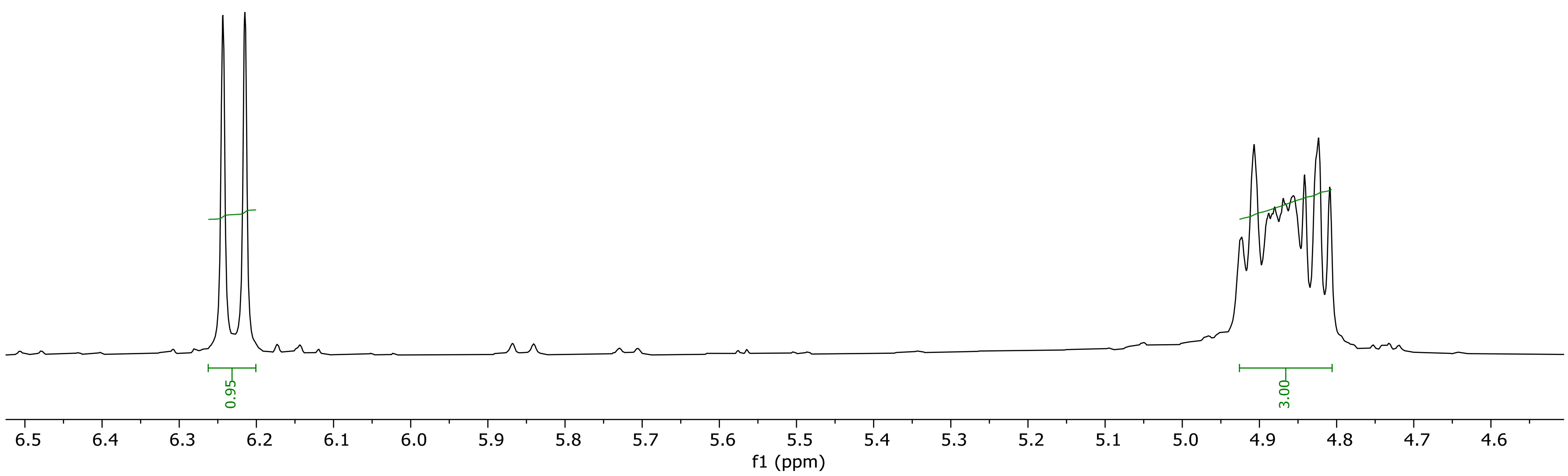
0.95
3.00
6.5
6.4
6.3
6.2
6.1
6.0
5.9
5.8
5.7
5.6
5.5
5.4
5.3
5.2
5.1
5.0
4.9
4.8
4.7
4.6
f1 (ppm)

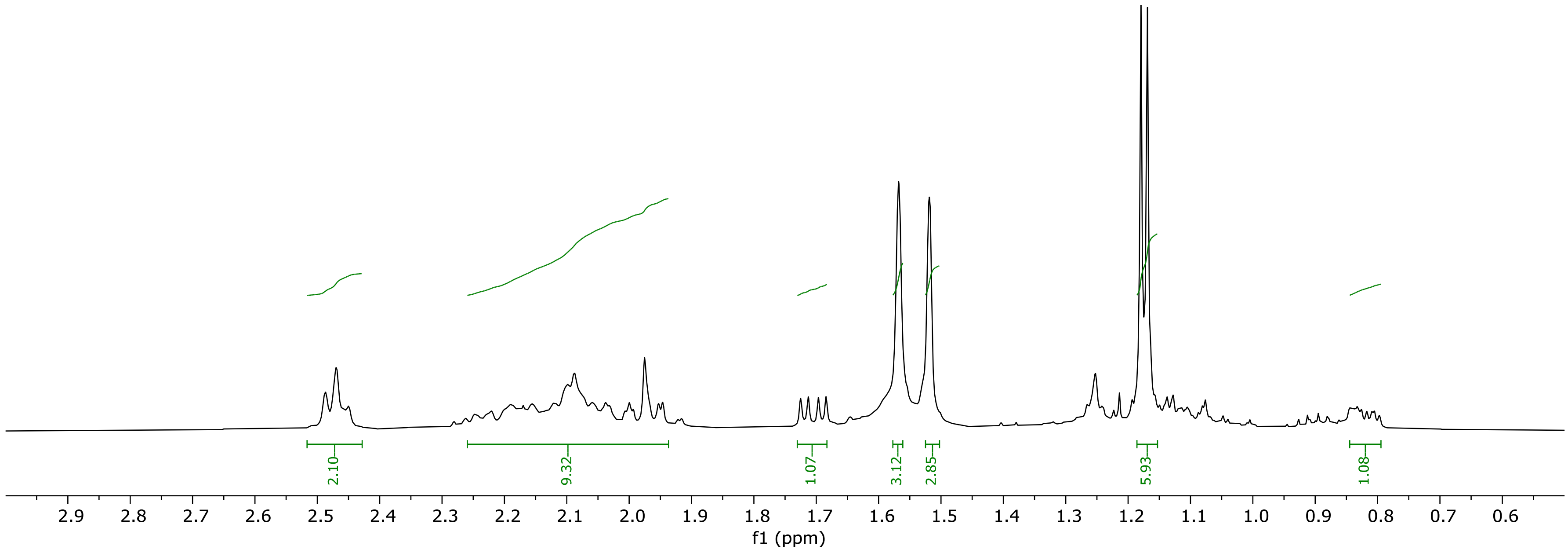
2.10
9.32
1.07
3.12
2.85
5.93
1.08
2.9
2.8
2.7
2.6
2.5
2.4
2.3
2.2
2.1
2.0
1.9
1.8
1.7
1.6
1.5
1.4
1.3
1.2
1.1
1.0
0.9
0.8
0.7
0.6
f1 (ppm)

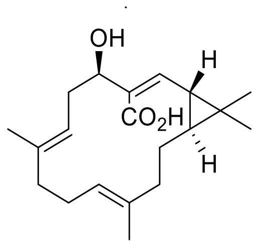
OH
H
CO2H
H

***ent*-14**

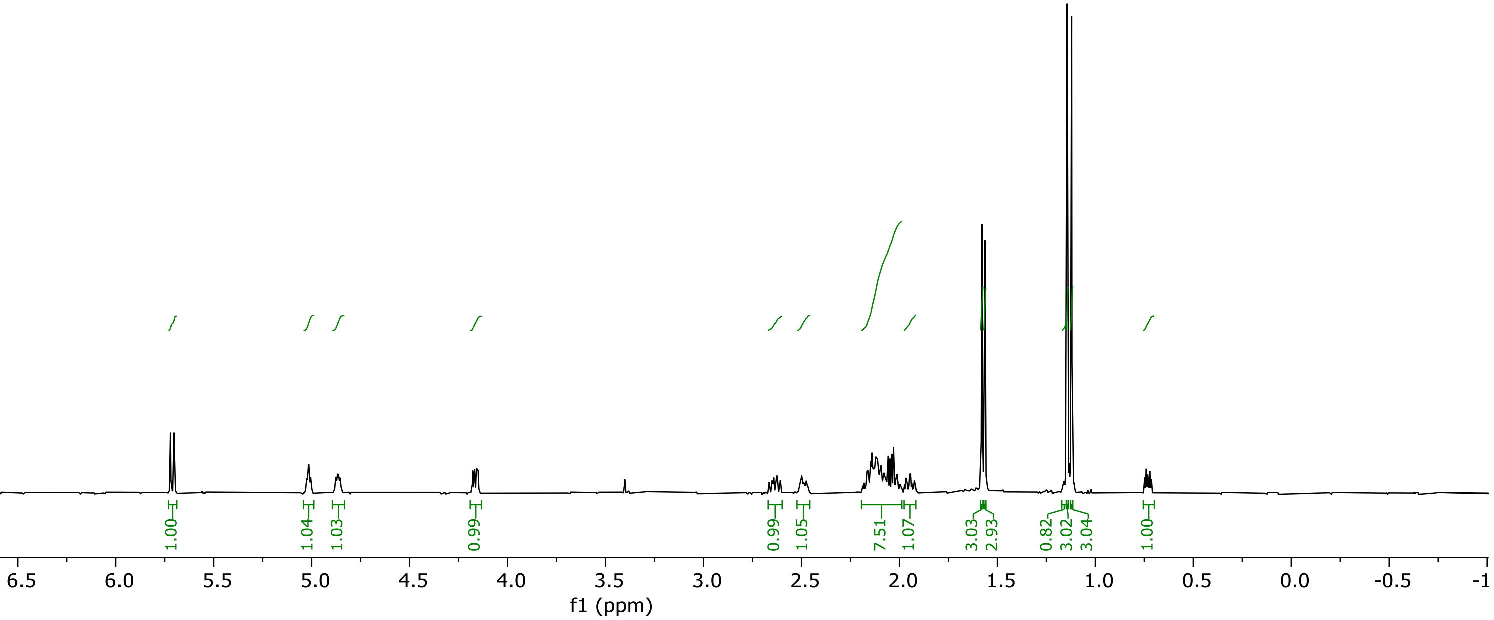
1.00
1.04
1.03
0.99
0.99
1.05
7.51
1.07
3.03
2.93
0.82
3.02
3.04
1.00
f1 (ppm)

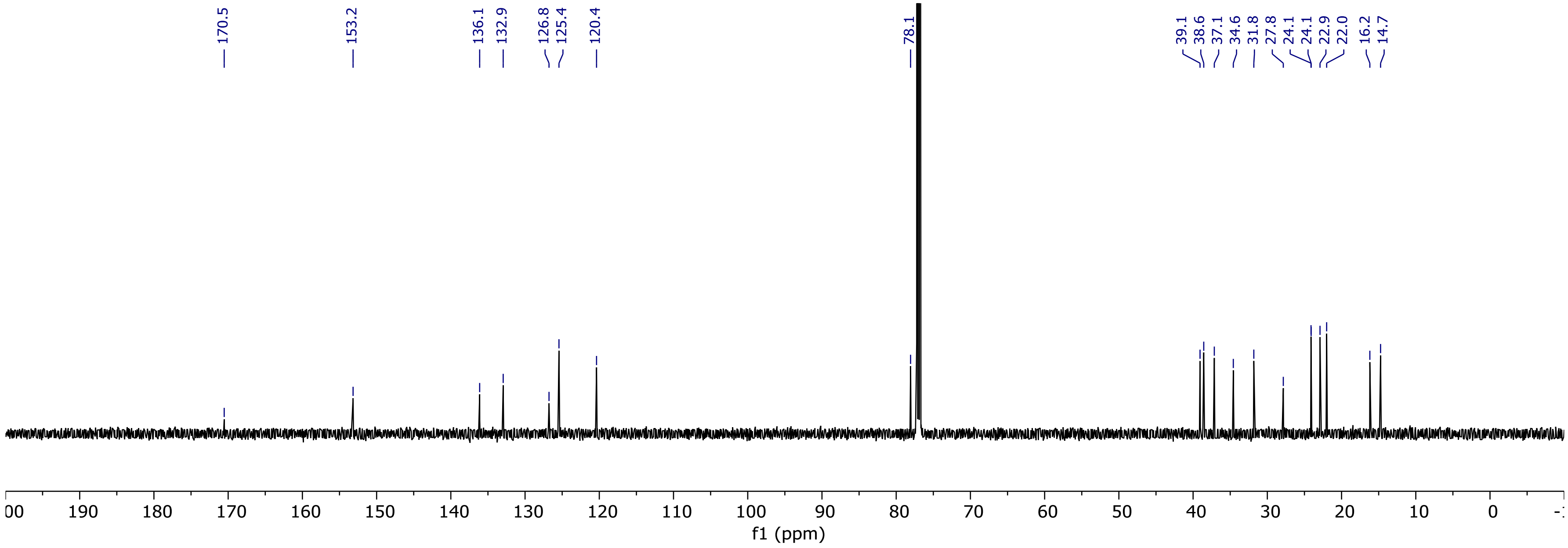
170.5
153.2
136.1
132.9
126.8
125.4
120.4
78.1
39.1
38.6
37.1
34.6
31.8
27.8
24.1
24.1
22.9
22.0
16.2
14.7
f1 (ppm)

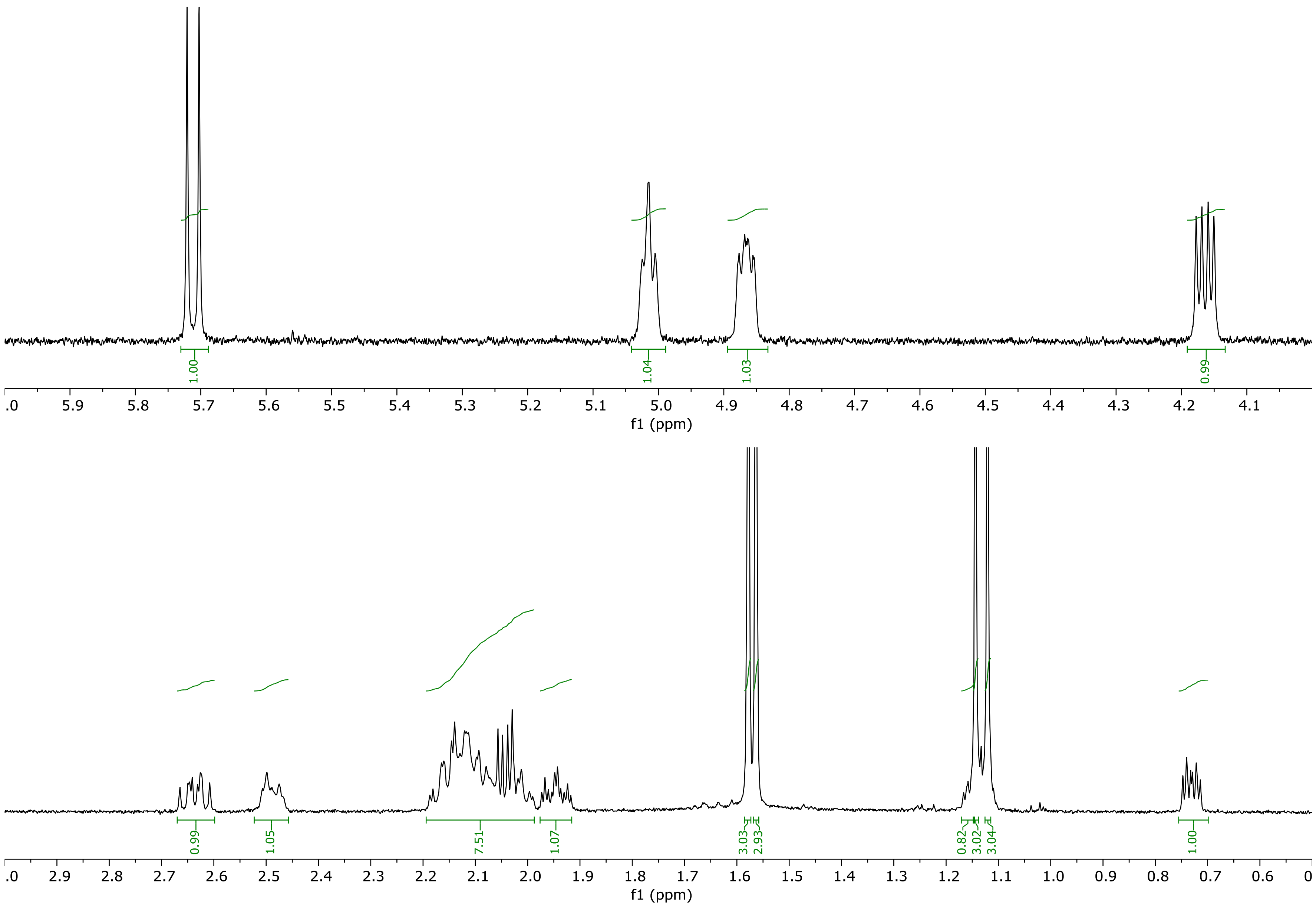
1.00
1.04
1.03
0.99
f1 (ppm)
0.99
1.05
7.51
1.07
3.03
2.93
0.82
3.02
3.04
1.00
f1 (ppm)
